# A foundation systematic review of natural language processing applied to gastroenterology & hepatology

**DOI:** 10.1186/s12876-025-03608-5

**Published:** 2025-02-06

**Authors:** Matthew Stammers, Balasubramanian Ramgopal, Abigail Owusu Nimako, Anand Vyas, Reza Nouraei, Cheryl Metcalf, James Batchelor, Jonathan Shepherd, Markus Gwiggner

**Affiliations:** 1 University Hospital Southampton, Tremona Road, Southampton, SO16 6YD UK; 2 Southampton Emerging Therapies and Technologies (SETT) Centre, Southampton, SO16 6YD UK; 3 Clinical Informatics Research Unit (CIRU), Coxford Road, Southampton, SO16 5AF UK; 4 University of Southampton, Southampton, SO17 1BJ UK; 5 Queen’s Medical Centre, ENT Department, Nottingham, NG7 2UH UK; 6 School of Healthcare Enterprise and Innovation, University of Southampton, University of Southampton Science Park, Enterprise Road, Chilworth, Southampton, SO16 7NS UK; 7 Southampton Health Technologies Assessment Centre (SHTAC), Enterprise Road, Alpha House, Southampton, SO16 7NS England

**Keywords:** Colonoscopy, Inflammatory bowel disease, Hepatocellular carcinoma, Gastroscopy, Pancreatic disease, Natural language Processing

## Abstract

**Objective:**

This review assesses the progress of NLP in gastroenterology to date, grades the robustness of the methodology, exposes the field to a new generation of authors, and highlights opportunities for future research.

**Design:**

Seven scholarly databases (ACM Digital Library, Arxiv, Embase, IEEE Explore, Pubmed, Scopus and Google Scholar) were searched for studies published between 2015 and 2023 that met the inclusion criteria. Studies lacking a description of appropriate validation or NLP methods were excluded, as were studies ufinavailable in English, those focused on non-gastrointestinal diseases and those that were duplicates. Two independent reviewers extracted study information, clinical/algorithm details, and relevant outcome data. Methodological quality and bias risks were appraised using a checklist of quality indicators for NLP studies.

**Results:**

Fifty-three studies were identified utilising NLP in endoscopy, inflammatory bowel disease, gastrointestinal bleeding, liver and pancreatic disease. Colonoscopy was the focus of 21 (38.9%) studies; 13 (24.1%) focused on liver disease, 7 (13.0%) on inflammatory bowel disease, 4 (7.4%) on gastroscopy, 4 (7.4%) on pancreatic disease and 2 (3.7%) on endoscopic sedation/ERCP and gastrointestinal bleeding. Only 30 (56.6%) of the studies reported patient demographics, and only 13 (24.5%) had a low risk of validation bias. Thirty-five (66%) studies mentioned generalisability, but only 5 (9.4%) mentioned explainability or shared code/models.

**Conclusion:**

NLP can unlock substantial clinical information from free-text notes stored in EPRs and is already being used, particularly to interpret colonoscopy and radiology reports. However, the models we have thus far lack transparency, leading to duplication, bias, and doubts about generalisability. Therefore, greater clinical engagement, collaboration, and open sharing of appropriate datasets and code are needed.

**Supplementary Information:**

The online version contains supplementary material available at 10.1186/s12876-025-03608-5.

## Key Messages

• What is already known on this topic–*NLP can accurately detect polyp mentions in colonoscopy reports; however, no systematic review has yet been performed across clinical gastroenterology and hepatology.*

• What this study adds–*An overview of NLP applied to gastroenterology up to 2023 highlighting areas of current strength and opportunities for future focus in the age of the large-language model.*

• How this study might affect research, practice, or policy—*This study helps inform future priorities for NLP research in Gastroenterology and Hepatology while focusing on increased transparency and bias reduction within the field*

**Supplementary Information:**

The online version contains supplementary material available at 10.1186/s12876-025-03608-5.

## Introduction

Electronic healthcare records (EHRs) contain a rich collection of real-world clinical data that can be used to improve the understanding of gastrointestinal diseases. Human clinicians cognitively process this information, organising it into contextualised chunks. This semistructured information presents particular challenges for computer analysis because morphology (how words are formed), syntax (the arrangement of words), semantics (the meaning of words and phrases) and pragmatics (how language is used) [1] vary depending on the context.

**Fig. 1:**
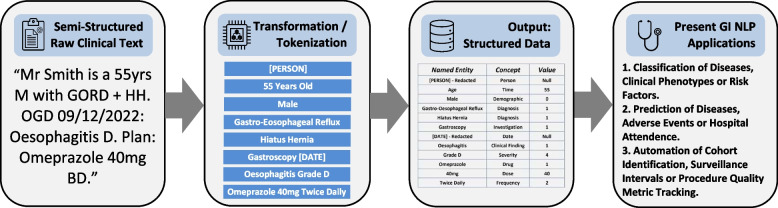
Applied Example of Natural Language Processing in Gastroenterology. Figure 1 provides a visual applied example of clincial natural langugage processing (NLP) in gastroenterology flowing from semi-structured free-text to transformed data, then on to structured output and finally some examples of present gastroenterology(GI) NLP applications

Natural language processing (NLP) describes computerised methods for assessing, evaluating, synthesising, generating, and interacting with free text. A spectrum of NLP technologies exists, ranging from rule-based (RB) methods to machine learning (ML) and deep learning (DL) methods [2]. The field accelerated with the advent of DL-based transformer models in 2017 [3]. Many NLP models can now interpret complex language in clinical text to help structure clinical information. (Fig. 1)

DL methods have the advantage of coping with larger volumes of data, typically at the cost of explainability. In particular, bidirectional encoder representations from transformers (BERT) models [4] and generative pre-trained transformers such as GPT-3 in 2020 [5], which were subsequently used to perform a literature review [6], have improved the profile and capabilities of clinical NLP. In contrast, RB methods often work well with smaller datasets but are more challenging to scale.

Moreover, the rapid ongoing expansion in demand for gastrointestinal services worldwide [7–11] is leading to intense and building pressures on the workforce [12, 13]. NLP is already used in other specialties to semi-automate clinical workloads. However, as in radiology, significant involvement is needed by both researchers and healthcare professionals to ensure that these methods are trustworthy [14], robust and representative.

Researchers are increasingly using NLP in gastroenterology [15], as recently described in a systematic review studying NLP adenoma detection from free-text colonoscopy reports [16]. Future clinical applications include diagnostic decision-making, referral classification, prediction of disease progression, clinical error flagging and personalised treatment planning.

Applying NLP in gastroenterology also presents some specific challenges. Most gastroenterological diagnoses, such as inflammatory bowel disease (IBD), can be diagnosed at multiple levels: histopathological, endoscopic and clinical. Thus, standalone algorithms based on singular reports will prove clinically insufficient. Although often semi-structured, liver, pancreatic, and endoscopic reports may vary substantially in content. Finally, neuro-gastroenterological problems are still open to some subjective interpretation, making NLP analysis incredibly challenging.

However, as a starting point, a general overview of the field is required to accelerate future progress. By learning from recent examples in radiology [17], cardiology [18] and psychiatry [19], this systematic review aimed to provide clinicians with an accessible understanding of NLP.

### Aim

This review assesses the progress of NLP within gastroenterology, grades the robustness of the methodology, exposes the field to a new generation of authors, and highlights future opportunities for clinical usage and recommendations for research.

## Methods

The review was registered on PROSPERO [20] as an original protocol in January 2023, with prespecified criteria published beforehand to minimise bias while assessing RB and ML NLP in gastroenterology.

### Article retrieval

This review follows the Preferred Reporting Items for Systematic Reviews and Meta-Analyses (PRISMA) guidelines [21] (Supplementary Material 1) for reporting in systematic reviews and the AMSTAR checklist [22]. Because it is well known that information specialists best develop search strategies [23], a medical librarian was involved in developing the search strategy for this review. The Peer Review of Electronic Search Strategies (PRESS) checklist [24] was used for this process, and the Transparent Reporting of a Multivariate Prediction Model for Individual Prognosis or Diagnosis checklist (TRIPOD) checklist [25] was used to rate the methodological robustness of all the prediction studies. When a meta-analysis was impossible, the Synthesis Without Meta-analysis (SWiM) guidelines [26] were used to maximise reporting robustness. The adapted Risk of Bias in Nonrandomized Studies – of Interventions (ROBINS-I) [27] checklist was used to assess the risk of bias (ROB) in primary studies. Further details of the checklist are provided in Supplementary Material 3.

Articles were searched for in seven scholarly databases covering medicine and computer science, namely, the ACM Digital Library, Arxiv, Embase, IEEE Explore, PubMed, Scopus and Google Scholar, between 1/1/2015 and 1/1/2023, available in the English language. Articles published in abstract form before 2023 were included. The year 2015 was selected as the starting year for this review because it covers the climax of the era of RB methods through the age following the discovery of the attention mechanism [3], which transformed the field and allowed for part self-supervised DL in clinical NLP.

A combination of search terms relating to NLP and gastroenterology was selected based on the Medical Subject Headings vocabulary (U.S. National Library of Medicine) with additional terms identified from prior NLP-focused reviews, in particular the work of Nehme et al. [15] who also collaborated with a medical information specialist. Extensive details of the search strategy are provided in Supplementary Material 2.

### Study selection

We used Covidence, a specialist software package, to manage the production of this systematic review (www.covidence.org) [28]. The studies considered eligible were those in which NLP algorithms were used to assess clinical free text for (1) diagnosis, (2) investigation, (3) treatment, (4) monitoring and (5) management of gastrointestinal diseases. RB, ML, and DL algorithms were included, but only those featuring Type 2a validation or higher, as TRIPOD [25] specified, because Type 1b validation or less is associated with unacceptable ROB in prediction/classification studies—Table 1.

**Table 1:** TRIPOD Model Validation Hierarchy

*Level of Validation*	*Study Type*
Type 1a	Development Only
Type 1b	Development and Validation Using Resampling
Type 2a	Random Split-Sample Development and Validation
Type 2b	Nonrandom split Sample Development and Validation performed robustly, allowing nonrandom variations between datasets
Type 3	Development and Validation Using Separable Data
Type 4	Validation Only

Duplicate references and studies lacking a description of NLP methods and focusing only on gastrointestinal disease risk factors were also excluded.

Following this strategy, three reviewers (MS, AV, AO) performed two rounds of independent study selection, with titles and abstracts screened in the first round and full texts reviewed in the second round. Disagreements between review authors over the eligibility of studies were resolved by a senior review author (MG). Agreement between reviewers was measured using Cohen’s kappa statistic, with values above 0.8 indicating excellent agreement and above 0.6 indicating good agreement.

### Data extraction and synthesis

Data from each included article were independently extracted by two reviewers (MS, BR), and discrepancies were resolved through discussion. The extracted data included general study information (design, objectives), clinical details (clinical subarea, patient characteristics), and natural language processing (NLP) details (methods, evaluation metrics and results). To reduce complexity, evaluation metrics were reported for primary study outcomes only and given as ranges when performance metrics for multiple cohorts or methods were reported separately. Where the primary outcome measure was not explicitly stated, an attempt was made to infer this from the study's aims. All the reviewers worked with the same understanding of the standard NLP terms and methods described in Table 2.

**Table 2:** Glossary of Core Terms and Metrics

*Computer Science Terms*	*Models and Methods*
Natural Language Processing (NLP)	Natural Language Processing describes a set of techniques which allow computers to extract meaning from semistructured textual information
Electronic Health Record (EHR)	Electronic Health Record. Software which manages patient and clinical records in typically either a hospital or primary care setting
Model	A representation of a problem or solution typically in the form of numbers with an underlying structure/architecture
Rule-Based (RB)	Use of an established set of rules or logic to define a search pattern, which is then executed deterministically
Machine-learning (ML)	Semiautomated learning from data using stochastic (~ randomness) models, which vary from well-known statistical models such as logistic regression to ‘deeper’ models such as XGBoost/Random Forest typically to make a prediction
Deep Learning (DL)	Computational imitation of human neural networks. It can be used to overcome some of the limitations of more traditional machine learning models, detecting more subtle or ‘deeper’ patterns hidden in the data to make predictions
Decision tree (DT)	A form of ML model where branching logic is utilized to make decisions by splitting on criteria thresholds. Simple and easy to understand
Logistic regression (LR)	Classification variant of linear regression. Often, it copes reasonably well with limited data but cannot cope with significant interactions between data points
Random forest (RF)	An ‘ensemble’ of decision trees is built to create a forest of DTs. The forest can better cope with complexities within the data at a cost to explainability
*Evaluation Methods*	
Manual annotation	Human annotation of concepts of interest or human marking/classification of documents
Cross-validation (CV)	A technique to evaluate predictive models by partitioning the original sample into a training set to train the model and a test set to evaluate it with reduced risk of overfitting/bias
Holdout Set	A section or part of the data is withheld from the model training process for testing only
*Performance Metrics*	
Accuracy	The percentage of results that were correct among all results from the system. Calc: (TP + TN)/(TP + FP + TN + FN)
Precision (PPV)	Also called positive predictive value (PPV). The percentage of true positive results among all results that the system flagged as positive. Calc: TP/(TP + FP)
Negative Predictive Value (NPV)	The percentage of results that were true negative (TN) among all results that the system flagged as negative. Calc: TN/(TN + FN)
Recall	Also called sensitivity. The percentage of results flagged positive among all results should have been obtained. Calc: TP/(TP + FN)
Specificity	The percentage of results that were flagged negative among all negative results. Calc: TN/(TN + FP)
F1-Score	The harmonic mean of PPV/precision and sensitivity/recall, in this case unweighted. Calc: 2 × (Precision x Recall)/(Precision + Recall)
Area Under the Curve (AUC)	Typically, it relies on a receiver-operator curve and is synonymous with AUROC – this type of AUC we refer to in this review. It acts as a measure of model predictive capture, with 0.9 being a strong predictive model and 0.6 weak

*Abbreviations: TP *True Positive*, FP *False Positive*, FN *False Negative*, *TP *True Negative*

Specifically, accuracy, precision, recall and harmonic mean (F1-score) were extracted for each study where available. Additional data extracted are described in the published protocol [20]. Synthesis was performed without meta-analysis as per SWiM.

### Quality appraisal of study quality, reporting and risk of bias

Relevant reporting standards specific to NLP research have yet to be established. Therefore, a modified quality appraisal based on the approach described by Koleck and colleagues [29], which has been used successfully in cardiology [18], was combined with additional machine-learning quality indicators, as defined by Nascimento [30]. This checklist included evaluations of tuning, generalisability, use of appropriate statistical tests, model costs (time), potential for explainability, code sharing and documentation. The adequacy of the reporting was assessed according to the principles of SwiM [26] by two review authors (MS, BR), who also independently assessed quality and ROB as high or low according to an adapted ROBINS-I and Cochrane Specification [27, 31] available in Supplementary Material 3. QUADAS-2 [32] was not used because of its narrow scope. As internationally recognised NLP benchmarks are established, standardised clinical NLP ROB frameworks will hopefully become formal.

Multiple checklists are used in this project to maximise the robustness of the approach in an emergent and currently somewhat heterogeneous field. For clarity, they are summarised below in Table 3 with their associated purpose within the review.

**Table 3:** Checklist Summary

*Checklist*	***Purpose Within Review***
PRISMA	Provides a standardised framework for reporting the systematic review, ensuring clarity, transparency, and replicability as well as an understanding of the numbers of papers screened.
AMSTAR	Evaluates the methodological quality and rigour of the systematic review, ensuring the reliability of study findings and reporting these faithfully.
TRIPOD	Guides the transparent reporting of prediction models, covering development, validation, and evaluation aspects. In this review, particular attention is paid to the validation component of TRIPOD which has the greatest bearing on model generalisability.
SWiM	Supports structured synthesis and reporting systematic reviews that do not include meta-analyses. In this study an adapted version of the quality checklists developed by Koleck [29] and Nascimento [30] were used.
ROBINS-I	Assesses the risk of bias in interventional studies, ensuring the validity of their findings. In this review, the checklist has been adapted for NLP studies in Gastroenterology.

## Results

### Article screening

**Fig. 2:**
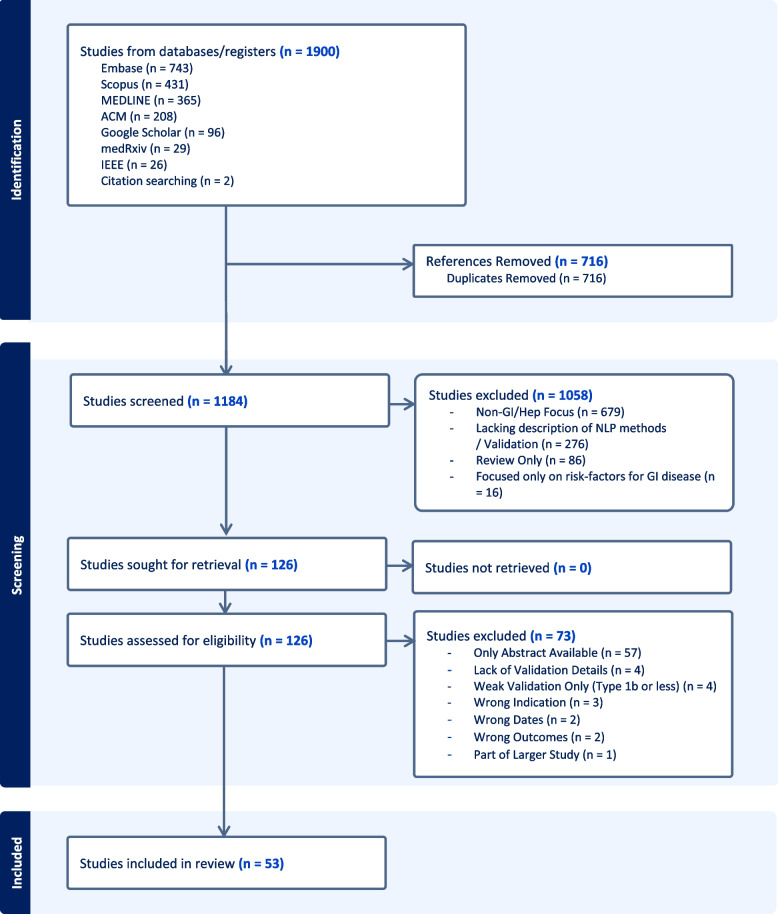
PRISMA Flow Diagram For Review. Figure 2 provides the full PRISMA flow diagram for the study from abstract identification and screening through to full paper screening and extraction

After applying the eligibility criteria, 53 articles were included in the review (Fig. 2). A total of 1900 studies were initially retrieved from scholarly databases; however, 716 (39.6%) of these studies were removed as duplicates. Of the 1184 unique references screened by title and abstract, 679 (57.3%) were excluded for not having a gastrointestinal focus, and 276 (23.3%) were excluded for not using NLP or describing NLP methods or validation. Eighty-six (7.3%) articles were reviews only, and 16 (1.4%) articles focused only on gastrointestinal disease risk factors. See Supplementary Material 10 for details of all abstracts screened and Supplementary Material 6 for interobserver agreement results during screening. A full PRISMA flow diagram is provided in Fig. 2.

During the full-text screening 126, studies were excluded because they were available only in abstract form 57 (45.2%), performed only weak validation 4 (3.2%) or did not provide sufficient details about NLP methods or validation 4 (3.2%). A total of 3 (2.4%) studies were excluded due to irrelevant indications (limited gastroenterology focus), 2 (1.6%) were first published outside the date range, 2 (1.6%) were focused primarily on reviewing the literature, and one (0.8%) study was a substudy focused on consensus building. See Supplementary Material 9 for the full details of the excluded studies.

### Key characteristics of the included studies

Of the 53 included studies, 29 (54.7%) were published in biomedical informatics or computer science journals, 19 (35.8%) were published in gastroenterology clinical journals, and 5 (9.4%) were published in non gastroenterology-focused clinical journals.

A total of 18 (34.0%) studies were based on data from a single centre, and 35 (66.0%) were multisite or registry. Regarding technological maturity, 47 (88.7%) studies were performed in a development/laboratory environment. In comparison, 6 (11.3%) studies were launched as part of a clinical pilot, and only one (1.9%) was deployed as part of a production clinical human-in-the-loop system [33]. No systems are currently being used unsupervised in production.

In terms of clinical focus, 22 (41.5%) studies focused primarily on obtaining additional information from clinical investigations, 20 (37.8%) studies focused on detecting/extracting diagnoses, and 10 (18.9%) studies focused on improving the monitoring of a disease or calculating surveillance intervals. Only a single study (1.9%) focused on treatment/management [34].

The total number of documents available to investigators ranged from 101 [35] to 14.6 million [36], with up to 610,684 [37] individual patients in the available sample population. However, given the high costs involved in annotation, high-quality manually annotated model development document samples varied between 101 [35] and 6836 [38], and manually annotated validation document samples ranged from 100 [39] to 2988 [40] in size.

### Study tools/methods used

The authors used a wide array of methodologies/tools, including 26 (49.1%) studies using RB methods, 15 (28.3%) using a hybrid (ML + RB) approach, 10 (18.9%) using singular ML models and 2 (3.8%) using an ML ensemble [38, 41]. Popular established open-source tools utilised included CLAMP [42], cTAKES [43] and PyCONtext [44]/MedSpacy [45], with Python, n = 15 (28.3%) the most popular nonstructured query language explicitly mentioned, followed by Java, n = 10 (18.9%), Prolog 3 (5.7%) and PERL 1 (1.9%). Four commercial algorithms (I2E™, EHRead™, ClixNLP™ and EasyCIE™) were mentioned across 5 (9.4%) studies. Table 4 provides an overview of the primary open-source NLP tools described.

**Table 4:** Key NLP Tools Currently Used in Gastroenterology/Hepatology

Tool	Description	Link	Example Usage
Commonly Used Ontologies/Clinical Data Models			
ICD-10	WHO International Classification of Diseases version 10	https://icd.who.int/browse10/2010/en	Coding of gastroenterology diagnoses on discharge summaries as a validation standard
SNOMED-CT	SNOMED Clinical Terminology system	https://www.snomed.org/get-snomed	Coding of gastroenterology diagnoses on discharge summaries as a validation standard
UMLS Metathesaurus	Open-source compendium of controlled vocabularies curated by the US Library of Medicine	http://www.nlm.nih.gov/research/umls/	Standardisation of Free-Text terms to aid with tokenisation (breaking up) of free-text
OMOP	Observation of Medical Outcomes Partnership Common Data Model	https://www.ohdsi.org/data-standardization/	Mapping of clinical information to a standardised data model to aid interoperability
Java-Based Open-Source Tools			
cTAKES	Open-source NLP system for information extraction from electronic medical record clinical free text	http://ctakes.apache.org/	Used to process and extract concepts such as from free text
GATE	Suite of tools for NLP tasks, including information extraction	https://gate.ac.uk/	Used to extract concepts such as hepatitis from clinical free text
MALLET	Java-based package for statistical NLP, document classification, clustering, topic modelling and information extraction	http://mallet.cs.umass.edu/	Used to build a text-to-model pipeline, perhaps to diagnose IBD and perform NLP analysis on that model
CLAMP	Clinical Language Annotation, Modelling and Processing Toolkit	https://clamp.uth.edu/	Used to annotate clinical free-text, perhaps for training a model for diagnosis of pancreatic cysts in radiology reports
Python-Based Open-Source Tools			
NLTK	Python’s natural language processing toolkit	https://www.nltk.org/	Identify abdominal pain tokens in clinic letters
Spacy	Self-described as industrial-strength natural language processing in python	https://spacy.io/	Label patients with polyps with coloring and build a pipeline
MedSpacy	Successor to PyContextNLP combining the original implementation with Spacy	https://github.com/medspacy/medspacy	Build a fully functional app annotating endoscopy reports
Chexpert-labeler	Initially, developed to help label chest X-rays adapted in some studies to review CTs and MRIs	https://github.com/stanfordmlgroup/chexpert-labeler	Label radiology reports of patients with, for instance, pancreatic cysts

Substantial heterogeneity in study datasets, ontologies, tools, models, and methods makes direct comparisons between study methods extremely challenging. Only 4 studies provided code links, and only one used a publicly available dataset (MIMIC-II), substantially limiting replicability. These are highlighted in Supplementary Material 7 for reference.

### Demographics of the included studies

Only 30 (56.6%) of the studies reported patient demographics. Ages ranged from 16 [46] to 85 [47] years, while sex balance ranged from 1.8% [48] to 63% [49] female. Only 17 (32.1%) studies reported underlying ethnicity and detailed information on participant socioeconomic status or comorbidities was provided in only 5 (9.4%) studies. A full breakdown of the reported study populations is provided in Supplementary Material 7.

### Study purpose and primary findings

**Fig. 3:**
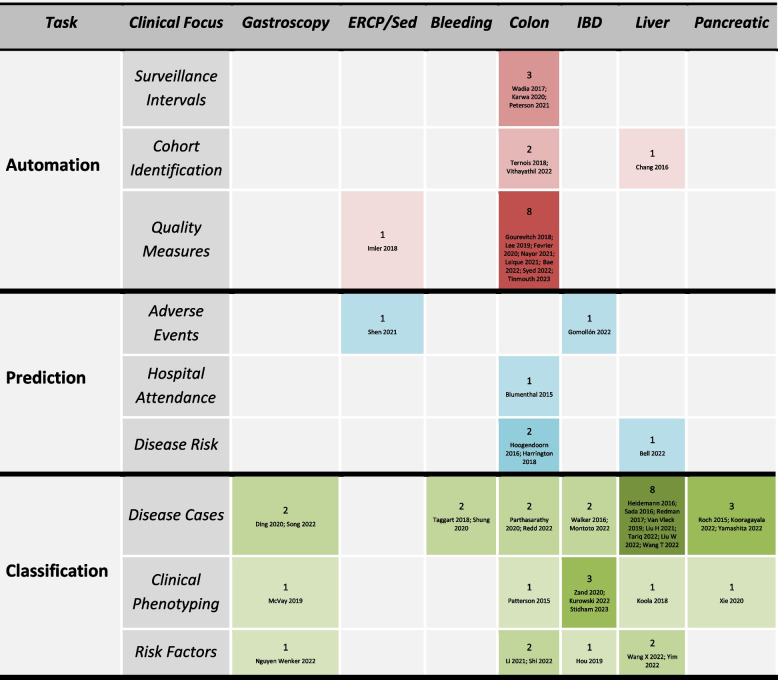
Distribution of Available NLP Studies across Gastroenterology and Hepatology. Figure 3 visually examines the distribution of available NLP studies across varied clinical, data science and task domains

Specifically, 21 (39.6%) of the studies focused on colonoscopy, 13 (24.5%) on liver disease, 7 (13.2%) focused on inflammatory bowel disease (IBD), 4 (7.5%) focused on gastroscopy, 4 (7.5%) focused on pancreatic pathology, 2 (3.8%) focused on gastrointestinal bleeding, one (1.9%) focused on endoscopic retrograde cholangiopancreatography (ERCP) and one (1.9%) focused on the optimisation of sedation in endoscopic practice more generally. Figure 3 presents a summary of the primary clinical areas of application.

As anticipated, classification tasks accounted for 32 (59.2%) studies, given that prediction and automation typically depend upon accurate classification. Nineteen (59.4%) of these studies focused on disease case identification. A broader array of clinical tasks presently exists within colonoscopy studies. The complete results of all the included studies are provided in Supplementary Material 8.

### Colonoscopy

Gourevitch et al. examined pathologist variation in colorectal adenoma classification and reported substantial average variations in reported adenoma detection rates (ADRs) between endoscopists (28.5%−42.4%), depending purely on the reporting pathologist [46]. Blumenthal et al. managed to predict colonoscopy nonattendance with an AUC of 0.70 [47]. Li et al. achieved 100% precision and recall while stratifying a sample of 300 Lynch syndrome mismatch repair status reports [48]. Shi et al. achieved 94% precision and recall in identifying cancers in family histories [49]. Paterson et al. achieved precision and recall values of 0.861 and 0.885, respectively, for predicting colonoscopy indication [50]. Hoogendorm et al. achieved an AUC of 0.896 for predicting colorectal cancer at the population level by including information derived from NLP [36].

A systematic review has already been performed regarding the automated detection of adenomas using NLP, for which a pooled precision of 99.7% was found [16]. However, the studies included in this review were rule-based and thus likely brittle. Table 5 summarises the key results of all colonoscopy result extraction studies focusing on polyp detection, where data were available.

**Table 5:** Colonoscopy Result Extraction Studies

*Study*	*Study Aim*	*Outcome*	*Model*	*Accuracy*	*Precision*	*Recall*	*F1 Score*
*Adenoma Studies*							
*Syed 2022* [51]	Extract clinical concepts from colonoscopy reports	Polyp Detection	*DL(BERT)*	*NR*	*0.91*	*0.94*	*0.92*
*Vithayathil 2022* [52]	Develop a large colonoscopy-based longitudinal cohort	Adenoma Detection	*RB*	*1*	*1*	*1*	*1*
*Nayor 2018* [53]	Automate calculation of ADR	Adenoma Detection	*RB*	*1*	*1*	*1*	*1*
*Laique 2021* [54]	Extract clinical information from colonoscopy reports	Polyp Detection	*RB*	*0.96*	*0.99*	*0.92*	*0.96*
*Tinmouth 2023* [55]	Identify colorectal adenomas in pathology reports	Non-Advanced Adenomas	*RB*	*0.99*	*1*	*0.99*	*0.99*
*Lee 2019* [56]	Identify colonoscopy quality and polyp findings	Polyps > 10 mm	*Commercial – I2E*	*0.95*	*1*	*0.91*	*0.95*
*Fevrier 2020* [37]	Extracting Polyp Variables	Adenoma Detection	*RB*	*NR*	*0.99*	*0.97*	*0.98*
*Bae 2022* [57]	Focusing on polyp detection	Adenoma Detection	*RB*	*0.99*	*1*	*0.99*	*0.99*
*Non-Adenoma Studies*							
*Redd 2022* [58]	Identify colorectal cancer in US military Veterans	Colorectal Cancer	*ML – LDA & DNN*	*0.99*	*0.91*	*0.97*	*0.94*
*Parthasarathy 2020* [59]	Automatically Diagnose Serrated Polyposis Syndrome (SPS)	Serrated Polyposis Syndrome	*RB*	*0.93*	*NR*	*NR*	*NR*
*Ternois 2018* [60]	Automatic coding system for colonoscopies	Attribute reports to CCAM codes	*RB*	*NR*	*0.92*	*0.92*	*0.92*

*Footnote: NR-Not reported. Precision (PPV)* = *TP/(TP* + *FP). Recall (sensitivity): TP/(TP* + *FN). Confidence intervals are reported in only a minority of studies*

Harrington et al. attempted to personalise colorectal cancer screening follow-up plans, achieving a maximum AUC of 0.65 for this task [61]. Three studies focused on clinical decision support for colorectal cancer surveillance interval calculations, each taking a different approach. Wadia et al.’s decision support system divided reports into actionable and nonactionable, achieving precision and recall of 92.8% and 98.9%, respectively [62]. Peterson et al.’s algorithm achieved an accuracy of 92% for assigning recommended surveillance intervals for colonoscopy [39], while Karwa et al. reported 100% accuracy on the same task [63]. In comparison, human surveillance judgments exhibited significantly more deviation from guidelines with a tendency toward earlier surveillance.

### Endoscopic retrograde cholangiopancreatography (ERCP) and endoscopic sedation

Shen et al.’s. human-in-the-loop clinical decision support system (CDSS), aimed to identify patients at higher risk of sedation errors preemptively [33], reduced the sedation-type error rate from 0.39% to 0.037%. Although the system had a high recall (sensitivity) of 89.2%, it suffered from low precision (28.5%). Imler et al.’s study focused on automated RB quality metric extraction for ERCP [64]. The model identified 13 pre, intra- and postprocedure quality measures from free text; however, the algorithm struggled more with complex concepts such as precut sphincterotomy (84% precision) and pancreatic stent placement (90% precision).

### Gastrointestinal bleeding

These studies used a combination of RB and ML/DL models to detect gastrointestinal bleeding in clinical free-texts—one in the emergency department (ED) [40] and the other in intensive care (ICU) [65]. Taggart et al.’s ICU study achieved the following precision: RB: 62.7%, ML: 55.9% and recall: RB: 91.1%, ML: 84.9% on MIMIC-III [66], while Shung et al.’s study achieved the following precision: RB: 72.0%, DL: 84.0% and recall: RB: 87.0%, DL: 90% for detecting bleeding among ED clinical text narratives. In both studies, the NLP approach exceeded the results of using ICD codes alone, but the transformer-based approach was strongest overall.

### Gastroscopy

Half of these studies focused on identifying gastric pathology from reports. The ML-ensemble model proposed by Ding et al. achieved an AUC of 0.891 for predicting gastric cancer from gastroscopy report text [38]. However, even this model was associated with a 25.6% missed diagnosis rate. Song et al. achieved even more impressive results while attempting to extract ten different gastric diseases from 1,000 validation gastroscopy reports, achieving a precision of > = 97.2% [67] in their centre.

McVay et al. used a 250-patient holdout set to detect dysphagia [68] and achieved a precision of 98.6% and an F1 score of 91.1% on this task. Finally, Nguyen Wenker et al. attempted to detect Barrett’s dysplasia in gastroscopy reports. In this task, they achieved 93.2% precision, although the algorithm could not effectively discriminate between low- and high-grade dysplasia [69].

### Inflammatory bowel disease (IBD)

Stidham et al. used an RB algorithm to identify the status of many skin, eye and joint-related IBD extraintestinal manifestations (EIMs), achieving average recalls of 92% for EIM presence [70]. Kurowski et al. created a computational Crohn’s disease state model with symptomatic/asymptomatic, active/inactive and tested/untested states. They reported that 20% of patients were lost to follow-up every 24 months [71]. Zand et al. classified flare-line conversations with IBD patients and reported that 90% of the dialogues could be assigned to one of seven categories [72]. Walker et al. achieved a precision of 79% and a recall of 92% for detecting liver test derangement in an IBD cohort [73].

Montoto et al. achieved precision and recall values of 88% and 98%, respectively, for the diagnosis of Crohn’s disease, 91% and 71%, for disease flares and 86% and 94%, for vedolizumab [74] across a Spanish cohort. Gomollón et al. built upon this work by attempting to predict disease flares in that cohort, achieving a precision and recall of 67% and 71%, respectively, using a random forest model and two years of input data [75]. Finally, Hou et al. achieved precision and recalls of 87% and 96.6%, respectively, for detecting low-grade dysplasia in IBD surveillance biopsies within a US cohort [76].

### Liver

Bell et al. reported that donor text narratives strongly predict liver utilisation(AUC = 0.81) but not 30-day (AUC = 0.53) or 1-year mortality (AUC = 0.52) [34]. Koola et al. phenotyped hepatorenal syndrome (HRS) with precision and recall ranging from 53–73% and 65–84%, respectively, with the final phenotyping algorithm achieving an AUC of 0.93 [77] on a small cohort.

Chang et al. achieved 98.4% precision and 90% sensitivity in identifying patients with cirrhosis [78]. Redman et al. and Van Fleck et al. achieved 89–91.8% precision and 90–93% recall for identifying obesity-related liver disease from liver imaging reports [79, 80]. Heidemann et al. attempted to identify drug-induced liver injury (DILI) cases [81]. However, with their four-term RB system, they achieved precision and recall values of 64% and 53%, respectively; in another study, Wang X et al. attempted to attribute the causality of idiopathic DILI, reaching a precision of 86% and recall of 82%, respectively, with their system [82].

The six remaining studies focused on identifying liver cancer, predominantly hepatocellular carcinoma (HCC), in radiology reports are summarised in Table 6.

**Table 6:** NLP Liver Cancer Identification Results

Study	Clinical Focus	Imaging Modalities	Accuracy	Precision	Recall	F1 Score
Yim 2017 [35]	Identifying and Classifying Tumour-event Attributes	Not Specified	NR	0.83–0.88	0.68–0.76	0.72
Tariq 2022 [83]	HCC	US/MR using templating	NR	0.97 for MR 0.68 for US	0.96 for MR 0.66 for US	0.95 for MR 0.67 for US
Liu W 2022 [41]	Liver Metastases in Colorectal Cancer	CT/MRI	0.96	NR	NR	NR
Liu H 2021 [84]	Predicting the Phrase: ‘hyperintense enhancement in the arterial phase.’	CT Only	0.98	0.98	0.99	0.98
Sada 2016 [85]	HCC	CT/MRI	NR	0.68	0.75	0.71
Wang T 2022 [86]	HCC	Predominantly US with some CT/MRI	0.99	0.86	1	0.92

Table Footnote: *NR* Not Reported. Precision (PPV) = TP/(TP + FP). Recall (sensitivity): TP/(TP + FN)

### Pancreas

Three systems reported precisions ranging between 33 and 99% and recalls ranging from 25 to 99.9% for detecting pancreatic cysts in radiological examinations [87–89]. These studies included 269,221 individual patients, but substantial heterogeneity in the methods, environments, and underlying imaging studies renders reliable meta-analysis challenging. Xie et al. achieved precision and recall values of 85.5–100% and 88.7–98.7%, respectively, for various chronic pancreatitis features [90], finding a more significant ten-year mortality (32.5% vs 21.2%) in those with more advanced radiological features.

### Quality assessment

Only 6 (11.3%) studies explored algorithm running costs, while model explainability was mentioned in only 5 (9.4%) studies. However, 34 (64.1%) of the studies explicitly mentioned generalisability. Open-source code was only available for 5 (9.3%) studies. Supplementary Material 4 summarises the quality appraisal results for each study.

### Risk of bias assessment

All studies were assessed across ten areas of potential bias. All studies were scored low for deviation bias (a measure of unclear aims). Only 5 (9.4%) studies had a low risk of bias across all domains. Supplementary Material 5 summarises the ROB results. Validation bias was the most common, with only 13 (24.5%) studies scoring as low risk in this domain.

## Discussion

In gastroenterology, NLP algorithms have successfully extracted diagnoses and clinical features from radiology, histopathology, and endoscopy reports. This enables healthcare providers to identify patients at risk of liver disease, polyps/cancer, and sedation-related endoscopy errors. Furthermore, NLP systems have demonstrated effectiveness in analysing clinicians’ notes to predict disease flare in the context of IBD, thereby facilitating timely intervention.

The author lists suggest that few research groups are presently active in this field. Most NLP work within gastroenterology is concentrated on only a few clinical domains, most obviously colonoscopy. A relatively narrow range of clinical tasks, such as automated endoscopic or radiological report interpretation, is being prioritised. Encouragingly, most studies focus on open-source software, although code sharing is rare.

The employed methodologies were highly heterogeneous, suggesting poor consensus regarding optimal methods at this point, impeding meta-analysis and consensus building. Positive results have been obtained in some areas, such as automated adenoma, pancreatic cyst, and hepatocellular carcinoma detection. However, limited external validation and a preference for rule-based methods cast doubt on model robustness and generalisability.

Rule-based (RB) methods are widely used due to their transparency and ease of understanding, fostering greater clinician trust. Their limitations are well-defined, and when carefully designed, RB methods often achieve higher recall rates than machine learning (ML) approaches. This makes them particularly useful for excluding patients unlikely to have a specific condition. Additionally, RB methods are cost-effective to develop and execute, making them an economical choice in many settings. Conversely, when trained on high-quality data, ML methods can achieve significantly higher precision and handle greater complexity than RB systems. However, they often require substantial computational resources that may not be available in all clinical environments, and they can act as ‘black-boxes’. Moreover, ML models are more susceptible to errors arising from flaws in training data. While large language models (LLMs) have garnered considerable attention, their high operational costs, comparative slowness and unpredictability currently limit their clinical utility.

However, the quality of the included studies varied considerably, with explainability, costs, and parameterisation generally being poorly explored. A total of 43.3% of the studies provided no demographic information, meaning that inherent algorithm biases cannot be examined at all in these models. None of the studies in the review discussed demographic parity, equal opportunity, or disparate impact analysis, which means that fairness cannot be adequately assessed in any of the models studied. Where demographic information was provided, patient samples were predominantly Caucasian and male, limiting the generalisability and, thus, the applicability of any trained models. This poses significant ethical questions about using these algorithms in clinical practice and suggests a need for more robust future reporting.

As colonoscopy studies have highlighted, model sharing is almost nonexistent, leading to substantial duplication of effort. Incentivising transparency must become a priority for publishers and grant-awarding bodies, or future progress will be stunted.

Future work should also focus on managing and investigating functional bowel disorders, nutrition, and intestinal failure, which are presently absent in the peer-reviewed literature. Opportunities for future research abound. Potential future research directions are suggested below:

1. Developing and applying NLP approaches which can accomplish complex tasks such as generating disease timelines, monitoring clinical progress and developing complex clinical phenotypes.
2. Encouraging open-sharing of published NLP models while maintaining data protection and patient privacy. Enabling algorithm fine-tuning by others.
3. Applying NLP to study a broader range of gastrointestinal diseases and more diverse, representative patient samples to reduce bias in trained models.
4. Exploring open-source code sharing internationally across health systems to facilitate testing of interoperability and model assessment in varied clinical practice settings.
5. More robust evaluation of NLP algorithms, considering cost-effectiveness, bias/fairness, time savings, carbon footprint, and acceptability within a clinical workflow.

## Conclusion

NLP can unlock substantial clinical information from free-text notes stored in EPRs and is already being used, particularly to interpret colonoscopy and radiology reports. However, the models we have thus far lack transparency, leading to duplication, bias, and doubts about generalisability. Therefore, greater clinical engagement, collaboration, and open sharing of appropriate datasets and code are needed before validated, trusted, semiautonomous NLP systems can be deployed widely and significant clinical benefits can be realised.

## Supplementary Information

Supplementary Material 1.

Supplementary Material 2.

Supplementary Material 3.

Supplementary Material 4.

Supplementary Material 5.

Supplementary Material 6.

Supplementary Material 7.

Supplementary Material 8.

Supplementary Material 9.

Supplementary Material 10.

## Data Availability

All data generated or analysed during this study are included in this published article and its supplementary information files.
