# Supplementary material for: A foundation systematic review of natural language processing applied to gastroenterology & hepatology
Source: BMC Gastroenterol. 2025 Feb 6;25:58. doi: 10.1186/s12876-025-03608-5 (PMC11800601; doi:10.1186/s12876-025-03608-5)
Supplement: Supplementary file 2 — Supplementary Material 2. [file 12876_2025_3608_MOESM2_ESM.pdf]

Supplemental File 2: Search Strategy

Table B. Detailed Applied Search Strategy Per Database	
**Search Query String**	**Database**
(("gastroenterol*" or "egd" or "ogd" or "endoscopy" or "upper endoscopy" or "esophagogastroduodenoscopy" or "colonoscopy" or "lower endoscospy" or "colorectal cancer screening" or "colon cancer screening" or "adenoma detection rate" or "screening colonoscopy" or "surveillance colonoscopy" or "gerd" or "gord" or "gastroesophageal reflux" or "acid reflux" or "reflux disease" or "barrett's esophagus" or "barrett's oesophagus" or "ppi" or "proton pump inhibitor" or "gastrointestinal hemorrhage" or "gastrointestinal haemorrhage" or "variceal bleeding" or "gi bleeding" or "inflammatory bowel disease" or "ulcerative colitis" or "crohn*" or "inflammatory bowel disea*" or "chronic liver disease" or "chronic hepatitis b" or "hepatitis a immuni#ation" or "hepatitis b immuni#ation" or "diagnostic paracentesis" or "early paracentesis" or "ascites" or "esophageal varices" or "gastric varices" or "screening egd" or "hepatocellular carcinoma screening" or "hepatic encephalopathy" or "liver disease" or "hepatic disease" or "pancreatobiliary disease" or "acute pancreatitis" or "chronic pancreatitis" or "alcoholic pancreatitis" or "gallstone pancreatitis" or "ercp" or "pancreatic insufficiency" or "ibs" or "irritable bowel syndrome" or "neurogastroenterology" or "functional gastroenterology" or "iron deficiency anaemia" or "iron deficiency anemia" or "ida" or ((("nutritionist" or "nutrition referral" or "endoscopic biopsy" or "serologic diagnosis" or "serologic testing" or "endoscopic diagnosis") and "celiac disease") or "celiac" or "coeliac" or "sprue")) and (("Natural Language Processing" or "Medical Language Processing" or "nlp") and (Electronic Health Record* or EHR or EMR or electronic medical record* or report or chart or reports or charts or "clinical notes" or "clinical text" or "medical notes" or "medical text") and (ontolog* or concept* or encod* or annotat* or "code" or "coding" or "named entity" or "linkage"))).mp.	EMBASE & MEDLINE (OVID) Full Free-Text Search Terms
(exp gastroenterology/ or exp digestive tract endoscopy/ or exp fiberscope endoscopy/ or exp high resolution endoscopy/ or exp magnifying endoscopy/ or exp narrow band imaging/ or exp magnifying endoscopy/ or exp nasopharyngoscopy/ or exp nasopharyngoscopy/ or exp nasopharyngoscopy/ or exp pharyngoscopy/ or exp videoendoscopy/ or exp white light endoscopy/ or exp endoscopic retrograde cholangiopancreatography/ or exp liver disease/ or exp liver cirrhosis/ or exp hepatobiliary disease/ or exp hepatobiliary disease/ or exp hepatobiliary system hemorrhage/ or exp hepatobiliary system infection/ or exp hepatobiliary system inflammation/ or exp hepatobiliary system metastasis/ or exp hepatobiliary system tumor/ or exp pancreatitis/ or exp pancreas disease/ or exp biliary tract disease/ or exp hepatitis C/ or exp hepatitis B/ or exp celiac disease/ or exp inflammatory bowel disease/ or exp ulcerative colitis/ or exp colitis/ or exp Crohn disease/ or exp enteritis/ or exp parenteral nutrition/ or exp total parenteral nutrition/ or exp parenteral nutrition bag/ or exp peripheral parenteral nutrition/ or exp intestinal failure/ or exp digestive system function disorder/ or exp irritable colon/ or exp iron	EMBASE & MEDLINE (OVID) MeSH Terms

deficiency anemia/) and (exp natural language processing/ and (exp electronic health record/ or exp information retrieval/ or exp information processing/ or exp data mining/ or exp algorithm/))	
((gastro* or hepat* or endosco* or "liver disease" or reflux or barrett* or bleed* or ibd or colitis or "inflammatory bowel disease" or crohn* or ibs or ibd or "irritable bowel" or pancreati* or coelia* or "iron deficiency" or "total parenteral nut*" or "colorectal cancer screening" or cirrhosis or varices) and ("natural language processing" or "nlp" or "medical language processing")).mp.	EMBASE String Search
((gastro* OR hepat* OR endosco* OR "liver disease" OR reflux OR barrett* OR bleed* OR ibd OR colitis OR "inflammatory bowel disease" OR crohn* OR ibs OR ibd OR "irritable bowel" OR pancreati* OR coelia* OR "iron deficiency" OR "total parenteral nut*" OR "colorectal cancer screening" OR cirrhosis OR varices) AND ("natural language processing" OR "nlp" OR "medical language processing"))	Pubmed String Search
(TITLE-ABS-KEY-AUTH ((gastro* OR hepat* OR endosco* OR "liver disease" OR reflux OR barrett* OR bleed* OR ibd OR colitis OR "inflammatory bowel disease" OR crohn* OR ibs OR ibd OR "irritable bowel" OR pancreati* OR coelia* OR "iron deficiency" OR "total parenteral nut*" OR "colorectal cancer screening" OR cirrhosis OR varices) AND ("natural language processing" OR "nlp" OR "medical language processing")))	Scopus String Search
(("gastro*" OR "hepat*" OR "endosco*" OR "liver disease" OR reflux OR "barrett*" OR "bleed*" OR ibd OR colitis OR "inflammatory bowel disease" OR "crohn*" OR ibs OR ibd OR "irritable bowel" OR "pancreati*" OR "coelia*" OR "iron deficiency" OR "total parenteral nut*" OR "colorectal cancer screening" OR cirrhosis OR varices) AND ("natural language processing" OR "nlp" OR "medical language processing"))	ACM Digital Library String Search
(((gastro* OR hepat* OR endos* OR "liver disease" OR reflux OR barrett* OR bleed* OR ibd OR colitis OR "inflammatory bowel disease" OR crohn* OR ibs OR ibd OR "irritable bowel" OR pancreati* OR coelia* OR "iron deficiency" OR "total parenteral nut*" OR "colorectal cancer screening" OR cirrhosis OR varices) AND ("natural language processing" OR "nlp" OR "medical language processing")))	IEEE String Search
gastroenterology or hepatology or endoscopy or liver disease and natural language processing or nlp or medical language processing	Google Scholar String Search
gastroenterology OR hepatology OR endoscopy OR "liver disease" AND "natural language processing" OR nlp OR "medical language processing"	Arixv String Search
