# Supplementary material for: A foundation systematic review of natural language processing applied to gastroenterology & hepatology
Source: BMC Gastroenterol. 2025 Feb 6;25:58. doi: 10.1186/s12876-025-03608-5 (PMC11800601; doi:10.1186/s12876-025-03608-5)
Supplement: Supplementary file 3 — Supplementary Material 3. [file 12876_2025_3608_MOESM3_ESM.pdf]

Supplemental File 3: Quality Assessment, Reporting and Risk of Bias

**Table C1.** Quality Metrics	
Metric	***Principle***
1.	Clearly defined purpose
2.	Clearly defined primary outcome
3.	Detailed description of NLP approach
4.	Number of documents specified
5.	Number of patients specified
6.	Patient demographic information reported
7.	Justification for evaluation design and metrics
8.	Interoperable definition of outcomes used (common data model / standard)
9.	Assessment of level of generalisability
10.	Evaluation metrics reported
11.	Use of appropriate statistical tests
12.	Statistical treatment of results (e.g., confidence tests)
13.	Detailed description of comparative evaluation design
14.	Was parameterisation and tuning conducted
15.	Discussion of model costs (time, resources) and explain-ability
16.	Assessment of potential for explainability
17.	Availability & standardisation of code (sharing) and datasets for reproducibility

**Table C2.** Principles of Synthesis Without Meta-Analysis (SWiM)	
Number	***Principle***
1.	A rationale given for groupings, interventions, or outcomes.
2.	Standardised metrics for each outcome will be described and an explanation given of why the metric was chosen.
3.	Any transformation will be explicitly explained especially if synthesis has been required.
4.	Supporting Justification for selecting studies will be given based on design, risk of bias (ROB) and relevance to the study question.
5.	The methods used to examine heterogeneity will be stated where meta-analysis is not possible.
6.	A measure of certainty will be provided for the findings.
7.	Any limitations will be openly reported especially where these relate to questions of grouping in the context of the original research question.

**Table C3.** Risk of Bias Assessment Questions	
Question	***Explanation***
Q1. [METRIC BIAS]	Was the outcome data complete?
Q2. [ATTRITION BIAS]	Were participant exclusions, attrition and incomplete outcome data adequately addressed in the published report?
Q3. [REPORTING BIAS]	Were outcomes reported for all cohorts in full or only selectively?
Q4. [SELECTION BIAS]	Is there evidence of selective outcome reporting, and might this have affected study results?
Q5. [VALIDATION BIAS]	Was the validation methodology used in the study robust and valid?
Q6. [CONFOUNDING BIAS]	Could any confounding have caused an issue in this study, and if so, how?
Q7. [CLASSIFICATION BIAS]	Do you think the interventions in the study are classified clinically appropriately?
Q8. [DEVIATION BIAS]	Did the researchers inadvertently deviate from their original intentions during the study?
Q9. [OMISSION BIAS]	Was any critical data missing from the study or not fully reported/discussed?
Q10. [MEASUREMENT BIAS]	Were any inappropriate measurements used in the study?
