# Supplementary material for: A foundation systematic review of natural language processing applied to gastroenterology & hepatology
Source: BMC Gastroenterol. 2025 Feb 6;25:58. doi: 10.1186/s12876-025-03608-5 (PMC11800601; doi:10.1186/s12876-025-03608-5)
Supplement: Supplementary file 4 — Supplementary Material 4. [file 12876_2025_3608_MOESM4_ESM.pdf]

Supplemental File 4: Study Quality Appraisal

Table D - Study Quality Appraisal (Based on the approaches of Koleck[29] & Nascimento[30] according to the principles of SwiM[26])

Author	Clearly Described Purpose	Number of Patients Specified	Patient Demographic Info Reported	Number of Documents Specified	Description of the NLP Approach	Parameterisation Described	Validation Process Clearly Described	Justification For Chosen Methodology Reported	Justification Given for Evaluation Design and Metrics	Evaluation Metrics Reported	Statistical Treatment of Results	Measures of Certainty Given	Costs of Running Algorithm Discussed	Model Explainability Discussed in Paper	Model Generalisability Discussed	Availability of code, models or datasets for reproducibility
Colonoscopy																
Harrington 2018 (61)	✓	✓			✓	✓		✓	✓	✓	✓	✓				
Gourevitch 2018 (46)	✓		✓	✓	✓	✓	✓									
Wadia 2017 (62)	✓		✓	✓	✓	✓	✓	✓	✓	✓		✓	✓		✓	
Hoogendoorn 2016 (36)	✓	✓	✓	✓	✓	✓		✓		✓	✓	✓			✓	
Syed 2022 (51)	✓			✓	✓	✓	✓	✓	✓	✓	✓	✓	✓		✓	
Karwa 2020 (63)	✓			✓	✓		✓								✓	
Li 2021 (48)	✓	✓	✓	✓	✓		✓		✓	✓	✓	✓				
Vithayathil 2022 (52)	✓	✓	✓	✓	✓	✓	✓			✓						
Nayor 2018 (53)	✓	✓			✓	✓	✓		✓	✓	✓	✓			✓	
Parthasarathy 2020 (59)	✓	✓		✓	✓		✓		✓	✓		✓				
Laique 2021 (54)	✓			✓	✓		✓	✓	✓	✓		✓	✓	✓	✓	
Peterson 2021 (39)	✓	✓		✓	✓		✓	✓	✓	✓		✓			✓	
Tinmouth 2023 (55)	✓		✓	✓	✓		✓	✓	✓	✓		✓		✓	✓	
Redd 2022 (58)	✓	✓	✓	✓	✓		✓	✓	✓	✓	✓	✓	✓			
Blumenthal 2015 (47)	✓	✓	✓		✓	✓	✓	✓	✓	✓	✓	✓			✓	
Lee 2019 (56)	✓		✓	✓	✓		✓	✓	✓	✓		✓			✓	
Fevrier 2020 (37)	✓	✓	✓	✓	✓			✓	✓	✓		✓			✓	✓
Shi 2022 (49)	✓	✓	✓	✓	✓	✓	✓	✓	✓	✓	✓	✓			✓	
Bae 2022 (57)	✓	✓	✓	✓	✓		✓	✓	✓	✓	✓	✓			✓	
Patterson 2015 (50)	✓			✓	✓		✓	✓	✓	✓		✓				
Ternois 2018 (60)	✓			✓	✓		✓			✓		✓			✓	
ERCP & Sedation																
Imler 2018 (64)	✓	✓	✓	✓	✓	✓		✓	✓	✓	✓	✓				
Shen 2021 (33)	✓		✓	✓	✓		✓	✓	✓	✓	✓	✓			✓	
Gastrointestinal Bleeding																
Shung 2021 (40)	✓	✓	✓	✓	✓	✓	✓	✓	✓	✓	✓	✓				
Taggart 2018 (65)	✓	✓	✓	✓	✓	✓	✓		✓	✓	✓	✓			✓	✓

Gastroscopy																
McVay 2018 (68)	✓	✓		✓	✓	✓	✓	✓	✓	✓	✓					
NguyenWenker 2023(69)	✓	✓			✓		✓		✓	✓					✓	
Ding 2020 (38)	✓			✓	✓	✓	✓	✓	✓	✓	✓	✓			✓	
Song 2022 (67)	✓	✓	✓	✓	✓	✓	✓	✓	✓	✓	✓	✓	✓			
IBD																
Gomollón 2022 (75)	✓	✓	✓		✓	✓		✓	✓	✓	✓	✓				
Hou 2016 (76)	✓	✓		✓	✓		✓	✓	✓	✓		✓				
Stidham 2022 (70)	✓	✓	✓	✓	✓		✓	✓	✓	✓	✓	✓			✓	
Walker 2016 (73)	✓	✓	✓		✓		✓	✓	✓	✓	✓	✓			✓	
Zand 2020 (72)	✓	✓	✓	✓	✓		✓	✓			✓					
Montoto 2022 (74)	✓	✓	✓	✓	✓	✓	✓	✓	✓	✓	✓	✓			✓	
Kurowski 2022 (71)	✓	✓							✓		✓		✓	✓		
Liver																
Bell 2022 (34)	✓	✓	✓		✓	✓	✓	✓	✓	✓	✓	✓			✓	
Heidemann 2017 (81)	✓	✓	✓	✓	✓		✓	✓	✓	✓	✓	✓			✓	
Redman 2017 (79)	✓	✓		✓	✓		✓	✓	✓	✓	✓	✓			✓	
Wang X 2022 (82)	✓	✓			✓	✓		✓	✓	✓	✓	✓		✓	✓	✓
Liu W 2022 (41)	✓	✓			✓		✓	✓	✓	✓	✓	✓			✓	
VanVleck 2019 (80)	✓	✓	✓	✓	✓		✓	✓	✓	✓	✓	✓		✓	✓	
Yim 2017 (35)	✓	✓		✓	✓	✓	✓	✓	✓	✓		✓			✓	
Koola 2018 (77)	✓	✓	✓		✓			✓	✓	✓		✓			✓	
Tariq 2022 (83)	✓			✓	✓		✓	✓	✓	✓		✓				
Chang 2016 (78)	✓	✓			✓		✓	✓	✓	✓	✓	✓			✓	
Liu H 2021 (84)	✓	✓		✓	✓			✓	✓	✓		✓			✓	
Sada 2016 (85)	✓	✓	✓		✓		✓	✓	✓	✓	✓	✓			✓	
Wang T 2022 (86)	✓			✓	✓		✓	✓	✓	✓	✓	✓			✓	✓
Pancreas																
Kooragayala 2022 (89)	✓	✓	✓	✓	✓	✓		✓	✓	✓		✓				
Roch 2015 (87)	✓	✓		✓	✓	✓	✓	✓	✓	✓	✓				✓	
Xie 2020 (90)	✓	✓	✓	✓	✓	✓	✓	✓	✓	✓	✓	✓				
Yamashita 2022 (88)	✓	✓	✓	✓	✓	✓	✓	✓	✓	✓	✓	✓			✓	✓

Footnote: ✓-Present, blank-Absent
