# Supplementary material for: A foundation systematic review of natural language processing applied to gastroenterology & hepatology
Source: BMC Gastroenterol. 2025 Feb 6;25:58. doi: 10.1186/s12876-025-03608-5 (PMC11800601; doi:10.1186/s12876-025-03608-5)
Supplement: Supplementary file 5 — Supplementary Material 5. [file 12876_2025_3608_MOESM5_ESM.pdf]

Supplemental File 5: Risk of Bias Assessment

Table E. Risk of Bias Assessment (Based on a modified ROBINS-I[27] checklist)

Author	Outcome Data Complete	Attrition Bias	Reporting Bias	Selection Bias	Validation Bias	Confounding Bias	Classification Bias	Deviation Bias	Omission Bias Risk	Inappropriate Measurements
Colonoscopy										
Harrington 2018 (61)				↓				↓		↓
Gourevitch 2018 (46)		↓	↓	↓		↓	↓	↓		
Wadia 2017 (62)						↓	↓	↓		↓
Hoogendoorn 2016 (36)	↓			↓	↓			↓	↓	↓
Syed 2022 (51)	↓	↓	↓	↓	↓	↓	↓	↓	↓	↓
Karwa 2020 (63)								↓		
Li 2021 (48)	↓		↓				↓	↓		↓
Vithayathil 2022 (52)	↓						↓	↓		
Nayor 2018 (53)	↓		↓			↓		↓		↓
Parthasarathy 2020 (59)							↓	↓		↓
Laique 2021 (54)	↓				↓	↓		↓		↓
Peterson 2021 (39)						↓	↓	↓		↓
Tinmouth 2023 (55)	↓		↓			↓		↓		↓
Redd 2022 (58)	↓	↓						↓	↓	↓
Blumenthal 2015 (47)				↓			↓	↓		↓
Lee 2019 (56)		↓				↓	↓	↓		↓
Fevrier 2020 (37)	↓		↓	↓		↓	↓	↓	↓	↓
Shi 2022 (49)	↓	↓	↓	↓		↓	↓	↓	↓	↓
Bae 2022 (57)	↓	↓	↓	↓	↓	↓	↓	↓	↓	↓
Patterson 2015 (50)	↓	↓	↓	↓	↓	↓	↓	↓	↓	↓
Ternois 2018 (60)			↓					↓		↓
ERCP & Sedation										
Imler 2018 (64)		↓	↓	↓		↓	↓	↓		
Shen 2021 (33)		↓					↓	↓		↓
Gastrointestinal Bleeding										
Shung 2021 (40)	↓	↓	↓	↓		↓	↓	↓	↓	↓
Taggart 2018 (65)	↓	↓	↓	↓	↓	↓	↓	↓	↓	↓
Gastroscopy										

McVay 2018 (68)					↓	↓	↓	↓		↓
NguyenWenker 2023(69)	↓	↓				↓	↓	↓		↓
Ding 2020 (38)	↓	↓	↓	↓		↓		↓	↓	↓
Song 2022 (67)	↓		↓			↓	↓	↓		↓
IBD										
Gomollón 2022 (75)		↓				↓	↓	↓	↓	↓
Hou 2016 (76)					↓		↓	↓		↓
Stidham 2022 (70)		↓	↓		↓	↓	↓	↓	↓	↓
Walker 2016 (73)								↓		↓
Zand 2020 (72)		↓						↓		
Montoto 2022 (74)	↓			↓		↓	↓	↓		↓
Kurowski 2022 (71)		↓		↓				↓		↓
Liver										
Bell 2022 (34)		↓		↓	↓		↓	↓		↓
Heidemann 2017 (81)		↓		↓		↓	↓	↓		↓
Redman 2017 (79)	↓		↓		↓	↓		↓		↓
Wang X 2022 (82)		↓	↓	↓			↓	↓	↓	↓
Liu W 2022 (41)		↓		↓		↓	↓	↓	↓	↓
VanVleck 2019 (80)								↓		↓
Yim 2017 (35)	↓		↓	↓		↓		↓	↓	↓
Koola 2018 (77)	↓		↓	↓	↓	↓	↓	↓	↓	↓
Tariq 2022 (83)	↓	↓	↓	↓		↓	↓	↓	↓	↓
Chang 2016 (78)	↓			↓		↓	↓	↓		↓
Liu H 2021 (84)	↓	↓		↓		↓	↓	↓		↓
Sada 2016 (85)	↓					↓	↓	↓		↓
Wang T 2022 (86)		↓						↓	↓	↓
Pancreas										
Kooragayala 2022 (89)		↓		↓				↓	↓	↓
Roch 2015 (87)		↓	↓	↓		↓	↓	↓	↓	↓
Xie 2020 (90)	↓	↓	↓	↓		↓	↓	↓		↓
Yamashita 2022 (88)	↓	↓	↓	↓	↓	↓	↓	↓	↓	↓

Footnote: ↓-Low ROB, Blank- Unclear or more commonly High ROB
