# Supplementary material for: A foundation systematic review of natural language processing applied to gastroenterology & hepatology
Source: BMC Gastroenterol. 2025 Feb 6;25:58. doi: 10.1186/s12876-025-03608-5 (PMC11800601; doi:10.1186/s12876-025-03608-5)
Supplement: Supplementary file 6 — Supplementary Material 6. [file 12876_2025_3608_MOESM6_ESM.pdf]

Supplemental File 6: Screening Inter-Observer Agreement

**Table F.** Abstract Screening Inter-Observer Agreement			
**Reviewer A**	**Reviewer B**	**Proportionate Agreement**	**Cohen's Kappa**
MS	AO	0.97782	**0.81672**
AV	MS	0.92691	**0.70593**
