# Supplementary material for: A foundation systematic review of natural language processing applied to gastroenterology & hepatology
Source: BMC Gastroenterol. 2025 Feb 6;25:58. doi: 10.1186/s12876-025-03608-5 (PMC11800601; doi:10.1186/s12876-025-03608-5)
Supplement: Supplementary file 7 — Supplementary Material 7. [file 12876_2025_3608_MOESM7_ESM.pdf]

Supplemental File 7: Populations Studied and Methods for Included Studies

Table F. Populations Studied and Methods for Included Studies

Authors	Patient Population	Patient Sample Size	Patient Demographic Characteristics	Setting or Dataset	Number of Documents	Document Types	NLP Tools and Methods	NLP Evaluation Methods
Colonoscopy								
Harrington 2018 (61)	Individuals from a tertiary academic care centre in New Hampshire from 2011 to 2017. The patients underwent colonoscopy.	952 individuals from a tertiary academic care centre in New Hampshire	The study reports that demographic values such as age were used to derive the model, but no actual values were published in the report.	Tertiary Teaching Hospital, Single Site	The specific number of documents is not given.	Colonoscopy records with associated pathology records	Python's NLTK (Natural Language Toolkit) for NLP tasks, including tokenisation, n-gram generation, and text processing from electronic medical records to extract polyp characteristics for predicting colorectal polyp recurrence risk using machine learning models,	The researchers derived the validation dataset by randomly splitting the dataset into training and validation sets, with the validation dataset consisting of 20% of the total dataset, approximately 190 individuals. Additionally, they used 10-fold nested cross-validation on six different ML models to increase algorithm robustness. Validation level Type 2a.
Gourevitch 2018 (46)	A population undergoing colonoscopies, primarily for screening or surveillance, with a significant portion between the ages of 50 and 69. Adenomas were detected in 35% of cases, and serrated polyps were detected in 7%.	The number of patients is not given.	The study reports some demographic values for patient age and colonoscopy indication. For patient age, the values are as follows: Under 50 (6,127 patients, 7.2%), 50–59 (31,525 patients, 36.9%), 60–69 (29,177 patients, 34.1%), and 70+ (18,622 patients,	Specifically examining the variation in pathologist classification of colorectal adenomas and serrated polyps.	85,526 Colonoscopy reports with 50,453 reports with associated polyp specimens	Colonoscopy reports and associated pathology reports from a multi-site sample.	The details of the tool had been published in a prior study	The researchers derived the validation dataset by excluding inpatient colonoscopies, those on patients under 40 years old, and patients with inflammatory bowel disease. They also excluded colonoscopies performed by low-volume endoscopists or where the pathology specimen was assessed by a low-volume pathologist and validated the algorithm used to ensure >95% accuracy in a prior study.

			27.8%). Regarding colonoscopy indication, the values are Screening (40,429 patients, 47.3%), Surveillance (27,732 patients, 32.4%), Diagnostic (15,536 patients, 18.2%), and Missing (1,829 patients, 2.1%).					
Wadia 2017 (62)	Monitoring post-colonoscopy patients for follow-up and scheduling. The study specifically targeted African-American male veteran patients undergoing colonoscopy.	The number of patients is not given.	Demographic values for African-American male veteran patients undergoing colonoscopy are given, emphasising the higher prevalence of proximal colon polyps and villous histology in this group.	Patient care post-colonoscopy, specifically monitoring and scheduling follow-up for African-American male veteran patients undergoing colonoscopy from 2011-2015 at the West Haven VA and six other VA sites.	2685 pathology reports, with 2085 in the training corpus and 600 in the testing corpus.	Surgical pathology reports from 2011-2015 at the West Haven VA for manual annotation. These reports were classified into three categories: non-actionable (NO-ACT), actionable (ACT), and other anatomic sites, with similar distributions in the training and testing corpus.	EHOST for manual annotation of surgical pathology reports, natural language processing (NLP) tools for classifying reports into three categories, and a thesaurus-based solution for recognising salient pathological terms. Regular expressions were employed for extracting diagnostic portions, and a discriminant function was developed using SQL statements to differentiate diagnoses and identify cases requiring follow-up. The discriminant function was refined with additional terms for classification. The NLP tools were applied to analyse and categorise report content.	Criteria were validated by categorising 20 duplicate pathology reports reviewed by two reviewers separately. No discrepancies were found in the categorisation between reviewers. One hundred consecutive reports were chosen from each site for manual classification with a 600-patient validation hold-out set.

Hoogendoorn 2016 (36)	Aged 30 and above, specifically individuals at risk for colorectal cancer, including those with and without the condition.	A population of 90,000 patients with 588 cases of colorectal cancer.	The study states that adding text data with age and gender increased the AUC from 0.831 to 0.865 for predicting colorectal cancer. However, only very limited demographic details are given, such as the 30-year-old cut-off.	Uncoded consultation notes from electronic medical records for predictive modelling of colorectal cancer in a primary care setting in Utrecht, the Netherlands	14.6 million consultation notes were available. However, the study dataset included 502,000 consultations, 344,000 medication prescriptions, 316,000 lab result records, 2800 referrals and 1.25 million consultation notes.	Consultation notes, medication prescriptions, lab result records, and referrals from electronic medical records, along with uncoded and coded data.	NLP tools such as MetaMap, HITEx, and cTAKES process medical text and match it to a medical ontology. These tools perform pre-processing operations like tokenisation and stemming before matching text with a medical ontology for accurate term attribution.	Random splitting and 5-fold cross-validation. Type 2a validation at most.
Syed 2022 (51)	Patient population undergoing colonoscopies	The number of patients is not given	Demographic information is not given	Colonoscopies for colonoscopy concept compilation using combined contextual embeddings	The original dataset included 16,900 colonoscopy, 11,182 pathology, and 7,364 radiology reports, respectively. A random sample of 1,281 reports was selected for annotation. The unlabeled corpus contains 34,165 notes from the three document types used to pre-train LMs.	Colonoscopy reports, pathology reports, and radiology reports.	BERT (Bidirectional Encoder Representations from Transformers) and FLAIR embeddings as NLP tools for clinical concept extraction from colonoscopy, pathology, and radiology reports. These embeddings were combined in a hybrid Artificial Neural Network (h-ANN) to enhance word representations and improve the performance of the clinical NLP system.	The researchers derived the validation dataset for the study by manually reviewing 300 randomly selected colonoscopy procedures, 219 associated pathology notes, and 123 radiology notes. These notes were reviewed for 15 entities related to colonoscopy quality improvement and colorectal cancer research. The validation dataset was screened by four reviewers, including one medical student and three trained data warehouse analysts, under the guidance of a domain expert.

Karwa 2020 (63)	Patient populations undergoing colonoscopies at the Cleveland Clinic system from 2012 to 2016. Excluded cases had incomplete data, non-screening or surveillance indications, inadequate bowel preparation, incomplete procedures, piecemeal polyp resection, personal history of inflammatory bowel disease, colorectal cancer (CRC), high-risk CRC syndromes, or were inpatient procedures.	2739 colonoscopies (300 in the validation set), but the number of patients is not specified.	Age, Gender and Race were in the dataset, but they were not reported in the study	Colonoscopies were performed at the Cleveland Clinic system in the US from 2012 to 2016.	156,258 colonoscopy reports	Electronic medical records (EMR) containing colonoscopy and pathology reports.	Prolog, a general-purpose programming language, parsed colonoscopy and pathology reports.	From the cases included in the smaller validation dataset (n=300 -> 263 after applying exclusions), patient, procedural, and pathologic data points were extracted from the EMR by NLP and by manual chart review by one of the investigators (G.P.).
Li 2021 (48)	Individuals with colorectal cancer (CRC) who underwent Lynch syndrome (LS) screening using reflex mismatch repair (MMR) immunohistoch	5570 patients. The patients included individuals of various ages (<50, 50-69, 70+), sexes (female, male), race/ethnicity (African	The specific values provided are as follows:<br>- Age: <50 (957), 50-69 (2670), 70+ (1943)<br>- Sex: Female (2741), Male (2829)<br>- Race/ethnicity:	Lynch syndrome (LS) screening in colorectal cancer (CRC) patients within a large, community-based, integrated	1,000 randomly selected pathology reports were utilised for developing the NLP tool, and a validation set of 300 patients was employed	Pathology reports were primarily used in this study	The study utilised Natural Language Processing (NLP) tools developed and validated for extracting mismatch repair immunohistochemistry (IHC) results in Lynch syndrome screening. These NLP tools were refined through manual review findings until	The researchers derived the validation dataset by randomly selecting 300 patients from the 5,570 colorectal cancer patients with mismatch repair immunohistochemistry (IHC) results. The validation dataset included individuals across various age groups, sexes, races/ethnicities, and cancer stages. The final NLP query tool was validated by

	emistry (IHC) on CRC specimens.	American, Asian, Hispanic, Non-Hispanic white, Other), and cancer stages (early stage, advanced stage, unknown stage).	African American (403), Asian (844), Hispanic (694), Non-Hispanic white (3570), Other (59)<br>- Cancer stage: Early stage (3072), Advanced stage (2471), Unknown stage (27)	healthcare system at Kaiser Permanente Northern California.	to validate the NLP tool		achieving a high level of concordance. Performance characteristics, including kappa statistics for overall agreement with manual chart review, were calculated for the NLP tool.	independent manual chart review (D.L. and E.L.).
Vithayathil 2022 (52)	Aged at least 18 years without a prior colorectal cancer (CRC) diagnosis	213,924 individuals undergoing colonoscopy between October 2007 and September 2018	The mean age of patients was 56.7 years (±13.1). Gender distribution was 55.3% female and 44.6% male. Ethnicity breakdown was 76.8% Caucasian, 7.0% African American, 5.1% Hispanic, 3.8% Asian, 3.4% Other, and 3.8% Unknown.	An extensive integrated healthcare system, Mass General Brigham, specifically analysed endoscopy quality data from colonoscopies performed between 2007 and 2018.	At least 305,656 documents were matched (endoscopy and histology). However, only 75,068 had polyps at index colonoscopy. 31.7% of patients had linked genetic data in the biobank.	Endoscopy reports, corresponding pathology reports and genetic data from the Partners Biobank.	The study utilised a modified and extended 5-step NLP pipeline to extract detailed pathological data from endoscopy and pathology reports. Additionally, a high throughput NLP pipeline was employed to match endoscopy and histology data for a large patient cohort, identifying patients with polyps and enabling investigations into CRC risk factors.	Pathology reports from a randomly selected sample of 500 colonoscopies in the Partners Colonoscopy Cohort were manually reviewed by a study physician (M.V). Any questions about the coding were addressed through discussion with another study investigator (M.S).
Nayor 2018 (53)	Population undergoing screening colonoscopies between June 2010 and August 2015.	8032 patients who underwent screening colonoscopies, performed by 24 endoscopists.	The study reports demographic values related to Adenoma Detection Rates (ADRs), with male ADR at 35.7% and female ADR at 24.9%. However, specific information on the demographics	Screening colonoscopies performed between June 2010 and August 2015.	The specific number of documents is not given.	The study utilised colonoscopy and pathology reports extracted from the institutional Research Patient Data Registry (RPDR), which stores data from electronic health record systems like	The NLP tools used in this study included a Sectionizer, Sentence splitter, Findings annotator, Location assignment, and Negative status detection in the NLP pipeline to identify adenomas and Sessile Serrated Polyps (SSPs) in colonoscopy and pathology reports.	The researchers derived the validation dataset by randomly dividing the cohort into a training set of 500 colonoscopies and a test set of 100 colonoscopies, reviewed by two independent, blinded reviewers (JN and LB).

			of the underlying dataset needs to be given.			Epic, Provation®, and Sunquest PowerPath.		
Parthasarathy 2020 (59)	Patient population undergoing colonoscopy. Attempting to identify Serrated Polyposis Syndrome (SPS) within this cohort.	255,074 distinct patients underwent 323,494 colonoscopies between 2012 and 2016.	Demographic information is not given	Outpatient colonoscopies performed in a hospital system (Cleveland, Ohio, USA)	30,000 cases (colonoscopy and pathology reports) from 323,494 colonoscopies performed from 2012 to 2016.	Colonoscopy and pathology reports	Prolog for natural language processing (NLP) to parse colonoscopy and pathology reports. A SAS program, "the SPS tool," was developed to combine data from all procedures for each patient and identify those with Serrated Polyposis Syndrome (SPS).	Data extraction accuracy was assessed as the percentage of agreement based on a manual review of variables. 71 patients were identified with SPS by the SPS tool. Most of these patients met WHO criterion 1 (70/71), and one met WHO criterion 3. A manual chart review of the EMR of these 71 patients confirmed that the SPS diagnosis was correct in 66 patients (93%). The incorrect diagnosis of SPS in the remaining 5 cases occurred because of errors in polyp data extraction by NLP. No further details are given on the derivation of the core cohort.
Laique 2021 (54)	Patients undergoing colonoscopy procedures	The number of patients is not given	Demographic information is not given	Colonoscopy procedures for colorectal cancer screening in the United States.	589 colonoscopies.	Colonoscopy reports, pathology reports, and electronic health records (EHR) from the Epic, ProVation, and Sunquest PowerPath systems for data extraction.	A natural language processing (NLP) algorithm developed in Prolog by a member of the Cleveland Clinic Digestive Disease & Surgery Institute was employed. A Java-based NLP tool previously validated by the Pittsburgh Medical Center healthcare system was also employed. The study also mentioned using cTAKES, another Java-based NLP system developed by the Mayo Clinic, and a Java-based NLP tool by Gawron et al.	An initial manual review by one of the authors (J.M.) showed 88% agreement with the parsed data in a prior study. The selection process in this study involved arranging patients in numerical order of their medical record number and including every third patient. These patients were manually reviewed.<br>by two researchers (SL and MM). Any discrepancies were resolved by a third researcher (SS).
Peterson 2021 (39)	Individuals with colorectal polyps undergoing	224 individuals with colorectal polyps undergoing	Demographic information is not given.	Individuals with colorectal polyps	346 documents, 224 colonoscopy reports and 122	Colonoscopy reports and pathology reports	spaCy, a Python tool for natural language processing (NLP) tasks, along with	The researchers derived the validation dataset by randomly selecting 100

	colonoscopy surveillance.	colonoscopy surveillance.		undergoing colonoscopy surveillance in a clinical setting involving the Department of Medicine, Division of General Internal Medicine and Health Services Research, David Geffen School of Medicine, University of California Los Angeles, Los Angeles, California, USA.	pathology reports used for evaluation. The validation set consisted of 100 colonoscopy and pathology reports, respectively.		various tools for colonoscopy quality metrics reporting such as those by Imler et al., Karwa et al., Nayor et al., etc.	documents and two independent reviewers then screened this.
Tinmouth 2023 (55)	The study is focused on patients aged over 50 years who underwent colonoscopies in Ontario, Canada	14 million Ontarians in the total population, but specific patient numbers of patients are not given for the study specifically	For age, it includes patients over 80 years old, with 21 individuals in this category (4.7%), 20.7% of the patients were aged 70-80, 36.4% 60-70 and 38.2% were aged 50-60 years old. In terms of sex, the study includes 252 males (56%)	Colonoscopies in Ontario within the Ontario healthcare system include 14 regional health networks, over 100 public hospitals, and around 70 private clinics.	23,060 colonoscopies and sigmoidoscopies with biopsy sampling and/or polypectomy performed between June 1 and June 30, 2015, were identified using OHIP claims data; however, a much smaller sample was	Pathology reports, health administrative databases, and documents related to colonoscopies, sigmoidoscopies, biopsy sampling, polypectomy, excision of tumours or strictures, and endoscopies of the large intestine for NLP algorithm validation.	Base SAS 9.4 for developing the natural language processing (NLP) algorithm - data preprocessing, concept recognition, contextual processing, and information integration.	The researchers derived the validation dataset by having two clinicians (K.C. and D.M.) annotate 1,000 reports in the validation set.

			and 198 females (44%).		used in the study. Specifically, there were 450 documents in the training set and 1,000 in the validation set.			
Redd 2022 (58)	Identifying colorectal cancer in US military Veterans aged 35-49.	4339 patients	The age ranges of the patients are given: 35-49.	Identifying colorectal cancer in US military Veterans aged 35-49 using structured and free text clinical data - in particular, colonoscopy reports	(n = 33,135) clinical notes are mentioned in the report	Pathology, colonoscopy, and other types of clinic documents, including oncology and haematology clinic letters.	Latent Dirichlet Allocation (LDA) for topic extraction from clinical notes and specific NLP tools for categorising indications, findings, and pathology reports from colonoscopies and pathology reports. The authors also used Theano and Lasagne (python libraries) to train and evaluate the logistic regression (LR), support vector machine (SVM), random forest (RF), and deep neural network (DNN) models.	Statistical and ML models were trained and tested using a subset of CDW data that had undergone a chart review for a case-control study on risk factors for early-onset CRC in Veterans. However, further details regarding the validation set derivation process are not given. To avoid overfitting in the DNN training, the data was partitioned into 70% training, 20% validation, and 10% testing groups, with performance being measured in the testing group only.
Blumenthal 2015 (47)	Patients undergoing outpatient colonoscopies at an academic medical center in the Northeastern United States.	The final study population was 321 patients, with 107 cases and 214 controls.	The information includes age (mean of 57 years), Gender (51% male), Education level, Insurance type, and History of polyps/CRC or psychiatric illness.	Patients are undergoing outpatient colonoscopies at an academic medical centre in the Northeastern United States from 2009 to 2011.	The core validation cohort is based on 1200 documents, but few details are given about the broader pool of patients and documents	Patient charts and records from outpatient colonoscopies.	The study utilised the Queriable Patient Inference Dossier (QPID), a natural language search engine developed at the institution, to develop and deploy regular-expression-based NLP queries for searching free text in patient Electronic Medical Records (EMR).	The researchers derived the validation dataset by randomly selecting 1,200 individuals from the study population. The final validation cohort comprised 1,114 patients, including 89 no-shows and 1,025 adherent patients. Chart reviews were performed by a research assistant and two physicians blinded to case/control status.
Lee 2019 (56)	A patient population who	The population in this research	Mean age of 62.8 years (+/-SD:8.6).	Colonoscopy patients in a	800 colonoscopy	De-identified colonoscopy	Linguamatics I2E NLP software from the United	The validation dataset was derived at random. A board-certified

	had undergone colonoscopy.	consists of 800 reports but the total number of patients is not given.	51% Females. 58.7% white patients.	clinical setting within Kaiser Permanente Northern California (KPNC), an integrated healthcare delivery organisation serving over 3.9 million members across 21 medical centres and hospitals in urban, suburban, and semirural regions.	reports were used for training and validation,	reports for testing the NLP tool to extract exam quality-related and extensive polyp information. The reports varied in format, including dictated, free text, and standardised templates, with no fixed text fields for direct data extraction	Kingdom to develop query strategies for identifying key concepts in colonoscopy reports.	gastroenterologist (JKL) manually reviewed the training set of 500 colonoscopy reports and assembled term and phrase variations for the variables of interest. Discrepancies in findings between the use of the NLP query strategies and manual review of the 500 colonoscopy reports were investigated through error analysis, and query strategies were then refined to reduce errors. Iterative development continued until the performance of the NLP tool reached a high level of accuracy.
Fevrier 2020 (37)	A patient population that underwent colonoscopy procedures at Kaiser Permanente Northern California.	610,684 patients who underwent 866,578 colonoscopies from 2006 to 2016 at Kaiser Permanente Northern California, with 401,566 procedures having linked pathology reports. Gastroenterologists who performed fewer than 300	For age, percentages are provided for the categories 50-59 (34%, 37%, 35%), 60-69 (38%, 37%, 38%), and ≥70 (29%, 26%, 28%) in the training, validation, and test sets respectively. Sex is reported as Male (50%, 55%, 53%) and Female (50%, 45%, 47%) in the same sets. Race percentages are given for	Clinical setting of colonoscopies and pathology reports at Kaiser Permanente Northern California (KPNC) from 2006 to 2016, involving patients aged 50 years and older who underwent colonoscopy procedures with linked	866,578 colonoscopy encounters in 610,684 patients at Kaiser Permanente, Northern California. Of these, 401,566 procedures in 312,776 patients had linked pathology reports. For validation, 3,000 linked colonoscopy-	Linked colonoscopy-pathology reports from Kaiser Permanente Northern California (KPNC) from 2006 to 2016. These reports included pathology reports, colonoscopy reports, and free-text reports.	The study utilised SAS® and PERL NLP tools with regular expressions to extract critical variables related to colonoscopy quality from free-text reports. Separate NLP algorithms were developed for each of the five variables: extent of procedure, quality of bowel preparation, and type, size, and location of polyps. These tools were used to define variables such as polyp type, location, size, colonoscopy extent, and quality of preparation from colonoscopy and pathology	The validation dataset was derived using a random sample of 3,000 linked colonoscopy-pathology reports from 2,991 patients. They state that manual review was used as the reference standard, but few details are given on how this was performed.

		colonoscopies or fewer than 100 screening colonoscopies were excluded from the study.	White (64%, 61%, 63%), African-American (6%, 6%, 7%), Asian/Pacific Islander (13%, 15%, 14%), Hispanic (9%, 10%, 9%), and Other (8%, 9%, 8%).	pathology reports signed within 8 days of the procedure.	pathology reports and 2,991 patients were used, while the test set comprised 397,566 linked colonoscopy-pathology reports in 310,319 patients.		reports. The associated code for this project is not declared in the paper but was found associated with the institution at: https://github.com/kpwhri/precise_nlp	
Shi 2022 (49)	Patients eligible for genetic testing for hereditary cancers based on family health history data in electronic health records, explicitly targeting high-risk patients with a history of hereditary cancers such as breast, ovarian, and colorectal cancers.	After splitting the data set, 2398 patients with 12,430 FHH entries were included in the NLP development or evaluation data set and 66,853 patients with 494,880 FHH entries were included in the NCCN algorithm evaluation data set.	Characteristic NLP development or evaluation data set (n=2398) NCCN algorithm evaluation data set (n=66,853) Gender (male):, n (%) 998 (41.2) 24,524 (36.7) Age (years):, mean (SD) 40.2 (9.6) 42.6 (9.9)Race, n (%) Caucasian: 1752 (73.2) 51,171 (76.5) Other: 359 (15) 9510 (14.2) Asian: 141 (5.9) 2973 (4.4) Black or African American: 67 (2.8) 1450 (2.2) Not reported: 56 (2.3) 1226 (1.8) American Indian or Alaska Native:	Patients eligible for genetic testing for hereditary cancers based on family health history data in electronic health records in a clinical setting at a US academic medical center.	2,430 FHH entries were included in the NLP development or evaluation data set and 494,880 FHH entries were included in the NCCN algorithm evaluation data set.	Electronic health records (EHRs) as documents for analysis, which contained structured and unstructured family health history (FHH) data, including coded disease, relationship, and age of onset information.	EasyCIE's rule-processing engine was used for entity reconciliation and heuristic rules development based on annotated data from the training set.	A total of 2 physicians designed the annotation schema based on the FHH attributes relevant to the NCCN guidelines for genetic testing of hereditary breast or ovarian and colorectal cancers. All NLP components of EasyCIE are configurable through rules without the need to develop new pipelines. A total of 1300 FHH entries were used to develop the rules. They evaluated the NLP solution by comparing its output with the test set annotations of the snippet-level data set (1000 FHH entries). To save time and effort, entities with no relation were not annotated (eg, an entry that only has a condition without mentioning any family member); therefore, they did not evaluate the NLP performance for named entity recognition

			17 (0.7) 523 (0.8) Hispanic ethnicity: 327 (13.6) 9147 (13.7)					
Bae 2022 (57)	Patients aged 50 years and above who underwent screening and surveillance colonoscopies at Seoul National University Hospital Gangnam Center	36,119 patients examined between 2003 and 2019 were derived from SUPREME (Seoul National University Hospital Patients Research Environment),	The mean age for the testing data set was 60.4 years with a standard deviation of 6.5, and for the training data set, it was 58.6 years with a standard deviation of 6.4. In terms of sex, 59.0% were male and 41.0% were female in the testing data set, while in the training data set, 59.4% were male and 40.6% were female.	Colonoscopies , specifically screening and surveillance colonoscopies , at Seoul National University Hospital Gangnam Center between January 2010 and December 2019.	2000 screening colonoscopy reports from a single health care system, with an associated 1425 pathology reports. The NLP system was then tested on a data set of 1000 colonoscopy reports and its performance was compared with that of 5 human annotators. Additionally, data from 54,562 colonoscopies performed between 2010 and 2019 were analyzed using the NLP pipeline.	Colonoscopy and pathology reports	Regular expressions in Python (3.7.10) and smartTA (1.0b) were used for developing the NLP pipeline, which involved text preprocessing, information extraction, and summarization of colonoscopy and pathology reports. Regular expressions were employed for complex text processing, while smartTA aided in linguistic pattern analysis and lexicon construction.	The NLP system was tested on a data set of 1000 colonoscopy reports and its performance was compared with that of 5 human annotators.
Patterson 2015 (50)	Patients who underwent colonoscopy procedures at VA facilities within the Department of	The number of patients is not given.	Demographic information is not given.	Patients who underwent colonoscopy procedures at VA facilities within the Department	3,000 documents, with 2,000 in the training dataset and 1,000 in the validation	Colonoscopy reports. These reports included narrative descriptions.	VINCI Leo framework for NLP, based on the Unstructured Information Management Architecture Asynchronous Scaleout (UIMA AS). Specific NLP modules included document type detection,	Each document was independently reviewed by 2 chart abstractors and classified as “Screening”, “Non-screening”, “Non-colonoscopy”, or “Unknown” using an open-source annotation tool eHOST. Annotation disagreements were adjudicated by a gastroenterologist.

	Veterans Affairs Healthcare System Database.			of Veterans Affairs healthcare system from fiscal years 2009 to 2012.	dataset. The test dataset consisted of 1,000 documents.		indication section identification, and non-screening indication detection. The system employed regular expressions and logic modules for rule-based classification based on identified keywords and phrases within colonoscopy reports. Additionally, the study mentioned the use of the automated retrieval console (ARC) for clinical information retrieval and the Mayo Clinical Text Analysis and Knowledge Extraction System (cTAKES) for text analysis and knowledge extraction. The primary ML model is a bag-of-words (BOW) model.	Annotator inconsistency was noted when the same phrase was marked as being indicative of different categories. The conflicting documents were reviewed again by the gastroenterologist, and discrepancies were resolved.
Ternois 2018 (60)	The patient population was undergoing diagnostic digestive endoscopies at the Avicenne Hospital in Bobigny, France, between 2015 and 2016.	The number of patients is not given.	Demographic information is not given.	Diagnostic digestive endoscopy reports	3586 endoscopy reports were collected, but the primary test set in the study featured only 1639 endoscopy reports.	Endoscopy reports were collected between 2015 and 2016 at the Avicenne Hospital in Bobigny, France.	Preprocessing steps to remove French accents and special characters, conversion of reports into matrices containing unigrams and bigrams with TF-IDF scores, and classification algorithms tested with unigrams and bigrams. The reports were classified into six classes, including five CCAM codes and a class labelled "Other." The study evaluated four algorithms provided by the Python Scikit-learn library: RandomForestClassifier,	The associated procedure CCAM codes were extracted. This selection was made with the help of the gastroenterologist, who also specified the anatomical bounds of each procedure. A gold standard dataset was created by using the initial CCAM codes and cleaning it using a semi-automatic method, flagging a report when the associated code does not match the requirements of its description, such as a report coded like a complete colonoscopy but not containing a mention of the caecum. The label "Other" has been attributed to the reports not being within

							LinearSVC, MultinomialNB, and LogisticRegression, selecting the best algorithm based on macro average F1 score obtained through 5-fold cross-validation.	the scope of the study (non-diagnostic digestive endoscopies).
ERCP & Sedation								
Imler 2018 (64)	Patients who underwent Endoscopic Retrograde Cholangiopancreatography (ERCP) procedures	13,299 patients underwent a total of 23,674 Endoscopic Retrograde Cholangiopancreatography (ERCP) procedures.	The mean patient age is 52.9 years, with 59.6% of patients female and the majority being Caucasian (75.9%).	Endoscopic Retrograde Cholangiopancreatography (ERCP) procedures in a US Setting	63,119 documents initially, with 39,440 documents excluded as they were not procedure reports, leaving 23,679 ERCP procedure reports.	ERCP reports	An Apache Unstructured Information Management Applications (UIMA™)- based NLP system called nDepth was used. This system employed NLP techniques such as negation, regular expressions, and standard terminologies for processing text documents. Other tools mentioned in the study include ICD-9 CM coding. NLP extraction was specifically focused on quality indicators like deep cannulation of ducts, pancreatic cannulation, pancreatic injection, pancreatic stent placement, and precut sphincterotomy.	Individuals screened the validation dataset to ensure accuracy. However, the researchers only manually screened/annotated 50-100 documents. Few details are given on this process.
Shen 2021 (33)	Patients undergoing endoscopy procedures	22,755 endoscopy cases, but the number of patients is not specified	The mean patient age was similar between the pre-pilot and pilot periods (P = .88). Female predominance was observed in both periods, with 62.0% in the pre-pilot and 61.8% in the pilot, showing no significant difference (P = .79).	Patients undergoing endoscopy procedures in a clinical setting at a single academic medical center.	22,755 endoscopy reports	Endoscopy records and records sedation-type order errors.	Parsing free-text data, detecting free-text recommendations in prior endoscopy reports, and flagging relevant free-text through heuristic checks of keywords, partial word fragments, abbreviations, and misspellings.	The researchers validated their algorithm live via a human-in-the-loop pilot. A simple NLP using a heuristic check of certain keywords was used to parse free-text data. Requirement 2 (high-level): The CDSS must integrate into the existing workflow with a human check. The reintegration point needed to give ultimate decision-making responsibility to an endoscopy triage nurse, a health professional qualified to make such a medical decision. A formatted report of “high-risk patients” was sent regularly to an endoscopy triage nurse for manual review. A retrospective analysis was conducted to evaluate the effectiveness of the CDSS on

								sedation-type order errors at the time of endoscopy.
Gastrointestinal Bleeding								
Shung 2021 (40)	Patients with acute gastrointestinal bleeding (GIB) in the emergency department (ED)	10,132 patients, with 7,144 patients in the training/internal validation dataset and 2,988 patients in the external validation dataset.	Male (48-49%), Mean Age: 56-57, White 45%. 66% of patients were admitted.	Emergency department (ED)	10,132 encounters, with 7,144 patients in the training/internal validation dataset and 2,988 patients in the external validation dataset. 650 patients with hematemesis and/or melena out of 2,988, and 1,316 patients with hematochezia out of 2,988 were in the validation set, but the number of documents is not given.	The study utilised unstructured text notes written by physician providers in the emergency department.	SCISPACY was used for advanced natural language processing (NLP), syntax-based NLP algorithms, the elastic net classifier, support vector machines, and the BERT neural network model. These tools demonstrated improved precision, sensitivity, and specificity performance compared to SNOMED codes for identifying acute gastrointestinal bleeding.	They derived the validation dataset by selecting 30% of the encounters, resulting in 2,988 patients for external validation. The gold standard for GIB diagnosis was the independent dual manual review of medical records performed by two clinical domain experts (DS and CT). They reviewed the medical records of all patients and classified each patient as having the phenotype or not based on expert opinion and a prespecified structured evaluation text.
Taggart 2018 (65)	Critically ill patients in ICU (MIMIC III)	The study population consists of 1,296 patients, with 769 unique patients in the training set and 527 unique patients in the test set.	Mean ages(67.42 years for training, 67.86 years for test). Females were 38.5% in the training set, and 40% in the test set.	Critically ill patients in ICU (MIMIC III)	There were 1,650 documents, with 990 notes in the training set and 660 in the test set. [MIMIC III is available at: https://physionet.org/content/mimiciii/1.4/]	Clinical notes from critically ill patients in a healthcare setting to identify bleeding events using natural language processing methods.	Python's pyConText software package was used for rule-based NLP tasks to identify bleeding events in clinical notes. The ML models used in the study included SVM, ET, and CNN, and they were trained using term frequency–inverse document frequency vectors and global vectors for word representation.	Two physicians (R.U.S. and B.A.S.) reviewed the batches of notes, and a third physician (B.T.B.) adjudicated discrepant annotations. Each reference to bleeding in the note was classified as bleeding present or absent (mention-level annotation).
Gastroscopy								
McVay 2018 (68)	Patients with dysphagia indications undergoing	1,000 patients with dysphagia indications undergoing	Demographic information is not given	Oesophagogastroduodenoscopy (OGD/EGD)	396,856 Esophagogastroduodenoscopy (EGD) notes from	Oesophagogastroduodenoscopy (OGD/EGD) procedure notes,	The Extensible Human Oracle Suite of Tools (eHOST) is used to manually annotate OGD/EGD	All documents underwent dual annotation and adjudication for disagreement for dysphagia indications. Discrepancies were resolved by

	Oesophagogastroduodenoscopy (OGD/EGD) procedures within a large US-integrated healthcare system, specifically within the national VA healthcare system.	Esophagogastroduodenoscopy (EGD) procedures within the national VA healthcare system and US Veterans from 2010 to 2014.		procedure reports	123 VA sites in the United States from 2008-2014. However, only a random sample of 1000 notes was used for development (N=250), training (N=250) and final test (N=500) sets.	patient demographics, and ICD codes associated with OGD/EGD procedures were used in this study.	procedure notes to identify dysphagia indications. Additionally, the study employed pyConText, an enhanced Python implementation of the ConText algorithm, for natural language processing (NLP) to identify dysphagia indications in the notes.	adjudication from a clinical domain expert (Dr. Gawron).
NguyenWenker 2023(69)	Patients with Barrett's Oesophagus	1,000 patients with Barrett's Oesophagus (BE) within the National Veterans Affairs (VA) system.	No demographic information is given.	Patients with Barrett's Oesophagus (BE) within the US National Veterans Affairs (VA) system.	The development cohort appears to have contained 457 patients. However, the specific number of documents is not given.	Full-text pathology reports from patients with Barrett's Oesophagus	Clinical Language Annotation, Modeling, and Processing (CLAMP) software for the development and validation of a Natural Language Processing (NLP) algorithm to identify dysplasia in pathology reports of patients with Barrett's Esophagus (BE) within the National Veterans Affairs (VA) system.	Randomly split dataset. The presence of BE and the presence/grade of dysplasia was determined by 2 independent reviewers (K.C. and F.N.), and verified with an expert gastroenterologist (Y.N.) in cases of ambiguity.
Ding 2020 (38)	A patient population underwent gastroscopy and pathological examinations, with data acquired from March 2004 to June 2017 from the Department of Gastroenterology of the First Affiliated Hospital of Anhui Medical University, China.	Patient numbers are not given	Demographic information is not given	Gastroscopy reports and pathological examinations at the Department of Gastroenterology of the First Affiliated Hospital of Anhui Medical University, China, from March 2004 to June 2017.	15,955 case samples were obtained; however, only 8,546 were retained as complete.	Gastroscopy reports.	ML tools such as LR (Logistic Regression), SVM (Support Vector Machine), XGBoost, and MLP (Multi-Layer Perceptron) were utilised to predict gastric cancer based on gastroscopy reports. The PLWA-based homogeneous weighted ensemble method significantly improved gastric cancer screening performance compared to AUC and average weighting-based methods.	The researchers derived the validation dataset by randomly selecting 80% of the total gastroscopy reports for training (X_train) and allocating the remaining 20% for testing (X_test). They used PLWA to set the weights. The training set included 2,803 cancer-positive and 4,033 cancer-negative reports, while the test set comprised 704 cancer-positive and 1,006 cancer-negative reports. The validation dataset was screened using LR, SVM, XGBoost, and MLP models for predicting gastric cancer based on gastroscopy reports; however, no individuals who screened the validation dataset are explicitly mentioned in the provided text.
Song 2022 (67)	A patient population of individuals undergoing oesophagogastro	Population of 140,694 patients who underwent oesophagogastro duodenoscopy	Age values are categorised into age groups (<30, 30–49, 50–69, ≥70) with corresponding	Oesophagogastroduodenoscopy (OGD/EGD) reports at Seoul National	The NLP pipeline was applied to 248,966 consecutive EGD reports, and	Oesophagogastroduodenoscopy (OGD/EGD). These reports encompassed three	Python (3.7.10) and the regular expression package 're' for processing texts in Korean, English, or a combination of both	A validation dataset was created by randomly selecting 1,000 documents. The NLP pipeline was assessed by calculating the overall agreement between it and the gold standard

	duodenoscopy (OGD/EGD) at Seoul National University Hospital.	(OGD/EGD) between 2003 and 2019. However, the core training dataset was 2000 with a test dataset of 1000 patients.	percentages for each group. ~61% of participants were female and ~42% had atrophic gastritis with metaplasia reported for ~23%.	University Hospital between 2003 and 2019.	50,096 associated pathology reports in 97,998 patients. However, the core dataset included only 3000 training and validation documents.	types of language forms: Korean, English, and Korean with English terminology. They focused on processing multi-language reports and building a lexicon of medical terms, synonyms, and endoscopic abbreviations.	languages. The NLP pipeline focused on extracting information on gastric diseases from esophagogastroduodenoscopy (EGD) reports, including terms related to findings and impressions of gastric diseases. A customised NLP dictionary was created based on medical terminology sources to process multi-language reports and build a lexicon of medical terms, synonyms, and endoscopic abbreviations.	created by two gastroenterologists with manual annotation.
IBD								
Gomollón 2022 (75)	Individuals with Crohn's disease in a Spanish registry	5938 individuals with Crohn's disease drawn from a population of 2242730 patients	Mean age (48.3 years), percentage of adults (97.37% ≥18 years old), gender distribution (51.1% female).	Crohn's disease relapses in a clinical setting, analysing predictors such as patient age, leukocytes, haemoglobin, and fibrinogen levels.	The specific number of documents is not given.	Electronic health records (EHRs) and clinical notes, particularly physician notes, are also used.	Commercial Savanna algorithm described	The researchers derived the validation dataset by randomly splitting the patient population into a training set comprising 70% of the patients and a validation set comprising 30%. The human-annotated corpus, or 'gold standard,' is only briefly described in minor detail.
Hou 2016 (76)	National Cohort of Veterans with Inflammatory Bowel Disease (IBD).	44,099 veterans with IBD drawn from a larger population of 74,258 unique Inflammatory Bowel Disease (IBD) patients	Demographic information is not given	A national cohort of Veterans with Inflammatory Bowel Disease (IBD), specifically focused on pathology reporting.	22,431 colonoscopy-related histopathology reports drawn from a larger sample of 116,338 available pathology reports. A validation cohort was created using a random sample of 2000 reports.	Colonoscopy-related histopathology reports.	Clinical Language Annotation, Modeling, and Processing (CLAMP) was used to develop and validate the NLP algorithm. The pipeline identified diagnosis and related comment sections from reports, processed intended concepts and phrases, and assigned dysplasia status using rule-based methods.	The researchers derived the validation dataset by manually adjudicating a random sample of 2000 Inflammatory Bowel Disease (IBD) colonoscopy pathology reports. Classification of dysplasia was determined by two reviewers, and discrepancies were adjudicated by a third reviewer.
Stidham 2022 (70)	A patient population of	4108 individuals with	The mean age of the patient population	A single-centre retrospective	The study mentions	Outpatient gastroenterology	An NLP system was developed using Python 3.6 to identify	Reviewers labelled a preliminary set of 150 documents for training and to discuss, clarify,

	individuals with inflammatory bowel disease (IBD).	inflammatory bowel disease (IBD), with 1240 unique patients selected for analysis based on the presence of one or more extraintestinal manifestations (EIMs).	is 41.8 years, with a standard deviation of 14.2 years. The gender distribution is 47.4% male.	clinical outpatient setting.	reviewers labelling a preliminary set of 150 documents for the validation set, but further details of the number of documents are not given	notes extracted from electronic medical records for identified patients.	extraintestinal manifestations (EIMs) in outpatient gastroenterology notes. The NLP pipeline involved preprocessing steps like the removal of extraneous characters, tokenisation of phases, and part-of-speech labelling. A SECtag approach was employed to determine the document section containing EIMs.	and revise EIM status definitions and establish uniformity in handling ambiguous documentation. In the full document set labelled for training and testing, EIM status disagreements were adjudicated by both reviewers based on discussion and consensus; if consensus was not possible, EIMs were labelled as the uncertain class.
Walker 2016 (73)	A patient population of individuals with inflammatory bowel disease (IBD), specifically those with associated acute liver dysfunction.	29,336 individuals with IBD	The patient population was 59% female, with ages at first IBD diagnosis ranging from 18 to 79 years. The most common diagnoses were ulcerative colitis and regional enteritis.	Electronic health records (EHR) in individuals with inflammatory bowel disease (IBD) and acute liver dysfunction (ALD)	The specific number of documents is not given.	Electronic health records (EHR).	SNOMED and MedDRA dictionaries were used to capture patient complaints in a structured format, including attributes like intermittency, severity, and duration. Humedica's data and tooling were involved.	The enriched population was initially chosen using attributes considered by the two physician authors (AA, a gastroenterologist, and AW, a drug-safety epidemiologist) to include all cases that might include ALD and from consideration of codes and terms identified from previous literature reviews and efforts at case definition. After the first round of case review, the two reviewers dropped several nonspecific diagnoses from the inclusion criteria. They added other findings that should qualify an individual as a possible case.
Zand 2020 (72)	Patients with inflammatory bowel disease (IBD) contacting the flare line	424 patients with inflammatory bowel diseases using the ULCA eIBD service	The study reports demographic values for a patient population with inflammatory bowel diseases, including 51.9% female, 50.7% with Crohn's disease, 46.9% with ulcerative colitis, 67.0% white, 66.7% employed, and a mean enrollment in the care program of 4.6 years.	Patients with inflammatory bowel diseases (IBD) enrolled in the University of California, Los Angeles (UCLA) Center for IBD Electronic Care Management Platform (UCLA eIBD)	16,453 lines of electronic dialogue, with 8324 lines sent by 424 patients to their healthcare providers.	The study analysed electronic messaging data, patient narrative texts, and electronic health records (EHRs).	Bag-of-words model for categorisation based on keywords extracted from patient-to-healthcare provider dialogues.	Three independent raters categorised 100 random lines of dialogue in the exact style of the algorithm used in the study. The raters were AZ, DH, and CR. The validation dataset was then compared using Krippendorf alpha to assess agreement between the raters and the algorithm. Two physicians screened the validation dataset.
Montoto 2022 (74)	Patients with Crohn's disease in Spanish electronic health records.	800 patients with Crohn's disease in Spanish electronic	The gold standard data set included 41.4% with Crohn's disease, 21.3% with	Patients with Crohn's disease in a Spanish	800 clinical documents from 800 patients with Crohn's disease in	The study used clinical documents from the gastroenterology service, including	The study utilised the EHRead technology, a clinical natural language processing (NLP) system, to identify Crohn's	The researchers derived the validation dataset by randomly selecting 100 records from each of the eight sites containing electronic health records (EHRs) with and without Crohn's

		health records across 8 sites.	Crohn's disease flare, and 10% with vedolizumab treatment. However, no data is given on specific demographics such as age, sex or ethnicity.	electronic health registry.	Spanish electronic health records.	consultation, hospitalisation, and emergency reports.	disease-related variables in Spanish electronic health records. The system's terminology is based on SNOMED-CT, a comprehensive medical concept terminology.	disease-related information. Physicians then annotated the validation dataset to generate a gold standard. Additionally, 15 records per site were reviewed by two independent annotators to assess interannotator agreement. After resolving disagreements, the final gold standard was built, and one of the two physicians annotated the remaining 85% of clinical records to complete the gold standard. Interannotator agreement values were higher than 0.8 for all comparisons.
Kurowski 2022 (71)	Crohn's disease patients	7033 patients with Crohn's disease, including 4963 adult-onset patients and 2060 pediatric-onset patients.	The results contain references to the paediatric and adult populations under review. However, they do not contain details of clinical demographic information.	Crohn's disease patients in a clinical setting, explicitly utilising electronic medical records to develop a computable phenotype for Crohn's disease.	232 encounters to validate objective test results and symptoms are mentioned, but not the number of documents.	Electronic medical records (EMRs).	The clinical notes use ICD 10 codes and UMLS to identify IBD patients, but few details are given on this. Health state utility assessments, such as the EQ Index Score, EQ Visual Analog Scale, and Paper Standard Gamble, were used for adult and pediatric Crohn's disease patients.	The validation dataset was derived using a subset of 500 adult-onset and 400 pediatric-onset patients for specificity and positive predictive value assessment. The results were based on manual chart validation, but few details are given about how this was performed.
Liver								
Bell 2022 (34)	Deceased donors and associated liver transplant outcomes.	104,607 deceased donors from 2001 to 2021, with 17.9% being Donation after Circulatory Death (DCD) donors. For the mortality model cohort, 75,591 transplanted donor livers were analysed.	Demographic values such as age, DCD status, race, height, and causes of death are reported. The average age for the utilisation and mortality cohorts is 40 and 39 years old, respectively, with a standard deviation of 17 years for both. The three largest demographics for both cohorts were White (66% for utilisation, 64% for mortality), Black (16% for utilisation,	US Donor registry complete with donor text narratives.	The specific number of documents is not given.	The study utilised unstructured text documents from DonorNet and free-text clinical notes from donor evaluations.	Natural Language Processing (NLP) techniques, including stemming with the Porter stemmer, removal of stop words using the NLTK package in Python, and conversion of unigrams and bigrams to a vector of term-frequency inverse-document-frequency (TF-IDF) values using the default Sci-Kit Learn implementation. Logistic regression, random forest, and gradient-boosting classifiers were employed to predict liver utilisation outcomes. A stacking classifier, composed of four logistic regression models, was also utilised to address class	A random 75%/25% training/validation data split was used. Then, a separate stacking classifier was also built, utilising a 56.25%/18.75%/25% split for training, validation, and test sets with validation data derived from actual donor outcomes. Validation level 2a or greater.

			18% for mortality), and Hispanic (14% for both utilisation and mortality).				imbalance in the liver utilisation dataset.	
Heidemann 2017 (81)	Patients with idiosyncratic drug-induced liver injury (DILI)	101 DILI cases out of a larger pool of 2564 potential DILI cases	The mean age of probable DILI cases is 51 years +/- SD:18, with 63% being female. 11% of cases had underlying liver disease, and the median duration of suspect drug use was ten days (ranging 1 day to 3 years). The median RUCAM score for probable DILI cases was seven points (range -3 to 11).	Patients with idiosyncratic drug-induced liver injury (DILI) in various clinical settings, including outpatient, emergency room, and hospitalised cases. Including cases from the DILIN and Idiosyncratic Liver Injury associated with Drugs (ILIAD) retrospective studies. The ILIAD study was ongoing and required subjects to sign informed consent.	4577 documents are mentioned in the total cohort, but the number of documents in the core cohort is not given	Electronic Medical Record (EMR) data. The study also involved documents from the DILIN and ILIAD retrospective studies, but no specifics were given.	The study utilised natural language processing (NLP) algorithms to identify idiosyncratic drug-induced liver injury (DILI) cases using liver injury terms. Still, little in the way of specifics is given other than that a series of 4 iterative searches was undertaken to identify the optimal search terms.	The validation dataset included 101 DILI cases identified during a 17-month study period and was selected via expert opinion causality assessment and manual checking of case notes.
Redman 2017 (79)	A patient population of veterans was screened with readable abdominal image reports. The study subjects included veterans nationwide, and Fatty Liver Disease (FLD) was determined based on specific criteria defined by experienced	1000 veterans were screened for abdominal image reports, with 652 patients having readable reports.	No demographic information is given.	The algorithms were developed and validated for ultrasound, CT scans, and MRI reports. Concordance rates between different imaging modalities were also reported, showing agreement percentages for	1199 imaging studies from 652 individuals available selected from a population of 8 million veterans.	Veterans Affairs (VA) Corporate Data Warehouse (CDW) radiology reports.	Clinical Language Annotation, Modeling, and Processing Toolkit (CLAMP).	Radiographic fatty liver disease was determined by manual review by two physicians and verified with an expert radiologist. The validation cohort included 136 ultrasound reports, 247 CT scans, and 17 MRI reports and was randomly selected 70/30%. At least Type 2a validation.

	radiologists and hepatologists.			FLD assessment.				
Wang X 2022 (82)	Patient population with idiosyncratic drug-induced liver injury (iDILI)	175 patients with idiosyncratic drug-induced liver injury (iDILI)	Demographic information is not given	Idiosyncratic drug-induced liver injury (iDILI) in patients, specifically exploring causal inference in free text using an AI-powered framework called DeepCausality.	The exact number of documents is not given. Only 175 patient clinical case reports are described.	The study utilised text patient case reports for causal inference in patients with idiosyncratic drug-induced liver injury (iDILI).	Natural language processing (NLP) tools such as InferBERT, BioBERT, and BERN for causal inference in patients with idiosyncratic drug-induced liver injury (iDILI). Specifically, NER-based Do-calculus and Biomedical-based named entity recognition (NER) were employed to identify causal terms related to iDILI. The study also integrated the Do-calculus causal function into the BioBERT source code for analysis. The code for this project is available at this URL: https://github.com/XingqiaoWang/https-github.com-XingqiaoWang-DeepCausality-LiverTox	87 Biomedical-based name entities were taken, and 24 were enriched (with an adjusted p-value of < 0.05). However, only 18 terms in the end were felt to be highly consistent with clinical knowledge and, in the end, retained. Further details on the clinical validation process are not given.
Liu W 2022 (41)	Patients with colorectal cancer (CRC) undergoing CT/MRI examinations of the upper abdomen.	2790 patients with colorectal cancer (CRC) undergoing CT/MRI examinations of the upper abdomen between October 1, 2014, and April 30, 2021.	Demographic information is not given	CT/MRI examinations of the upper abdomen.	The specific number of reports adds up to the same number of patients so the assumption is 2790 documents but this is not explicitly clarified in the text.	The examination methods used were CT without contrast (10.2%), CT with/without contrast (73.3%), MRI without contrast (1.8%), and MRI with/without contrast (14.6%).	ML tools such as XGBoost, LR (logistic regression), RF (random forest), multinomial NB (naive Bayes), MLP (multilayer perceptron), KNN (k-nearest neighbours), and SVM (support vector machine) for text analysis and classification of liver results from medical imaging reports of colorectal cancer patients undergoing CT/MRI examinations of the upper abdomen. XZ was responsible for the NLP of text, and the study involved the construction of different algorithm models by XW and YCL. The NLP model used was a bag-of-words model.	Two individuals, YWL and ZY, screened the validation dataset and finalized the published version. They describe manual binary flagging of the documents with 0 and 1 for the presence/absence of a term but no more.

VanVleck 2019 (80)	Patients with non-alcoholic fatty liver disease (NAFLD) and those at risk for disease progression.	38,575 patients	Mean age (59.8), percentage male (42%), race distribution (African American: 20%, Caucasian/European : 23%, Asian: 2%, Hispanic: 48%, Other: 6%), mean liver serology at baseline (Aspartate Aminotransferase: 45.7, Alanine Aminotransferase: 48.5), baseline comorbidities (Diabetes Mellitus: 40.7%, Hypertension: 68.6%), and mean Body Mass Index (31.8).	Identifying patients with non-alcoholic fatty liver disease (NAFLD) from electronic health records in the Mount Sinai BioMe cohort.	7,766,654 notes from 38,575 BioMe enrollees from July 8, 2002, through December 31, 2017. However, the validation cohort appears to have been only 200 documents.	Clinical documentation such as progress notes, radiology reports, discharge summaries, and pathology reports	CLiX clinical NLP engine by Clinithink to map patient facts in clinical narratives to post-coordinated SNOMED expressions.	We compared all approaches (NLP/text search/ICD) to manual validation using a blinded manual chart review. Two physicians independently, without knowing case/control status, reviewed all records on 200 patients, 100 case patients identified as having NAFLD and 100 randomly (from a similar cohort, as explained below) selected patients identified as controls.
Yim 2017 (35)	University of Washington Medical Center hepatocellular carcinoma patients.	160 University of Washington Medical Center hepatocellular carcinoma patients.	Demographic information is not given.	Tumor event attribute classification in radiology reports of hepatocellular carcinoma patients at the University of Washington Medical Center.	101 abdomen radiology reports from 160 University of Washington Medical Center hepatocellular carcinoma patients	Abdominal Radiology Reports	Biomedical ontology, ClearNLP, MALLET toolkit, n-gram features, lemmatisations, Unified Medical Language System (UMLS) features, dependency features, shortest path through dependency tree features, entity-specific features, rule-based entity features, assertion classifiers, sentence windows, word windows, and specific features like ASSERTUMLS, CLOSESTCERTAINTYCUE, CLOSESTLESIONITEM, CLOSESTNEOPLASM, ENHANCEWASHOUT, LIRADS, NUMMEAS, NUMTUMREF, CLOSESTDATE, and SURRUNI were used to classify tumor event attribute classification in radiology reports of	Annotation, chiefly semantics-related, was performed by a biomedical informatics student and medical student and used to validate the classifier's effectiveness.

							hepatocellular carcinoma patients.	
Koola 2018 (77)	A patient population of cirrhotic patients with acute kidney injury (AKI) who experienced hepatorenal syndrome (HRS) during hospitalisation.	504 patients were used in the gold standard cohort	The mean age of the patients with HRS is 60 years, with a standard deviation of 7.9 years. Most patients are male, comprising 99% of the study population. 73.3% of the HRS patients were Caucasian.	Hepatorenal syndrome in cirrhotic patients during hospitalisation among 124 medical centers in the Department of Veterans Affairs	504 hospitalisations, but the specific number of documents involved in the study or validation and test set numbers were not provided in the text.	Electronic health records (EHR) data, patient charts, and clinical notes authored by physicians and advanced practice providers.	clinical Text Analysis Knowledge Extraction System (cTAKES) version 3.2 was used for converting documents into Concept Unique Identifiers (CUIs) mapped to the Unified Medical Language System (UMLS). High-throughput phenotyping methods, specifically AFEP and SAFE, were employed, selecting thirty-six and three CUIs, respectively. NLP strategies included a priori CUI selection, semantically informed clustering, and document embedding using CUIs, which significantly improved performance compared to raw text usage.	Based on manual annotation, there were 87 cases with Type I HRS, 19 with Type II HRS, 16 with Type Indeterminate, 88 with Maybe HRS, and 294 without HRS. However, further details on manual validation are not given.
Tariq 2022 (83)	Patients at high risk for hepatocellular carcinoma (HCC), including those with cirrhosis and/or chronic hepatitis B viral infection, who undergo imaging surveillance for early detection of HCC.	The number of patients is not given	Demographic information is not given	Hepatocellular carcinoma (HCC) screening using magnetic resonance imaging (MRI) at Emory University Healthcare (EUH) and Stanford Health Care (SHC).	10,018 MRI exams were performed between 2010-2019 for HCC screening. The US reports included 1462 documents split into training and test sets, with 29 'malignant' and 264 'benign' reports in the test set. The MR reports consisted of 944 documents split into training and test sets, with 81 'malignant' and 108 'benign' reports in the test set.	Templated ultrasound (US) reports from the Stanford dataset and templated magnetic resonance (MR) reports from the Emory University Healthcare (EUH) MRI dataset for hepatocellular carcinoma (HCC) malignancy scoring.	Word2Vec, Glove, BERT, ELMo, 1DCNN, and NLTK for language modelling, classification tasks, and text normalisation.	The validation dataset for the study was derived by using templated ultrasound (US) reports from the Stanford dataset, with varying numbers of documents screened by different individuals. Additionally, for the magnetic resonance (MR) reports from the Emory University Healthcare (EUH) dataset, the validation dataset was obtained by selecting 112 unstructured MR reports, with LI-RADS scores assigned by two expert radiologists.

Chang 2016 (78)	Patients with cirrhosis within the UCLA primary care population	5343 patients with chronic liver disease were identified within the UCLA primary care population.	Demographic information is not given.	A primary care setting at UCLA utilising an algorithm with natural language NLP applied to radiologist reports for abdominal imaging.	No details are given on the number of documents.	Abdominal ultrasounds (US), magnetic resonance imaging (MRI), or computed tomography (CT) reports	The authors describe an iterative process that includes set phrases, ICD-9 codes, negation terms, and a few other rules.	Researchers derived the validation dataset for the study by conducting medical chart reviews of UCLA primary care patients with chronic liver disease, identified by a chronic liver disease ICD-9 code (571.XX). The gold standard for cirrhosis diagnosis involved Stage 4 cirrhosis on liver biopsy, cirrhotic-appearing liver on abdominal imaging (CT, US, or MRI), or clinical diagnosis based on comprehensive chart review. The validation dataset was reviewed by two authors (E.K.C. and C.Y.Y.) under the guidance of an experienced hepatologist (B.A.R.)
Liu H 2021 (84)	Individuals with liver cancer.	1089 patients, 480 diagnosed with liver cancer. The patients were from a tertiary hospital in Beijing, China, between 2012 and 2019.	Demographic information is not given.	Liver cancer diagnoses in China, explicitly utilising radiology reports.	The study states 480 radiology reports with liver cancer and 609 without	Radiology reports	The study utilised the BERT (Bidirectional Encoder Representations from Transformers) NLP tool to extract evidence from Chinese radiology reports for computer-aided liver cancer diagnosis. Additionally, named-entity recognition (NER) was employed using BERT to extract evidence from the reports. The study also utilised a BERT-BiLSTM-CRF model for identifying specific phrases in radiology reports and a fine-tuned BERT language model with BiLSTM-CRF for evidence extraction.	Data was randomly split 8:2. The whole pipeline was based on a lexicon constructed manually according to Chinese grammatical characteristics. A few reports were sampled randomly to generate the lexicon by manual reading.
Sada 2016 (85)	Patients with hepatocellular cancer (HCC) were identified through ICD-9 codes from Veterans Affairs administrative data between 2005-2010.	1,138 patients, with 773 individuals having hepatocellular cancer (HCC) and 365 individuals without HCC, were identified through ICD-9 codes from Veterans Affairs administrative	For age among the HCC patients, the percentages are <55 (15%), 55-64 (47%), 65-74 (18%), ≥75 (20%). Gender distribution is Male (99%) and Female (1%). Race distribution is Black (25%), White (72%), Other (3%). Ethnicity breakdown	Hepatocellular cancer (HCC) within the US Department of Veterans Affairs medical care system, utilising administrative data and electronic health records for case-finding algorithms	The number of documents is not given.	Radiology reports, pathology reports, and clinician notes from the electronic health record.	Automated Retrieval Console (ARC) is NLP-based software that combines features derived from NLP pipelines with supervised machine learning classification algorithms. ARC creates an algorithm to classify documents based on specific NLP features like noun phrases, verb phrases, or negating words. The Clinical Text Analysis and Knowledge Extraction System (cTAKES)	HCC diagnoses were verified by manually reviewing provider notes, laboratory data, radiology reports, and pathology reports in the electronic health record. They also reviewed a random sample of 612 patients with ICD-9 codes for cirrhosis (571.2, 571.5, 571.6) and without HCC codes to calculate the sensitivity of HCC codes. For this cirrhosis comparator group, they performed a manual chart review and identified 40 cases of HCC during the study period. NLP algorithms were developed using a separate 70%/30% split validation method for pathology

		data between 2005-2010.	is Hispanic (16%), Not Hispanic (84%), and Unknown (1%).	validated through natural language processing.			system was also used. Little detail is given about ARC.	and radiology reports. Documents were randomly divided into a 70% training set for ARC to generate algorithms and a 30% testing set to validate algorithms. Manual classification of radiology reports was based on classic imaging features of arterial enhancement or contrast wash-out on delayed phases, lesion size, clinical history, and final assessment reported by the radiologist. Disagreement was resolved by a third physician (HES).
Wang T 2022 (86)	Individuals with Hepatocellular Carcinoma (HCC).	The number of patients is not given.	Demographic information is not given.	Hepatocellular carcinoma diagnosis using NLP applied to imaging reports	1,140 imaging reports, with 655 reports at Site 1 and 485 at Site 2. This project used the NIHR HIC Viral Hepatitis theme in the UK.	Imaging reports from ultrasound, CT, and MRI examinations.	negSpacy (Python) for negation detection, Stanza for syntactic analysis and named entity recognition (NER) in biomedical domains. NLTK and spaCy (Python) were also used for natural language processing tasks such as text cleaning, pre-processing, and data extraction from imaging reports. Word2Vec was used for text representation, and pre-trained DL models like BERT and ClinicalBERT were fine-tuned for evaluation. An accessible code link was provided with the original paper: https://github.com/tinaty/NLPHCC - although this appears to have now been taken down.	Labels were annotated by medical students in each site. Reports were initially manually annotated as binary classes (HCC vs. non-HCC). Imaging reports were manually labelled (i.e., assigning a binary label ('yes' or 'no') to an imaging report to indicate whether there is HCC presence in that imaging examination). We then selected imaging reports that have been manually labelled to develop and validate a rule-based method. To further verify the correctness of our proposed algorithm on non-HCC cases in the test set, 47 reports (10%) of the original 468 non-HCC reports were randomly selected, for which ground truth labels were manually checked. They found that our proposed NLP algorithm accurately identified all these 47 reports.
Pancreas								
Kooragayala 2022 (89)	Trauma centre patients aged over 18 who were admitted between 2010 and 2020 and underwent abdominal-based CT imaging within 24 hours of arrival	18,769 adult trauma centre patients were admitted.	The study reports demographic values such as age, body mass index (BMI), gender, and ethnicity. The mean age of patients with positive findings was 70.47 years (SD 18.456), and the	Abdominal-based CT imaging within 24 hours of arrival to the emergency department.	The validation set consisted of 400 CT scan reports, and the positive test set included 28 patients who underwent pancreatic surgery.	CT scan reports, electronic medical records, and radiology reports were used in this study.	The study utilised an open-source NLP software for querying pancreatic lesions, requiring manual configuration for optimal effectiveness.	The validation set of 400 CT scan reports, each of which was classified manually for the presence or absence of a pancreatic lesion of interest, but further details were not given.

	to the emergency department.		mean BMI was 26.447 kg/m2 (SD 6.075), with a higher proportion of females (55.2%) than males (44.8%) having positive findings. White/Caucasian patients had the highest frequency within the study population, comprising 78% of the patients with positive findings. Ethnicity distribution included White/Caucasian (78%), African American (12.5%), Hispanic (4.7%), and Asian (1.3%).					
Roch 2015 (87)	Patients with pancreatic cysts, particularly mucinous cysts, which are established precancerous lesions with the potential to develop into invasive adenocarcinoma.	50669 Patients	Demographic information is not given	A hospital setting for early detection of pancreatic cancer, with a system implemented on the hospital server for real-time tracking and daily execution.	566,233 reports from 50,669 unique patients over a 7-month period.	Longitudinal electronic medical records (EMR) containing clinical, radiological, surgical, and pathological narrative reports from various healthcare organisations in Indiana.	Unstructured Information Management Architecture (UIMA) framework. A rule-based algorithm was created to identify specific keywords related to pancreatic cysts in the free text of EMRs.	Manual analysis of clinical reports was also performed to determine commonly used 'pancreatic cyst' descriptors. The final assembled list of 'pancreatic cyst' concepts was used in the NLP software to identify patents with a pancreatic cyst. The extraction process was first performed on a training set (obtained after randomization). Physician experts in pancreatology performed the validation manually, ensuring the algorithm's performance met target precision and recall levels before applying it to the test set.
Xie 2020 (90)	Patients with an initial diagnosis of chronic pancreatitis (CP) at Kaiser Permanente Southern California (KPSC)	3,672 patients, with 1,330 patients having advanced chronic pancreatitis and 2,342 patients having non-	Advanced Chronic Pancreatitis (N = 1,330). Age at CP diagnosis: Mean 62.8, Female: 588 (44.2%), White: 757 (56.9%). Not advanced	Patients with chronic pancreatitis (CP) at Kaiser Permanente Southern California (KPSC) between	Pool of 58,085 radiology studies.	Radiology imaging reports	Negex, ContextNLP, NLTK (Python), and Stanford Core NLP for algorithm development. These tools were integrated into an internally developed NLP platform on a Linux server.	A sample of 100 CP patients (20 for each imaging feature) was randomly selected from the study cohort. Their corresponding radiology imaging reports<br>(total = 1,253) were manually reviewed by the clinical study team to determine the presence of each of the individual pancreatitis-related

	between January 2006 and December 2015. The patient population included those with an initial CP diagnosis, excluding individuals with pancreatic cancer before CP diagnosis, CP diagnosis before 2006, and less than one year of continuous health plan enrollment. Patients were required to have at least one pancreas-related image (abdominal ultrasound, computed tomography CT, or magnetic resonance imaging MRI).	advanced acute pancreatitis.	Pancreatitis (N = 2,342). Mean 53.7, Female: 1206 (51.5%), White: 1056 (45.1%). Income, BMI, prior smoking and alcohol history were also given.	January 2006 and December 2015. Specifically, the study aimed to identify a subset of patients with advanced chronic pancreatitis based on radiographic findings and clinical presentation.				imaging findings. The results were used for initial algorithm development for each imaging feature. An additional set of 500 imaging reports from a separate sample of 450 patients were then randomly selected from the remaining imaging reports and reviewed manually by the study radiologists. The results of the manual review of these 500 imaging reports were used to provide further refining of the rule-based computer generated. A total of 500 randomly selected imaging reports from another sample of 453 patients were used to generate the reference standard for validation. The study radiologists fully reviewed all reports to identify the study's five imaging features algorithms.
Yamashita 2022 (88)	Population that underwent CT and MRI examinations for pancreatic lesions.	199,783 unique patients.	Median age of 70.0 years, with 57.5% being female and 42.5% male.	Identifying and measuring pancreatic cystic lesions from free-text radiology reports using natural language processing (NLP) techniques.	430,426 free-text radiology reports from 199,783 unique patients, with 15,621 reports (3.63%) from 8,504 patients (4.26%) identified as positive for pancreatic cystic lesions.	Free-text radiology reports, including CT reports and MRI reports.	A BioBERT-based question-answering model, the CheXpert-labeler implementation, and measurement extraction code are available on GitHub at this URL: https://github.com/stanfordmlgroup/chexpert-labeler	The labeller was set up in three stages: mention extraction, classification, and aggregation. First, the labeller extracted mentions of observations from the free-text radiology reports. A list of phrases for mention extraction was curated by a radiologist (R.Y.) with 12 years of experience in body imaging by reviewing 204 randomly sampled reports that were subsequently excluded from the rest of the analysis. Second, the extracted mentions of observations were classified as negative (e.g., “no evidence of pancreatic cystic lesion”), uncertain (e.g., “low-attenuation may represent small pancreatic cystic lesion”), or positive (e.g., “hypodensity representing small pancreatic cystic lesion”).

								Finally, they used the classification for mentions of observations to arrive at a final label for the presence or absence of a PCL. The model's performance for identifying reports with PCLs was assessed through a reader study using 1000 reports from 1000 patients selected via stratified random sampling. They recruited eight radiologists (K.B., P.Y.C.C., J.H.D., M.N.F., D.G., L.N.M., A.S., A.L.W.)<br>with 3–10 years of experience each, and each radiologist independently annotated 250 reports as to the presence or absence of a PCL.
