# Supplementary material for: A foundation systematic review of natural language processing applied to gastroenterology & hepatology
Source: BMC Gastroenterol. 2025 Feb 6;25:58. doi: 10.1186/s12876-025-03608-5 (PMC11800601; doi:10.1186/s12876-025-03608-5)
Supplement: Supplementary file 8 — Supplementary Material 8. [file 12876_2025_3608_MOESM8_ESM.pdf]

Supplemental File 8: Included Data & Outcomes

Table H. Included Data & Outcomes			
**Authors**	**Study Purpose**	**Performance of NLP Algorithm**	**Study Outcomes**
Colonoscopy			
Harrington 2018 (61)	The study aimed to develop machine learning models to predict colorectal polyp recurrence risk, personalising colorectal cancer screening follow-up plans by tailoring surveillance regimens to patients' risk levels. It also aimed to utilise these models to optimise colonoscopy follow-up plans, potentially reducing unnecessary screenings, healthcare costs, and psychological stress associated with colorectal cancer screening programs.	The logistic elastic net model had an AUC of 0.65 (Â±0.04), the gradient boosting model had an AUC of 0.63 (Â±0.04), the random forest model had an AUC of 0.62 (Â±0.04), the lasso logistic model had an AUC of 0.62 (Â±0.04), the SVM radial model has an AUC of 0.55 (Â±0.04). The weighted KNN model had an AUC of 0.52 (Â±0.04).	The best-performing model achieved an AUC performance between 0.63 and 0.65, with all models except for the k-nearest neighbours model displaying significantly superior performance to random chance. These models aimed to personalise colorectal cancer screening follow-up plans by tailoring surveillance regimens to patients' risk levels, potentially reducing unnecessary screening and associated healthcare costs.
Gourevitch 2018 (46)	To investigate the variation in pathologist classification of colorectal adenomas and serrated polyps using clinical data from a large multi-site sample of pathologists, focusing on the impact on endoscopists' quality scores, particularly regarding serrated polyp detection rates.	The NLP program demonstrated high accuracy in identifying both adenomas and serrated polyps, with an accuracy rate exceeding 95% for both outcomes.	Pathologists demonstrated high variation, particularly in the classification of sessile serrated lesions.
Wadia 2017 (62)	The study aimed to develop a clinical decision support system for monitoring post-colonoscopy patient follow-up and scheduling. The system aimed to improve patient care coordination and triaging based on pathology findings.	The primary performance outcomes for the NLP algorithm in this study included achieving a recall of 100% for detecting lower GI cases with a specificity of 98.5%, a precision of 95.2%, and an F-1 score of 0.975. When predicting "actionable" versus lower GI and other reports, the recall and specificity were 98.9% and 98.6%, respectively, with a precision of 92.8% and an F-1 score of 0.958.	The successful development of a clinical decision support system for monitoring post-colonoscopy patient follow-up and scheduling.
Hoogendoorn 2016 (36)	To utilise uncoded consultation notes from electronic medical records for predictive modelling of colorectal cancer in a primary care dataset of 90,000 patients in Utrecht, the Netherlands.	The AUC values ranged from 0.831 to 0.900 for various approaches, including Bag of Words, Topic modelling with oversampling, Separate topic modelling for two classes, Topic modelling for text beyond consultation code, Coding using ICPC descriptions, and Coding using UMLS.	Predictive modelling of colorectal cancer based on the analysis of uncoded consultation notes within a specific primary care population in Holland.
Syed 2022 (51)	The study's primary purpose was to develop a hybrid Artificial Neural Network (h-ANN) model that utilises combined BERT and FLAIR embeddings for clinical concept extraction from colonoscopy, pathology, and radiology reports to enhance word representations and improve clinical NLP systems. The aim was to compile comprehensive colonoscopy concepts to aid in	F1 Scores: Pathology (h-ANNpath): 89.66 – 92.25. Mean: 91.03 ± 1.53. Colonoscopy (h-ANNcol): 89.42 – 91.76. Mean: 90.60 ± 1.19. Radiology (h-ANNrad): 85.91 – 88.55. Mean: 87.22 ± 1.45	Development of a hybrid Artificial Neural Network (h-ANN) model utilising combined BERT and FLAIR embeddings for clinical concept extraction from colonoscopy, pathology, and radiology reports. The model achieved higher scores for identifying entities in reports using concatenated embeddings than individual embeddings, with the most significant improvement seen in radiology concept extraction.

	colonoscopy quality improvement and colorectal cancer research.		
Karwa 2020 (63)	To develop an automated algorithm for generating guideline-based recommendations for follow-up colonoscopies utilising a hybrid NLP tool.	The NLP was reported to be 100% accurate and in complete concordance with the guideline recommendations. The agreement between the CDS algorithm and guidelines is reported to have a Cohen's k of 0.972.	Development of an automated algorithm generating accurate guideline-based recommendations for follow-up colonoscopies.
Li 2021 (48)	To develop and validate Natural Language Processing (NLP) tools for accurately identifying colorectal cancer mismatch repair status in Lynch syndrome screening using pathology reports.	100% positive predictive value for the expression of all four mismatch repair proteins and 100% sensitivity and PPV in mismatch repair-deficient colorectal cancers.	The successful development and validation of Natural Language Processing (NLP) tools for accurately identifying colorectal cancer mismatch repair status in Lynch syndrome screening. Additionally, the NLP tool accurately determined Lynch syndrome likelihood and eligibility for germline analysis based on mismatch repair immunohistochemistry results, BRAF V600E mutation, and MLH1 promoter methylation.
Vithayathil 2022 (52)	To develop a large colonoscopy-based longitudinal cohort for integrated research of colorectal cancer, focusing on endoscopic early detection and removal of pre-cancerous polyps to prevent cancer progression, stratify future cancer risk, gather detailed procedural, histopathological, molecular, and genetic data, and enhance understanding of the natural history of colorectal cancer (CRC) for improved screening and post-polypectomy strategies.	A sensitivity of 99.4% for extracting polyp histology, location, subtype, and dysplasia from endoscopy and pathology reports. The validation study demonstrated a PPV of 100% and NPV of 99.3% for polyp histology and a PPV of 100% and NPV of 99.6% for colorectal adenoma subtype identification.	The development of a large colonoscopy-based longitudinal cohort for integrated research of colorectal cancer. This cohort focused on endoscopic early detection and removal of pre-cancerous polyps to prevent cancer progression, gather detailed procedural, histopathological, molecular, and genetic data, and enhance understanding of the natural history of colorectal cancer for improved screening and post-polypectomy strategies.
Nayor 2018 (53)	The study aimed to develop and validate an accurate automated process using NLP to calculate Adenomas Detection Rates (ADRs) and Sessile Serrated Polyp Detection Rates (SDRs) in colonoscopy and pathology reports. It aimed to achieve 100% accuracy in identifying adenomas and SSPs to enable efficient quality improvement and research in colonoscopy procedures.	Precisions of 1 for all variables, recall of 1 for all except colonic location (0.98)	Development and validation of an automated process using natural language processing (NLP) to calculate Adenoma Detection Rates (ADRs) and Sessile Serrated Polyp Detection Rates (SDRs) in colonoscopy and pathology reports.
Parthasarathy 2020 (59)	To develop a natural language processing (NLP) tool to automatically extract and combine colonoscopy and pathology data to diagnose Serrated Polyposis Syndrome (SPS) with high accuracy, aiming to improve detection rates compared to clinician diagnosis.	Accuracy: 93%	Development of a tool that could automatically extract and combine colonoscopy and pathology data to accurately diagnose Serrated Polyposis Syndrome (SPS) within a health system.
Laique 2021 (54)	The study applied optical character recognition (OCR) and natural language processing (NLP) techniques to extract clinical information from colonoscopy reports, pathology reports, and electronic health records (EHR). It aimed to	Accuracy: Polyp - 564/589 (95.8%), Polyp location (Distal) - 571/589 (96.9%), Polyp pathology (Adenoma) - 580/589 (98.5%). Precision: Polyp - 243/246 (98.8%), Polyp location (Distal) - 155/156 (99.4%), Polyp pathology (Adenoma) -	Optical character recognition (OCR) and natural language processing (NLP) techniques were applied to extract clinical information from colonoscopy reports, pathology reports, and electronic health

	assess the validity and accuracy of NLP tools to extract variables such as examination indication, examination extent, bowel preparation quality, and other vital parameters from these documents.	136/141 (96.5%). Sensitivity: Polyp - 243/265 (91.7%), Polyp location (Distal) - 155/172 (90.1%), Polyp pathology (Adenoma) - 136/14 (97.1%).	records (EHR). Notable accuracy rates were achieved for detecting advanced adenomas, inadequate bowel preparation, failed cecal intubation, high-grade dysplasia, polyps, and adenomas.
Peterson 2021 (39)	To develop an automated system for identifying and assigning colonoscopy surveillance recommendations for individuals with colorectal polyps. The system aims to improve compliance with colorectal cancer surveillance guidelines, enhance colorectal cancer prevention and screening outcomes, reduce workload, ensure timely follow-up, and improve quality through data-driven programs.	Accuracy (>84%) for polyp risk stratification, guideline-concordant surveillance recommendations (>90%), and overall accuracy of 92% for assigning recommended surveillance intervals for colonoscopy. The algorithm demonstrated precision (PPV) of 92% and sensitivity of 95% for extracting targeted polyp properties from colonoscopy reports, and for pathology report extraction, the average PPV was 95% and sensitivity was 97%.	Development of an automated system utilising natural language processing (NLP) to identify and assign colonoscopy surveillance recommendations for individuals with colorectal polyps.
Tinmouth 2023 (55)	To develop and validate a natural language processing (NLP) algorithm to identify colorectal adenomas in pathology reports and measure adenoma detection rates at the population level in Ontario, Canada. The study aimed to assess the completeness of pathology data from lower-bowel endoscopies, develop and validate an NLP algorithm to identify adenomas and measure the adenoma detection rate for all endoscopists in Ontario using health administrative databases.	All extracted values had an F1 score > 96 in the validation set aside from 'Adenomas not otherwise specified' where the F1 Score was 0.74	Developing and validating an NLP algorithm to identify colorectal adenomas in pathology reports.
Redd 2022 (58)	The primary purpose of the study was to identify colorectal cancer in US military Veterans aged 35-49 using structured and free-text clinical data.	(Recall, Specificity, Accuracy, AUC-ROC)<br>Logistic regression: 0.757, 0.891, 0.869, 0.875<br>Support vector machine: 0.884, 0.995, 0.976, 0.939<br>Random forest: 0.662, 0.998, 0.942, 0.975<br>Deep neural network: 0.911, 0.997, 0.982, 0.965	Successful identification of colorectal cancer in US military Veterans aged 35-49 using structured and free text clinical data.
Blumenthal 2015 (47)	The study aimed to predict non-adherence with outpatient colonoscopies using a novel electronic tool that measures prior non-adherence. It aimed to develop a non-adherence model incorporating variables such as education level, gender, history of psychiatric illness, Non-Adherence Ratio (NAR), wait time, and number of prior missed endoscopies to predict colonoscopy non-adherence.	The validation cohort had an AUC of 70.2%, with specific sensitivity and specificity values at a Non-Adherence Score of 0.46.	Development of a non-adherence model to predict non-adherence with outpatient colonoscopies. The model incorporated variables such as education level, gender, history of psychiatric illness, Non-Adherence Ratio (NAR), wait time, and the number of prior missed endoscopies.
Lee 2019 (56)	The study aimed to accurately identify colonoscopy quality and polyp findings using NLP tools and extract exam quality-related and large polyp information from de-identified colonoscopy reports.	Compared to manual review, sensitivity for screening indication was 100% (95% confidence interval: 95.3%–100%), PPV was 90.6% (82.3%–95.8%), and accuracy was 98.2% (97.0%–99.4%). For cecal intubation, sensitivity was 99.6% (98.0%–100%), PPV was 100% (98.5%–100%), and accuracy was 99.8% (99.5%–100%).	The study's primary outcome was the accurate identification of colonoscopy quality and polyp findings using natural language processing (NLP) tools.

Fevrier 2020 (37)	The study's primary purpose was to develop and validate NLP tools to extract critical variables related to colonoscopy quality from free-text reports, explicitly focusing on variables such as polyp type, location, size, colonoscopy extent, and quality of preparation. The study aimed to create a method that could be evaluated and adapted for other healthcare organisations, focusing on improving the reporting of specific polyp subtypes.	PPV's ranged from 62-100%. Recall: 95-100%.	Development and validation of a Natural Language Processing (NLP) tool that accurately extracted critical variables related to colonoscopy quality from free-text reports. These variables included polyp type, location, size, colonoscopy extent, and quality of preparation.
Shi 2022 (49)	The study aimed to automate the identification of high-risk patients for genetic testing. Specifically, it aimed to extract information from free-text family health history comments to enhance patient identification for genetic testing eligibility based on NCCN criteria. It focused on high-risk patients for genetic testing of hereditary cancers such as breast and colorectal cancer.	Precision, Recall and F1 Score were all 0.94 for detecting cancers in family history. However, performance for detecting a syndrome was much poorer: Precision: 0.67, Recall: 0.40, F1Score: 0.50, although these instances were far fewer in the dataset (n=6).	Attempted automatic identification of high-risk patients for genetic testing using natural language processing (NLP) tools on family health history data from electronic health records.
Bae 2022 (57)	To develop and evaluate an NLP pipeline for assessing quality indicators in free-text colonoscopy and pathology reports, focusing on polyp detection and surveillance interval measurement using a 10-year set of colonoscopy reports.	An overall accuracy of 0.99-1.00 for identifying polyp subtypes, 0.99-1.00 for identifying the anatomical location of polyps, and 0.98 for counting the number of neoplastic polyps. The NLP pipeline demonstrated performance similar to clinical experts in assessing adenoma detection rate (ADR), sessile serrated lesion detection rate (SDR), and surveillance intervals.	Development and evaluation of a natural language processing (NLP) pipeline for assessing quality indicators in free-text colonoscopy and pathology reports. The NLP algorithm achieved high accuracy rates for identifying polyp subtypes, anatomical locations of polyps, and counting neoplastic polyps, demonstrating performance comparable to clinical experts in assessing adenoma detection rate, sessile serrated lesion detection rate, and surveillance intervals.
Patterson 2015 (50)	Classify the indications for colonoscopy procedures using NLP techniques to determine their appropriateness based on documented indications in colonoscopy reports.	The rule-based system performed best in predicting colonoscopy indications, with a Precision of 0.861, NPV of 0.974, Sensitivity of 0.885, and Specificity of 0.968.	Classification of colonoscopy indications using NLP. The study compared a rule-based NLP system to machine learning models, with the rule-based system demonstrating better accuracy and sensitivity in classifying colonoscopy indications.
Ternois 2018 (60)	To automatically develop an automatic coding system for digestive endoscopies using natural language processing (NLP) tools and classification algorithms to automatically attribute CCAM codes to endoscopy reports. This would simplify physicians' manual coding tasks and correlate medical report content with CCAM codes for billing and research purposes.	The reports were coded with an average precision and recall of 0.92 on the 1639 text corpus.	Developing an automatic coding system for digestive endoscopies using NLP tools and classification algorithms. The system successfully attributed CCAM codes to new endoscopy reports based on the algorithm's performance, simplifying manual coding tasks by physicians and correlating medical report content with CCAM codes for billing and research purposes.
ERCP & Sedation			

Imler 2018 (64)	To evaluate provider-specific quality measures for Endoscopic Retrograde Cholangiopancreatography (ERCP) using Natural Language Processing (NLP) techniques, procedural volumes and quality metrics were calculated across providers to determine variations in quality of care.	Precision for the 13 quality measures ranging from 84-100% and accuracy ranging from 90-100%	Evaluation of provider-specific quality measures for Endoscopic Retrograde Cholangiopancreatography (ERCP) procedures using Natural Language Processing (NLP) techniques to try and improve care.
Shen 2021 (33)	To develop and implement a clinical decision support system (CDSS) using expert consensus-derived logic and NLP to reduce sedation-type order errors for patients undergoing endoscopy procedures.	There was a significant decrease in the primary outcome of sedation-type order error rate on the day of endoscopy between the pre-pilot (0.39%) and pilot (0.037%) groups with an odds ratio of 0.094, Fisher's exact P value < 1e–8. For the pilot, 3.5 patients were manually reviewed for every intercepted patient. During the pilot, 172 of the 16 321 total cases (1.1%) were identified as containing potential sedation errors by the CDSS. Forty-nine of these cases were determined to be true positives and intercepted. Six cases of sedation error were not detected during the pilot (false negatives) and were discovered on the day of the procedure. The CDSS had a precision of 28.5%, negative predictive value of 99.9%, recall/sensitivity of 89.1%, and specificity of 99.2%.	Reduction of sedation-type order errors for patients undergoing endoscopy procedures through developing and implementing a clinical decision support system (CDSS) using expert consensus-derived logic and NLP.
Gastrointestinal Bleeding			
Shung 2021 (40)	To identify patients with acute gastrointestinal bleeding (GIB) early using natural language processing (NLP) and decision rules, aiming to develop and validate risk stratification models within electronic health records (EHRs) for accurate identification of acute GIB at admission.	SNOMED codes (Reference): PPV 29%, Sensitivity 71%, Specificity 46%. Decision rule 2 modified: upper GI bleed-specific triage terms + ROS fields: PPV 82%, Sensitivity 34%, Specificity 98%. Syntax-based NLP with random forest: PPV 85%, Sensitivity 46%, Specificity 98%. BERT neural network NLP: PPV 78%, Sensitivity 72%, Specificity 94%.	Development and validation of risk stratification models within electronic health records (EHRs) to accurately identify acute gastrointestinal bleeding (GIB) at admission.
Taggart 2018 (65)	The study aimed to assess the effectiveness of various models (Support Vector Machine (SVM) and Extra Trees (ET)) in detecting bleeding events in clinical notes, with a focus on sensitivity due to the clinical significance of these events.	The rules-based model had the best overall performance: 84.6% specificity, 62.7% positive predictive value, and 97.1% negative predictive value.	The rule-based natural language processing approach outperformed machine learning models in identifying bleeding events among critically ill patients.
Gastroscopy			
McVay 2018 (68)	Accurately identifying dysphagia indications for Oesophagogastroduodenoscopy (OGD/EGD) procedures using natural language processing (NLP) with implications for classifying overuse and quality measurement.	The primary performance outcomes for the NLP algorithm in this study were as follows:- Sensitivity: 84.7%- Specificity: 99.7%- Accuracy: 97.0%- Negative Predictive Value (NPV): 96.7%- Positive Predictive Value (PPV): 98.6%- F measure: 91.1%.	Successful identification of dysphagia indications for Oesophagogastroduodenoscopy (OGD/EGD) procedures using natural language processing (NLP)
NguyenWenker 2023(69)	To develop and validate a Natural Language Processing (NLP) algorithm using the Clinical Language Annotation, Modeling, and Processing (CLAMP) software to automatically identify dysplasia in pathology reports of patients with Barrett's Esophagus (BE) within the National Veterans Affairs (VA) system.	In the validation set of 230 patients with Barrett's Esophagus, recall was 92.3%, precision was 100%, accuracy was 98.7%, and F-measure was 96.0%.	Development and validation of a Natural Language Processing (NLP) algorithm using the Clinical Language Annotation, Modeling, and Processing (CLAMP) software to automatically identify dysplasia in pathology reports of patients with Barrett's Esophagus (BE).

Ding 2020 (38)	The study's primary purpose was to predict gastric cancer based on gastroscopy reports through a PLWA-based homogeneous weighted ensemble method compared to other weighting methods.	LR model Specificity (%) 89.94, Sensitivity (%) 74.44<br>SVM model Specificity (%) 89.52, Sensitivity (%) 69.45<br>MLP model Specificity (%) 84.68, Sensitivity (%) 73.45<br>XGB model Specificity (%) 91.85, Sensitivity (%) 73.72	Enhancement of gastric cancer screening performance using ML
Song 2022 (67)	The study aimed to develop an NLP pipeline to extract key concepts from oesophagogastroduodenoscopy (OGD/EGD) reports and linked pathology reports for ten gastric diseases. The NLP pipeline aimed to extract information on the diseases' extent, location, stage, and size, evaluate its performance against a gold standard created by gastroenterologists, and demonstrate its utility in clinical research by analysing ten years of historical data to identify typical demographics of gastric diseases.	Precisions ranged between 0.80 and 1, with Sensitivities ranging between 0.75 and 1.	Development and evaluation of a natural language processing (NLP) pipeline for extracting critical concepts related to gastric diseases from oesophagogastroduodenoscopy (OGD/EGD) reports and linked pathology reports in English and Korean.
IBD			
Gomollón 2022 (75)	The study aimed to analyse clinical characteristics and predict Crohn's disease relapses by extracting insights from unstructured, free text captured in physicians' notes. Disease characteristics are based on the Montreal Classification. The study applies logistic regression, decision trees, and random forests to analyse predictors of Crohn's disease relapses.	Random Forest - 2 years of data. Accuracy: 0.83, Precision: 0.67, Recall: 0.71, F1-Score: 0.69 and AUC 0.91 for predicting relapse risk.	The development and validation of a Crohn's NLP algorithm. Key predictors identified by models included age, past relapses, complications, tobacco/substance abuse, cumulative relapses, obesity, abdominal imaging, intake of belladonna and its derivatives, past surgery, laboratory results, malabsorption-related variables and unexpected factors like respiratory infections.
Hou 2016 (76)	To develop and validate a Natural Language Processing (NLP) algorithm using the CLAMP tool to accurately identify colorectal dysplasia in patients with Inflammatory Bowel Disease (IBD) within a national cohort of Veterans.	Accuracy of 97.1% for detecting low-grade dysplasia, with a precision of 87%, recall of 96.6%, and an F-measure of 91.5%. For high-grade dysplasia, the accuracy was 97.7%, precision 36.6%, recall 96.2%, and F-measure 53.1%.	Development and validation of an NLP algorithm to accurately identify colorectal dysplasia in patients with Inflammatory Bowel Disease (IBD) within a national cohort of Veterans.
Stidham 2022 (70)	To develop a proof-of-concept natural language processing (NLP) pipeline to automatically detect mentions of extraintestinal manifestations (EIMs) and infer EIM activity or status using electronic clinical documents.	Accuracy of 94.1%, sensitivity of 0.92, specificity of 0.95, and agreement of κ = 0.76 when predicting general EIM status.	Development of an NLP pipeline for predicting general extraintestinal manifestations (EIM) status in patients with inflammatory bowel disease (IBD) using electronic clinical documents.
Walker 2016 (73)	The study aimed to develop a computer-assisted expert case definition using electronic health records (EHRs) to enhance the identification of acute liver dysfunction cases in individuals with inflammatory bowel disease (IBD). It also aimed to compare machine-implementable rules to expert adjudication and calculate performance statistics for the rule compared to expert adjudication.	Sensitivity of 92% and a positive predictive value of 79% when compared against clinical review in an independent test round.	Development of a computer-assisted expert case definition using electronic health records (EHRs) to enhance the identification of acute liver dysfunction cases in individuals with inflammatory bowel disease (IBD).
Zand 2020 (72)	The study aimed to create a high-quality knowledge base of human dialogues through the UCLA IBD platform to train an inflammatory bowel disease (IBD) chatbot using natural language processing (NLP). It aimed to categorise large amounts of electronic messaging data related to IBD into a reasonable number of categories for chatbot development and testing in a patient population with IBD.	In 95.0% (285/300) of cases, there were minor or no differences in categorisation between the algorithm and the three independent physicians.	The successful development and validation of a natural language processing (NLP) algorithm to categorise large datasets of electronic messages between patients with inflammatory bowel disease (IBD) and healthcare providers (HCPs). The NLP algorithm accurately identified keywords associated with ulcerative colitis and Crohn's disease in patient narrative texts, surpassing the accuracy of models relying on billing codes.

Montoto 2022 (74)	To assess the performance of Savanna's EHRead technology in a Spanish healthcare registry	Precision of 0.88, Recall of 0.98, and an F1 score of 0.93 for detecting Crohn's disease. For Crohn's disease flare, Precision was 0.91, Recall was 0.71, and F1 score was 0.80. Vedolizumab detection had a Precision of 0.86, Recall of 0.94, and F1 score of 0.90.	The study evaluates the EHRead technology's performance in identifying Crohn's disease-related variables in Spanish electronic health records. It highlights the potential of clinical natural language processing systems in extracting key patient-centred information from EHRs for improved disease management.
Kurowski 2022 (71)	To extract clinical information using NLP from the EPR to construct a computable phenotype of a Crohn's disease natural history model.	A PPV of 98.5% is described for the model.	Developing a computable phenotype for Crohn's disease using electronic medical records (EMRs) and health state utility assessments.
Liver			
Bell 2022 (34)	The study aimed to predict liver utilisation rate and post-transplant outcomes using Natural Language Processing (NLP) techniques on donor text narratives and clinical notes to improve the Donor Risk Index (DRI) performance in predicting liver utilisation and mortality. The study aimed to leverage unstructured clinical notes for more accurate predictions.	The NLP model achieved AUC scores of 0.78, 0.78, and 0.81 for liver utilisation prediction using logistic regression, random forest, and gradient boosting classifiers, respectively. The logistic regression model had the highest Cohen Kappa score of 0.43 and specificity of 0.62, indicating its effectiveness in identifying unutilised livers. The gradient boosting classifier had the highest sensitivity of 0.97 in identifying utilised livers.	The study's primary outcome measure was the liver utilisation rate, defined as the proportion of donated livers recovered from the deceased donor. 30-day and 1-year mortality rates were also examined. Particular phrases such as 'alcohol' and 'dcd' were found to be associated with unutilised livers using LIME, an explainability algorithm.
Heidemann 2017 (81)	To identify patients with idiosyncratic drug-induced liver injury (DILI) attributed to specific drugs using natural language processing (NLP) algorithms to improve search term specificity.	The NLP algorithm led to a fivefold increase in detecting DILI cases, improved search term specificity, reduced review workload, and decreased time needed to exclude non-DILI cases. PPV of 64% is reported for the four very high-yield search terms strategy with an NPV of 100% and Sensitivity of 53%	Natural language processing (NLP) algorithms significantly improved search efficiency, positive predictive value, and detection of idiosyncratic drug-induced liver injury (DILI) cases. This improvement was achieved by refining search terms and focusing on high-yield liver injury terms. This led to a fivefold increase in identifying DILI cases, reduced review workload, and decreased time needed to exclude non-DILI cases.
Redman 2017 (79)	Accurately identifying Fatty Liver Disease (FLD) using natural language processing algorithms applied to ultrasound, CT scan, and MRI reports from veterans' electronic medical records.	Sensitivities of 0.90-1, Precisions of 0.918-1 and F1 scores of 0.909-1 across all imaging modalities.	Development and validation of natural language processing (NLP) algorithms to accurately identify Non-Alcoholic Fatty Liver Disease (NAFLD) in radiology reports.
Wang X 2022 (82)	To develop and apply an AI-powered causal inference framework called DeepCausality to analyse free text data related to idiosyncratic drug-induced liver injury (iDILI) in patients, aiming to enhance causal inference capabilities in the clinical setting by leveraging natural language processing tools and integrating causal functions into existing models.	The test set's accuracy was 0.92, F1-score was 0.84, Precision was 0.86, and Recall was 0.82. Based on current knowledge, the NLP algorithm effectively identified causal terms related to idiosyncratic drug-induced liver injury (iDILI) with a high enrichment rate of 90%.	Successful development and application of the DeepCausality AI-powered causal inference framework to analyse free text data related to idiosyncratic drug-induced liver injury (iDILI) in patients. This framework effectively stratified iDILI patients based on various clinical parameters and demonstrated a high correlation with expert-based patient stratification, showcasing its effectiveness in classifying patients into severity groups.
Liu W 2022 (41)	To establish an ensemble learning classification model based on natural language processing (NLP) methods to classify radiological reports concerning liver lesion detection in patients with colorectal cancer (CRC) undergoing CT/MRI examinations of the upper abdomen. The study aimed to establish an ensemble learning classification model using NLP methods to improve the efficiency of clinicians' interpretations of these reports.	The ensemble learning classification model achieved 95.91% accuracy in the CT with/without contrast dataset using XGBoost. Other NLP tools, such as logistic regression, support vector machine and random forest, also demonstrated good classification accuracy, with rates of 95.89%, 95.04%, and 95.00%, respectively.	Developing an ensemble learning classification model based on natural language processing (NLP) methods to classify radiological reports concerning liver lesion detection in patients with colorectal cancer into five categories.

VanVleck 2019 (80)	To utilise augmented intelligence with natural language processing applied to electronic health records to identify patients with non-alcoholic fatty liver disease (NAFLD) and those at risk for disease progression in a clinical setting.	Precision (0.89), Recall (0.93), and F1 scores (0.91), with a low false positive rate (FPR) of 0.11. The NLP approach significantly outperformed the ICD and text-based approaches regarding precision, recall, and F1 scores. Combining the ICD results with the NLP findings increased sensitivity to 0.96 without reducing specificity (0.89). Additionally, the NLP algorithm demonstrated a sensitivity of 0.95, specificity of 0.97, positive predictive value of 0.90, and negative predictive value of 0.98 for identifying NAFLD patients at risk for disease progression.	Successful identification of patients with non-alcoholic fatty liver disease (NAFLD) and those at risk for disease progression within a large cohort.
Yim 2017 (35)	The study aimed to develop methods for accurately identifying and classifying attributes such as negation, temporality, and malignancy to enhance cohort identification, clinical research, and clinical decision support.	F1 score of 0.94, past temporality classification had an F1 score of 0.62, malignancy classification had an F1 score of 0.77, and incorporating attributes into full templates improved tumor-related event classification by 0.72 F1 over a baseline of 0.65 F1.	Development of an algorithm to identify and classify attributes such as negation, temporality, and malignancy in the radiology reports of HCC patients at Washington Medical Centre
Koola 2018 (77)	The study aimed to develop an automated phenotyping algorithm for hepatorenal syndrome (HRS) using a combination of structured data and natural language processing (NLP) variables to categorise hospitalisations into specific HRS types. By utilising population health data and comparing different phenotyping algorithms, the study aimed to refine diagnostic decision-making for HRS.	(AUCs) ranging from 0.73 to 0.93. The penalised logistic regression demonstrated the best discriminatory performance, with calibration modest for logistic regression but superior for gradient boosting and support vector machines. A priori variable selection performed similarly to dimensionality reduction using high-throughput phenotyping and semantic similarity-informed clustering, with an AUC of 0.81 – 0.82.	Developing an automated phenotyping algorithm for hepatorenal syndrome (HRS) using structured data and NLP variables. This study demonstrated improved phenotyping over ICD-9 coding.
Tariq 2022 (83)	To develop a malignancy prediction model for hepatocellular carcinoma (HCC) using natural language processing (NLP) tools on templated ultrasound (US) and magnetic resonance (MR) reports, focusing on language modelling, classification tasks, and text normalisation.	Calculated weighted F1 Scores of 0.95 and 0.93 for malignant and benign classifications using a US-finetuned BERT+Random Forest Classifier, compared different language models paired with classifiers, and visualised the model's inner workings through sensitivity analysis.	The development of a malignancy prediction model for hepatocellular carcinoma (HCC) using natural language processing (NLP) tools on templated ultrasound (US) and magnetic resonance (MR) reports. The study focused on enhancing language models' performance in HCC malignancy scoring, achieving weighted f1-scores of 0.95 and 0.93 for malignant and benign classifications using the US-finetuned BERT+Random Forest Classifier. Transfer learning techniques were leveraged to address challenges related to syntax variations in structured and unstructured documents.
Chang 2016 (78)	To develop an algorithm using NLP tools to automatically identify patients with cirrhosis within the UCLA primary care population. The aim is to improve healthcare for individuals with cirrhosis by accurately identifying them based on radiologist reports of abdominal imaging and chronic liver disease ICD-9 codes.	The primary performance outcomes for the NLP algorithm in this study were as follows:<br>- Sensitivity: 95.71%<br>- Specificity: 93.88%<br>- Positive Predictive Value (PPV): 91.78%<br>- Negative Predictive Value (NPV): 96.84%	The successful development of an algorithm using natural language processing (NLP) tools to automatically identify patients with cirrhosis within the UCLA primary care population.
Liu H 2021 (84)	To develop a computer-aided liver cancer diagnosis framework using a BERT-based deep learning method to extract evidence from Chinese radiology reports. The focus is on named-entity recognition (NER) and evidence extraction to aid in diagnosing liver cancer.	F1 scores of 89.14% and 82.19% at the character level and F1 scores of 98.40% and 90.67% at the report level for APHE and PDPH features, respectively, using the BERT-BiLSTM-CRF model. This model demonstrated superior performance compared to other models evaluated in the study.	The successful development of a computer-aided liver cancer diagnosis framework using a BERT-based deep learning method. The framework achieved high F1 scores of 98.40% and 90.67% for identifying APHE and PDPH features in Chinese radiology reports, respectively.
Sada 2016 (85)	The study aimed to validate case-finding algorithms for hepatocellular cancer (HCC) using NLP and assess their accuracy in identifying HCC cases from clinical and administrative documents.	The HCC ICD-9 code algorithm had a PPV of 0.67, a sensitivity of 0.95, and a specificity of 0.93. The pathology ARC algorithm had a PPV of 0.96, sensitivity of 0.96, and specificity of 0.97. The radiology	The study achieved a moderate positive predictive value, high sensitivity, and specific performance metrics for its

		ARC algorithm had a PPV of 0.75, sensitivity of 0.94, and specificity of 0.68.	algorithm in classifying pathology and radiology documents to identify HCC cases.
Wang T 2022 (86)	To identify Hepatocellular Carcinoma (HCC) from imaging reports using NLP.	HAN with word2vec obtained a higher sensitivity (75%) than other supervised learning models; however, it is still significantly inferior to the rule-based method (100%). Although the pre-trained transformer models can achieve comparable specificity (97.67% to 99.79%), they had low sensitivity (33.33% to 58.33%) due to the small sample size of data for the class of HCC. The rule-based method was capable of handling skewed data distributions by achieving a much higher F1 score and prevision (F1 score = 0.9231; Precision = 99.59%) compared to supervised learning models regardless of the model architecture and feature representation method (F1 score = 0.11 to 0.47; Precision = 6.3% to 80.0%).	Identifying Hepatocellular Carcinoma (HCC) from imaging reports using NLP techniques.
Pancreas			
Kooragayala 2022 (89)	To utilise natural language processing (NLP) software to identify pancreatic lesions in trauma centre patients who underwent abdominal-based CT imaging within 24 hours of arrival to the emergency department. The study aimed to improve early identification of these lesions to enhance treatment and follow-up, ultimately impacting 5-year survival rates.	Sensitivity: 0.33, Specificity: 0.99, F1 score: 0.29	The NLP algorithm demonstrated some effectiveness in the early identification of these lesions, with the most frequently identified lesions being IPMNs, traumatic findings, pancreatitis, pancreatic cysts, concerning lesions or masses, and ductal abnormalities.
Roch 2015 (87)	The study focused on identifying patients with pancreatic cysts, particularly mucinous cysts. It aimed to detect patients with premalignant cysts for improved pancreatic cancer screening and early detection, emphasising close monitoring for clinically relevant changes to prevent and detect pancreatic cancer early.	The algorithm achieved a mean sensitivity of 99.9% and specificity of 98.8%. It demonstrated high sensitivity and specificity in identifying pancreatic cysts and ductal dilation, with sensitivity ranging from 98.98% to 100% and specificity ranging from 96.3% to 100%.	Development of an automated pancreatic cyst screening system using NLP. This system demonstrated accuracy in detecting patients with pancreatic cysts, particularly mucinous cysts, aiding in the early detection of pancreatic cancer within a hospital setting.
Xie 2020 (90)	Leveraging NLP technologies to systematically assess patients diagnosed with chronic pancreatitis in a diverse, integrated community-based healthcare system.	For individual features, NLP yielded a sensitivity of 88.7% to 95.3% and a specificity of 98.2% to 100.0%.	Developed an algorithm to automatically sub-classify chronic pancreatitis images.
Yamashita 2022 (88)	To automate the identification and measurement extraction of pancreatic cystic lesions from free-text radiology reports using natural language processing (NLP) tools. The study aimed to develop an NLP-based system to identify patients with pancreatic cystic lesions, extract the most extensive measurements of the cysts, and address limitations such as measurement variability, reliability of cyst growth assessment, integration of imaging data, and validation of specific PCL types, aligning with American College of Radiology (ACR) guidelines, and analysing growth patterns of these lesions over time.	The interobserver agreement between the model and two radiologists was almost perfect (Fleiss k = 0.951), and the false-positive rate and true-positive rate were 3.0% and 98.2%, respectively, against the consensus of radiologists' annotations as ground truths. The overall accuracy and Lin concordance correlation coefficient for measurement extraction were 0.958 and 0.874, respectively, against radiologists' annotations as ground truths.	The development of an NLP-based system for identifying patients with pancreatic cystic lesions and extracting the most extensive measurements of the cysts.
