# Supplementary material for: A foundation systematic review of natural language processing applied to gastroenterology & hepatology
Source: BMC Gastroenterol. 2025 Feb 6;25:58. doi: 10.1186/s12876-025-03608-5 (PMC11800601; doi:10.1186/s12876-025-03608-5)
Supplement: Supplementary file 9 — Supplementary Material 9. [file 12876_2025_3608_MOESM9_ESM.pdf]

Supplemental File 9: Excluded Full Texts

Table I. Full Text Screening Exclusions

Title	Authors	Year	Journal	Vol	Issue	Pages	DOI	Study	Notes
Automated tracking of emergency department abdominal CT findings during the COVID-19 pandemic using natural language processing.	Li, Matthew D; Wood, Peter A; Alkasab, Tarik K; Lev, Michael H; Kalpathy-Cramer, Jayashree; Succi, Marc D	2021	Am J Emerg Med	49	aa2, 8309942	52-57	10.1016/j.ajem.2021.05.057	Li 2021	Exclusion reason: Wrong indication ;
Development and validation of a risk prediction model for post-polypectomy colorectal cancer in 4 prospective US cohorts	Knudsen M.D.; Wang L.; Wang K.; Polychronidis G.; Berstad P.; Wu K.; He X.; Hang D.; Fang Z.; Ogino S.; Chan A.T.; Giovannucci E.L.; Wang M.; Song M.	2022	Cancer Research	82	23 Supplement 1		https://dx.doi.org/10.1158/1538-7445.CRC22-PR002	Knudsen 2022	Exclusion reason: Only Abstract Available;
MACHINE LEARNING APPLICATIONS FOR NAFLD DIAGNOSIS IN THE POPULATION	Allen A.M.; Zhang N.; Canning R.; Benson J.T.; Udompap P.; Dierkhising R.; Yin J.; Therneau T.M.	2022	Hepatology	76	Supplement 1	S860-S861	https://dx.doi.org/10.1002/hep.32697	Allen 2022	Exclusion reason: Only Abstract Available;
PROGRESSION RATES TO CIRRHOSIS AND HCC BY FIBROSIS STAGE IN NON-ALCOHOLIC FATTY LIVER DISEASE AS ESTIMATED BY	Sherman M.S.; Challa P.K.; Ott A.; Przybyszewski	2022	Hepatology	76	Supplement 1	S778	https://dx.doi.org/10.1002/hep.32697	Sherman 2022	Exclusion reason: Only

NATURAL LANGUAGE PROCESSING OF PATHOLOGY REPORTS	E.; Anya E.U.; Wilechansky R.M.; McGoldrick J.; Goessling W.; Khalili H.; Simon T.G.								Abstract Available;
USING ARTIFICIAL INTELLIGENCE AGENT DEEPSUGGEST TO IDENTIFY PATIENTS WITH RUMINATION SYNDROME	Sabella J.; Sezgin E.; Lu P.; Van Diest A.K.; Huang Y.; Bali N.; Vaz K.; Yacob D.; Di Lorenzo C.; Lee J.	2022	Journal of Pediatric Gastroenterology and Nutrition	75	Supplement 1	S328-S329		Sabella 2022	Exclusion reason: Only Abstract Available;
VALIDATION OF A NATURAL LANGUAGE PROCESSING ALGORITHM TO EXTRACT DATA FOR SYSTEM-LEVEL ADENOMA DETECTION RATE CALCULATION	Morgan D.; Chorneyko K.; Swain D.; Bowes B.; Lee V.; Tinmouth J.	2019	Journal of the Canadian Association of Gastroenterology	2	Supplement 2	409	https://dx.doi.org/10.1093/jcag/gwz006.208	Morgan 2019	Exclusion reason: Only Abstract Available;
NATURAL LANGUAGE PROCESSING AND NAMED ENTITY RECOGNITION IN INFLAMMATORY BOWEL DISEASE REFERRALS	Stammers M.; George M.; Regas C.; May G.; Rahmany S.; Davis C.; Borca F.; Knibbs W.; Chandrabalan V.V.; Gwiggner M.	2022	Gut	71	Supplement 1	A194-A195	https://dx.doi.org/10.1136/gutjnl-2022-BSG.370	Stammers 2022	Exclusion reason: Only Abstract Available;
DEVELOPMENT OF A COMPUTATIONAL MACHINE LEARNING ESOPHAGEAL ADENOCARCINOMA AND BARRETT'S ESOPHAGUS RISK PREDICTION MODEL FROM A	Iyer P.G.; Sachdeva K.; Codipilly D.C.; Leggett C.L.; Asfahan S.; Awasthi S.; Padhye C.V.; Anand P.;	2022	Gastroenterology	162	7 Supplement	S-127	https://dx.doi.org/10.1016/S0016-5085%2822%2960307-X	Iyer 2022	Exclusion reason: Only Abstract Available;

DEIDENTIFIED ELECTRONIC MEDICAL RECORD DATABASE	Kumar P.; Behera K.C.; Singh S.P.; Shukla S.; Bade S.; Barve R.; Thiagarajan V.; Mahto C.; Singh N.; Yadav S.								
VALIDATION OF A MACHINE LEARNING ALGORITHM TO MEASURE BOWEL PREPARATION QUALITY FOR SCREENING COLONOSCOPY IN A LARGE HEALTH SYSTEM	Yang J.O.; Galoosian A.; Peterson E.K.; Soroudi C.; Myint A.; Hsu W.; Maehara C.K.; Kang Y.; Naini B.V.; Muthusamy V.R.; Esrailian E.; May F.F.P.	2022	Gastroenterology	162	7 Supplement	S-128	https://dx.doi.org/10.1016/S0016-5085%2822%2960309-3	Yang 2022	Exclusion reason: Only Abstract Available;
NATURAL LANGUAGE PROCESSING ENABLES HIGHLY ACCURATE EXTRACTION OF DETAILED HISTOPATHOLOGIC FEATURES OF NONALCOHOLIC STEATOHEPATITIS AND FIBROSIS FROM PATHOLOGY REPORTS	Sherman M.; Challa P.K.; Ott A.T.; Przybyszewski E.M.; Anya E.U.; Wilechansky R.; McGoldrick J.; Goessling W.; Khalili H.; Simon T.	2022	Gastroenterology	162	7 Supplement	S-1203	https://dx.doi.org/10.1016/S0016-5085%2822%2963566-2	Sherman 2022	Exclusion reason: Only Abstract Available;
CONFIDENCE ESTIMATION IN CLINICAL DECISION SUPPORT SYSTEM FOR DETERMINATION OF COLONOSCOPY SURVEILLANCE INTERVAL	Bae J.H.; Shin Y.; Piao Y.; Lim J.H.; Kim S.	2022	Gastroenterology	162	7 Supplement	S-839	https://dx.doi.org/10.1016/S0016-5085%2822%2961981-4	Bae 2022	Exclusion reason: Only Abstract Available;

AN AUTOMATED ALGORITHM ACCURATELY IDENTIFIES CIRRHOSIS AMONG PATIENTS WITH NONALCOHOLIC FATTY LIVER DISEASE (NAFLD), INCLUDING THOSE WITHOUT INTERNATIONAL CLASSIFICATION OF DISEASES (ICD) CODES FOR CIRRHOSIS OR ELEVATED FIBROSIS-4 SCORES	Goyal T.; Song M.W.; Moran I.J.; Chen V.L.	2022	Gastroenterology	162	7 Supplement	S-1238	https://dx.doi.org/10.1016/S0016-5085%2822%2963653-9	Goyal 2022	Exclusion reason: Only Abstract Available;
AUTOMATED REPORTING OF MEDICATION USE HISTORY FOR INFLAMMATORY BOWEL DISEASE USING NATURAL LANGUAGE PROCESSING OF CLINICAL NOTES IS ACCURATE AND OUTPERFORMS ELECTRONIC ADMINISTRATIVE RECORDS	Zhao X.; Yu D.; Bishu S.; Rice M.D.; Vydiswaran V.G.V.; Stidham R.	2022	Gastroenterology	162	7 Supplement	S-256	https://dx.doi.org/10.1016/S0016-5085%2822%2960607-3	Zhao 2022	Exclusion reason: Only Abstract Available;
IMPLEMENTATION OF A MACHINE LEARNING ALGORITHM TO MEASURE ADENOMA DETECTION RATES IN A LARGE HEALTH SYSTEM	Galoosian A.; Peterson E.K.; Maehara C.K.; Yang J.O.; Soroudi C.; Myint A.; Naini B.V.; Kang Y.; Muthusamy V.R.; Esrailian E.; Hsu W.; May F.F.P.	2022	Gastroenterology	162	7 Supplement	S-861	https://dx.doi.org/10.1016/S0016-5085%2822%2962035-3	Galoosian 2022	Exclusion reason: Only Abstract Available;
No Incidentaloma Left Behind: The Utility of Natural Language Processing Software in the Identification of Worrisome Pancreatic Lesions in the Trauma Population	Kooragayala K.; Lou J.; Crudeli C.; Bhat V.; Sandilos G.; Butchy M.; Jennifer W.; Rockett G.; Echeverria K.;	2022	Annals of Surgical Oncology	29	SUPPL 2	S482-S483	https://dx.doi.org/10.1245/s10434-022-11828-2	Kooragayala 2022	Exclusion reason: Only Abstract Available;

	Atabek U.; Hong Y.								
VALIDATION OF THE NEW VA NATIONAL PATHOLOGY DATABASE FOR REPORTING COLONOSCOPY ASSOCIATED COLORECTAL ADENOMA AND ADENOCARCINOMA DETECTION RATES FOR QUALITY ASSURANCE	Gawron A.J.; McKee G.; Dominitz J.A.; Yao Y.; Whooley M.A.; Kaltenbach T.R.	2022	Gastrointestinal Endoscopy	95	6 Supplement	AB84-AB85	https://dx.doi.org/10.1016/j.gie.2022.04.243	Gawron 2022	Exclusion reason: Only Abstract Available;
NATIONAL IMPLEMENTATION OF EVIDENCE-BASED COLONOSCOPY QUALITY MEASUREMENT AND REPORTING - INITIAL UPTAKE OF A LARGE OPERATIONAL PROGRAM	Kaltenbach T.R.; Dominitz J.A.; Gupta S.; Yao Y.; McKee G.; Bailey T.; Helfrich C.; Mog A.; Millar M.; Presson A.P.; Patterson O.; Whooley M.A.; Gawron A.	2022	Gastrointestinal Endoscopy	95	6 Supplement	AB168	https://dx.doi.org/10.1016/j.gie.2022.04.445	Kaltenbach 2022	Exclusion reason: Only Abstract Available;
NATURAL LANGUAGE PROCESSING OF ESOPHAGOGASTRODUODENOSCOPY REPORTS FOR INFORMATION EXTRACTION OF GASTRIC DISEASES	Bae J.H.; Han H.W.; Song G.	2022	Gastrointestinal Endoscopy	95	6 Supplement	AB247-AB248	https://dx.doi.org/10.1016/j.gie.2022.04.646	Bae 2022	Exclusion reason: Only Abstract Available;
Using natural language processing to improve automatic ascertainment of key colonoscopy findings	Wang L.; Murphy C.C.; Skinner C.S.; Skinner M.A.; Borton E.K.; Ortiz C.M.; Hu E.; Lykken J.M.; Halm E.A.	2021	American Journal of Gastroenterology	116	SUPPL	S129	https://dx.doi.org/10.14309/01.ajg.0000773652.52042.1a	Wang 2021	Exclusion reason: Only Abstract Available;

Validation of a machine learning algorithm to measure adenoma detection rate from screening colonoscopies in a large health system	Galoosian A.; Soroudi C.; Jain S.; Kahlon S.; McLeod M.R.; Kokaly A.N.; Yang J.; Peterson E.; Maehara C.; Yang L.; Muthusamy V.; Esrailian E.; Hsu W.; May F.	2021	American Journal of Gastroenterology	116	SUPPL	S115	https://dx.doi.org/10.14309/01.ajg.0000773508.71234.2b	Galoosian 2021	Exclusion reason: Only Abstract Available;
A natural language processing algorithm significantly improves nafld recognition within a large academic health system	Pung L.; Knechtle W.; Gao M.; Gollapudy S.; Hintze B.; Nichols M.; Sendak M.; Balu S.; Muir A.J.; Patel Y.	2021	Hepatology	74	SUPPL 1	994A	https://dx.doi.org/10.1002/hep.32188	Pung 2021	Exclusion reason: Only Abstract Available;
Identification and characterization of fistulizing Crohn's disease in EHR using structured and unstructured data	Huisingh C.; Ye Y.; Wegrzyn L.	2021	Pharmacoepidemiology and Drug Safety	30	SUPPL 1	107	https://dx.doi.org/10.1002/pds.5305	Huisingh 2021	Exclusion reason: Only Abstract Available;
ID: 3522314 EXCESSIVE BIOPSIES IN PATIENTS WITH AN IRREGULAR Z LINE AND NO HISTORY OF BARRETT'S ESOPHAGUS	Kaminsky T.J.; Colletier K.; Fang J.C.; Byrne K.R.; Gawron A.J.	2021	Gastrointestinal Endoscopy	93	6 Supplement	AB306	https://dx.doi.org/10.1016/j.gie.2021.03.629	Kaminsky 2021	Exclusion reason: Only Abstract Available;
Phenotypic characterisation of suspected small bowel crohn's disease with natural language processing of mre reports	Materacki L.; Collins D.; Zeki S.; Brown J.	2021	Gut	70	SUPPL 1	A107-A108	https://dx.doi.org/10.1136/gutjnl-2020-bsgcampus.201	Materacki 2021	Exclusion reason: Only

									Abstract Available;
Natural language processing driven comparison of small bowel MRI and capsule endoscopy reporting for crohn's	Stammers M.; Hang P.; Borca F.; Minto M.; Khurshid B.; Rahmany S.; Hawkes E.; Rahman I.; Smith T.; Batchelor J.; Gwiggner M.	202 0	United European Gastroenterology Journal	8	8 SUPPL	823-824	https://dx.doi.org/10.1177/2050640620927345	Stammers 2020	Exclusion reason: Only Abstract Available;
Identification & service evaluation/improvement of a primary sclerosing cholangitis cohort using natural language processing	Stammers M.; Phan H.; Borca F.; Dixey A.; Minto M.; Rubio-Padilla M.; Razzi Q.; Tehami N.; Smith T.; Batchelor J.; Gwiggner M.; Patel J.	202 0	United European Gastroenterology Journal	8	8 SUPPL	640	https://dx.doi.org/10.1177/2050640620927345	Stammers 2020	Exclusion reason: Only Abstract Available;
Polygenic risk scores for transaminases are associated with hepatic steatosis and cirrhosis	Chen V.; Du X.; Chen Y.; Stetson L.; Speliotes E.K.	202 0	Hepatology	72	1 SUPPL	366A	https://dx.doi.org/10.1002/hep.31579	Chen 2020	Exclusion reason: Only Abstract Available;
Identification of patients with non-alcoholic steatohepatitis (NASH) in an electronic health record (EHR) database	Bertoia M.L.; Seeger J.D.; Ness E.; Capozza T.; Wong B.; Titievsky L.	202 0	Hepatology	72	1 SUPPL	974A	https://dx.doi.org/10.1002/hep.31579	Bertoia 2020	Exclusion reason: Only Abstract Available;

DETECTION AND CHARACTERIZATION OF EXTRA-INTESTINAL MANIFESTATIONS OF IBD IN CLINICAL OFFICE NOTES USING NATURAL LANGUAGE PROCESSING	Stidham R.; Yu D.; Lahiri S.; Vydiswaran V.	2020	Gastroenterology	158	6 Supplement 1	S-702	https://dx.doi.org/10.1016/S0016-5085%2820%2932446-X	Stidham 2020	Exclusion reason: Only Abstract Available;
ACCURACY OF NATURAL LANGUAGE PROCESSING AND BIOINFORMATICS APPROACHES IN IDENTIFYING PATIENT ELIGIBILITY FOR BARRETT'S ESOPHAGUS SCREENING CLINICAL TRIALS	White B.A.; Wang Y.; Johnson M.L.; Lansing R.; Leggett C.L.; Hongfang L.; Iyer P.G.	2020	Gastroenterology	158	6 Supplement 1	S-307	https://dx.doi.org/10.1016/S0016-5085%2820%2931466-9	White 2020	Exclusion reason: Only Abstract Available;
IDENTIFYING PATIENTS WITH ACUTE GASTROINTESTINAL BLEEDING WITH ELECTRONIC HEALTH RECORD PHENOTYPES	Shung D.; Tsay C.; Laine L.; Thomas P.; Partridge C.M.; Hsiao A.; Taylor R.A.	2020	Gastroenterology	158	6 Supplement 1	S-252	https://dx.doi.org/10.1016/S0016-5085%2820%2931310-X	Shung 2020	Exclusion reason: Only Abstract Available;
VALIDATION OF A NATURAL LANGUAGE PROCESSING ALGORITHM TO IDENTIFY COLONIC ADENOMAS ACROSS A HEALTH SYSTEM	Morgan D.G.; Chorneyko K.; Swain D.; Bowes B.; Lee V.; Tinmouth J.	2019	Gastroenterology	156	6 S1	S-56	https://dx.doi.org/10.1016/S0016-5085%2819%2936923-9	Morgan 2019	Exclusion reason: Only Abstract Available;
INCIDENT DIAGNOSES OF GASTRIC INTESTINAL METAPLASIA IN THE US: PATIENT CHARACTERISTICS, EGD FINDINGS, AND CLINICAL PRACTICE PATTERNS AT A LARGE US TERTIARY CARE CENTER	Parbhu S.K.; Cole G.G.; Fang J.C.; Sossenheimer M.; Peterson K.A.; Gawron A.J.	2019	Gastroenterology	156	6 Supplement 1	S-520	https://dx.doi.org/10.1016/S0016-5085%2819%2938178-8	Parbhu 2019	Exclusion reason: Weak Validation Only (Type 1b or less);

USING A MACHINE LEARNING PROGRAM - THE CLINICAL ANNOTATION RESEARCH KIT (CLARK!) - TO IDENTIFY PATIENTS WITH UNDIAGNOSED NAFLD	Kim H.P.; Bradford R.L.; Pfaff E.; Barritt A.S.	2019	Gastroenterology	156	6 Supplement 1	S-76	https://dx.doi.org/10.1016/S0016-5085%2819%2936976-8	Kim 2019	Exclusion reason: Only Abstract Available;
Usefulness of Natural Language Processing (NLP) combined with deep machine learning as a tool for clinical research in Crohn's Disease. PRE MONITION-CD Study	Fernandez-Nistal A.; Martinez V.; Gomollon Garcia F.; Tagarro I.; Medrano I.; Montoto Otero C.	2019	United European Gastroenterology Journal	7	8 Supplement	881	https://dx.doi.org/10.1177/205064061985467	Fernandez-Nistal 2019	Exclusion reason: Only Abstract Available;
Diarrheal symptoms extracted from clinical notes for patients testing positive for clostridioides difficile in the inpatient, outpatient, and long-term care settings	Stevens V.W.; Divita G.; Khader K.; Samore M.	2019	Antimicrobial Resistance and Infection Control	8	Supplement 1		https://dx.doi.org/10.1186/s13756-019-0567-6	Stevens 2019	Exclusion reason: Only Abstract Available;
Identification of IBD cohorts from linked endoscopy and histology reports using natural language processing	Brown J.; Zeki S.	2019	Gut	68	Supplement 2	A224	https://dx.doi.org/10.1136/gutjnl-2019-BSGAbstracts.426	Brown 2019	Exclusion reason: Only Abstract Available;
Use of natural language processing (NLP) to identify neurocognitive deficits in end-stage liver disease	Dickerson L.; Rouhizadeh M.; Bowring M.; Mogul D.	2019	American Journal of Transplantation	19	Supplement 1	29-30	https://dx.doi.org/10.1111/ajt.15186	Dickerson 2019	Exclusion reason: Only Abstract Available;
Development and comparison of two natural language processing	Taggart M.; Chapman W.W.; Steinberg B.A.;	2018	Circulation: Cardiovascular	11	Supplement 1			Taggart 2018	Exclusion reason: Only

methods for identifying bleeding events in clinical text	Ruckel S.; Pregenzer-Wenzler A.; Du Y.; Ferraro J.; Bucher B.T.; Lloyd-Jones D.M.; Rondina M.T.; Shah R.U.		Quality and Outcomes						Abstract Available;
Ultra-high accuracy in artificial intelligence-based classification of radiology reports from a necrotizing enterocolitis database: Results from a cross-institutional investigation	Crowley P.A.; Brociner E.; Uppili H.; Kohane I.	2018	Journal of Pediatric Gastroenterology and Nutrition	67	Supplement 1	S10-S11	https://dx.doi.org/10.1097/MPG.0000000000002164	Crowley 2018	Exclusion reason: Only Abstract Available;
Risk of nonalcoholic fatty liver disease (NAFLD)-related liver complications in the diabetic population in Israel	Goldshtein I.; Fernandes G.; Chodick G.; Rajpathak S.; Karasik A.	2018	Diabetes	67	Supplement 1	A419		Goldshtein 2018	Exclusion reason: Only Abstract Available;
A national us healthcare system web-based colonoscopy quality report card: Accurate, usable, and robust	Gawron A.J.; Yao Y.; Thompson W.; Patterson O.; Cole G.G.; Gupta S.; Scuba W.; Divita G.; Dominitz J.A.; Whooley M.A.; Shergill A.K.; Kahi C.J.; Kaltenbach T.R.	2018	Gastrointestinal Endoscopy	87	6 Supplement 1	AB114-AB115		Gawron 2018	Exclusion reason: Only Abstract Available;
Using natural language processing to assess palliative care processes in cancer patients receiving venting gastrostomy tube	Lindvall C.; Lilley E.J.; Cooper Z.; Forsyth A.W.; Barzilay R.; Lorenz K.A.;	2017	Journal of Clinical Oncology	35	31 Supplement 1	7	https://dx.doi.org/10.1200/JCO.2017.35.31_suppl.7	Lindvall 2017	Exclusion reason: Only Abstract Available;

	Walling A.M.; Tulsky J.A.								
SemEHR: Surfacing semantic data from clinical notes in electronic health records for tailored care, trial recruitment, and clinical research	Wu H.; Toti G.; Morley K.I.; Ibrahim Z.; Folarin A.; Kartoglu I.; Jackson R.; Agrawal A.; Stringer C.; Gale D.; Gorrell G.M.; Roberts A.; Broadbent M.; Stewart R.; Dobson R.J.B.	2017	The Lancet	390	SPEC.ISS 1	S97		Wu 2017	Exclusion reason: Wrong indication ;
Validation of a hybrid natural language processing tool utilizing optical character recognition for data extraction from scanned colonoscopy reports	Hayat U.; Isseh M.; Isseh N.; Ibrahim M.; McMichael J.; Lopez R.; Bhatt A.; Rhodes C.; Burke C.A.; Rizk M.	2017	Gastrointestinal Endoscopy	85	5 Supplement 1	AB417-AB418		Hayat 2017	Exclusion reason: Only Abstract Available;
Serrated polyp detection is related to specialty training and colonoscopy volume: Results from a large multicenter colonoscopy quality study	Crockett S.; Gourevitch R.A.; Morris M.; Carrell D.; Rose S.; Leffler D.; Greer J.B.; Schoen R.E.; Mehrotra A.	2017	Gastrointestinal Endoscopy	85	5 Supplement 1	AB373		Crockett 2017	Exclusion reason: Only Abstract Available;
Utilizing natural language processing (NLP) to accurately identify fatty liver disease	Redman J.S.; Natarajan Y.; Wang J.; Hanif M.; Feng H.; Kramer J.R.; Desiderio R.; Xu	2017	Gastroenterology	152	5 Supplement 1	S1115		Redman 2017	Exclusion reason: Only Abstract Available;

	H.; El-Serag H.B.; Hou J.K.; Kanwal F.								
Development and validation of a novel, semi-automated adenoma detection rate reporting system utilizing natural language processing in a veterans affairs gastroenterology department	Wu R.M.; Kaplan D.E.; Khan N.	2017	Gastroenterology	152	5 Supplement 1	S218		Wu 2017	Exclusion reason: Only Abstract Available;
Increased biologic use and lower surgical rates in pediatric versus adult-onset Crohn's disease at a large tertiary-care center over a 6-year follow-up	Kurowski J.A.; Milinovich A.; Chagin K.; Ji X.; Bauman J.; Sugano D.; Kattan M.; Achkar J.-P.	2017	Gastroenterology	152	5 Supplement 1	S201-S202		Kurowski 2017	Exclusion reason: Only Abstract Available;
Variation in colonoscopy quality across physicians in a large, multicenter study	Mehrotra A.; Gourevitch R.A.; Morris M.; Rose S.; Carrell D.; Leffler D.; Greer J.B.; Crockett S.; Schoen R.E.	2017	Gastroenterology	152	5 Supplement 1	S52-S53		Mehrotra 2017	Exclusion reason: Only Abstract Available;
Extracting phenotypes of hepatocellular carcinoma from electronic medical records (EMRs) using natural language processing	Chen L.; Song L.; Li D.; Ding K.	2017	Hepatology International	11	1 Supplement 1	S436	https://dx.doi.org/10.1007/s12072-016-9783-9	Chen 2017	Exclusion reason: Only Abstract Available;
An electronic medical record-based scoring system for estimating the risk of advanced colorectal neoplasia in veterans	Imperiale T.F.; Imler T.; Sherer E.A.; Kahi C.; Larson J.; Cardwell J.; Johnson C.S.;	2016	American Journal of Gastroenterology	111	Supplement 1	S105-S106	https://dx.doi.org/10.1038/ajg.2016.353	Imperiale 2016	Exclusion reason: Only Abstract Available;

	Antaki F.; Ashley C.; Baffy G.; Cho I.; Dominitz J.A.; Hou J.K.; Korsten M.A.; Nagar A.B.; Patel S.; Promrat K.; Robertson D.; Saini S.; Shaw R.; Shergill A.; Smalley W.E.								
Quality improvement natural language processing colonoscopy evaluation tool (QUINCE): A flexible, portable tool to extract pathology results for colonoscopy quality reporting	Gawron A.J.; Pacheco J.A.; Scuba B.; Chapman W.; Kaltenbach T.; Thompson W.K.	2016	Gastroenterology	150	4 SUPPL. 1	S637		Gawron 2016	Exclusion reason: Only Abstract Available;
Natural language processing accurately calculates adenoma and sessile serrated polyp detection rates	Nayor J.; Goryachev S.; Gainer V.S.; Saltzman J.R.	2016	Gastroenterology	150	4 SUPPL. 1	S61		Nayor 2016	Exclusion reason: Only Abstract Available;
Proximal serrated polyp detection rate does not differ among morning or afternoon colonoscopy: Analysis of a large, single, high-volume center using natural language processing	Patel V.; Thompson W.; Goldstein J.L.; Yen E.	2015	American Journal of Gastroenterology	110	Supplement 1	S590-S591		Patel 2015	Exclusion reason: Only Abstract Available;
Indexed natural language processing to identify adenoma detection RATES	Imler T.D.; Imperiale T.F.	2015	Gastrointestinal Endoscopy	81	5 SUPPL. 1	AB215-AB216	https://dx.doi.org/10.1016/j.gie.2015.03.208	Imler 2015	Exclusion reason: Only Abstract Available;

Using an automated diagnostic algorithm that utilizes electronic health records and natural language processing to define a population with cirrhosis	Chang E.K.; Yu C.Y.; Clarke R.; Hackbarth A.D.; Sanders T.; Esrailian E.; Hommes D.W.; Runyon B.A.	2015	Gastroenterology	148	4 SUPPL. 1	S1074		Chang 2015	Exclusion reason: Only Abstract Available;
Web-based EMR-linked cancer tracking application facilitates earlier detection of hepatocellular carcinoma	Shen L.; Taddei T.	2015	Gastroenterology	148	4 SUPPL. 1	S1069		Shen 2015	Exclusion reason: Only Abstract Available;
Cohort identification of hepatocellular adenoma by natural language processing	Imler T.D.; Lammert C.	2015	Gastroenterology	148	4 SUPPL. 1	S965		Imler 2015	Exclusion reason: Only Abstract Available;
Medical, social, and legal risks to predict alcoholic liver disease using natural language processing and advanced analytics	Imler T.D.; Ring N.; Crabb D.W.	2015	Gastroenterology	148	4 SUPPL. 1	S499		Imler 2015	Exclusion reason: Only Abstract Available;
Reliably Filter Drug-Induced Liver Injury Literature With Natural Language Processing and Conformal Prediction	Zhan, X.; Wang, F.; Gevaert, O.	2022	IEEE J. Biomedical Health Informat.	26	10	5033-5041	10.1109/JBHI.2022.3193365	Zhan 2022	Exclusion reason: Wrong outcomes;
Consolidated EHR workflow for endoscopy quality reporting	Syed, S.; Tharian, B.; Syeda, H.B.; Zozus, M.; Greer, M.L.; Bhattacharyya,	2021	Public Health and Inform.: Proc. of MIE 2021			427-431	10.3233/SHTI210194	Syed 2021	Exclusion reason: Weak Validation Only

	S.; Syed, M.; Prior, F.								(Type 1b or less);
Can natural language processing help differentiate inflammatory intestinal diseases in China? Models applying random forest and convolutional neural network approaches	Tong, Y.; Lu, K.; Yang, Y.; Li, J.; Lin, Y.; Wu, D.; Yang, A.; Li, Y.; Yu, S.; Qian, J.	2020	BMC Med. Informatics Decis. Mak.	20	1		10.1186/s12911-020-01277-w	Tong 2020	Exclusion reason: Lack of Validation;
Identification and Characterization of Nonalcoholic Fatty Liver Disease	Goldshtein, I.; Chodick, G.; Kochba, I.; Gal, N.; Webb, M.; Shibolet, O.	2020	Clin. Gastroenterol. Hepatol.	18	8	1887-1889	10.1016/j.cgh.2019.08.007	Goldshtein 2020	Exclusion reason: Lack of Validation;
Use of Narrative Concepts in Electronic Health Records to Validate Associations Between Genetic Factors and Response to Treatment of Inflammatory Bowel Diseases	Ananthakrishnan, A.N.; Cagan, A.; Cai, T.; Gainer, V.S.; Savova, G.; Shaw, S.Y.; Churchill, S.; Burke, K.E.; Karlson, E.W.; Murphy, S.N.; Kohane, I.; Liao, K.P.; Xavier, R.J.	2020	Clin. Gastroenterol. Hepatol.	18	8	1890-1892	10.1016/j.cgh.2019.08.005	Ananthakrishnan 2020	Exclusion reason: Lack of Validation;
Language impairment in adults with end-stage liver disease: application of natural language processing towards patient-generated health records	Dickerson, L.K.; Rouhizadeh, M.; Korotkaya, Y.; Bowring, M.G.; Massie, A.B.; McAdams-Demarco, M.A.; Segev, D.L.; Cannon, A.; Guerrerio, A.L.; Chen, P.-H.; Philosophe,	2019	npj Digit. Med.	2	1		10.1038/s41746-019-0179-9	Dickerson 2019	Exclusion reason: Lack of Validation;

	B.N.; Mogul, D.B.								
Natural Language Processing of Radiology Reports in Patients With Hepatocellular Carcinoma to Predict Radiology Resource Utilization	Brown, A.D.; Kachura, J.R.	2019	J. Am. Coll. Radiol.	16	6	840-844	10.1016/j.jacr.2018.12.004	Brown 2019	Exclusion reason: Wrong indication;
A Case Study of the Incremental Utility for Disease Identification of Natural Language Processing in Electronic Medical Records	Weiss, L.S.; Zhou, X.; Walker, A.M.; Ananthakrishnan, A.N.; Shen, R.; Sobel, R.E.; Bate, A.; Reynolds, R.F.	2018	Pharm. Med.	32	1	31-37	10.1007/s40290-017-0216-4	Weiss 2018	Exclusion reason: Weak Validation Only (Type 1b or less)
Natural language processing as an alternative to manual reporting of colonoscopy quality metrics	Raju, G.S.; Lum, P.J.; Slack, R.S.; Thirumurthi, S.; Lynch, P.M.; Miller, E.; Weston, B.R.; Davila, M.L.; Bhutani, M.S.; Shafi, M.A.; Bresalier, R.S.; Dekovich, A.A.; Lee, J.H.; Guha, S.; Pande, M.; Blechacz, B.; Rashid, A.; Routbort, M.; Shuttlesworth, G.; Mishra, L.; Stroehlein, J.R.; Ross, W.A.	2015	Gastrointest. Endosc.	82	3	512-519	10.1016/j.gie.2015.01.049	Raju 2015	Exclusion reason: Weak Validation Only (Type 1b or less);

Clinical Decision Support With Natural Language Processing Facilitates Determination of Colonoscopy Surveillance Intervals	Imler, Timothy D.; Morea, Justin; Imperiale, Thomas F.	2014	Clinical Gastroenterology and Hepatology	12	7	1130-1136	10.1016/j.cgh.2013.11.025	Imler 2014	Exclusion reason: Wrong Dates;
TAX-Corpus: Taxonomy based Annotations for Colonoscopy Evaluation	Syed, S et al	2022	Biomed Eng Syst Tech			162-169	doi:10.5220/0010876100003123	Syed 2022	Exclusion reason: Part of Larger Study;
Tumor reference resolution and characteristic extraction in radiology reports for liver cancer stage prediction.	Yim et al.	2023	J_Biomed_Inform	64		179-191		Yim 2023	Exclusion reason: Wrong Dates;
Utilising NLP With Chemical and Disease Similarities to Drive the Identification of Drug-Induced Liver Injury Literature	Katritsis et al	2022	Front Genet.	13			doi: 10.3389/fgene.2022.894209	Katritsis 2022	Exclusion reason: Wrong outcomes;
