# Supplementary material for: A foundation systematic review of natural language processing applied to gastroenterology & hepatology
Source: BMC Gastroenterol. 2025 Feb 6;25:58. doi: 10.1186/s12876-025-03608-5 (PMC11800601; doi:10.1186/s12876-025-03608-5)
Supplement: Supplementary file 10 — Supplementary Material 10. [file 12876_2025_3608_MOESM10_ESM.pdf]

Supplemental File 10: Abstract Screening (Excluded Abstracts)

Table J. Abstract Screening Exclusions

Title	Authors	Year	Journal	Vol	Issue	Pages	DOI	Study	Notes
Sa1399 Emergency Re-Admissions Within 30 Days Following ERCP: Occurrence, Predictors, and Relation With All-Cause Mortality in a Nationwide Population-Based Cohort	Kalaitzakis, Evangelos	2015	Gastrointestinal Endoscopy	81	5	AB198		Kalaitzakis 2015	Non-NLP Focus
PTH-34 Diagnostic outcomes after cancelled gastroscopy: telephone triage of 600 patients during the COVID-19 pandemic	Mozdiak, Ella; Chambers, J. C.; Owusu, R. A. S.; Palaneer, S.; Kulendrarajah, B.; Scarlett, D. E.; Hebbar, S.	2021						Mozdiak 2021	Non-NLP Focus
Artificial intelligence in gastroenterology: A state-of-the-art review	KrÃ¶ner, Paul T.; Engels, Megan ML; Glicksberg, Benjamin S.; Johnson, Kipp W.; Mzaik, Obaie; van Hooft, Jeanin E.; Wallace, Michael B.; El-Serag, Hashem B.; Krittanawong, Chayakrit	2021	World journal of gastroenterology	27	40	6794		KrÃ¶ner 2021	Review/Perspective Article Only

Supporting information retrieval from electronic health records: A report of University of Michiganâ€™s nine-year experience in developing and using the Electronic Medical Record Search Engine (EMERSE)	Hanauer, David A.; Mei, Qiaozhu; Law, James; Khanna, Ritu; Zheng, Kai	2015	Journal of biomedical informatics	55		290-300		Hanauer 2015	Non-Gastro/Hepatology Focus
Pretreatment frailty is independently associated with increased risk of infections after immunosuppression in patients with inflammatory bowel diseases	Kochar, Bharati; Cai, Winston; Cagan, Andrew; Ananthakrishnan, Ashwin N.	2020	Gastroenterology	158	8	2104-2111		Kochar 2020	Non-NLP Focus
The Natural History of Asymptomatic Gallstones: A Longitudinal Study and Prediction Model	Morris-Stiff, Gareth; Sarvepalli, Shashank; Hu, Bo; Gupta, Niyati; Lal, Pooja; Burke, Carol A.; Garber, Ari; McMichael, John; Rizk, Maged K.; Vargo, John J.	2022	Clinical Gastroenterology and Hepatology					Morris-Stiff 2022	Non-NLP Focus
High-powered magnet exposures in children: a	Middelberg, Leah K.; Leonard, Julie	2022	Pediatrics	149	3			Middelberg 2022	Non-Gastro/Hepatology Focus

multi-center cohort study	C.; Shi, Junxin; Aranda, Arturo; Brown, Julie C.; Cochran, Christina L.; Eastep, Kasi; Gonzalez, Raquel; Haasz, Maya; Herskovitz, Scott								
NIH/NCI R03CA230947	Dominitz, Robertson MPI								Non-NLP Focus
Risk factors and incidence of gastric cancer after detection of Helicobacter pylori infection: a large cohort study	Kumar, Shria; Metz, David C.; Ellenberg, Susan; Kaplan, David E.; Goldberg, David S.	2020	Gastroenterology	158	3	527-536		Kumar 2020	Non-NLP Focus
Power of big data to improve patient care in gastroenterology	Catlow, Jamie; Bray, Benjamin; Morris, Eva; Rutter, Matt	2022	Frontline Gastroenterology	13	3	237-244		Catlow 2022	Review/Perspective Article Only
Big data in IBD: a look into the future	Olivera, Pablo; Danese, Silvio; Jay, Nicolas; Natoli, Gioacchino; Peyrin-Biroulet, Laurent	2019	Nature Reviews Gastroenterology & Hepatology	16	5	312-321		Olivera 2019	Review/Perspective Article Only
A systematic review of Crohn's disease case definitions in administrative or claims databases	Hutfless, Susan; Jasper, Ryan A.; Tilak, Aman; Ghosh, Tamoghna; Kedia, Saurabh; Liu, Simon; Urrunaga, Nathalie H.;	2022	Inflamm Bowel Dis					Hutfless 2022	Review/Perspective Article Only

	Josephson, Matthew; Narang, Arshit; Miller, Steve								
Intelligent oncology: The convergence of artificial intelligence and oncology	Lin, Bo; Tan, Zhibo; Mo, Yaqi; Yang, Xue; Liu, Yajie; Xu, Bo	2022	Journal of the National Cancer Center					Lin 2022	Non-Gastro/Hepatology Focus
A Framework for Augmented Intelligence in Allergy and Immunology Practice and Researchâ€”A Work Group Report of the AAAAI Health Informatics, Technology, and Education Committee	Khoury, Paneez; Srinivasan, Renganathan; Kakumanu, Sujani; Ochoa, Sebastian; Keswani, Anjeni; Sparks, Rachel; Rider, Nicholas L.	2022	The Journal of Allergy and Clinical Immunology: In Practice	10	5	1178-1188		Khoury 2022	Non-Gastro/Hepatology Focus
Application of artificial intelligence in pancreaticobiliary diseases	Goyal, Hemant; Mann, Rupinder; Gandhi, Zainab; Perisetti, Abhilash; Zhang, Zhongheng; Sharma, Neil; Saligram, Shreyas; Inamdar, Sumant; Tharian, Benjamin	2021	Therapeutic advances in gastrointestinal endoscopy	14		2.63E+15		Goyal 2021	Non-NLP Focus

A bibliometric analysis of 23,492 publications on rectal cancer by machine learning: basic medical research is needed	Wang, Kangtao; Feng, Chenzhe; Li, Ming; Pei, Qian; Li, Yuqiang; Zhu, Hong; Song, Xiangping; Pei, Haiping; Tan, Fengbo	2020	Therapeutic advances in gastroenterology	13		1.76E+15		Wang 2020	Review/Perspective Article Only
Colonoscopy quality assessment	Fayad, Nabil F.; Kahi, Charles J.	2015	Gastrointestinal Endoscopy Clinics	25	2	373-386		Fayad 2015	Review/Perspective Article Only
Machine learning for liver disease classification	Jesty, Benjamin	2019						Jesty 2019	Non-NLP Focus
The challenges of implementing artificial intelligence into surgical practice	Tranter-Entwistle, Isaac; Wang, Holly; Daly, Kenny; Maxwell, Scott; Connor, Saxon	2021	World Journal of Surgery	45	2	420-428		Tranter-Entwistle 2021	Non-Gastro/Hepatology Focus
AI REVOLUTIONIZING HEALTH CARE: A DESCRIPTIVE REVIEW.	Priya, Bansal; Kumar, Jha Avishek; Khushboo, Arora; Neha, Gupta; Vanshika, Bhardwaj; Abhishek, Kumar	2021	Journal of Advanced Scientific Research	12				Priya 2021	Review/Perspective Article Only
Modern Machine Learning Practices in Colorectal Surgery: A Scoping Review	Taha-Mehlitz, Stephanie; DÃ¤ster, Silvio; Bach, Laura; Ochs, Vincent; von FlÃ¼e, Markus; Steinemann,	2022	Journal of Clinical Medicine	11	9	2431		Taha-Mehlitz 2022	Review/Perspective Article Only

	Daniel; Taha, Anas								
ohnson, KW, Mzaik, O	Kroner, P. T.; Engels, M. M.; Glicksberg, B. S.	2021	Hooft,. E. an, Krittanawong, C			6794-6824		Kroner 2021	Review/Perspective Article Only
History, current status, and future directions of artificial intelligence	Kubassova, Olga; Shaikh, Faiq; Melus, Carlos; Mahler, Michael	2021	Precision Medicine and Artificial Intelligence			Jan-38		Kubassova 2021	Review/Perspective Article Only
Role of artificial intelligence in multidisciplinary imaging diagnosis of gastrointestinal diseases	BerbÃs, M. Alvaro; Aneiros-FernÃ¡ndez, JosÃ©; Olivares, F. Javier Mendoza; Nava, Enrique; Luna, Antonio	2021	World Journal of Gastroenterology	27	27	4395		BerbÃs 2021	Non-NLP Focus
Revealing potential drug-disease-gene association patterns for precision medicine	Wang, Xuefeng; Zhang, Shuo; Wu, Yao; Yang, Xuemei	2021	Scientometrics	126	5	3723-3748		Wang 2021	Non-Gastro/Hepatology Focus
Artificial intelligence in gastric cancer: a systematic review	Jin, Peng; Ji, Xiaoyan; Kang, Wenzhe; Li, Yang; Liu, Hao; Ma, Fuhai; Ma, Shuai; Hu, Haitao; Li, Weikun; Tian, Yantao	2020	Journal of cancer research and clinical oncology	146	9	2339-2350		Jin 2020	Review/Perspective Article Only
Using a Machine Learning Model to Risk Stratify for the Presence of Significant Liver Disease in a	Bennett, Lucy; Mostafa, Mohamed; Hammersley, Richard; Purssell, Huw; Patel, Manish; Street, Oliver; Athwal, Varinder; Hanley, Karen Piper; Hanley, Neil A.; Morling, Joanne R.								Non-NLP Focus

Primary Care Population									
The use of administrative data to investigate the population burden of hepatic encephalopathy	Bloom, Patricia P.; Tapper, Elliot B.	2020	Journal of Clinical Medicine	9	11	3620		Bloom 2020	Review/Perspective Article Only
Update on Electronic Health Records	Kinberg, Sivan	2017	Update					Kinberg 2017	Review/Perspective Article Only
Smart Health: Intelligent Healthcare Systems in the Metaverse, Artificial Intelligence, and Data Science Era	Yang, Yin; Siau, Keng; Xie, Wen; Sun, Yan	2022	Journal of Organizational and End User Computing (JOEUC)	34	1	Jan-14		Yang 2022	Review/Perspective Article Only
Trends in Worldwide Research in Inflammatory Bowel Disease Over the Period 2012â€“2021: A Bibliometric Study	Li, Kemin; Feng, Chenzhe; Chen, Haolin; Feng, Yeqian; Li, Jingnan	2022	Frontiers in Medicine			1389		Li 2022	Review/Perspective Article Only
Artificial intelligence in perioperative management of major gastrointestinal surgeries	Solanki, Sohan Lal; Pandrowala, Saneya; Nayak, Abhirup; Bhandare, Manish; Ambulkar, Reshma P.; Shrikhande, Shailesh V.	2021	World Journal of Gastroenterology	27	21	2758		Solanki 2021	Review/Perspective Article Only

Clinical Applications of Artificial Intelligenceâ€”An Updated Overview	Busnatu, È˜tefan; Niculescu, Adelina-Gabriela; Bolocan, Alexandra; Petrescu, George ED; PÄƒduraru, Dan Nicolae; NÄƒstasÄƒ, Iulian; LupuÈ™oru, Mircea; GeantÄƒ, Marius; Andronic, Octavian; Grumezescu, Alexandru Mihai	2022	Journal of Clinical Medicine	11	8	2265		Busnatu 2022	Review/Perspective Article Only
Core concepts in pharmacoepidemiology: Validation of health outcomes of interest within real-world healthcare databases	Weinstein, Erica J.; Ritchey, Mary Elizabeth; Lo Re III, Vincent	2022	Pharmacoepidemiology and Drug Safety					Weinstein 2022	Non-Gastro/Hepatology Focus
Artificial intelligence and its impact on the domains of universal health coverage, health emergencies and health promotion: An overview of systematic reviews	Martinez-Millana, Antonio; Saez, Aida; Tornero, Roberto; Azzopardi-Muscat, Natasha; Traver, Vicente;	2022	International Journal of Medical Informatics			104855		Martinez-Millana 2022	Review/Perspective Article Only

	Novillo-Ortiz, David								
AIG	Maulahela, Hasan; Annisa, Nagita Gianty	2022						Maulahela 2022	Non-NLP Focus
Accuracy of an administrative database for pancreatic cancer by international classification of disease 10th codes: A retrospective large-cohort study	Hwang, Young-Jae; Park, Seon Mee; Ahn, Soomin; Lee, Jong-Chan; Park, Young Soo; Kim, Nayoung	2019	World Journal of Gastroenterology	25	37	5619		Hwang 2019	Non-NLP Focus
Outcome and Resource Use of Patients with Liver Disease: Analysis of Chinese Electronic Medical Records	Xu Jr, Yuan	2016						XuJr 2016	Abstract Only
A scoping review of artificial intelligence and machine learning in bariatric and metabolic surgery: current status and future perspectives	Pantelis, Athanasios G.; Stravodimos, Georgios K.; Lapatsanis, Dimitris P.	2021	Obesity Surgery	31	10	4555-4563		Pantelis 2021	Review/Perspective Article Only
Risk Prediction of Pancreatic Cancer in Patients With Abnormal Morphologic Findings Related to Chronic Pancreatitis: A	Chen, Wansu; Chen, Qiaoling; Parker, Rex A.; Zhou, Yichen; Lustigova, Eva; Wu, Bechien U.	2022	Gastro hep advances	1	6	1014-1026		Chen 2022	Non-NLP Focus

Machine Learning Approach									
BIBLIOMETRIC ANALYSIS OF LIVER LESION AND LIVER TUMOR DETECTION WITH ARTIFICIAL INâ„¡LIGENCE IN NUCLEAR MEDICINE	AGBOR, VALARIE ORU	2021						AGBOR 2021	Non-NLP Focus
â‰¥ 3 Nonadvanced Adenomas are More Common in the Era of Contemporary Colonoscopy and Not Associated With Metachronous Advanced Neoplasia	Rouphael, Carol; Lopez, Rocio; McMichael, John; Burke, Carol A.	2021	Journal of Clinical Gastroenterology	55	4	343-349		Rouphael 2021	Non-NLP Focus
Accuracy of Referring Provider and Endoscopist Impressions of Colonoscopy Indication	Naveed, Mariam; Clary, Meredith; Ahn, Chul; Kubiliun, Nisa; Agrawal, Deepak; Cryer, Byron; Murphy, Caitlin; Singal, Amit G.	2017	Journal of the National Comprehensive Cancer Network	15	7	920-925		Naveed 2017	Non-NLP Focus
A Network Medicine Approach to Drug Repurposing for Chronic Pancreatitis	Golden, Megan; Wilson, Jabe	2020	bioRxiv					Golden 2020	Non-NLP Focus

Artificial Intelligence: The Milestone in Modern Biomedical Research	Athanasopoulou, Konstantina; Daneva, Glykeria N.; Adamopoulos, Panagiotis G.; Scorilas, Andreas	2022	BioMedInformatics	2	4	727-744		Athanasopoulou 2022	Review/Perspective Article Only
Outlook of the future landscape of artificial intelligence in medicine and new challenges	Xing, Lei; Kapp, Daniel S.; Giger, Maryellen L.; Min, James K.	2021	Artificial intelligence in medicine			503-526		Xing 2021	Review/Perspective Article Only
Multi-Center Colonoscopy Quality Measurement Utilizing Natural Language Processing	Imler, Timothy D; Morea, Justin; Kahi, Charles; Cardwell, Jon; Johnson, Cynthia S; Xu, Huiping; Ahnen, Dennis; Antaki, Fadi; Ashley, Christopher; Baffy, Gyorgy; Cho, Ilseung; Dominitz, Jason; Hou, Jason; Korsten, Mark; Nagar, Anil; Promrat, Kittichai; Robertson, Douglas; Saini, Sameer; Shergill, Amandeep; Smalley, Walter;	2015	American Journal of Gastroenterology	110	4	543-552	10.1038/ajg.2015.51	Imler 2015	Non-NLP Focus

	Imperiale, Thomas F								
Challenges in adapting existing clinical natural language processing systems to multiple, diverse health care settings	Carrell, David S; Schoen, Robert E; Leffler, Daniel A; Morris, Michele; Rose, Sherri; Baer, Andrew; Crockett, Seth D; Gourevitch, Rebecca A; Dean, Katie M; Mehrotra, Ateev	2017	Journal of the American Medical Informatics Association	24	5	986-991	10.1093/jamia/ocx039	Carrell 2017	Non-Gastro/Hepatology Focus
Artificial Intelligence and the Future of Gastroenterology and Hepatology	Penrice, Daniel D.; Rattan, Puru; Simonetto, Douglas A.	2022	Gastro Hep Advances	1	4	581-595	10.1016/j.gastha.2022.02.025	Penrice 2022	Review/Perspective Article Only
History of artificial intelligence in medicine	Kaul, Vivek; Enslin, Sarah; Gross, Seth A.	2020	Gastrointestinal Endoscopy	92	4	807-812	10.1016/j.gie.2020.06.040	Kaul 2020	Review/Perspective Article Only
The overview of the deep learning integrated into the medical imaging of liver: a review	Xiang, Kailai; Jiang, Baihui; Shang, Dong	2021	Hepatol Int	15	4	868-880	10.1007/s12072-021-10229-z	Xiang 2021	Non-NLP Focus
Development of BDMS Utilization Review Technology (BURT): An Artificial Intelligence Tool Using Thai Natural Language Processing to Assess	Panyasorn, Jinhatha; Banomyong, Piemchok; Phetchunsakul, Kusuma; Phengpinit, Noppadol; Wiseschinda, Varut; Kunanusont, Chaiyos	2020	bkkmedj	16	2	182-195	10.31524/bkkmedj.2020.21.012	Panyasorn 2020	Non-Gastro/Hepatology Focus

Appropriateness of Hospitalization									
Application of Big Data analysis in gastrointestinal research	Cheung, Ka-Shing; Leung, Wai K; Seto, Wai-Kay	2019	WJG	25	24	2990-3008	10.3748/wjg.v25.i24.2990	Cheung 2019	Review/Perspective Article Only
Role of Artificial Intelligence Applications in Real-Life Clinical Practice: Systematic Review	Yin, Jiamin; Ngiam, Kee Yuan; Teo, Hock Hai	2021	J Med Internet Res	23	4	e25759	10.2196/25759	Yin 2021	Non-Gastro/Hepatology Focus
Optimizing Colonoscopy Quality: From Bowel Preparation to Surveillance	Abou Fadel, Carla G.; Shayto, Rani H.; Sharara, Ala I.	2016	Curr Treat Options Gastro	14	1	115-127	10.1007/s11938-016-0073-3	AbouFadel 2016	Non-NLP Focus
Recent Advances in Computed Tomography and MR Imaging	Potigailo, Valeria; Kohli, Ajay; Pakpoor, Jina; Cain, Donald Wesley; Passi, Neena; Mohsen, Nancy	2020	PET Clinics	15	4	381-402	10.1016/j.cpet.2020.07.001	Potigailo 2020	Non-Gastro/Hepatology Focus
Artificial Intelligence: Review of Current and Future Applications in Medicine	Thomas, Brannon	2021	Federal Practitioner		38 (11)		10.12788/fp.0174	Thomas 2021	Review/Perspective Article Only
The future of artificial intelligence in healthcare	Radakovich, Nathan; Nazha, Aziz	2021	Machine Learning in Cardiovascular Medicine			371-394		Radakovich 2021	Review/Perspective Article Only
The road map of cancer precision medicine with the innovation of advanced cancer detection	Low, Siew-Kee; Nakamura, Yusuke	2019	Japanese Journal of Clinical Oncology	49	7	596-603	10.1093/jjco/hyz073	Low 2019	Non-Gastro/Hepatology Focus

technology and personalized immunotherapy									
Breath VOC analysis and machine learning approaches for disease screening: a review	P, Haripriya; Rangarajan, Madhavan; Pandya, Hardik J	2023	J. Breath Res.				10.1088/1752-7163/acb283	P 2023	Non-Gastro/Hepatology Focus
Deep Learning Framework for Cancer Diagnosis and Treatment	Bahadur, Shiv; Kumar, Prashant	2022	Deep Learning for Targeted Treatments			229-245		Bahadur 2022	Review/Perspective Article Only
Narrative review of intraductal papillary mucinous neoplasms: pathogenesis, diagnosis, and treatment of a true precancerous lesion	Ma, Gang; Li, Guichen; Xiao, Zhihuan; Gou, Anjiang; Xu, Yuanhong; Song, Shaowei; Guo, Kejian; Liu, Zhe	2021	Gland Surg	10	7	2313-2324	10.21037/gs-21-450	Ma 2021	Review/Perspective Article Only
Artificial Intelligence in Translational Medicine	Brogi, Simone; Calderone, Vincenzo	2021	IJTM	1	3	223-285	10.3390/ijtm1030016	Brogi 2021	Review/Perspective Article Only
Colon Cancer Diagnosis Based on Machine Learning and Deep Learning: Modalities and Analysis Techniques	Tharwat, Mai; Sakr, Nehal A.; El-Sappagh, Shaker; Soliman, Hassan; Kwak, Kyung-Sup; Elmogy, Mohammed	2022	Sensors	22	23	9250	10.3390/s22239250	Tharwat 2022	Review/Perspective Article Only
New Perspectives on Machine Learning in Drug Discovery	Musella, Simona; Verna, Giulio; Fasano, Alessio; Di Micco, Simone	2021	CMC	28	32	6704-6728	10.2174/0929867327666201111144048	Musella 2021	Non-Gastro/Hepatology Focus

Diagnostic Error in Medicine: 10th International Conference		2017	Diagnosis	4	4	eA43-eA124	10.1515/dx-2017-0034		Non-Gastro/Hepatology Focus
When will the mist clear? On the Interpretability of Machine Learning for Medical Applications: a survey	Banegas-Luna, Antonio-JesÃºs; PeÃ±a-GarcÃa, Jorge; Iftene, Adrian; Guadagni, Fiorella; Ferroni, Patrizia; Scarpato, Noemi; Zanzotto, Fabio Massimo; Bueno-Crespo, AndrÃ©s; PÃ©rez-SÃ¡nchez, Horacio	2020					10.48550/ARXIV.2010.00353	Banegas-Luna 2020	Non-Gastro/Hepatology Focus
2019 Diabetes Technology Meeting Abstracts		2020	J Diabetes Sci Technol	14	2	361-492	10.1177/1932296819897652		Non-Gastro/Hepatology Focus
Computing and the national science foundation, 1950â€"2016: Building a foundation for modern computing	Freeman, Peter A.; Adrion, W. Richards; Aspray, William	2019						Freeman 2019	Non-Gastro/Hepatology Focus
The handbook of multimodal-multisensor interfaces: Foundations, user modeling, and common modality combinations - volume 1		2017		14					Non-Gastro/Hepatology Focus
ARES '22: Proceedings of the 17th international conference on availability, reliability and security		2022							Non-Gastro/Hepatology Focus

SA '15: SIGGRAPH asia 2015 visualization in high performance computing		2015							Non-Gastro/Hepatology Focus
Exploiting partial assignments for efficient evaluation of answer set programs with external source access	Eiter, Thomas; Kaminski, Tobias; Redl, Christoph; Weinzierl, Antonius	2018	J. Artif. Int. Res.	62	1	665-727	10.1613/jair.1.11221	Eiter 2018	Non-Gastro/Hepatology Focus
Expressive '18: Proceedings of the joint symposium on computational aesthetics and sketch-based interfaces and modeling and non-photorealistic animation and rendering		2018							Non-Gastro/Hepatology Focus
ICDCN '23: Proceedings of the 24th international conference on distributed computing and networking		2023							Non-Gastro/Hepatology Focus
MobileHCI '22: Adjunct publication of the 24th international conference on human-computer interaction with mobile devices and services		2022							Non-Gastro/Hepatology Focus
A novel approach to detecting epistasis using random sampling regularisation	Hind, Jade; Lisboa, Paulo; Hussain, Abir J.; Al-Jumeily, Dhiya	2020	IEEE/ACM Trans. Comput. Biol. Bioinformatics	17	5	1535-1545	10.1109/TCBB.2019.2948330	Hind 2020	Non-Gastro/Hepatology Focus
Making personalized movie recommendations for children	Tan, Eunice; Seaman, Iris; Leung, Humphrey; Ng, Yiu-Kai	2016				96-105	10.1145/3011141.3011142	Tan 2016	Non-Gastro/Hepatology Focus
Duplicate detection in programming question	Zhang, Wei Emma; Sheng, Quan Z.; Lau, Jey Han; Abebe,	2018	ACM Trans. Internet Technol.	18	3		10.1145/3169795	Zhang 2018	Non-Gastro/Hepatology Focus

answering communities	Ermyas; Ruan, Wenjie								
Integrative high dimensional multiple testing with heterogeneity under data sharing constraints	Liu, Molei; Xia, Yin; Cho, Kelly; Cai, Tianxi	2022	J. Mach. Learn. Res.	22	1			Liu 2022	Non-Gastro/Hepatology Focus
Accessible conversational user interfaces: Considerations for design	Lister, Kate; Coughlan, Tim; Iniesto, Francisco; Freear, Nick; Devine, Peter	2020					10.1145/3371300.3383343	Lister 2020	Non-Gastro/Hepatology Focus
Ensemble deep learning on wearables using small datasets	Mauldin, Taylor; Ngu, Anne H.; Metsis, Vangelis; Canby, Marc E.	2021	ACM Trans. Comput. Healthcare	2	1		10.1145/3428666	Mauldin 2021	Non-Gastro/Hepatology Focus
SA '16: SIGGRAPH ASIA 2016 courses		2016							Non-Gastro/Hepatology Focus
Joint optimization of masks and deep recurrent neural networks for monaural source separation	Huang, Po-Sen; Kim, Minje; Hasegawa-Johnson, Mark; Smaragdis, Paris	2015	IEEE/ACM Trans. Audio, Speech and Lang. Proc.	23	12	2136-2147	10.1109/TASLP.2015.2468583	Huang 2015	Non-Gastro/Hepatology Focus
Generating look-alike names for security challenges	Han, Shuchu; Hu, Yifan; Skiena, Steven; Coskun, Baris; Liu, Meizhu; Qin, Hong; Perez, Jaime	2017				57-67	10.1145/3128572.3140441	Han 2017	Non-Gastro/Hepatology Focus
IDEAS '22: Proceedings of the 26th international database engineered applications symposium		2022							Non-Gastro/Hepatology Focus

SnapToReality: Aligning augmented reality to the real world	Nuernberger, Benjamin; Ofek, Eyal; Benko, Hrvoje; Wilson, Andrew D.	2016				1233-1244	10.1145/2858036.2858250	Nuernberger 2016	Non-Gastro/Hepatology Focus
IMMS '22: Proceedings of the 5th international conference on information management and management science		2022							Non-Gastro/Hepatology Focus
A method to analyze multiple social identities in twitter bios	Pathak, Arjunil; Madani, Navid; Joseph, Kenneth	2021	Proc. ACM Hum.-Comput. Interact.	5	CSCW2		10.1145/3479502	Pathak 2021	Non-Gastro/Hepatology Focus
ICCIP '22: Proceedings of the 8th international conference on communication and information processing		2022							Non-Gastro/Hepatology Focus
SIGGRAPH '18: ACM SIGGRAPH 2018 appy hour		2018							Non-Gastro/Hepatology Focus
Crawling, indexing, and retrieving moments in videogames	Zhang, Xiaoxuan; Zhan, Zeping; Holtz, Misha; Smith, Adam M.	2018					10.1145/3235765.3235786	Zhang 2018	Non-Gastro/Hepatology Focus
Spooky technology: The ethereal and otherworldly as a resource for design	Byrne, Daragh; Lockton, Dan; Hu, Meijie; Luong, Miranda; Ranade, Anuprita; Escarcha, Karen; Giesa, Katherine; Huang, Yiwei; Yochum, Catherine; Robertson, Gordon; Yeung, Lisa (Yip Yan);	2022				759-775	10.1145/3532106.3533547	Byrne 2022	Non-Gastro/Hepatology Focus

	Cruz, Matthew; Danner, Christi; Wang, Elizabeth; Khurana, Malika; Chen, Zhenfang; Heyison, Alexander; Fu, Yixiao								
Data and process quality evaluation in a textual big data archiving system	Fugini, Mariagrazia; Finocchi, Jacopo	2022	J. Comput. Cult. Herit.	15	1		10.1145/3461015	Fugini 2022	Non-Gastro/Hepatology Focus
Data-driven shape analysis and processing	Xu, Kai; Kim, Vladimir G.; Huang, Qixing; Mitra, Niloy; Kalogerakis, Evangelos	2016					10.1145/2988458.2988473	Xu 2016	Non-Gastro/Hepatology Focus
â€œI didnâ€™t know I looked angryâ€�: Characterizing observed emotion and reported affect at work	Kaur, Harmanpreet; McDuff, Daniel; Williams, Alex C.; Teevan, Jaime; Iqbal, Shamsi T.	2022					10.1145/3491102.3517453	Kaur 2022	Non-Gastro/Hepatology Focus
Polls, clickbait, and commemorative 2 bills: Problematic political advertising on news and media websites around the 2020 U.S. elections	Zeng, Eric; Wei, Miranda; Gregersen, Theo; Kohno, Tadayoshi; Roesner, Franziska	2021				507-525	10.1145/3487552.3487850	Zeng 2021	Non-Gastro/Hepatology Focus
CSCW '17: Proceedings of the 2017 ACM conference on computer		2017							Non-NLP Focus

supported cooperative work and social computing									
Computational prediction of protein complexes from protein interaction networks	Srihari, Sriganesh; Yong, Chern Han; Wong, Limsoon	2017		16				Srihari 2017	Non-Gastro/Hepatology Focus
The handbook on socially interactive agents: 20 years of research on embodied conversational agents, intelligent virtual agents, and social robotics volume 2: Interactivity, platforms, application		2022		48					Non-Gastro/Hepatology Focus
Parting the red sea: Sociotechnical systems and lived experiences of menopause	Lazar, Amanda; Su, Norman Makoto; Bardzell, Jeffrey; Bardzell, Shaowen	2019				Jan-16	10.1145/3290605.3300710	Lazar 2019	Non-Gastro/Hepatology Focus
Differential query semantic analysis: Discovery of explicit interpretable knowledge from E-com search logs	Labhishetty, Sahiti; Zhai, ChengXiang; Xie, Min; Gong, Lin; Sharnagat, Rahul; Chembolu, Satya	2022				535-543	10.1145/3488560.3498503	Labhishetty 2022	Non-Gastro/Hepatology Focus
FashionQ: An AI-Driven creativity support tool for facilitating ideation in fashion design	Jeon, Youngseung; Jin, Seungwan; Shih, Patrick C.; Han, Kyungsik	2021					10.1145/3411764.3445093	Jeon 2021	Non-Gastro/Hepatology Focus
Auto-EM: End-to-end fuzzy entity-matching using pre-trained deep	Zhao, Chen; He, Yeye	2019				2413-2424	10.1145/3308558.3313578	Zhao 2019	Non-Gastro/Hepatology Focus

models and transfer learning									
Applied affective computing	Tian, Leimin; Oviatt, Sharon; Muszynski, Michal; Chamberlain, Brent C.; Healey, Jennifer; Sano, Akane	2022		41				Tian 2022	Non-NLP Focus
ASSETS '22: Proceedings of the 24th international ACM SIGACCESS conference on computers and accessibility		2022							Non-Gastro/Hepatology Focus
The handbook of multimodal-multisensor interfaces: Signal processing, architectures, and detection of emotion and cognition - volume 2		2018		21					Non-Gastro/Hepatology Focus
Monadic decomposition	Veanes, Margus; BjÃ¸rner, Nikolaj; Nachmanson, Lev; Bereg, Sergey	2017	J. ACM	64	2		10.1145/3040488	Veanes 2017	Non-Gastro/Hepatology Focus
TransNets: Learning to transform for recommendation	Catherine, Rose; Cohen, William	2017				288-296	10.1145/3109859.3109878	Catherine 2017	Non-Gastro/Hepatology Focus
Addressing complex and subjective product-related queries with customer reviews	McAuley, Julian; Yang, Alex	2016				625-635	10.1145/2872427.2883044	McAuley 2016	Non-NLP Focus
Roles in the discussion: An analysis of social	Johnson, Jazette; Black,	2020	Proc. ACM Hum.-Comput. Interact.	4	CSCW2		10.1145/3415198	Johnson 2020	Non-Gastro/Hepatology Focus

support in an online forum for people with dementia	Rebecca W.; Hayes, Gillian R.								
DataPrism: Exposing disconnect between data and systems	Galhotra, Sainyam; Fariha, Anna; LourenÃ§o, Raoni; Freire, Juliana; Meliou, Alexandra; Srivastava, Divesh	2022				217-231	10.1145/3514221.3517864	Galhotra 2022	Non-Gastro/Hepatology Focus
CUI '22: Proceedings of the 4th conference on conversational user interfaces		2022							Non-Gastro/Hepatology Focus
CSCW'22 companion: Companion publication of the 2022 conference on computer supported cooperative work and social computing		2022							Non-Gastro/Hepatology Focus
Experience selection in deep reinforcement learning for control	De Bruin, Tim; Kober, Jens; Tuyls, Karl; BabuÅ¡ka, Robert	2018	J. Mach. Learn. Res.	19	1	347-402		DeBruin 2018	Non-Gastro/Hepatology Focus
SA '17: SIGGRAPH asia 2017 courses		2017							Non-Gastro/Hepatology Focus
CHI PLAY '22: Extended abstracts of the 2022 annual symposium on computer-human interaction in play		2022							Non-Gastro/Hepatology Focus
ICMI '16: Proceedings of the 18th ACM international conference on multimodal interaction		2016							Non-Gastro/Hepatology Focus
SC '22: Proceedings of the international conference on high performance computing, networking, storage and analysis		2022							Non-Gastro/Hepatology Focus

CSSE '22: Proceedings of the 5th international conference on computer science and software engineering		2022							Non-Gastro/Hepatology Focus
SIGSPATIAL '22: Proceedings of the 30th international conference on advances in geographic information systems		2022							Non-Gastro/Hepatology Focus
NordiCHI '22: Adjunct proceedings of the 2022 nordic human-computer interaction conference		2022							Non-Gastro/Hepatology Focus
1974â€“1986		2019	Computing and the national science foundation, 1950â€“2016: Building a foundation for modern computing						Non-Gastro/Hepatology Focus
ICMI '22: Proceedings of the 2022 international conference on multimodal interaction		2022							Non-Gastro/Hepatology Focus
ICBIP '22: Proceedings of the 7th international conference on biomedical signal and image processing		2022							Non-Gastro/Hepatology Focus
Voice-controlled clinical coding companion (VC4) for ICD-10-AM and ACHI code assignment	Kaur, Rajvir; Anupama Ginige, Jeewani	2020					10.1145/3373017.3373060	Kaur 2020	Non-Gastro/Hepatology Focus
Relational path mining in structured knowledge	Bornea, Mihaela; Barker, Ken	2015					10.1145/2815833.2815840	Bornea 2015	Non-Gastro/Hepatology Focus
Mask and reason: Pre-training knowledge graph transformers for complex logical queries	Liu, Xiao; Zhao, Shiyu; Su, Kai; Cen, Yukuo; Qiu, Jiezhong; Zhang, Mengdi; Wu, Wei; Dong, Yuxiao; Tang, Jie	2022				1120-1130	10.1145/3534678.3539472	Liu 2022	Non-Gastro/Hepatology Focus
WWW '19: The world wide web conference		2019							Non-Gastro/Hepatology Focus

Application of artificial intelligence technology in the field of traditional chinese medicine	Chen, Zhencai; Li, Qi	2022				518-521	10.1145/3570773.3570847	Chen 2022	Review/Perspective Article Only
ReBoc: Accelerating block-circulant neural networks in ReRAM	Wang, Yitu; Chen, Fan; Song, Linghao; Shi, C.-J. Richard; Li, Hai "Helen"; Chen, Yiran	2020				1472-1477		Wang 2020	Non-Gastro/Hepatology Focus
Cross-corpora unsupervised learning of trajectories in autism spectrum disorders	Elibol, Huseyin Melih; Nguyen, Vincent; Linderman, Scott; Johnson, Matthew; Hashmi, Amna; Doshi-Velez, Finale	2016	J. Mach. Learn. Res.	17	1	4597-4634		Elibol 2016	Non-Gastro/Hepatology Focus
EnergyVis: Interactively tracking and exploring energy consumption for ML models	Shaikh, Omar; Saad-Falcon, Jon; Wright, Austin P; Das, Nilaksh; Freitas, Scott; Asensio, Omar; Chau, Duen Horng	2021					10.1145/3411763.3451780	Shaikh 2021	Non-Gastro/Hepatology Focus
Optimizing sparse tensor times matrix on multi-core and many-core architectures	Li, Jiajia; Ma, Yuchen; Yan, Chenggang; Vuduc, Richard	2016				26-33		Li 2016	Non-Gastro/Hepatology Focus
Feature-level domain adaptation	Kouw, Wouter M.; Van Der Maaten, Laurens J. P.;	2016	J. Mach. Learn. Res.	17	1	5943-5974		Kouw 2016	Non-Gastro/Hepatology Focus

	Krijthe, Jesse H.; Loog, Marco								
Learning to estimate external forces of human motion in video	Louis, Nathan; Corso, Jason J.; Templin, Tylan N.; Eliason, Travis D.; Nicolella, Daniel P.	2022				3540-3548	10.1145/3503161.3548377	Louis 2022	Non-Gastro/Hepatology Focus
Modeling 3D worlds: Outdoor	Aliaga, Daniel	2017					10.1145/3134472.3134493	Aliaga 2017	Non-Gastro/Hepatology Focus
MedRetriever: Target-driven interpretable health risk prediction via retrieving unstructured medical text	Ye, Muchao; Cui, Suhan; Wang, Yaqing; Luo, Junyu; Xiao, Cao; Ma, Fenglong	2021				2414-2423	10.1145/3459637.3482273	Ye 2021	Non-Gastro/Hepatology Focus
Norms matter: Contrasting social support around behavior change in online weight loss communities	Chancellor, Stevie; Hu, Andrea; De Choudhury, Munmun	2018				Jan-14	10.1145/3173574.3174240	Chancellor 2018	Non-Gastro/Hepatology Focus
Split computing and early exiting for deep learning applications: Survey and research challenges	Matsubara, Yoshitomo; Levorato, Marco; Restuccia, Francesco	2022	ACM Comput. Surv.	55	5		10.1145/3527155	Matsubara 2022	Non-Gastro/Hepatology Focus
Towards triggering higher-order thinking behaviors in MOOCs	Wang, Xu; Wen, Miaomiao; RosÃ©, Carolyn P.	2016				398-407	10.1145/2883851.2883964	Wang 2016	Non-Gastro/Hepatology Focus
Automatic differentiation in	Baydin, AtÄ±lÄ±m GÃ¼nes;	2017	J. Mach. Learn. Res.	18	1	5595-5637		Baydin 2017	Non-Gastro/Hepatology Focus

machine learning: A survey	Pearlmutter, Barak A.; Radul, Alexey Andreyevich; Siskind, Jeffrey Mark								
Pairwise multi-class document classification for semantic relations between wikipedia articles	Ostendorff, Malte; Ruas, Terry; Schubotz, Moritz; Rehm, Georg; Gipp, Bela	2020				127-136	10.1145/3383583.3398525	Ostendorff 2020	Non-Gastro/Hepatology Focus
Machine learning based knowledge organization in online communities	Houda, Sekkal; Naila, Amrous; Samir, Bennani	2020					10.1145/3372938.3372965	Houda 2020	Non-Gastro/Hepatology Focus
FLOP: Federated learning on medical datasets using partial networks	Yang, Qian; Zhang, Jianyi; Hao, Weituo; Spell, Gregory P.; Carin, Lawrence	2021				3845-3853	10.1145/3447548.3467185	Yang 2021	Non-Gastro/Hepatology Focus
Contextualized keyword representations for multi-modal retinal image captioning	Huang, Jia-Hong; Wu, Ting-Wei; Worring, Marcel	2021				645-652	10.1145/3460426.3463667	Huang 2021	Non-Gastro/Hepatology Focus
Generating explainable abstractions for wikidata entities	Klein, Nicholas; Ilievski, Filip; Szekely, Pedro	2021				89-96	10.1145/3460210.3493580	Klein 2021	Non-Gastro/Hepatology Focus
The diversity of music recommender systems	Baracskay, Ian; Baracskay III, Donald J; Iqbal, Mehtab; Knijnenburg, Bart Piet	2022				97-100	10.1145/3490100.3516474	Baracskay 2022	Non-Gastro/Hepatology Focus

WWW '22: Companion proceedings of the web conference 2022		2022							Non-Gastro/Hepatology Focus
How AI is driving the esports boom	Kugler, Logan	2022	Commun. ACM	65	9	17-18	10.1145/3546956	Kugler 2022	Non-Gastro/Hepatology Focus
Deep unsupervised cardinality estimation	Yang, Zongheng; Liang, Eric; Kamsetty, Amog; Wu, Chenggang; Duan, Yan; Chen, Xi; Abbeel, Pieter; Hellerstein, Joseph M.; Krishnan, Sanjay; Stoica, Ion	2019	Proc. VLDB Endow.	13	3	279-292	10.14778/3368289.3368294	Yang 2019	Non-Gastro/Hepatology Focus
Algorithmic differentiation of numerical methods: Tangent and adjoint solvers for parameterized systems of nonlinear equations	Naumann, Uwe; Lotz, Johannes; Leppkes, Klaus; Towara, Markus	2015	ACM Trans. Math. Softw.	41	4		10.1145/2700820	Naumann 2015	Non-Gastro/Hepatology Focus
Advancing the frontier of data-driven healthcare	Razavian, Narges	2015	XRDS	21	4	34-37	10.1145/2788506	Razavian 2015	Review/Perspective Article Only
Learning effective representations for person-job fit by feature fusion	Jiang, Junshu; Ye, Songyun; Wang, Wei; Xu, Jingran; Luo, Xiaosheng	2020				2549-2556	10.1145/3340531.3412717	Jiang 2020	Non-Gastro/Hepatology Focus
Generators that read	Kreminski, Max; Karth, Isaac;	2019					10.1145/3337722.3341849	Kreminski 2019	Non-Gastro/Hepatology Focus

	Wardrip-Fruin, Noah								
DeepRemaster: Temporal source-reference attention networks for comprehensive video enhancement	Iizuka, Satoshi; Simo-Serra, Edgar	2019	ACM Trans. Graph.	38	6		10.1145/3355089.3356570	Iizuka 2019	Non-Gastro/Hepatology Focus
How do users perceive multimodal expressions of affects?	Martin, Jean-Claude; Clavel, CÃ©line; Courgeon, Matthieu; Ammi, Mehdi; Amorim, Michel-Ange; Tsalamlal, Yacine; Gaffary, Yoren	2018	The handbook of multimodal-multisensor interfaces: Signal processing, architectures, and detection of emotion and cognition - volume 2			263-285		Martin 2018	Non-Gastro/Hepatology Focus
Multimodal-multisensor affect detection	D'Mello, Sidney K.; Bosch, Nigel; Chen, Huili	2018	The handbook of multimodal-multisensor interfaces: Signal processing, architectures, and detection of emotion and cognition - volume 2			167-202		D'Mello 2018	Non-Gastro/Hepatology Focus
ICCIR '22: Proceedings of the 2022 2nd international conference on control and intelligent robotics		2022							Non-Gastro/Hepatology Focus
Automatic identification and precise attribution of DRAM bandwidth contention	Helm, Christian; Taura, Kenjiro	2020					10.1145/3404397.3404422	Helm 2020	Non-Gastro/Hepatology Focus
Model slicing for supporting complex analytics with elastic inference cost and	Cai, Shaofeng; Chen, Gang; Ooi, Beng Chin; Gao, Jinyang	2019	Proc. VLDB Endow.	13	2	86-99	10.14778/3364324.3364325	Cai 2019	Non-Gastro/Hepatology Focus

resource constraints									
Pain level modeling of intensive care unit patients with machine learning methods: An effective congeneric clustering-based approach	Fang, Ruijie; Zhang, Ruoyu; Hosseini, Sayed M.; Faghih, Mahya; Rafatirad, Soheil; Rafatirad, Setareh; Homayoun, Houman	2022				89-95	10.1145/3524086.3524100	Fang 2022	Non-Gastro/Hepatology Focus
Patients reactions to non-invasive and invasive prenatal tests: A machine-based analysis from reddit posts	Delnevo, Giovanni; Mirri, Silvia; Monti, Lorenzo; Prandi, Catia; Putra, Manesha; Roccetti, Marco; Salomoni, Paola; Sokol, Robert J.	2020				980-987		Delnevo 2020	Non-Gastro/Hepatology Focus
Decomposing convolutional neural networks into reusable and replaceable modules	Pan, Rangeet; Rajan, Hridesh	2022				524-535	10.1145/3510003.3510051	Pan 2022	Non-Gastro/Hepatology Focus
CHI '21: Proceedings of the 2021 CHI conference on human factors in computing systems		2021							Non-Gastro/Hepatology Focus
A survey of naturalistic programming technologies	Pulido-Prieto, Oscar; JuÃ¡rez-MartÃnez, Ulises	2017	ACM Comput. Surv.	50	5		10.1145/3109481	Pulido-Prieto 2017	Non-Gastro/Hepatology Focus
TacTok: Semantics-aware proof synthesis	First, Emily; Brun, Yuriy; Guha, Arjun	2020	Proc. ACM Program. Lang.	4	OOPSLA		10.1145/3428299	First 2020	Non-Gastro/Hepatology Focus

Representation learning for dynamic graphs: A survey	Kazemi, Seyed Mehran; Goel, Rishab; Jain, Kshitij; Kobyzev, Ivan; Sethi, Akshay; Forsyth, Peter; Poupart, Pascal	2022	J. Mach. Learn. Res.	21	1			Kazemi 2022	Non-Gastro/Hepatology Focus
Open information extraction with meta-pattern discovery in biomedical literature	Wang, Xuan; Zhang, Yu; Li, Qi; Chen, Yinyin; Han, Jiawei	2018				291-300	10.1145/3233547.3233594	Wang 2018	Non-Gastro/Hepatology Focus
Implicit self-regularization in deep neural networks: Evidence from random matrix theory and implications for learning	Martin, Charles H.; Mahoney, Michael W.	2022	J. Mach. Learn. Res.	22	1			Martin 2022	Non-Gastro/Hepatology Focus
Theories of conversation for conversational IR	Thomas, Paul; Czerwinksi, Mary; Mcduff, Daniel; Craswell, Nick	2021	ACM Trans. Inf. Syst.	39	4		10.1145/3439869	Thomas 2021	Non-Gastro/Hepatology Focus
ITCC '22: Proceedings of the 4th international conference on information technology and computer communications		2022							Non-Gastro/Hepatology Focus
The ICARUS ontology: A general aviation ontology developed using a multi-layer approach	Stefanidis, Dimosthenis; Christodoulou, Chrysovalantis; Symeonidis, Moysis; Pallis, George; Dikaiakos,	2020				21-32	10.1145/3405962.3405983	Stefanidis 2020	Non-Gastro/Hepatology Focus

	Marios; Pouis, Loukas; Orphanou, Kalia; Lampathaki, Fenareti; Alexandrou, Dimitrios								
Data sets, modeling, and decision making in smart cities: A survey	Ma, Meiyi; Preum, Sarah M.; Ahmed, Mohsin Y.; TÃ¤rneberg, William; Hendawi, Abdeltawab; Stankovic, John A.	2019	ACM Trans. Cyber-Phys. Syst.	4	2		10.1145/3355283	Ma 2019	Non-Gastro/Hepatology Focus
Evaluation gaps in machine learning practice	Hutchinson, Ben; Rostamzadeh, Negar; Greer, Christina; Heller, Katherine; Prabhakaran, Vinodkumar	2022				1859-1876	10.1145/3531146.3533233	Hutchinson 2022	Non-Gastro/Hepatology Focus
EHR coding with multi-scale feature attention and structured knowledge graph propagation	Xie, Xiancheng; Xiong, Yun; Yu, Philip S.; Zhu, Yangyong	2019				649-658	10.1145/3357384.3357897	Xie 2019	Non-Gastro/Hepatology Focus
Mirror ritual: An affective interface for emotional self-reflection	Rajcic, Nina; McCormack, Jon	2020				Jan-13	10.1145/3313831.3376625	Rajcic 2020	Non-Gastro/Hepatology Focus
Extracting gene-disease relations from text to	Thompson, Paul;	2017				180-189	10.1145/3079452.3079472	Thompson 2017	Non-Gastro/Hepatology Focus

support biomarker discovery	Ananiadou, Sophia								
Human behavior inspired machine reading comprehension	Zheng, Yukun; Mao, Jiaxin; Liu, Yiqun; Ye, Zixin; Zhang, Min; Ma, Shaoping	2019				425-434	10.1145/3331184.3331231	Zheng 2019	Non-Gastro/Hepatology Focus
Nationality classification using name embeddings	Ye, Junting; Han, Shuchu; Hu, Yifan; Coskun, Baris; Liu, Meizhu; Qin, Hong; Skiena, Steven	2017				1897-1906	10.1145/3132847.3133008	Ye 2017	Non-Gastro/Hepatology Focus
CausalTriad: Toward pseudo causal relation discovery and hypotheses generation from medical text data	Zhao, Sendong; Jiang, Meng; Liu, Ming; Qin, Bing; Liu, Ting	2018				184-193	10.1145/3233547.3233555	Zhao 2018	Non-Gastro/Hepatology Focus
Text nailing: An efficient human-in-the-loop text-processing method	Kartoun, Uri	2017	Interactions	24	6	44-49	10.1145/3139488	Kartoun 2017	Non-Gastro/Hepatology Focus
Online disease diagnosis with inductive heterogeneous graph convolutional networks	Wang, Zifeng; Wen, Rui; Chen, Xi; Cao, Shilei; Huang, Shao-Lun; Qian, Buyue; Zheng, Yefeng	2021				3349-3358	10.1145/3442381.3449795	Wang 2021	Non-Gastro/Hepatology Focus
Medical dialogue response generation with pivotal information recalling	Zhao, Yu; Li, Yunxin; Wu, Yuxiang; Hu, Baotian; Chen, Qingcai; Wang, Xiaolong; Ding,	2022				4763-4771	10.1145/3534678.3542674	Zhao 2022	Non-Gastro/Hepatology Focus

	Yuxin; Zhang, Min								
On infectious intestinal disease surveillance using social media content	Zou, Bin; Lampos, Vasileios; Gorton, Russell; Cox, Ingemar J.	2016				157-161	10.1145/2896338.2896372	Zou 2016	NLP used only as a study adjunct/enabler
Modeling clinical data from publications	Barhak, Jacob	2015				47-52		Barhak 2015	Non-Gastro/Hepatology Focus
LuckyFind: Leveraging surprise to improve user satisfaction and inspire curiosity in a recommender system	Niu, Xi; Al-Doulat, Ahmad	2021				163-172	10.1145/3406522.3446017	Niu 2021	Non-Gastro/Hepatology Focus
The man who had them all	Kartoun, Uri	2017	Interactions	24	4	22-23	10.1145/3096966	Kartoun 2017	Review/Perspective Article Only
ICCCM '22: Proceedings of the 10th international conference on computer and communications management		2022							Non-Gastro/Hepatology Focus
Predicting MeSH beyond MEDLINE	Kehoe, Adam K.; Torvik, Vetle I.; Ross, Matthew B.; Smalheiser, Neil R.	2017				49-56	10.1145/3057148.3057155	Kehoe 2017	Non-Gastro/Hepatology Focus
Hate speech detection using brazilian imageboards	Nascimento, Gabriel; Carvalho, Flavio; Cunha, Alexandre Martins da; Viana, Carlos Roberto; Guedes, Gustavo Paiva	2019				325-328	10.1145/3323503.3360619	Nascimento 2019	Non-Gastro/Hepatology Focus

Learning to score economic development from satellite imagery	Han, Sungwon; Ahn, Donghyun; Park, Sungwon; Yang, Jeasurk; Lee, Susang; Kim, Jihee; Yang, Hyunjoo; Park, Sangyoon; Cha, Meeyoung	2020				2970-2979	10.1145/3394486.3403347	Han 2020	Non-Gastro/Hepatology Focus
Empath: Understanding topic signals in large-scale text	Fast, Ethan; Chen, Binbin; Bernstein, Michael S.	2016				4647-4657	10.1145/2858036.2858535	Fast 2016	Non-Gastro/Hepatology Focus
Survey: Big data application in biomedical research	Bachiller, Yvonne; Busch, Peter; Kavakli, Manolya; Hamey, Len	2018				174-178	10.1145/3192975.3192986	Bachiller 2018	Review/Perspective Article Only
Phenotypical ontology driven framework for multi-task learning	Ghalwash, Mohamed; Yao, Zijun; Chakraporty, Prithwish; Codella, James; Sow, Daby	2021				183-192	10.1145/3450439.3451881	Ghalwash 2021	Non-Gastro/Hepatology Focus
Lexical complexity prediction: An overview	North, Kai; Zampieri, Marcos; Shardlow, Matthew	2022	ACM Comput. Surv.				10.1145/3557885	North 2022	Non-Gastro/Hepatology Focus
ISAIMS '22: Proceedings of the 3rd international symposium on artificial intelligence for medicine sciences		2022							Non-Gastro/Hepatology Focus
A structured approach to understanding recovery and relapse in AA	Zhang, Yue; Ramesh, Arti; Golbeck, Jennifer; Sridhar, Dhanya; Getoor, Lise	2018				1205-1214	10.1145/3178876.3186019	Zhang 2018	Non-NLP Focus

Understanding user needs in videogame moment retrieval	Anderson, Barrett R.; Smith, Adam M.	2019					10.1145/3337722.3337728	Anderson 2019	Non-Gastro/Hepatology Focus
Emotion bubbles: Emotional composition of online discourse before and after the COVID-19 outbreak	Zhunis, Assem; Lima, Gabriel; Song, Hyeonho; Han, Jiyoung; Cha, Meeyoung	2022				2603-2613	10.1145/3485447.3512132	Zhunis 2022	Non-Gastro/Hepatology Focus
Prediction of chronic kidney disease risk using multimodal data	Ma, Dongfang; Li, Ximin; Mou, Shenghong; Cheng, Zhiyuan; Yan, Xiaoqian; Lu, Ying; Yan, Ruijian; Cao, Shiyue	2021				20-25	10.1145/3456529.3456533	Ma 2021	Non-Gastro/Hepatology Focus
Incorporating explicit knowledge in pre-trained language models for passage re-ranking	Dong, Qian; Liu, Yiding; Cheng, Suqi; Wang, Shuaiqiang; Cheng, Zhicong; Niu, Shuzi; Yin, Dawei	2022				1490-1501	10.1145/3477495.3531997	Dong 2022	Non-Gastro/Hepatology Focus
VeriCCM: Improving the syntax and semantics of requirements models	Gaither, Danielle; Madala, Kaushik; Do, Hyunsook; Bryant, Barrett R.	2019				1881-1884	10.1145/3297280.3299745	Gaither 2019	Non-Gastro/Hepatology Focus
Scalable verification of GNN-Based job schedulers	Wu, Haoze; Barrett, Clark; Sharif, Mahmood; Narodytska, Nina; Singh, Gagandeep	2022	Proc. ACM Program. Lang.	6	OOPSLA2		10.1145/3563325	Wu 2022	Non-Gastro/Hepatology Focus

DATE: Dual attentive tree-aware embedding for customs fraud detection	Kim, Sundong; Tsai, Yu-Che; Singh, Karandeep; Choi, Yeonsoo; Ibok, Etim; Li, Cheng-Te; Cha, Meeyoung	2020				2880-2890	10.1145/3394486.3403339	Kim 2020	Non-Gastro/Hepatology Focus
Verifying deep-RL-driven systems	Kazak, Yafim; Barrett, Clark; Katz, Guy; Schapira, Michael	2019				83-89	10.1145/3341216.3342218	Kazak 2019	Non-Gastro/Hepatology Focus
High-risk prediction of cardiovascular diseases via attention-based deep neural networks	An, Ying; Huang, Nengjun; Chen, Xianlai; Wu, Fangxiang; Wang, Jianxin	2019	IEEE/ACM Trans. Comput. Biol. Bioinformatics	18	3	1093-1105	10.1109/TCBB.2019.2935059	An 2019	Non-Gastro/Hepatology Focus
Expanding SNOMED-CT through spanish drug summaries of product characteristics	Calleja, Pablo; GarcÃa-Castro, RaÃºl; Aguado-de-Cea, Guadalupe; GÃ³mez-PÃ©rez, AsunciÃ³n	2017					10.1145/3148011.3148028	Calleja 2017	Non-Gastro/Hepatology Focus
ICMHI '22: Proceedings of the 6th international conference on medical and health informatics		2022							Non-Gastro/Hepatology Focus
Understanding challenges in prehabilitation for patients with multiple chronic conditions	Zhu, Haining; Moffa, Zachary; Wang, Xiying; Abdullah, Saeed; Julaiti, Juxihong; Carroll, John	2018				138-147	10.1145/3240925.3240959	Zhu 2018	Non-Gastro/Hepatology Focus

ArcheGEO: Towards improving relevance of gene expression omnibus search results	Chua, Huey-Eng; Tucker-Kellogg, Lisa; Bhowmick, Sourav S	2022					10.1145/3535508.3545531	Chua 2022	Non-Gastro/Hepatology Focus
Temporal pointwise convolutional networks for length of stay prediction in the intensive care unit	Rocheteau, Emma; LiÃ², Pietro; Hyland, Stephanie	2021				58-68	10.1145/3450439.3451860	Rocheteau 2021	Non-Gastro/Hepatology Focus
Question tagging via graph-guided ranking	Zhang, Xiao; Liu, Meng; Yin, Jianhua; Ren, Zhaochun; Nie, Liqiang	2021	ACM Trans. Inf. Syst.	40	1		10.1145/3468270	Zhang 2021	Non-Gastro/Hepatology Focus
Unpacking the use of laboratory test results in an online health community throughout the medical care trajectory	Reynolds, Tera L.; Zhang, Jiawen; Zheng, Kai; Chen, Yunan	2022	Proc. ACM Hum.-Comput. Interact.	6	CSCW2		10.1145/3555086	Reynolds 2022	Non-Gastro/Hepatology Focus
4SDrug: Symptom-based set-to-set small and safe drug recommendation	Tan, Yanchao; Kong, Chengjun; Yu, Leisheng; Li, Pan; Chen, Chaochao; Zheng, Xiaolin; Hertzberg, Vicki S.; Yang, Carl	2022				3970-3980	10.1145/3534678.3539089	Tan 2022	Non-Gastro/Hepatology Focus
A systematic literature review of automated feedback generation for	Keuning, Hieke; Jeuring, Johan; Heeren, Bastiaan	2018	ACM Trans. Comput. Educ.	19	1		10.1145/3231711	Keuning 2018	Non-Gastro/Hepatology Focus

programming exercises									
EDarkFind: Unsupervised multi-view learning for sybil account detection	Kumar, Ramnath; Yadav, Shweta; Daniulaityte, Raminta; Lamy, Francois; Thirunarayan, Krishnaprasad; Lokala, Usha; Sheth, Amit	2020				1955-1965	10.1145/3366423.3380263	Kumar 2020	Non-Gastro/Hepatology Focus
Towards a systematic review of automated feedback generation for programming exercises	Keuning, Hieke; Jeuring, Johan; Heeren, Bastiaan	2016				41-46	10.1145/2899415.2899422	Keuning 2016	Non-Gastro/Hepatology Focus
Multi-stage machine learning model for hierarchical tie valence prediction	Singh, Karandeep; Lee, Seungeon; Labianca, Giuseppe (Joe); Fagan, Jesse Michael; Cha, Meeyoung	2023	ACM Trans. Knowl. Discov. Data				10.1145/3579096	Singh 2023	Non-Gastro/Hepatology Focus
A structured and linguistic approach to understanding recovery and relapse in AA	Bailey, Shawn; Zhang, Yue; Ramesh, Arti; Golbeck, Jennifer; Getoor, Lise	2020	ACM Trans. Web	15	1		10.1145/3423208	Bailey 2020	Non-NLP Focus
Effective transfer learning for identifying similar questions: Matching user questions to COVID-19 FAQs	McCreery, Clara H.; Katariya, Namit; Kannan, Anitha; Chablani, Manish;	2020				3458-3465	10.1145/3394486.3412861	McCreery 2020	Non-Gastro/Hepatology Focus

	Amatriain, Xavier								
Automated ICD-9 coding via A deep learning approach	Li, Min; Fei, Zhihui; Zeng, Min; Wu, Fang-Xiang; Li, Yaohang; Pan, Yi; Wang, Jianxin	2019	IEEE/ACM Trans. Comput. Biol. Bioinformatics	16	4	1193-1202	10.1109/TCBB.2018.2817488	Li 2019	Non-Gastro/Hepatology Focus
ACM notice of article removal: Deep learning based medical diagnosis system using multiple data sources - originally published in the ACM digital library on 29-Aug-2018	Xue, Qinghan; Chuah, Mooi Choo	2018				699-706	10.1145/3233547.3233730	Xue 2018	Non-NLP Focus
Firsthand opiates abuse on social media: Monitoring geospatial patterns of interest through a digital cohort	Balsamo, Duilio; Bajardi, Paolo; Panisson, AndrÃ©	2019				2572-2579	10.1145/3308558.3313634	Balsamo 2019	Non-Gastro/Hepatology Focus
Computer vision and natural language processing: Recent approaches in multimedia and robotics	Wiriyathammabhum, Peratham; Summers-Stay, Douglas; FermÃ¼ller, Cornelia; Aloimonos, Yiannis	2016	ACM Comput. Surv.	49	4		10.1145/3009906	Wiriyathammabhum 2016	Non-Gastro/Hepatology Focus
SocialNLPâ€™22: 10th international workshop on natural language	Li, Cheng-Te; Ku, Lun-Wei; Tsai, Yu-Che; Wang, Wei-Yao	2022				849-851	10.1145/3487553.3524876	Li 2022	Non-Gastro/Hepatology Focus

processing for social media									
Natural language processing for EHR-Based computational phenotyping	Zeng, Zexian; Deng, Yu; Li, Xiaoyu; Naumann, Tristan; Luo, Yuan	2019	IEEE/ACM Trans. Comput. Biol. Bioinformatics	16	1	139-153	10.1109/TCBB.2018.2849968	Zeng 2019	Non-Gastro/Hepatology Focus
Informal-to-formal word conversion for persian language using natural language processing techniques	Naemi, Amin; Mansourvar, Marjan; Naemi, Mostafa; Damirchilu, Bahman; Ebrahimi, Ali; Kock Wiil, Uffe	2021					10.1145/3468691.3468710	Naemi 2021	Non-Gastro/Hepatology Focus
Cost-efficient quality assurance of natural language processing tools through continuous monitoring with continuous integration	Schreiber, Marc; Kraft, Bodo; ZÃ¼ndorf, Albert	2016				46-52	10.1145/2897022.2897029	Schreiber 2016	Non-Gastro/Hepatology Focus
Offline versus online representation learning of documents using external knowledge	Tamine, Lynda; Soulier, Laure; Nguyen, Gia-Hung; Souf, Nathalie	2019	ACM Trans. Inf. Syst.	37	4		10.1145/3349527	Tamine 2019	Non-Gastro/Hepatology Focus
Write it like you see it: Detectable differences in clinical notes by race lead to differential model recommendations	Adam, Hammaad; Yang, Ming Ying; Cato, Kenrick; Baldini, Ioana; Senteio, Charles; Celi,	2022				Jul-21	10.1145/3514094.3534203	Adam 2022	Non-Gastro/Hepatology Focus

	Leo Anthony; Zeng, Jiaming; Singh, Moninder; Ghassemi, Marzyeh								
Sentiment analysis of twitter data about blockchain technology	Rocha, Rayana Souza; Saraiva, Lohanna Aires; de Castro, AngÃ©lica FÃ©lix; de Alencar Silva, PatrÃcio	2021					10.1145/3401895.3401913	Rocha 2021	Non-Gastro/Hepatology Focus
Retrieving videogame moments with natural language queries	Zhang, Xiaoxuan; Smith, Adam M.	2019					10.1145/3337722.3341867	Zhang 2019	Non-Gastro/Hepatology Focus
Interleaved Text/Image deep mining on a large-scale radiology database for automated image interpretation	Shin, Hoo-Chang; Lu, Le; Kim, Lauren; Seff, Ari; Yao, Jianhua; Summers, Ronald M.	2016	J. Mach. Learn. Res.	17	1	3729-3759		Shin 2016	Non-Gastro/Hepatology Focus
Quantifying self-reported adverse drug events on twitter: Signal and topic analysis	Plachouras, Vassilis; Leidner, Jochen L.; Garrow, Andrew G.	2016					10.1145/2930971.2930977	Plachouras 2016	Non-Gastro/Hepatology Focus
Interpretable bias mitigation for textual data: Reducing genderization in patient notes while maintaining classification performance	Minot, Joshua R.; Cheney, Nicholas; Maier, Marc; Elbers, Danne C.; Danforth, Christopher M.; Dodds, Peter Sheridan	2022	ACM Trans. Comput. Healthcare	3	4		10.1145/3524887	Minot 2022	Non-Gastro/Hepatology Focus

Semi-supervised approach to monitoring clinical depressive symptoms in social media	Yazdavar, Amir Hossein; Al-Olimat, Hussein S.; Ebrahimi, Monireh; Bajaj, Goonmeet; Banerjee, Tanvi; Thirunarayan, Krishnaprasad; Pathak, Jyotishman; Sheth, Amit	2017				1191-1198	10.1145/3110025.3123028	Yazdavar 2017	Non-Gastro/Hepatology Focus
Exploiting augmented intelligence in the modeling of safety-critical autonomous systems	Yang, Zhibin; Bao, Yang; Yang, Yongqiang; Huang, Zhiqiu; Bodeveix, Jean-Paul; Filali, Mamoun; Gu, Zonghua	2021	Form. Asp. Comput.	33	3	343-384	10.1007/s00165-021-00543-6	Yang 2021	Non-Gastro/Hepatology Focus
Extracting medical entities from social media	Scepanovic, Sanja; Martin-Lopez, Enrique; Quercia, Daniele; Baykaner, Khan	2020				170-181	10.1145/3368555.3384467	Scepanovic 2020	Non-Gastro/Hepatology Focus
TrollHunter2020: Real-time detection of trolling narratives on twitter during the 2020 U.S. elections	Jachim, Peter; Sharevski, Filipo; Pieroni, Emma	2021				55-65	10.1145/3445970.3451158	Jachim 2021	Non-Gastro/Hepatology Focus
Multimodal fusion of smart home and text-based behavior markers for clinical	Sprint, Gina; Cook, Diane J.; Schmitter-Edgecombe, Maureen;	2022	ACM Trans. Comput. Healthcare	3	4		10.1145/3531231	Sprint 2022	Non-Gastro/Hepatology Focus

assessment prediction	Holder, Lawrence B.								
Processing affect in social media: A comparison of methods to distinguish emotions in tweets	Meo, Rosa; Sulis, Emilio	2017	ACM Trans. Internet Technol.	17	1		10.1145/2996187	Meo 2017	Non-Gastro/Hepatology Focus
Deep-confidentiality: An IoT-Enabled privacy-preserving framework for unstructured big biomedical data	Moqurrab, Syed Atif; Anjum, Adeel; Khan, Abid; Ahmed, Mansoor; Ahmad, Awais; Jeon, Gwanggil	2021	ACM Trans. Internet Technol.	22	2		10.1145/3421509	Moqurrab 2021	Non-Gastro/Hepatology Focus
Attention-based unsupervised keyphrase extraction and phrase graph for COVID-19 medical literature retrieval	Ding, Haoran; Luo, Xiao	2021	ACM Trans. Comput. Healthcare	3	1		10.1145/3473939	Ding 2021	Non-Gastro/Hepatology Focus
CognitiveEMS: A cognitive assistant system for emergency medical services	Preum, Sarah; Shu, Sile; Hotaki, Mustafa; Williams, Ronald; Stankovic, John; Alemzadeh, Homa	2019	SIGBED Rev.	16	2	51-60	10.1145/3357495.3357502	Preum 2019	Non-Gastro/Hepatology Focus
Distinguishing fake and real news of twitter data with the help of machine learning techniques	Passi, Kalpdrum; Shah, Aanan	2022				01-Aug	10.1145/3548785.3548811	Passi 2022	Non-Gastro/Hepatology Focus

Detecting emergent intersectional biases: Contextualized word embeddings contain a distribution of human-like biases	Guo, Wei; Caliskan, Aylin	2021				122-133	10.1145/3461702.3462536	Guo 2021	Non-Gastro/Hepatology Focus
Automated question-answer medical model based on deep learning technology	Abdallah, Abdelrahman; Kasem, Mahmoud; Hamada, Mohamed A.; Sdeek, Shaymaa	2020					10.1145/3410352.3410744	Abdallah 2020	Non-Gastro/Hepatology Focus
Neural character-level syntactic parsing for chinese	Li, Zuchao; Zhou, Junru; Zhao, Hai; Zhang, Zhisong; Li, Haonan; Ju, Yuqi	2022	J. Artif. Int. Res.	73			10.1613/jair.1.13052	Li 2022	Non-Gastro/Hepatology Focus
Generation theory in HR practice: Text mining for talent management case	Nikitinsky, Nikita; Kachurina, Polina; Sergey, Shashev; Shamis, Evgeniya	2016				262-266	10.1145/3014087.3014126	Nikitinsky 2016	Non-Gastro/Hepatology Focus
Investigating comorbidity of mental and physical disorders in online health forums	Abdollahyan, Maryam; Smeraldi, Fabrizio; Patel, Rashmi; Bessant, Conrad	2020					10.1145/3378184.3378195	Abdollahyan 2020	Non-Gastro/Hepatology Focus
Systematic review of machine learning approaches for detecting	Barrett, Liam; Hu, Junchao; Howell, Peter	2022	IEEE/ACM Trans. Audio, Speech and Lang. Proc.	30		1160-1172	10.1109/TASLP.2022.3155295	Barrett 2022	Non-Gastro/Hepatology Focus

developmental stuttering									
ROSE-NER: Robust semi-supervised named entity recognition on insufficient labeled data	Chen, Haiyan; Yuan, Shuwei; Zhang, Xiang	2022				38-44	10.1145/3502223.3502228	Chen 2022	Non-Gastro/Hepatology Focus
Unsupervised classification of health content on reddit	Barros, Joana M.; Buitelaar, Paul; Duggan, Jim; Rebholz-Schuhmann, Dietrich	2019				85-89	10.1145/3357729.3357745	Barros 2019	Non-Gastro/Hepatology Focus
Preordering using a target-language parser via cross-language syntactic projection for statistical machine translation	Goto, Isao; Utiyama, Masao; Sumita, Eiichiro; Kurohashi, Sadao	2015	ACM Trans. Asian Low-Resour. Lang. Inf. Process.	14	3		10.1145/2699925	Goto 2015	Non-Gastro/Hepatology Focus
Data-driven sentence simplification: Survey and benchmark	Alva-Manchego, Fernando; Scarton, Carolina; Specia, Lucia	2020	Comput. Linguist.	46	1	135-187	10.1162/coli_a_00370	Alva-Manchego 2020	Non-Gastro/Hepatology Focus
Knowledge-based textual inference via parse-tree transformations	Bar-Haim, Roy; Dagan, Ido; Berant, Jonathan	2015	J. Artif. Int. Res.	54	1	Jan-57		Bar-Haim 2015	Non-Gastro/Hepatology Focus
Assessing ICD-9-CM and ICPC-2 use in primary care. An italian case study	Cardillo, Elena; Chiaravalloti, Maria Teresa; Pasceri, Erika	2015				95-102	10.1145/2750511.2750525	Cardillo 2015	Non-Gastro/Hepatology Focus
Construction of knowledge graph of spleen and stomach diseases in traditional	Li, Can; Lin, Feng; Xie, Dan	2022				294-301	10.1145/3570773.3570865	Li 2022	Non-Gastro/Hepatology Focus

chinese medicine based on neo4j									
Supporting personalized health care with social media analytics: An application to hypothyroidism	Grani, Giorgio; Lenzi, Andrea; Velardi, Paola	2021	ACM Trans. Comput. Healthcare	3	1		10.1145/3468781	Grani 2021	Non-Gastro/Hepatology Focus
Modelling dialogues in court using a gradual argumentation model: A case study	Wei, Bin; Huang, JinHua	2015				138-147	10.1145/2746090.2746104	Wei 2015	Non-Gastro/Hepatology Focus
A compositional framework for grounding language inference, generation, and acquisition in video	Yu, Haonan; Siddharth, N.; Barbu, Andrei; Siskind, Jeffrey Mark	2015	J. Artif. Int. Res.	52	1	601-713		Yu 2015	Non-Gastro/Hepatology Focus
Usability guidelines and evaluation criteria for conversational user interfaces: A heuristic and linguistic approach	Sugisaki, Kyoko; Bleiker, Andreas	2020				309-319	10.1145/3404983.3405505	Sugisaki 2020	Non-Gastro/Hepatology Focus
Accelerating prototype-based drug discovery using conditional diversity networks	Harel, Shahar; Radinsky, Kira	2018				331-339	10.1145/3219819.3219882	Harel 2018	Non-Gastro/Hepatology Focus
A survey on computational metaphor processing	Rai, Sunny; Chakraverty, Shampa	2020	ACM Comput. Surv.	53	2		10.1145/3373265	Rai 2020	Non-Gastro/Hepatology Focus

End-to-end distantly supervised information extraction with retrieval augmentation	Zhang, Yue; Fei, Hongliang; Li, Ping	2022				2449-2455	10.1145/3477495.3531876	Zhang 2022	Non-Gastro/Hepatology Focus
Exploring collective tagging as a mechanism to elicit language about health management	Chen, Annie T.; Carriere, Rachel; Kaplan, Samantha J.; Colht, Kelly; Morey, Ophelia T.; Flaherty, Mary Grace; Moser, Gail B.; Slager, Stacey L.; Price, Cynthia	2016						Chen 2016	Non-Gastro/Hepatology Focus
Synthetic target domain supervision for open retrieval QA	Gangi Reddy, Revanth; Iyer, Bhavani; Sultan, Md Arafat; Zhang, Rong; Sil, Avirup; Castelli, Vittorio; Florian, Radu; Roukos, Salim	2021				1793-1797	10.1145/3404835.3463085	GangiReddy 2021	Non-Gastro/Hepatology Focus
Machine translation and legal tech in legal translation training	Muravev, Yury	2021					10.1145/3446434.3446553	Muravev 2021	Non-Gastro/Hepatology Focus
Enhancing feature selection using word embeddings: The case of flu surveillance	Lampos, Vasileios; Zou, Bin; Cox, Ingemar Johansson	2017				695-704	10.1145/3038912.3052622	Lampos 2017	Non-Gastro/Hepatology Focus

Making emergency calls more accessible to older adults through a hands-free speech interface in the house	Vacher, Michel; Aman, FrÃ©dÃ©ric; Rossato, Solange; Portet, FranÃ§ois; Lecouteux, Benjamin	2019	ACM Trans. Access. Comput.	12	2		10.1145/3310132	Vacher 2019	Non-Gastro/Hepatology Focus
Donâ€™t parse, generate! A sequence to sequence architecture for task-oriented semantic parsing	Rongali, Subendhu; Soldaini, Luca; Monti, Emilio; Hamza, Wael	2020				2962-2968	10.1145/3366423.3380064	Rongali 2020	Non-Gastro/Hepatology Focus
Unsupervised clinical language translation	Weng, Wei-Hung; Chung, Yu-An; Szolovits, Peter	2019				3121-3131	10.1145/3292500.3330710	Weng 2019	Non-Gastro/Hepatology Focus
Artificial Intelligence in Hepatology- Ready for the Primetime.	Kalapala, Rakesh; Rughwani, Hardik; Reddy, D. Nageshwar	2023	J Clin Exp Hepatol	13	1	149-161	10.1016/j.jceh.2022.06.009	Kalapala 2023	Review/Perspective Article Only
Pancreatic cancer-initiating cell exosome message transfer into noncancer-initiating cells: the importance of CD44v6 in reprogramming.	Wang, Zhe; Sun, Hanxue; Provaznik, Jan; Hackert, Thilo; ZÃ¶ller, Margot	2019	J Exp Clin Cancer Res	38	1	132	10.1186/s13046-019-1129-8	Wang 2019	NLP used only as a study adjunct/enabler
Drugs for preventing postoperative nausea and vomiting in adults after general anaesthesia: a	Weibel, Stephanie; RÃ¼cker, Gerta; Eberhart, Leopold Hj; Pace, Nathan L.; Hartl, Hannah	2020	Cochrane Database Syst Rev	10	10	CD012859	10.1002/14651858.CD012859.pub2	Weibel 2020	Non-Gastro/Hepatology Focus

network meta-analysis.	M.; Jordan, Olivia L.; Mayer, Debora; Riemer, Manuel; Schaefer, Maximilian S.; Raj, Diana; Backhaus, Insa; Helf, Antonia; Schlesinger, Tobias; Kienbaum, Peter; Kranke, Peter								
Deep Learning: A Primer for Radiologists.	Chartrand, Gabriel; Cheng, Phillip M.; Vorontsov, Eugene; Drozdzal, Michal; Turcotte, Simon; Pal, Christopher J.; Kadoury, Samuel; Tang, An	2017	Radiographics	37	7	2113-2131	10.1148/rg.2017170077	Chartrand 2017	Non-Gastro/Hepatology Focus
Artificial Intelligence in Plastic Surgery: Applications and Challenges.	Liang, Xuebing; Yang, Xiaoning; Yin, Shan; Malay, Sunitha; Chung, Kevin C.; Ma, Jiguang; Wang, Keming	2021	Aesthetic Plast Surg	45	2	784-790	10.1007/s00266-019-01592-2	Liang 2021	Non-Gastro/Hepatology Focus
Tumor Necrosis Factor Inhibition and Parkinson Disease: A Mendelian	Kang, Xiaoying; Ploner, Alexander; Pedersen, Nancy L.; Bandres-Ciga,	2021	Neurology	96	12	e1672-e1679	10.1212/WNL.0000000000011630	Kang 2021	Non-Gastro/Hepatology Focus

Randomization Study.	Sara; Noyce, Alastair J.; Wirdefeldt, Karin; Williams, Dylan M.								
Escape steering by cholecystokinin peptidergic signaling.	Chen, Lili; Liu, Yuting; Su, Pan; Hung, Wesley; Li, Haiwen; Wang, Ya; Yue, Zhongpu; Ge, Ming-Hai; Wu, Zheng-Xing; Zhang, Yan; Fei, Peng; Chen, Li-Ming; Tao, Louis; Mao, Heng; Zhen, Mei; Gao, Shangbang	2022	Cell Rep	38	6	110330	10.1016/j.celrep.2022.110330	Chen 2022	Non-Gastro/Hepatology Focus
The Use of Natural Language Processing to Assess Social Support in Patients With Advanced Cancer.	Bhatt, Sunil; Johnson, P. Connor; Markovitz, Netana H.; Gray, Tamryn; Nipp, Ryan D.; Ufere, Nneka; Rice, Julia; Reynolds, Matthew J.; Lavoie, Mitchell W.; Clay, Madison A.; Lindvall, Charlotta; El-Jawahri, Areej	2022	Oncologist			oyac238	10.1093/oncolo/oyac238	Bhatt 2022	Non-Gastro/Hepatology Focus
Capturing Surgical Data: Comparing a Quality	Miller, Benjamin T.; Fafaj, Aldo;	2022	J Gastrointest Surg	26	7	1490-1494	10.1007/s11605-022-05282-4	Miller 2022	Non-Gastro/Hepatology Focus

Improvement Registry to Natural Language Processing and Manual Chart Review.	Tastaldi, Luciano; Alkhatib, Hemasat; Zolin, Samuel; AlMarzooqi, Raha; Tu, Chao; Alaedeen, Diya; Prabhu, Ajita S.; Krpata, David M.; Rosen, Michael J.; Petro, Clayton C.								
The use of natural language processing of infusion notes to identify outpatient infusions.	Nelson, Scott D.; Lu, Chao-Chin; Teng, Chia-Chen; Leng, Jianwei; Cannon, Grant W.; He, Tao; Zeng, Qing; Halwani, Ahmad; Sauer, Brian	2015	Pharmacoepidemiol Drug Saf	24	1	86-92	10.1002/pds.3720	Nelson 2015	Non-Gastro/Hepatology Focus
A natural language processing pipeline to synthesize patient-generated notes toward improving remote care and chronic disease management: a cystic fibrosis case study.	Hussain, Syed-Amad; Sezgin, Emre; Krivchenia, Katelyn; Luna, John; Rust, Steve; Huang, Yungui	2021	JAMIA Open	4	3	ooab084	10.1093/jamiaopen/ooab084	Hussain 2021	Non-Gastro/Hepatology Focus
Validation of a Natural Language Processing Algorithm for	Hardjojo, Antony; Gunachandran, Arunan; Pang,	2018	JMIR Med Inform	6	2	e36	10.2196/medinform.8204	Hardjojo 2018	Non-Gastro/Hepatology Focus

Detecting Infectious Disease Symptoms in Primary Care Electronic Medical Records in Singapore.	Long; Abdullah, Mohammed Ridzwan Bin; Wah, Win; Chong, Joash Wen Chen; Goh, Ee Hui; Teo, Sok Huang; Lim, Gilbert; Lee, Mong Li; Hsu, Wynne; Lee, Vernon; Chen, Mark I.-Cheng; Wong, Franco; Phang, Jonathan Siung King								
Automated conversational agents for post-intervention follow-up: a systematic review.	Geoghegan, L.; Scarborough, A.; Wormald, J. C. R.; Harrison, C. J.; Collins, D.; Gardiner, M.; Bruce, J.; Rodrigues, J. N.	2021	BJS Open	5	4		10.1093/bjsopen/zrab070	Geoghegan 2021	Non-Gastro/Hepatology Focus
Evaluating the potential of artificial intelligence in ulcerative colitis.	Sinonquel, Pieter; SchilirÃ², Alessandro; Verstockt, Bram; Vermeire, SÃ©verine; Bisschops, Raf	2023	Expert Rev Gastroenterol Hepatol			01-Sep	10.1080/17474124.2023.2166928	Sinonquel 2023	Review/Perspective Article Only
Prevalence of Advanced Colorectal Neoplasia in Veterans: Effects of Age, Sex, and Race/Ethnicity.	Imperiale, Thomas F.; Daggy, Joanne K.; Imler, Timothy D.; Sherer, Eric A.; Kahi, Charles J.;	2021	J Clin Gastroenterol	55	10	876-883	10.1097/MCG.0000000000001402	Imperiale 2021	Non-NLP Focus

	Larson, Jason; Cardwell, Jon; Johnson, Cynthia S.; Ahnen, Dennis J.; Antaki, Fadi; Ashley, Christopher; Baffy, Gyorgy; Dominitz, Jason A.; Hou, Jason; Korsten, Mark A.; Nagar, Anil; Promrat, Kittichai; Robertson, Douglas J.; Saini, Sameer; Shergill, Amandeep; Smalley, Walter E.								
Promoting Research, Awareness, and Discussion on AI in Medicine Using #MedTwitterAI: A Longitudinal Twitter Hashtag Analysis.	Nawaz, Faisal A.; Barr, Austin A.; Desai, Monali Y.; Tsagkaris, Christos; Singh, Romil; Klager, Elisabeth; Eibensteiner, Fabian; Parvanov, Emil D.; Hribersek, Mojca; Kletecka-Pulker, Maria; Willschke, Harald;	2022	Front Public Health	10		856571	10.3389/fpubh.2022.856571	Nawaz 2022	Non-Gastro/Hepatology Focus

	Atanasov, Atanas G.								
Big Dreams With Big Data! Use of Clinical Informatics to Inform Biomarker Discovery.	Singh, Siddharth	2019	Clin Transl Gastroenterol	10	3	e00018	10.14309/ctg.0000000000000018	Singh 2019	Review/Perspective Article Only
Feature engineering for sentiment analysis in e-health forums.	Carrillo-de-Albornoz, Jorge; RodrÃguez Vidal, Javier; Plaza, Laura	2018	PLoS One	13	11	e0207996	10.1371/journal.pone.0207996	Carrillo-de-Albornoz 2018	Non-Gastro/Hepatology Focus
A Framework (SOCRATex) for Hierarchical Annotation of Unstructured Electronic Health Records and Integration Into a Standardized Medical Database: Development and Usability Study.	Park, Jimyung; You, Seng Chan; Jeong, Eugene; Weng, Chunhua; Park, Dongsu; Roh, Jin; Lee, Dong Yun; Cheong, Jae Youn; Choi, Jin Wook; Kang, Mira; Park, Rae Woong	2021	JMIR Med Inform	9	3	e23983	10.2196/23983	Park 2021	Non-Gastro/Hepatology Focus
Assessment of Prevalence of Adolescent Patient Portal Account Access by Guardians.	Ip, Wui; Yang, Samuel; Parker, Jacob; Powell, Austin; Xie, James; Morse, Keith; Aikens, Rachael C.; Lee, Jennifer; Gill, Manjot; Vundavalli, Shravani; Huang, Yungui; Huang, Jeannie; Chen, Jonathan H.; Hoffman,	2021	JAMA Netw Open	4	9	e2124733	10.1001/jamanetworkopen.2021.24733	Ip 2021	Non-Gastro/Hepatology Focus

	Jeffrey; Kuelbs, Cynthia; Pageler, Natalie								
Can artificial intelligence increase the efficiency in referrals from primary to specialized care?	Tejedor, Marta; Herrero, Antonio; Castresana, Carlos; MesÃ³n, RaÃºl; Taracido, Juan Carlos; SÃ¡nchez, Marta; Delgado, MarÃa	2022	Rev Esp Enferm Dig				10.17235/reed.2022.9020/2022	Tejedor 2022	NLP used only as a study adjunct/enabler
High-throughput phenotyping with electronic medical record data using a common semi-supervised approach (PheCAP).	Zhang, Yichi; Cai, Tianrun; Yu, Sheng; Cho, Kelly; Hong, Chuan; Sun, Jiehuan; Huang, Jie; Ho, Yuk-Lam; Ananthakrishnan, Ashwin N.; Xia, Zongqi; Shaw, Stanley Y.; Gainer, Vivian; Castro, Victor; Link, Nicholas; Honerlaw, Jacqueline; Huang, Sicong; Gagnon, David; Karlson, Elizabeth W.; Plenge, Robert M.; Szolovits, Peter; Savova, Guergana; Churchill,	2019	Nat Protoc	14	12	3426-3444	10.1038/s41596-019-0227-6	Zhang 2019	Non-Gastro/Hepatology Focus

	Susanne; O'Donnell, Christopher; Murphy, Shawn N.; Gaziano, J. Michael; Kohane, Isaac; Cai, Tianxi; Liao, Katherine P.								
Phenome-driven disease genetics prediction toward drug discovery.	Chen, Yang; Li, Li; Zhang, Guo-Qiang; Xu, Rong	2015	Bioinformatics	31	12	i276-283	10.1093/bioinformatics/btv245	Chen 2015	Non-Gastro/Hepatology Focus
Relation Classification for Bleeding Events From Electronic Health Records Using Deep Learning Systems: An Empirical Study.	Mitra, Avijit; Rawat, Bhanu Pratap Singh; McManus, David D.; Yu, Hong	2021	JMIR Med Inform	9	7	e27527	10.2196/27527	Mitra 2021	Non-Gastro/Hepatology Focus
The outcome of surgical and non-surgical treatments for traumatic optic neuropathy: a comparative study of 685 cases.	Wei, Wang; Zhao, Shang-Feng; Li, Yong; Zhang, Jia-Liang; Wu, Jiang-Ping; Liu, Hao-Cheng; Sun, Si; Song, Gui-Dong; Ma, Jian-Min; Kang, Jun	2022	Ann Transl Med	10	10	542	10.21037/atm-22-1836	Wei 2022	Non-Gastro/Hepatology Focus
Association of Social Support With Overall Survival and Healthcare Utilization in Patients With Aggressive	Johnson, P. Connor; Markovitz, Netana H.; Gray, Tamryn F.; Bhatt, Sunil; Nipp, Ryan D.; Ufere, Nneka;	2021	J Natl Compr Canc Netw			01-Jul	10.6004/jnccn.2021.7033	Johnson 2021	Non-Gastro/Hepatology Focus

Hematologic Malignancies.	Rice, Julia; Reynolds, Matthew J.; Lavoie, Mitchell W.; Topping, Carlisle E. W.; Clay, Madison A.; Lindvall, Charlotta; El-Jawahri, Areej								
Role of delayed wider endoscopic optic decompression for traumatic optic neuropathy: a single-center surgical experience.	Zhao, Shang-Feng; Yong, Li; Zhang, Jia-Liang; Wu, Jiang-Ping; Liu, Hao-Cheng; Sun, Si; Song, Gui-Dong; Ma, Jian-Min; Kang, Jun	2021	Ann Transl Med	9	2	136	10.21037/atm-20-7810	Zhao 2021	Non-Gastro/Hepatology Focus
Prevention of Evisceration or Enucleation in Endogenous Bacterial Panophthalmitis with No Light Perception and Scleral Abscess.	Chen, Kuan-Jen; Chen, Yen-Po; Chao, An-Ning; Wang, Nan-Kai; Wu, Wei-Chi; Lai, Chi-Chun; Chen, Tun-Lu	2017	PLoS One	12	1	e0169603	10.1371/journal.pone.0169603	Chen 2017	Non-NLP Focus
Drug distribution in nanostructured lipid particles.	Saeidpour, Siavash; Lohan, Silke B.; Solik, Agnieszka; Paul, Victoria; Bodmeier, Roland; Zoubari, Gaith; Unbehauen, Michael; Haag, Rainer; Bittl, Robert; Meinke,	2017	Eur J Pharm Biopharm	110		19-23	10.1016/j.ejpb.2016.10.008	Saeidpour 2017	Non-Gastro/Hepatology Focus

	Martina C.; Teutloff, Christian								
TFF2-CXCR4 Axis Is Associated with BRAF V600E Colon Cancer.	Gala, Manish K.; Austin, Thomas; Ogino, Shuji; Chan, Andrew T.	2015	Cancer Prev Res (Phila)	8	7	614-619	10.1158/1940-6207.CAPR-14-0444	Gala 2015	Non-NLP Focus
Factors associated with improved survival after resection of pancreatic adenocarcinoma: a multivariable model.	Call, Tyler R.; Pace, Nathan L.; Thorup, Datus B.; Maxfield, Derric; Chortkoff, Ben; Christensen, Joslin; Mulvihill, Sean J.	2015	Anesthesiology	122	2	317-324	10.1097/ALN.0000000000000489	Call 2015	Non-NLP Focus
Symptoms timeline and outcomes in amyotrophic lateral sclerosis using artificial intelligence.	Segura, TomÃ¡s; Medrano, Ignacio H.; Collazo, Sergio; MatÃ©, Claudia; Sguera, Carlo; Del Rio-Bermudez, Carlos; Casero, Hugo; Salcedo, Ignacio; GarcÃa-GarcÃa, Jorge; Alcahut-RodrÃ-guez, Cristian; Taberna, Miren	2023	Sci Rep	13	1	702	10.1038/s41598-023-27863-2	Segura 2023	Non-Gastro/Hepatology Focus
Use of electronic medical records in development and validation of risk prediction models of hospital readmission: systematic review.	Mahmoudi, Elham; Kamdar, Neil; Kim, Noa; Gonzales, Gabriella; Singh, Karandeep; Waljee, Akbar K.	2020	BMJ	369		m958	10.1136/bmj.m958	Mahmoudi 2020	Non-Gastro/Hepatology Focus

Genotype-Guided P2Y(12) Inhibitor Therapy After Percutaneous Coronary Intervention: A Bayesian Analysis.	Parcha, Vibhu; Heindl, Brittain F.; Li, Peng; Kalra, Rajat; Limdi, Nita A.; Pereira, Naveen L.; Arora, Garima; Arora, Pankaj	2021	Circ Genom Precis Med	14	6	e003353	10.1161/CIRCGEN.121.003353	Parcha 2021	Non-Gastro/Hepatology Focus
Prototyping a precision oncology 3.0 rapid learning platform.	Sweetnam, Connor; Mocellin, Simone; Krauthammer, Michael; Knopf, Nathaniel; Baertsch, Robert; Shrager, Jeff	2018	BMC Bioinformatics	19	1	341	10.1186/s12859-018-2374-0	Sweetnam 2018	Non-Gastro/Hepatology Focus
Iodine-125 plaque brachytherapy for diffuse choroidal hemangioma.	Azarcon, Corrina P.; Qiu, Richard L. J.; Sobol, Ethan K.; Hubbard, G. Baker 3rd; Craven, Caroline M.; Bergstrom, Chris S.; Wells, Jill R.	2022	Retin Cases Brief Rep				10.1097/ICB.0000000000001334	Azarcon 2022	Non-Gastro/Hepatology Focus
Risk Factors Analysis for the Outcome of Indirect Traumatic Optic Neuropathy With Steroid Pulse Therapy.	Lai, I.-Li; Liao, Han-Tsung; Chen, Chien-Tzung	2016	Ann Plast Surg	76 Suppl 1		S60-67	10.1097/SAP.0000000000000694	Lai 2016	Non-Gastro/Hepatology Focus
Transfer of Learning from Vision to Touch: A Hybrid Deep	Rouhafzay, Ghazal; Cretu, Ana-Maria; Payeur, Pierre	2020	Sensors (Basel)	21	1		10.3390/s21010113	Rouhafzay 2020	Non-Gastro/Hepatology Focus

Convolutional Neural Network for Visuo-Tactile 3D Object Recognition.									
Incidence of fever and bleeding after percutaneous nephrolithotomy: a prospective cohort study.	Sierra-Diaz, Erick; DÃ¡vila-Radilla, Fernando; Espejo-VÃ¡zquez, AnalÃa; Ruiz-Velasco, Carlos B.; Gaxiola-Perez, Efren; Rosa, Alfredo de JesÃºs Celis-De la	2022	Cir Cir	90	1	57-63	10.24875/CIRU.20001130	Sierra-Diaz 2022	Non-Gastro/Hepatology Focus
Machine Learning in the OR: A Collaborative Environment for Surgical Interventions in Visceral Medicine.	Ostler, Daniel; Wilhelm, Dirk; Bernhard, Lukas; Fuchtmann, Jonas; Kranzfelder, Michael; Vogel, Thomas; Feussner, Hubertus	2020	Surg Technol Int	37		16-21		Ostler 2020	Non-Gastro/Hepatology Focus
Extraction of the molecular level biomedical event trigger based on gene ontology using radial belief neural network techniques.	Devendra Kumar, R. N.; C, Arvind; Srihari, K.	2021	Biosystems	199		104313	10.1016/j.biosystems.2020.104313	DevendraKumar 2021	Non-NLP Focus
Beyond opinion classification: Extracting facts,	Carrillo-de-Albornoz, Jorge; Aker, Ahmet;	2019	PLoS One	14	1	e0209961	10.1371/journal.pone.0209961	Carrillo-de-Albornoz 2019	Non-Gastro/Hepatology Focus

opinions and experiences from health forums.	Kurtic, Emina; Plaza, Laura								
Analysis of Risk Factors for Adjacent Segment Degeneration after Minimally Invasive Transforaminal Interbody Fusion at Lumbosacral Spine.	Dong, Shengtao; Li, Jie; Jia, Xiaomeng; Zhu, Jieyang; Chen, Yaoning; Yuan, Bo	2022	Comput Intell Neurosci	2022		4745534	10.1155/2022/4745534	Dong 2022	Non-Gastro/Hepatology Focus
A prediction model with measured sentiment scores for the risk of in-hospital mortality in acute pancreatitis: a retrospective cohort study.	Liu, Zhanxiao; Yang, Ya; Song, Huanhuan; Luo, Ji	2022	Ann Transl Med	10	12	676	10.21037/atm-22-1613	Liu 2022	NLP used only as a study adjunct/enabler
Identifying patterns in administrative tasks through structural topic modeling: A study of task definitions, prevalence, and shifts in a mental health practice's operations during the COVID-19 pandemic.	Pachamanova, Dessislava; Glover, Wiljeana; Li, Zhi; Docktor, Michael; Gujral, Nitin	2021	J Am Med Inform Assoc	28	12	2707-2715	10.1093/jamia/ocab185	Pachamanova 2021	Non-Gastro/Hepatology Focus
ViMRT: a text-mining tool and search engine for automated virus	Tong, Yuantao; Tan, Fanglin; Huang, Honglian;	2023	Bioinformatics	39	1		10.1093/bioinformatics/btac721	Tong 2023	Non-Gastro/Hepatology Focus

mutation recognition.	Zhang, Zeyu; Zong, Hui; Xie, Yujia; Huang, Danqi; Cheng, Shiyang; Wei, Ziyi; Fang, Meng; Crabbe, M. James C.; Wang, Ying; Zhang, Xiaoyan								
Traumatic optic neuropathy treatment trial (TONTT): open label, phase 3, multicenter, semi-experimental trial.	Kashkouli, Mohsen Bahmani; Yousefi, Sahar; Nojomi, Marzieh; Sanjari, Mostafa Soltan; Pakdel, Farzad; Entezari, Morteza; Etezad-Razavi, Mohammad; Razeghinejad, Mohammad Reza; Esmaeli, Manuchehr; Shafiee, Masoud; Bagheri, Mansoureh	2018	Graefes Arch Clin Exp Ophthalmol	256	1	209-218	10.1007/s00417-017-3816-5	Kashkouli 2018	Non-Gastro/Hepatology Focus
Neovascular age-related macular degeneration associated with no light perception.	Brown, Gary C.; Basha, Mahdi M.; Brown, Melissa M.	2015	Ophthalmic Surg Lasers Imaging Retina	46	2	229-234	10.3928/23258160-20150213-03	Brown 2015	Non-Gastro/Hepatology Focus
Ultra-mini PNL (UMP): Material, indications, technique,	Desai, Janak D.	2017	Arch Esp Urol	70	1	196-201		Desai 2017	Non-Gastro/Hepatology Focus

advantages and results.									
Missed Incidental Pulmonary Embolism: Harnessing Artificial Intelligence to Assess Prevalence and Improve Quality Improvement Opportunities.	Wildman-Tobriner, Benjamin; Ngo, Lawrence; Mammarappallil, Joseph G.; Konkel, Brandon; Johnson, Jacob M.; Bashir, Mustafa R.	2021	J Am Coll Radiol	18	7	992-999	10.1016/j.jacr.2021.01.014	Wildman-Tobriner 2021	Non-Gastro/Hepatology Focus
Investigation of the cutaneous penetration behavior of dexamethasone loaded to nano-sized lipid particles by EPR spectroscopy, and confocal Raman and laser scanning microscopy.	Lohan, Silke B.; Saeidpour, Siavash; Solik, Agnieszka; Schanzer, Sabine; Richter, Heike; Dong, Pin; Darvin, Maxim E.; Bodmeier, Roland; Patzelt, Alexa; Zoubari, Gaith; Unbehauen, Michael; Haag, Rainer; Lademann, JÃ¼rgen; Teutloff, Christian; Bittl, Robert; Meinke, Martina C.	2017	Eur J Pharm Biopharm	116		102-110	10.1016/j.ejpb.2016.12.018	Lohan 2017	Non-Gastro/Hepatology Focus
[A 212 cases analysis of treatment for traumatic optic neuropathy by	Chen, M.; Jiang, Y.; Pang, W. H.; Li, N.; Niu, Y. Z.; Zhao, H.	2017	Lin Chung Er Bi Yan Hou Tou Jing Wai Ke Za Zhi	31	18	1411-1414	10.13201/j.issn.1001-1781.2017.18.008	Chen 2017	Non-Gastro/Hepatology Focus

nasal endoscopic opticnerve decompression].									
A real-world experience with the bioactive human split thickness skin allograft for venous leg ulcers.	Kirsner, Robert S.; Margolis, David; Masturzo, Arti; Bakewell, Katie	2020	Wound Repair Regen	28	4	547-552	10.1111/wrr.12818	Kirsner 2020	Non-Gastro/Hepatology Focus
Specific Disruption of Abca1 Targeting Largely Mimics the Effects of miR-33 Knockout on Macrophage Cholesterol Efflux and Atherosclerotic Plaque Development.	Price, Nathan L.; Rotllan, Noemi; Zhang, Xinbo; CanfrÃ¡n-Duque, Alberto; Nottoli, Timothy; Suarez, Yajaira; FernÃ¡ndez-Hernando, Carlos	2019	Circ Res	124	6	874-880	10.1161/CIRCRESAHA.118.314415	Price 2019	Non-Gastro/Hepatology Focus
Natural Language Processing of Computed Tomography Reports to Label Metastatic Phenotypes With Prognostic Significance in Patients With Colorectal Cancer.	Causa Andrieu, Pamela; Golia Pernicka, Jennifer S; Yaeger, Rona; Lupton, Kaelan; Batch, Karen; Zulkernine, Farhana; Simpson, Amber L; Taya, Michio; Gazit, Lior; Nguyen, Huy; Nicholas, Kevin; Gangai, Natalie; Sevilimedu, Varadan; Dickinson, Shannan;	2022	JCO Clin Cancer Inform	6	1.02E+08	e2200014	10.1200/CCI.22.00014	CausaAndrieu 2022	Non-NLP Focus

	Paroder, Viktoriya; Bates, David D B; Do, Richard								
Establishing a colorectal cancer research database from routinely collected health data: the process and potential from a pilot study.	Tamm, Andres; Jones, Helen Js; Perry, William; Campbell, Des; Carten, Rachel; Davies, Jim; Galdikas, Algirdas; English, Louise; Garbett, Alex; Glampson, Ben; Harris, Steve; Khan, Khurum; Little, Stephanie; Malcomson, Lee; Matharu, Sheila; Mayer, Erik; Mercuri, Luca; Morris, Eva Ja; Muirhead, Rebecca; Norris, Ruth; O'Hara, Catherine; Papadimitriou, Dimitri; Peek, Niels; Renehan, Andrew; Roadknight, Gail; Starling, Naureen; Teare, Marion; Turner, Rachel; Varnai, Kinga A; Wasan, Harpreet;	2022	BMJ Health Care Inform	29	1		10.1136/bmjhci-2021-100535	Tamm 2022	Weak Validation Only (Type 1b or less)

	Woods, Kerrie; Cunningham, Chris								
Natural Language Processing to Assess Frequency of Functional Status Documentation for Patients Newly Diagnosed With Colorectal Cancer.	Sun, Lin	2021	JAMA Oncol	7	3	462-463	10.1001/jamaoncol.2020.7615	Sun 2021	Review/Perspective Article Only
Natural Language Processing to Assess Frequency of Functional Status Documentation for Patients Newly Diagnosed With Colorectal Cancer-Reply.	Agaronnik, Nicole D; Lindvall, Charlotta; Iezzoni, Lisa I	2021	JAMA Oncol	7	3	463	10.1001/jamaoncol.2020.7621	Agaronnik 2021	Review/Perspective Article Only
Natural Language Processing to Assess Frequency of Functional Status Documentation for Patients Newly Diagnosed With Colorectal Cancer.	Ooi, Rucira; Ooi, Setthasorn Zhi Yang	2021	JAMA Oncol	7	3	461-462	10.1001/jamaoncol.2020.7609	Ooi 2021	Review/Perspective Article Only
Use of Natural Language Processing to Assess Frequency of Functional Status Documentation for Patients Newly	Agaronnik, Nicole; Lindvall, Charlotta; El-Jawahri, Areej; He, Wei; Iezzoni, Lisa	2020	JAMA Oncol	6	10	1628-1630	10.1001/jamaoncol.2020.2708	Agaronnik 2020	NLP used only as a study adjunct/enabler

Diagnosed With Colorectal Cancer.									
Challenges of Developing a Natural Language Processing Method With Electronic Health Records to Identify Persons With Chronic Mobility Disability.	Agaronnik, Nicole D; Lindvall, Charlotta; El-Jawahri, Areej; He, Wei; Iezzoni, Lisa I	2020	Arch Phys Med Rehabil	101	10	1739-1746	10.1016/j.apmr.2020.04.024	Agaronnik 2020	Non-Gastro/Hepatology Focus
Mapping Patient Data to Colorectal Cancer Clinical Algorithms for Personalized Guideline-Based Treatment.	Becker, Matthias; Bockmann, Britta; Jockel, Karl-Heinz; Stuschke, Martin; Paul, Andreas; Kasper, Stefan; Virchow, Isabel	2020	Appl Clin Inform	11	2	200-209	10.1055/s-0040-1705105	Becker 2020	Non-NLP Focus
Knowledge-based best of breed approach for automated detection of clinical events based on German free text digital hospital discharge letters.	Konig, Maximilian; Sander, Andre; Demuth, Ilja; Diekmann, Daniel; Steinhagen-Thiessen, Elisabeth	2019	PLoS ONE	14	11	e0224916	10.1371/journal.pone.0224916	Konig 2019	Non-Gastro/Hepatology Focus
An artificial intelligent diagnostic system on mobile Android terminals for cholelithiasis by lightweight	Pang, Shanchen; Wang, Shuo; Rodriguez-Paton, Alfonso; Li, Pibao; Wang, Xun	2019	PLoS ONE	14	9	e0221720	10.1371/journal.pone.0221720	Pang 2019	Non-NLP Focus

convolutional neural network.									
Natural language processing of German clinical colorectal cancer notes for guideline-based treatment evaluation.	Becker, Matthias; Kasper, Stefan; Bockmann, Britta; Jockel, Karl-Heinz; Virchow, Isabel	2019	Int J Med Inf	127	ct4, 9711057	141-146	10.1016/j.ijmedinf.2019.04.022	Becker 2019	NLP used only as a study adjunct/enabler
3Dscript: animating 3D/4D microscopy data using a natural-language-based syntax.	Schmid, Benjamin; Tripal, Philipp; Fraas, Tina; Kersten, Christina; Ruder, Barbara; Gruneboom, Anika; Huisken, Jan; Palmisano, Ralf	2019	Nat Methods	16	4	278-280	10.1038/s41592-019-0359-1	Schmid 2019	Non-Gastro/Hepatology Focus
Needle in a Haystack: Natural Language Processing to Identify Serious Illness.	Udelsman, Brooks; Chien, Isabel; Ouchi, Kei; Brizzi, Kate; Tulsky, James A; Lindvall, Charlotta	2019	J Palliat Med	22	2	179-182	10.1089/jpm.2018.0294	Udelsman 2019	Non-Gastro/Hepatology Focus
NLPReViz: an interactive tool for natural language processing on clinical text.	Trivedi, Gaurav; Pham, Phuong; Chapman, Wendy W; Hwa, Rebecca; Wiebe, Janyce; Hochheiser, Harry	2018	J Am Med Inform Assoc	25	1	81-87	10.1093/jamia/ocx070	Trivedi 2018	Non-Gastro/Hepatology Focus
Social media for arthritis-related comparative effectiveness and	Curtis, Jeffrey R; Chen, Lang; Higginbotham, Phillip; Nowell,	2017	Arthritis Res Ther	19	1	48	10.1186/s13075-017-1251-y	Curtis 2017	Non-Gastro/Hepatology Focus

safety research and the impact of direct-to-consumer advertising.	W Benjamin; Gal-Levy, Ronit; Willig, James; Safford, Monika; Coe, Joseph; O'Hara, Kaitlin; Sa'adon, Roee								
RWD120 Understanding Treatment Patterns, Disruptions and Potential Limitations of Tyrosine Kinase Inhibitors in ROS1+ Non-Small Cell Lung Cancer Patients in a Retrospective Review of United States Medical Transcription Records	Shim A.; Singhania A.; Iwanyckyj D.; Otalora F.; Morrison B.; Wade S.	2022	Value in Health	25	12 Supplement	S472	https://dx.doi.org/10.1016/j.jval.2022.09.2345	Shim 2022	Non-Gastro/Hepatology Focus
RWD86 Healthcare Resource Utilization (HCRU) of United States ROS1+ Non-Small Cell Lung Cancer (NSCLC) Patients Treated With Tyrosine Kinase Inhibitors (TKIs): Analysis of Electronic Medical Transcription Records	Shim A.; Singhania A.; Iwanyckyj D.; Otalora F.; Morrison B.; Wade S.	2022	Value in Health	25	12 Supplement	S465	https://dx.doi.org/10.1016/j.jval.2022.09.2311	Shim 2022	Non-Gastro/Hepatology Focus

SURVEY ON VARIOUS PREDICATION MODELS FOR SIDE EFFECTS IN THE DRUG TO DRUG INTERACTIONS USING MACHINE LEARNING MODELS	Arunkumar M.; Baskaran T.S.	2022	NeuroQuantology	20	15	4668-4676	https://dx.doi.org/10.14704/NQ.2022.20.15.NQ88473	Arunkumar 2022	Non-Gastro/Hepatology Focus
OPTIMIZING HIGH-RISK HEPATITIS C VIRUS (HCV) IDENTIFICATION BY THE INCLUSION OF STRUCTURED, SEMI-STRUCTURED AND FREE-TEXT ELECTRONIC HEALTH RECORD DATA	Fong A.; Hughes J.; Gundapaneni S.; Hack B.; Barkhordar M.; Huang S.; Basch P.T.; Fernandez S.; Fishbein D.	2022	Hepatology	76	Supplement 1	S353-S354	https://dx.doi.org/10.1002/hep.32697	Fong 2022	Non-NLP Focus
CHARACTERIZING LIVER DISEASE (LD) PROGRESSION IN ALPHA-1 ANTITRYPSIN DEFICIENCY (AATD) WITH VERSUS (VS) WITHOUT LUNG DISEASE	Wu T.; Hagiwara M.; Gnass E.; Barman H.; Treem W.; Ren K.; Marins E.G.; Karki C.; Malhi H.	2022	Hepatology	76	Supplement 1	S137	https://dx.doi.org/10.1002/hep.32697	Wu 2022	NLP used only as a study adjunct/enabler
The value of real world evidence: The case of medical cannabis	Schlag A.K.; Zafar R.R.; Lynskey M.T.; Athanasiou-Fragkouli A.; Phillips L.D.; Nutt D.J.	2022	Frontiers in Psychiatry	13	(Schlag, Zafar, Lynskey, Athanasiou-Fragkouli, Phillips, Nutt) Drug Science, London,	1027159	https://dx.doi.org/10.3389/fpsyt.2022.1027159	Schlag 2022	Non-Gastro/Hepatology Focus

					United Kingdom(Schlag, Zafar, Nutt) Department of Brain Sciences, Faculty of Medicine, Imperial College London, London, United Kingdom(Phillips) Department of Management ,				
Leveraging Artificial Intelligence to Enhance Peer Review: Missed Liver Lesions on Computed Tomographic Pulmonary Angiography	Thomas S.P.; Fraum T.J.; Ngo L.; Harris R.; Balesh E.; Bashir M.R.; Wildman-Tobriner B.	2022	Journal of the American College of Radiology	19	11	1286-1294	https://dx.doi.org/10.1016/j.jacr.2022.07.013	Thomas 2022	Non-Gastro/Hepatology Focus
Clinically Significant Metachronous Colorectal Pathology Detected Among Young-Onset Colorectal Cancer Survivors: Implications for	Peacock O.; Vilar E.; Guraieb-Trueba M.; Thirumurthi S.; Chang G.J.; You Y.N.	2022	Gastroenterology	163	6	1682-1684.e2	https://dx.doi.org/10.1053/j.gastro.2022.08.030	Peacock 2022	Non-NLP Focus

Post-Resection Surveillance Guidelines									
Demographic Characteristics and Comorbidities of Patients with Narcolepsy: A Propensity Score Matched Cohort Study	Lipford M.C.; Ip W.; Awasthi S.; Moore J.L.; Tippmann-Peikert M.; Asfahan S.; Singh G.P.; Seiden D.; Gudeman J.	2022	Annals of Neurology	92	Supplement 29	S222	https://dx.doi.org/10.1002/ana.26484	Lipford 2022	Non-Gastro/Hepatology Focus
High Satisfaction with a Virtual Assistant for Plastic Surgery Frequently Asked Questions	Avila F.R.; Boczar D.; Spaulding A.C.; Quest D.J.; Samanta A.; Torres-Guzman R.A.; Maita K.C.; Garcia J.P.; Eldaly A.S.; Forte A.J.	2022	Aesthetic surgery journal		(Avila, Torres-Guzman, Maita, Garcia, Eldaly) postdoctoral research fellows(Boczar) PGY-1 surgery resident, Department of Surgery, University of Washington, Seattle, WA, United States(Spaulding) senior associate consultant, Robert D. and Patricia E. Kern		https://dx.doi.org/10.1093/asj/sjac290	Avila 2022	Non-Gastro/Hepatology Focus
Superinfection of Perirenal Hematoma: A Case Series and Systematic Review	Gaines J.M.; MacDonald E.J.; James J.; D'Souza N.; Kivitz S.; Lynch E.; Smith A.D.; Rai A.	2022	Journal of Endourology	36	Supplement 1	A214-A215	https://dx.doi.org/10.1089/end.2022.36001.abstracts	Gaines 2022	Non-Gastro/Hepatology Focus
Using Computer Vision and Natural Language Processing Techniques to Curate and Characterize Open Surgical Videos on YouTube	Zhang Y.; Goodman E.; Kennedy C.J.; Brat G.	2022	Journal of the American College of Surgeons	235	5 Supplement 1	S236-S237	https://dx.doi.org/10.1097/01.XCS.0000894860.70215.b1	Zhang 2022	Non-Gastro/Hepatology Focus

A real-time biosurveillance mechanism for early-stage disease detection from microblogs: a case study of interconnection between emotional and climatic factors related to migraine disease	Sarsam S.M.; Al-Samarraie H.; Ismail N.; Zaqout F.; Wright B.	2020	Network Modeling Analysis in Health Informatics and Bioinformatics	9	1	32	https://dx.doi.org/10.1007/s13721-020-00239-6	Sarsam 2020	Non-Gastro/Hepatology Focus
Introduction to Personalized Medicine in Pediatrics	Bupp C.P.; English B.K.; Rajasekaran S.; Prokop J.W.	2022	Pediatric Annals	51	10	e381-e386	https://dx.doi.org/10.3928/19382359-20220803-03	Bupp 2022	Review/Perspective Article Only
An augmented intelligence mobile phone chatbot for medication adherence and toxicity management among patients with gastrointestinal cancers on capecitabine	Lau-Min K.S.; Marini J.; Shah N.; Pucci D.; Blauch A.; Cambareri C.; Mooney B.; Johnston C.; Schumacher R.P.; White K.; Edward P.; Rosin G.R.; Jacobs L.A.; Shulman L.N.	2022	Journal of Clinical Oncology	40	28 Supplement	424	https://dx.doi.org/10.1200/JCO.2022.40.28_suppl.424	Lau-Min 2022	NLP used only as a study adjunct/enabler
How do others cope? Extracting coping strategies for adverse drug events from social media	Dirkson A.; Verberne S.; van Oortmerssen G.; Gelderblom H.; Kraaij W.	2022	Journal of biomedical informatics		(Dirkson, Verberne, Kraaij) Leiden Institute of Advanced Computer Science, Leiden University, Niels	104228	https://dx.doi.org/10.1016/j.jbi.2022.104228	Dirkson 2022	Non-Gastro/Hepatology Focus

					Bohrweg 1, CA Leiden 2333, Netherlands(van Oortmerssen) Leiden Institute of Advanced Computer Science, Leiden University, Niels Bohrweg 1, CA Leiden 2333,				
Representation of Racial Groups in Genomic Studies of Gastroenteropancreatic Neuroendocrine Neoplasms	Herring B.; Bonner A.; Guenter R.; Vickers S.; Chen H.; Yates C.; Rose J.B.	2022	HPB	24	Supplement 1	S325	https://dx.doi.org/10.1016/j.hpb.2022.05.687	Herring 2022	NLP used only as a study adjunct/enabler
ASSOCIATION OF SEPSIS SYMPTOMS AT ED PRESENTATION WITH RACE AND ETHNICITY	NEWBY R.; MISHRA P.; BARREDA F.; MYERS L.; KIPNIS P.; LIU V.	2022	Chest	162	4 Supplement	A1452	https://dx.doi.org/10.1016/j.chest.2022.08.1221	NEWBY 2022	Non-Gastro/Hepatology Focus
THE IMPACT OF CANDIDATE SELECTION ON EXTRACORPOREAL MEMBRANE OXYGENATION (ECMO) BRIDGE TO LUNG	WAHAB A.; C KENNEDY C.; Z SHAH S.; A SADDOUGHI S.; G PETERS S.; PENNINGTON K.	2022	Chest	162	4 Supplement	A2564	https://dx.doi.org/10.1016/j.chest.2022.08.2100	WAHAB 2022	Non-Gastro/Hepatology Focus

TRANSPLANT OUTCOMES									
Identifying Urinary Tract Infection-Related Information in Home Care Nursing Notes	Woo K.; Adams V.; Wilson P.; Fu L.-H.; Cato K.; Rossetti S.C.; McDonald M.; Shang J.; Topaz M.	2021	Journal of the American Medical Directors Association	22	5	1015-1021.e2	https://dx.doi.org/10.1016/j.jamda.2020.12.010	Woo 2021	Non-Gastro/Hepatology Focus
Management Outcomes in Patients with Suprachoroidal Hemorrhage after Anterior Segment Surgery	Fan J.; Hudson J.L.; Pakravan P.; Fan K.C.; Flynn H.W.	2022	Investigative Ophthalmology and Visual Science	63	7	3395-F0295		Fan 2022	Non-Gastro/Hepatology Focus
Endoscopic pars plana vitrectomy outcome after open globe injury with corneal opacity	Abouodah H.; Hamada K.; Pfannenstiel M.; Ajlan R.	2022	Investigative Ophthalmology and Visual Science	63	7	711-F0236		Abouodah 2022	Non-Gastro/Hepatology Focus
SWTRU: Star-shaped Window Transformer Reinforced U-Net for medical image segmentation	Zhang J.; Wu Q.; Wang Y.; Liu Y.; Xu X.; Song B.	2022	Computers in Biology and Medicine	150	(Zhang, Liu, Wu, Wang, Xu, Song) Qingdao University of Science and Technology, China(Liu) Dawning International Information Industry Co., Ltd, China(Liu) Sugon Nanjing Institute, Co., Ltd, China	105954	https://dx.doi.org/10.1016/j.compbiomed.2022.105954	Zhang 2022	Non-NLP Focus

Natural Language Processing of Symptoms Preceding Diagnosis and Palliative Radiotherapy for Bone Metastases	Chen J.J.; Friesner I.; Chang C.; Ni L.; Braunstein S.E.; Boreta L.; Hong J.C.	2022	International Journal of Radiation Oncology Biology Physics	114	3 Supplement	S18	https://dx.doi.org/10.1016/j.ijrobp.2022.07.364	Chen 2022	Non-Gastro/Hepatology Focus
1677P Risk factors predicting immune checkpoint inhibitors (ICIs) toxicity using machine learning computer algorithm	Holland R.; Miron K.; Goshen-Lago T.; Gordon N.; Zer A.; Ben-Aharon I.	2022	Annals of Oncology	33	Supplement 7	S1309	https://dx.doi.org/10.1016/j.annonc.2022.07.1756	Holland 2022	Non-NLP Focus
ARTIFICIAL INTELLIGENCE to CONNECT the USE of BIOLOGICS and SMALL MOLECULES in RHEUMATOID and PSORIATIC ARTHRITIS with A MULTIDISCIPLINARY EVALUATION: A REAL WORLD EVIDENCE APPROACH THROUGH NATURAL-LANGUAGE PROCESSING	Moranding P.; Maffia F.; Puggioni F.; Motta F.; Vecellio M.; Costanzo A.; Savevski V.; Selmi C.	2022	Annals of the Rheumatic Diseases	81	Supplement 1	392-393	https://dx.doi.org/10.1136/annrheumdis-2022-eular.3270	Moranding 2022	Non-Gastro/Hepatology Focus
#SurgOnc: Global discussions about surgical cancer care on Twitter during COVID-19	Gereta S.; Patel V.R.; Mackert M.; Haynes A.B.	2022	Journal of Clinical Oncology	40	16 Supplement 1		https://dx.doi.org/10.1200/JCO.2022.40.16_suppl.11045	Gereta 2022	Non-Gastro/Hepatology Focus

Predicting major bleeding events in anticoagulated cancer patients with venous thromboembolism using real-world data and machine learning	Martin A.J.M.; Palacios M.L.; Souto J.C.; Obispo B.; Aparicio J.; Garcia-Palomo A.; Sanchez A.; Aguayo C.; Abad D.G.; Vinuela-Beneitez M.C.; Benavent D.; Taberna M.; Arumi D.; Hernandez-Presa M.A.	2022	Journal of Clinical Oncology	40	16 Supplement 1		https://dx.doi.org/10.1200/JCO.2022.40.16_suppl.e18744	Martin 2022	Non-Gastro/Hepatology Focus
Evaluation of an automated artificial intelligence (AI)/ natural language processing (NLP) engine to match patients (pts) with advanced solid cancers to biomarker-driven early phase (EP) clinical trials	Morton C.; Ruta D.; Stavraka C.; Anam K.; Spicer J.F.; Regan A.; Sengul S.; Cernile G.; Borecki Y.; Schwering T.; Rai H.; Brierley P.; Duroe O.; Quarterman A.; Sarker D.	2022	Journal of Clinical Oncology	40	16 Supplement 1		https://dx.doi.org/10.1200/JCO.2022.40.16_suppl.e13513	Morton 2022	Non-Gastro/Hepatology Focus
Predicting early psychiatric readmission with natural language processing of narrative discharge summaries	Rumshisky A.; Ghassemi M.; Naumann T.; Szolovits P.; Castro V.M.; McCoy T.H.; Perlis R.H.	2016	Translational Psychiatry	6	10	e921	https://dx.doi.org/10.1038/TP.2015.182	Rumshisky 2016	Non-Gastro/Hepatology Focus
Progression of liver disease among patients with a new	Malhi H.; Wu T.; Hagiwara M.; Donadio G.; Gnass E.; Treem	2022	Journal of Hepatology	77	Supplement 1	S491-S492	https://dx.doi.org/10.1016/S0168-8278%2822%2901314-9	Malhi 2022	NLP used only as a study

diagnosis of protease inhibitor ZZ alpha-1 antitrypsin deficiency	W.; Ren K.; Marins E.G.; Karki C.								adjunct/enab ler
NAFL CIRRHOSIS IN PRIMARY CARE PATIENTS WITH DIABETES: HIGH PREVALENCE AND FREQUENCY OF SEVERE CLINICAL OUTCOMES	Chu J.N.; Fox R.K.; Goldman M.L.; Islam K.; Ross L.; Brandman D.	2022	Journal of General Internal Medicine	37	Supplement 2	S302	https://dx.doi.org/10.1007/s11606-022-07653-8	Chu 2022	Non-NLP Focus
IMPACT OF A DIAGNOSIS OF DIABETES ON THE FINANCIAL SUCCESS OF CROWDFUNDING CAMPAIGNS	Sloan C.; Doerstling S.; Ubel P.	2022	Journal of General Internal Medicine	37	Supplement 2	S285	https://dx.doi.org/10.1007/s11606-022-07653-8	Sloan 2022	Non-Gastro/Hepat ology Focus
Deep Learning for Cancer Symptoms Monitoring on the Basis of Electronic Health Record Unstructured Clinical Notes	Lindvall C.; Deng C.-Y.; Agaronnik N.D.; Kwok A.; Samineni S.; Umeton R.; MacKie-Jenkins W.; Kehl K.L.; Tulsky J.A.; Enzinger A.C.	2022	JCO Clinical Cancer Informatics	6	(Lindvall, Deng, Agaronnik, Kwok, Samineni, Umeton, MacKie-Jenkins, Kehl, Tulsky, Enzinger) Dana-Farber Cancer Institute, Boston, MA, United States(Lindval l, Agaronnik, Kehl, Tulsky, Enzinger) Harvard	e210013 6	https://dx.doi.org/10.1200/CCI.21.0013 6	Lindvall 2022	Non-Gastro/Hepat ology Focus

					Medical School, Boston, MA, United States(Lindvall, Ma				
Text classification of disease syndromes from emergency department free text notes	Tay S.Y.; Lim W.-Y.; Chen M.; Lee C.-H.; Janardanan K.	2022	Hong Kong Journal of Emergency Medicine	29	1 Supplement	13S	https://dx.doi.org/10.1177/1024907922109963	Tay 2022	Non-Gastro/Hepatology Focus
Identifying adverse drug reactions from free-text electronic hospital health record notes	Wasylewicz A.; van de Burgt B.; Weterings A.; Jessurun N.; Korsten E.; Egberts T.; Bouwman A.; Kerskes M.; Grouls R.; van der Linden C.	2022	British Journal of Clinical Pharmacology	88	3	1235-1245	https://dx.doi.org/10.1111/bcp.15068	Wasylewicz 2022	Non-Gastro/Hepatology Focus
Surgical instrument grounding for robot-assisted interventions	Gonzalez C.; Bravo-Sanchez L.; Arbelaez P.	2022	Computer Methods in Biomechanics and Biomedical Engineering: Imaging and Visualization	10	3	299-307	https://dx.doi.org/10.1080/21681163.2021.2002725	Gonzalez 2022	Non-Gastro/Hepatology Focus
RISK AND PREDICTORS OF COLORECTAL CANCER IN PATIENTS WITH ACUTE DIVERTICULITIS - A LARGE COHORT STUDY IN A DIVERSE U.S. POPULATION	Lin C.; Samant N.D.; Li D.; Baldawala T.B.	2022	Gastroenterology	162	7 Supplement	S-35	https://dx.doi.org/10.1016/S0016-5085%2822%2960088-X	Lin 2022	NLP used only as a study adjunct/enabler

COMPARATIVE EFFECTIVENESS OF VIRTUAL VS. IN-PERSON VISITS IN THE INITIAL EVALUATION OF ABDOMINAL PAIN PRIOR TO AND DURING THE COVID-19 PANDEMIC	Sasankan P.; Mcmichael J.; Lyu R.; Rouphael C.; Baggott B.; Garber A.	2022	Gastroenterology	162	7 Supplement	S-857	https://dx.doi.org/10.1016/S0016-5085%2822%2962029-8	Sasankan 2022	NLP used only as a study adjunct/enabler
Deep learning models for forecasting dengue fever based on climate data in Vietnam	Nguyen V.-H.; Tuyet-Hanh T.T.; Mulhall J.; Van Minh H.; Duong T.Q.; Van Chien N.; Nhung N.T.T.; Lan V.H.; Minh H.B.; Cuong D.; Bich N.N.; Quyen N.H.; Linh T.N.Q.; Tho N.T.; Nghia N.D.; Anh L.V.Q.; Phan D.T.M.; Hung N.Q.V.; Son M.T.	2022	PLoS Neglected Tropical Diseases	16	6	e0010509	https://dx.doi.org/10.1371/journal.pntd.0010509	Nguyen 2022	Non-Gastro/Hepatology Focus
IDENTIFICATION OF PANCREATIC DUCTAL ADENOCARCINOMA RISK FACTORS FROM ELECTRONIC HEALTH RECORDS USING NATURAL LANGUAGE PROCESSING	Sarwal D.; Wang L.; Gandhi S.; Sagheb E.; Janssens L.; Goncalves S.; Delgado A.; Doering K.; Hongfang L.; Majumder S.	2022	Gastroenterology	162	7 Supplement	S-243	https://dx.doi.org/10.1016/S0016-5085%2822%2960578-X	Sarwal 2022	Risk Factors For Disease Only

HIGHER PREVALENCE OF CARDIOVASCULAR DISEASE AMONG LEAN VERSUS NON-LEAN PATIENTS WITH NONALCOHOLIC FATTY LIVER DISEASE DESPITE LOWER PREVALENCE OF ATHEROGENIC RISK AND METABOLIC DISEASES	Wijarnpreecha K.; Li F.; Chen V.L.; Lok A.	2022	Gastroenterology	162	7 Supplement	S-1123	https://dx.doi.org/10.1016/S0016-5085%2822%2962697-0	Wijarnpreecha 2022	Non-NLP Focus
LONG TERM OUTCOMES OF INCIDENT ADVANCED NEOPLASIA 20 YEARS AFTER BASELINE SCREENING COLONOSCOPY IN CSP#380 PARTICIPANTS UNDERGOING FOLLOW-UP COLONOSCOPY	Sullivan B.; Redding T.S.; Qin X.; Thompson A.D.; Gupta S.; Bustamante R.; Earles A.; Weiss D.; Madison A.; Upchurch J.; Efird J.T.; Sims K.J.; Dominitz J.A.; Williams C.D.; Hauser E.R.; Lieberman D.A.	2022	Gastroenterology	162	7 Supplement	S-152	https://dx.doi.org/10.1016/S0016-5085%2822%2960367-6	Sullivan 2022	Non-NLP Focus
FISTULA HEALING IN PATIENTS WITH CROHN'S DISEASE ON USTEKINUMAB AND VEDOLIZUMAB	Newman K.L.; Johnson L.A.; Stidham R.; Higgins P.D.	2022	Gastroenterology	162	7 Supplement	S-822	https://dx.doi.org/10.1016/S0016-5085%2822%2961936-X	Newman 2022	NLP used only as a study adjunct/enabler
RACIAL AND ETHNIC DISPARITIES IN	Sanayei A.; Anand R.; Carli F.; Levi R.; Stein	2022	Gastroenterology	162	7 Supplement	S-694	https://dx.doi.org/10.1016/S0016-5085%2822%2961626-3	Sanayei 2022	NLP used only as a study

THE ACCURACY OF UPPER GI BLEEDING RISK PREDICTION MODELS	D.J.; Mironova M.; Celi L.A.								adjunct/enabler
Quality indicators in colonoscopy: the chasm between ideal and reality	Park S.B.; Cha J.M.	2022	Clinical Endoscopy	55	3	332-338	https://dx.doi.org/10.5946/ce.2022.037	Park 2022	Review/Perspective Article Only
A HEALTHCARE COMPANION USING MACHINE LEARNING LOGISTICS AND NATURAL LANGUAGE PROCESSING	Jeya Ramya V.; Srivel R.	2022	NeuroQuantology	20	5	4914-4925	https://dx.doi.org/10.14704/nq.2022.20.5.NQ22767	JeyaRamya 2022	Non-Gastro/Hepatology Focus
OPTIC NEURITIS CAUSED BY ACUTE LYMPHOCYTIC LEUKAEMIA	Jaya A.; Prihatningtias R.	2022	Neuro-Ophthalmology	46	Supplement 1	87-88	https://dx.doi.org/10.1080/01658107.2022.2064188	Jaya 2022	Non-Gastro/Hepatology Focus
Identification of Uncontrolled Symptoms in Cancer Patients Using Natural Language Processing	DiMartino L.; Miano T.; Wessell K.; Bohac B.; Hanson L.C.	2021	Journal of pain and symptom management		(DiMartino, Miano) RTI International, Research Triangle Park, United States(Wessell) Cecil G. Sheps Center for Health Services Research, University of North Carolina at Chapel Hill, Chapel Hill, United States(Bohac) North Carolina Translational and Clinic		https://dx.doi.org/10.1016/j.jpainsymman.2021.10.014	DiMartino 2021	Non-Gastro/Hepatology Focus
Predicting mortality in the surgical intensive care unit using artificial intelligence and	Parreco J.; Hidalgo A.; Kozol R.; Namias N.; Rattan R.	2018	American Surgeon	84	7	1190-1194	https://dx.doi.org/10.1177/000313481808400736	Parreco 2018	Non-Gastro/Hepatology Focus

natural language processing of physician documentation									
Rule and design of dual-function health food formula of Panacis Quinquefolii Radixbased on function orientation-algorithm integration	Song R.-L.; Ma J.-M.; Guo H.-F.; Yu S.; Yao J.-L.; Dong Y.; Zhong X.-J.; Shan D.-J.; Lyu F.; She G.-M.	2022	Chinese Traditional and Herbal Drugs	53	11	3415-3424	https://dx.doi.org/10.7501/j.issn.0253-2670.2022.11.018	Song 2022	Non-Gastro/Hepatology Focus
Natural Language Processing for Detection and Reporting of Findings Requiring Follow-Up in Ra	Domingo J.; Soni P.; Galal G.; Mukhin V.; Huang J.; Caron S.; Xinos S.; Slavicek R.; Creamer P.; Altman C.; Massa M.; Kadiyam K.; Ware B.; Suna M.; Gwardys H.; Bayer T.; Salamone P.; Gilstrap J.; Brooks T.; Byrd T.; Yuen L.; Etemadi M.	2022	Diagnosis	9	2	eA51-eA52	https://dx.doi.org/10.1515/dx-2022-0024	Domingo 2022	Non-Gastro/Hepatology Focus
SPACIOTMEPORAL MACHINE LEARNING ANALYSIS OF COMPLETE SMALL BOWEL ENDOSCOPY VIDEOS FOR	Kellerman R.; Bleiweiss A.; Samuel S.; Yehuda R.M.; Barzilay O.; Ben-Horin S.; Eliakim R.; Klang E.; Kopylov U.	2022	Gastrointestinal Endoscopy	95	6 Supplement	AB472-AB473	https://dx.doi.org/10.1016/j.gie.2022.04.1179	Kellerman 2022	Non-NLP Focus

PREDICTION OF OUTCOMES IN CROHN'S DISEASE									
LONG-TERM OUTCOMES OF PERIANAL FISTULAS IN PEDIATRIC CROHN'S DISEASE	Vu J.; McMichael J.; Kurowski J.A.; Hull T.L.; Lipman J.; Holubar S.; Steele S.R.; Lightner A.L.	2022	Diseases of the Colon and Rectum	65	5	43		Vu 2022	NLP used only as a study adjunct/enabler
ADENOMA DETECTION RATE (ADR) AND SERRATED DETECTION RATE (SDR) ARE CORRELATED IN ALL HOSPITAL SETTINGS BUT A HIGH ADR DOES NOT ENSURE ADEQUATE SDR	Sreedhar S.; D'Orazio B.; Yang A.; Halverson A.; Li T.; Keswani R.N.	2022	Gastrointestinal Endoscopy	95	6 Supplement	AB99	https://dx.doi.org/10.1016/j.gie.2022.04.264	Sreedhar 2022	Non-NLP Focus
DEVELOPMENT OF AN AUTOMATED REAL-TIME ERCP QUALITY REPORT CARD	Singh A.; Brenner T.A.; Bujnak B.; Patel L.; Mathews S.; Strauss A.T.; Khashab M.A.; Shin E.J.; Singh V.; Akshintala V.S.	2022	Gastrointestinal Endoscopy	95	6 Supplement	AB100-AB101	https://dx.doi.org/10.1016/j.gie.2022.04.266	Singh 2022	Non-NLP Focus
Clinical Trial Eligibility Criteria: A Structural Barrier to Diversity in Clinical Trial Enrollment	Snyder R.A.	2022	Journal of Clinical Oncology	173	(Snyder) Brody School of Medicine at East Carolina University, Greenville,	JCO.22.00537	https://dx.doi.org/10.1200/JCO.22.00537	Snyder 2022	Review/Perspective Article Only

					NC, United States				
AUTOMATED ELECTRONIC HEALTH RECORD ASCERTAINMENT OF TYPHLITIS: A CHILDREN'S ONCOLOGY GROUP REPORT	Miller T.P.; Li Y.; Masino A.; Vallee E.; Burrows E.; Castellino S.; Hawkins D.; Ramos M.; Lash T.; Aplenc R.; Grundmeier R.	2022	Pediatric Blood and Cancer	69	SUPPL 2	S62-S63	https://dx.doi.org/10.1002/pbc.29735	Miller 2022	Non-Gastro/Hepatology Focus
IS THERE AN ASSOCIATION BETWEEN ENDOMETRIAL THICKNESS AT TIME OF FROZEN EMBRYO TRANSFER AND THE INCIDENCE OF SUBCHORIONIC HEMATOMA OR VAGINAL BLEEDING?	Chang S.; Ottensoser L.; Wodoslawsky S.; Nazem T.G.; Gounko D.; Lee J.A.; Copperman A.B.; McAvey B.	2019	Fertility and Sterility	112	3 SUPPL	e165-e166		Chang 2019	Non-Gastro/Hepatology Focus
FIRST TRIMESTER VAGINAL BLEEDING DOES NOT PREDICT SMALL FOR GESTATIONAL AGE NEWBORNS FOLLOWING SINGLE EUPLOID FROZEN EMBRYO TRANSFER	Chang S.; Wodoslawsky S.; Ottensoser L.; Gharib Nazem T.; Gounko D.; Lee J.A.; Copperman A.B.	2019	Fertility and Sterility	112	3 SUPPL	e297		Chang 2019	Non-Gastro/Hepatology Focus
Association of Preinjury Medical Diagnoses with Pediatric Persistent	Yengo-Kahn A.M.; Hibshman N.; Bonfield C.M.; Torstenson E.S.;	2022	Journal of Head Trauma Rehabilitation	37	2	E80-E89	https://dx.doi.org/10.1097/HTR.0000000000000686	Yengo-Kahn 2022	Non-Gastro/Hepatology Focus

Postconcussion Symptoms in Electronic Health Records	Gifford K.A.; Belikau D.; Davis L.K.; Zuckerman S.L.; Dennis J.K.								
Gastric Intestinal Metaplasia is a Risk Factor for Progression to Gastric Dysplasia and Carcinoma in Patients Who Undergo Gastric Biopsy: A Population-Based Study	Shalaby A.; Kukowski M.; Ma C.C.; Davison J.	2022	Modern Pathology	35	SUPPL 2	517-519		Shalaby 2022	NLP used only as a study adjunct/enabler
SUPPORTING MODELLING IN TOXICOLOGY WITH NATURAL LANGUAGE PROCESSING	Teunis M.; Corradi M.	2022	Tissue Engineering - Part A	28	SUPPL 1	S634	https://dx.doi.org/10.1089/ten.tea.2022.29025.abstracts	Teunis 2022	Non-Gastro/Hepatology Focus
#ColonCancer: Social Media Discussions about Colorectal Cancer during the COVID-19 Pandemic	Patel V.R.; Gereta S.; Blanton C.J.; Chu A.L.; Reddy N.K.; MacKert M.; Nortje N.; Pignone M.P.	2022	JCO Clinical Cancer Informatics	6	(Patel, Gereta, Blanton, Chu, Reddy, Pignone) Dell Medical School, The University of Texas at Austin, Austin, TX, United States(MacKert) Center for Health Communication, Moody College of	e2100180	https://dx.doi.org/10.1200/CCI.21.00180	Patel 2022	Lack of Validation

					Communication, The University of Texas at Austin, Austin, TX, United				
Natural language processing and String Metric-assisted Assessment of Semantic Heterogeneity method for capturing and standardizing unstructured nursing activities in a hospital setting: a retrospective study	Vanalli M.; Cesare M.; Cocchieri A.; D'Agostino F.	2022	Annali di igiene : medicina preventiva e di comunita		(Vanalli, Cesare) Department of Biomedicine and Prevention, University of Rome Tor Vergata, Rome, Italy(Cocchieri) Fondazione Policlinico Universitario A. Gemelli IRCCS, University of Catholic Sacred Heart, Rome, Italy(D'Agostino) Saint Camillus Internati		https://dx.doi.org/10.7416/ai.2022.2517	Vanalli 2022	Non-Gastro/Hepatology Focus
Artificial intelligence-aided clinical annotation of a large multi-cancer genomic dataset	Kehl K.L.; Xu W.; Gusev A.; Bakouny Z.; Choueiri T.K.; Riaz I.B.; Elmarakeby H.; Van Allen E.M.; Schrag D.	2021	Nature Communications	12	1	7304	https://dx.doi.org/10.1038/s41467-021-27358-6	Kehl 2021	Non-Gastro/Hepatology Focus
Natural language processing for abstraction of cancer treatment toxicities: Accuracy versus human experts	Hong J.C.; Fairchild A.T.; Tanksley J.P.; Palta M.; Tenenbaum J.D.	2020	JAMIA Open	3	4	513-517	https://dx.doi.org/10.1093/jamiaopen/ooaa064	Hong 2020	Non-Gastro/Hepatology Focus

A novel methodology for building longitudinal, patient-centric real world datasets in hemophilia a	Skinner M.W.; Hanson G.; Xu T.; Ofori-Asenso R.; Ko R.H.; Cibelli E.; Nissen F.; Witkop M.; Sanabria F.; Shapiro A.D.	2021	Blood	138	SUPPL 1	594	https://dx.doi.org/10.1182/blood-2021-146160	Skinner 2021	Non-Gastro/Hepatology Focus
Derivation and validation of natural language processing algorithms to identify and classify venous thrombotic events from lower extremity duplex ultrasound reports	Abud A.; Houghton D.E.	2021	Blood	138	SUPPL 1	831	https://dx.doi.org/10.1182/blood-2021-144961	Abud 2021	Non-Gastro/Hepatology Focus
Neural networks for mining the associations between diseases and symptoms in clinical notes	Shah S.; Luo X.; Kanakasabai S.; Tuason R.; Klopper G.	2019	Health Information Science and Systems	7	1	1	https://dx.doi.org/10.1007/s13755-018-0062-0	Shah 2019	Non-Gastro/Hepatology Focus
Words prediction based on N-gram model for free-text entry in electronic health records	Yazdani A.; Safdari R.; Golkar A.; R. Niakan Kalhori S.	2019	Health Information Science and Systems	7	1	6	https://dx.doi.org/10.1007/s13755-019-0065-5	Yazdani 2019	Non-Gastro/Hepatology Focus
USING ANTICOAGULANT EXPOSURE TO MEASURE PRESCRIBING PATTERNS AND BLEEDING IN CANCER PATIENTS	Brundage J.; Barker S.; Herrick J.; Honholt T.; Bress A.P.; Lyons A.; Shah R.	2022	Journal of the American College of Cardiology	79	9 Supplement	1941	https://dx.doi.org/10.1016/S0735-1097%2822%2902932-1	Brundage 2022	Non-Gastro/Hepatology Focus

WITH ATRIAL FIBRILLATION									
Voice of Cancer Patients: Patient Experience Regarding Use of Tyrosine Kinase Inhibitors in Chronic Myeloid Leukemia	Aggarwal S.; Manish S.; Chaudhary G.; Aggarwal A.; Punetha H.; Jha J.; Gond R.K.	2019	Blood	134	Supplement 1	5843	https://dx.doi.org/10.1182/blood-2019-125195	Aggarwal 2019	Non-Gastro/Hepatology Focus
Using natural language processing to investigate diagnostic error in acute stroke	Mayampurath A.; Romo E.; Holl J.; Prabhakaran S.	2022	Stroke	53	SUPPL 1		https://dx.doi.org/10.1161/str.53.suppl_1.TMP38	Mayampurath 2022	Non-Gastro/Hepatology Focus
Intraocular silicone oil migration following a myocradial infarction	Gardner J.; Dihowm F.	2022	Journal of Investigative Medicine	70	2	681-682	https://dx.doi.org/10.1136/jim-2022-SRMC.472	Gardner 2022	Non-Gastro/Hepatology Focus
Predictors of treatment for inflammatory arthritis with immune modulating medications (IMM) in us veterans	Braaten T.; Pei S.; Rathod A.; Penmetsa G.; Douglas K.; Walker J.; Clewell J.; Walsh J.	2021	Arthritis and Rheumatology	73	SUPPL 9	1733-1735	https://dx.doi.org/10.1002/art.41966	Braaten 2021	Non-Gastro/Hepatology Focus
Application of Real-World Data to External Control Groups in Oncology Clinical Trial Drug Development	Yap T.A.; Jacobs I.; Baumfeld Andre E.; Lee L.J.; Beaupre D.; Azoulay L.	2021	Frontiers in Oncology	11	(Yap) Department of Investigational Cancer Therapeutics (Phase I Program), Division of	695936	https://dx.doi.org/10.3389/fonc.2021.695936	Yap 2021	Non-Gastro/Hepatology Focus

					Cancer Medicine, the University of Texas MD Anderson Cancer Center, Houston, TX, United States(Jacobs , Baumfeld Andre, Lee, Beaupre) Pfizer Inc, New York, NY, United St				
Machine Learning and Artificial Intelligence in Pharmaceutical Research and Development: a Review	Kolluri S.; Lin J.; Liu R.; Zhang Y.; Zhang W.	2022	AAPS Journal	24	1	19	https://dx.doi.org/10.1208/s12248-021-00644-3	Kolluri 2022	Non-Gastro/Hepatology Focus
P15-12 Improved QSAR model for cholestasis built with FDA drug label data	Girireddy M.; Saiakhov R.; Chakravarti S.	2021	Toxicology Letters	350	Supplement	S165	https://dx.doi.org/10.1016/S0378-4274%2821%2900631-7	Girireddy 2021	Non-Gastro/Hepatology Focus
A PREDICTION MODEL FOR METACHRONOUS ADVANCED NEOPLASIA AFTER COLORECTAL POLYPECTOMY UTILIZING POLYP, DEMOGRAPHIC, AND QUALITY FACTORS	Gupta S.; Bustamante R.; Earles A.; Demb J.; Yassin H.; Martinez M.E.; Saini S.D.; Fisher D.A.; Gawron A.J.; Kaltenbach T.R.; Patterson O.V.; DuVall S.L.; Messer K.; Liu L.	2021	Gastroenterology	160	6 Supplement	S-31	https://dx.doi.org/10.1016/S0016-5085%2821%2900820-9	Gupta 2021	Non-NLP Focus

NATURAL LANGUAGE PROCESSING IMPROVES THE IDENTIFICATION OF IPMN CYSTS AMONG PATIENTS WITH PANCREATIC CYST DIAGNOSTIC CODES	Khalaf N.; Abrams H.R.; Chiemeziem E.; Xu A.; Singh H.; Kanwal F.; El-Serag H.B.	2021	Gastroenterology	160	6 Supplement	S-479	https://dx.doi.org/10.1016/S0016-5085%2821%2901850-3	Khalaf 2021	Non-NLP Focus
Mild Adverse Events of Sputnik V Vaccine in Russia: Social Media Content Analysis of Telegram via Deep Learning	Jarynowski A.; Semenov A.; Kaminski M.; Belik V.	2021	Journal of Medical Internet Research	23	11	e30529	https://dx.doi.org/10.2196/30529	Jarynowski 2021	Non-Gastro/Hepatology Focus
The Clinician's Guide to the Machine Learning Galaxy	Shen L.; Kann B.H.; Taylor R.A.; Shung D.L.	2021	Frontiers in Physiology	12	(Shen) Department of Medicine, Brigham and Women's Hospital, Boston, MA, United States(Shen) Division of Gastroenterology, Hepatology and Endoscopy, Brigham and Women's Hospital, Boston, MA, United States(Kann) Department	658583	https://dx.doi.org/10.3389/fphys.2021.658583	Shen 2021	Non-Gastro/Hepatology Focus

					of Radiation Oncology, Dana-Farbe				
Engineering and clinical use of artificial intelligence (AI) with machine learning and data science advancements: radiology leading the way for future	Hameed B.M.Z.; Prerepa G.; Patil V.; Shekhar P.; Zahid Raza S.; Karimi H.; Paul R.; Naik N.; Modi S.; Vigneswaran G.; Prasad Rai B.; Chlosta P.; Somani B.K.	2021	Therapeutic Advances in Urology	13	(Hameed) Department of Urology, Father Muller Medical College, Mangalore, Karnataka, India(Prerepa) Department of Electronics and Communication, Manipal Institute of Technology, Manipal Academy of Higher Education, Manipal, Karnataka, India(Patil) Departm		https://dx.doi.org/10.1177/17562872211044880	Hameed 2021	Non-Gastro/Hepatology Focus
COVID-19 SignSym: A fast adaptation of a general clinical NLP tool to identify and normalize COVID-19 signs and symptoms to OMOP common data model	Wang J.; Abu-El-Rub N.; Gray J.; Pham H.A.; Zhou Y.; Manion F.J.; Liu M.; Song X.; Xu H.; Rouhizadeh M.; Zhang Y.	2021	Journal of the American Medical Informatics Association	28	6	1275-1283	https://dx.doi.org/10.1093/jamia/ocab015	Wang 2021	Non-Gastro/Hepatology Focus
Clinical inflection point detection on the basis of ehr data to identify clinical trial-ready patients with cancer	Kehl K.L.; Groha S.; Lepisto E.M.; Elmarakeby H.; Lindsay J.; Gusev A.; Van Allen E.M.; Hassett M.J.; Schrag D.	2021	JCO Clinical Cancer Informatics	5	(Kehl, Groha, Lepisto, Elmarakeby, Lindsay, Gusev, Van Allen, Hassett, Schrag) Division of Population Sciences, the Knowledge Systems	622-630	https://dx.doi.org/10.1200/CCI.20.00184	Kehl 2021	Non-Gastro/Hepatology Focus

					Group, Department of Medical Oncology, Dana-Farber Cancer Institute, Harvard Medical School, Boston, MA, United States				
Med-BERT: pretrained contextualized embeddings on large-scale structured electronic health records for disease prediction	Rasmy L.; Xiang Y.; Xie Z.; Tao C.; Zhi D.	2021	npj Digital Medicine	4	1	86	https://dx.doi.org/10.1038/s41746-021-00455-y	Rasmy 2021	Non-Gastro/Hepatology Focus
Evolution of Hematology Clinical Trial Adverse Event Reporting to Improve Care Delivery	Miller T.P.; Aplenc R.	2021	Current Hematologic Malignancy Reports	16	2	126-131	https://dx.doi.org/10.1007/s11899-021-00627-3	Miller 2021	Non-Gastro/Hepatology Focus
Current applications of artificial intelligence combined with urine detection in disease diagnosis and treatment	Tan J.; Qin F.; Yuan J.	2021	Translational Andrology and Urology	10	4		https://dx.doi.org/10.21037/tau-20-1405	Tan 2021	Non-Gastro/Hepatology Focus
Impact of Pharmacogenomic Information on Values of Care and	Zhu Y.; Lopes G.S.; Bielinski S.J.; Borah B.J.; Larson N.B.;	2021	Mayo Clinic Proceedings: Innovations,	5	1	35-45	https://dx.doi.org/10.1016/j.mayocpiqo.2020.08.009	Zhu 2021	Non-Gastro/Hepatology Focus

Quality of Life Associated with Codeine and Tramadol-Related Adverse Drug Events	Moyer A.M.; Olson J.E.; Wang L.; Weinshilboum R.; St. Sauver J.L.		Quality and Outcomes						
Natural language processing with machine learning to predict outcomes after ovarian cancer surgery	Barber E.L.; Garg R.; Persenaire C.; Simon M.	2021	Gynecologic Oncology	160	1	182-186	https://dx.doi.org/10.1016/j.ygyno.2020.10.004	Barber 2021	Non-Gastro/Hepatology Focus
Identifying Symptom Information in Clinical Notes Using Natural Language Processing	Koleck T.A.; Tatonetti N.P.; Bakken S.; Mitha S.; Henderson M.M.; George M.; Miaskowski C.; Smaldone A.; Topaz M.	2021	Nursing research	70	3	173-183	https://dx.doi.org/10.1097/NNR.0000000000000488	Koleck 2021	Non-Gastro/Hepatology Focus
Out-of-hours dedicated trans-nasal endoscopy service-a model for endoscopy recovery during COVID pandemic	Lim S.; Haboubi H.; Dawson P.; Machado A.; Mangsat E.; Santos S.; Anderson S.; Wong T.; Zeki S.; Dunn J.	2021	Gut	70	SUPPL 4	A188	https://dx.doi.org/10.1136/gutjnl-2021-BSG.350	Lim 2021	NLP used only as a study adjunct/enabler
Machine Learning-Based Prediction of Pancreatic Cancer in Patients with Pancreas Parenchymal or Ductal Abnormality on Cross-Sectional Imaging	Chen Q.; Chen W.; Zhou Y.; Lustigova E.; Wu B.U.	2021	Pancreas	50	7	1051	https://dx.doi.org/10.1097/MPA.0000000000001904	Chen 2021	NLP used only as a study adjunct/enabler

Risk of incident and fatal colorectal cancer after young onset adenoma diagnosis: A national cohort study	Casey Y.; Demb J.; Enwerem N.; Liu L.; Jackson C.; Bustamante R.; Earles A.; Shah S.; Gupta S.	2021	American Journal of Gastroenterology	116	SUPPL	S104-S105	https://dx.doi.org/10.14309/01.ajg.0000773412.18978.5f	Casey 2021	NLP used only as a study adjunct/enabler
Impact of baseline serrated polyp features on metachronous advanced serrated lesions	Rouphael C.; Bena J.; McMichael J.; Burke C.A.	2021	American Journal of Gastroenterology	116	SUPPL	S134-S135	https://dx.doi.org/10.14309/01.ajg.0000773704.07369.28	Rouphael 2021	NLP used only as a study adjunct/enabler
Are H. Pylori biopsies warranted in patients with prior negative results?	Loh J.; Rouphael C.; McMichael J.; Lyu R.; Click B.	2021	American Journal of Gastroenterology	116	SUPPL	S635	https://dx.doi.org/10.14309/01.ajg.0000779068.93813.78	Loh 2021	NLP used only as a study adjunct/enabler
Estimates of non-alcoholic fatty liver disease prevalence in a large, representative, northern california cohort using diagnosis codes, imaging and the dallas steatosis index	Saxena V.; Tucker L.-Y.S.; Seo S.; Mukhtar N.A.; Balasubramanian S.; MacDonald B.A.; Ready J.B.; Levin T.R.	2021	Hepatology	74	SUPPL 1	953A	https://dx.doi.org/10.1002/hep.32188	Saxena 2021	Non-NLP Focus
Prevalence and risk factors for ultrasoundpositive hepatic steatosis among primary care patients with diabetes	Fox R.K.; Chu J.; Sohn J.H.; Islam K.; Brandman D.	2021	Hepatology	74	SUPPL 1	979A	https://dx.doi.org/10.1002/hep.32188	Fox 2021	NLP used only as a study adjunct/enabler
The lmna r644c variant is associated with	Upadhyay K.; Du X.; Chen Y.; Stetson L.;	2021	Hepatology	74	SUPPL 1	1091A-1092A	https://dx.doi.org/10.1002/hep.32188	Upadhyay 2021	Risk Factors For Disease Only

hepatic steatosis in a large cohort and increases lipid accumulation in human hepatoma cells	Omary M.B.; Speliotes E.K.; Brady G.								
Natural language processing model to extract acute abnormalities from ct head reports	Torres-Lopez V.M.; Rovenolt G.; Garcia G.; Chacko S.; Olcese A.; Falcone G.; Payabvash S.; Sharma R.; Sansing L.; Sheth K.; Kim J.A.	2021	Annals of Neurology	90	SUPPL 27	S187	https://dx.doi.org/10.1002/ana.26180	Torres-Lopez 2021	Non-Gastro/Hepatology Focus
Brainclass: A classification model for summarizing brain injuries from ct reports after text extraction using brainner	Olcese A.J.; Lopez V.T.; Garcia G.E.; Chacko S.; Robinson A.; Gaiser E.; Falcone G.J.; Payabavash S.; Sharma R.; Sansing L.; Sheth K.N.; Kim J.A.	2021	Annals of Neurology	90	SUPPL 27	S183-S184	https://dx.doi.org/10.1002/ana.26180	Olcese 2021	Non-Gastro/Hepatology Focus
Development of a tool for detecting patient-level symptoms in relation with medications using disease blogs on the Internet	Matsuda S.; Ohtomo T.; Aoki K.; Okuyama M.; Miyake H.	2021	Pharmacoepidemiology and Drug Safety	30	SUPPL 1	194-195	https://dx.doi.org/10.1002/pds.5305	Matsuda 2021	Non-Gastro/Hepatology Focus
Identification of a diagnostically complex	Yu O.; Covey J.; Grafton J.; Cronkite D.;	2021	Pharmacoepidemiology and Drug Safety	30	SUPPL 1	240-241	https://dx.doi.org/10.1002/pds.5305	Yu 2021	Non-Gastro/Hepatology Focus

condition, polycystic ovarian syndrome, in a population-based cohort using electronic health record data	Kelley A.; Hansen K.; Hilpert J.; Schulze-Rath R.; Reed S.D.								
Incidence rates of select outcomes among patients with nonalcoholic steatohepatitis (NASH) and evidence of fibrosis and cirrhosis	Bertoia M.; Ness E.; Capozza T.; Titievsky L.; Seeger J.D.	2021	Pharmacoepidemiology and Drug Safety	30	SUPPL 1	31	https://dx.doi.org/10.1002/pds.5305	Bertoia 2021	NLP used only as a study adjunct/enabler
AI natural language processing in a digital integrative cancer care service to identify increased care requirements	Ballurkar K.; Chshipunova Y.; Yee J.; Lo J.; Tan Z.; Murali-Ganesh R.	2021	Journal of Medical Imaging and Radiation Oncology	65	SUPPL 1	204	https://dx.doi.org/10.1111/1754-9485.13302	Ballurkar 2021	Non-Gastro/Hepatology Focus
Live biotherapeutic probiotics human microbiomic: Machine learning insilco clinical trials. Novel practical approach to era of personalized probiotics medicine	Reyed R.M.	2021	Lifestyle Genomics	14	3	109	https://dx.doi.org/10.1159/000517609	Reyed 2021	Review/Perspective Article Only
Convolutional Neural Networks Naively Trained on Radiation	Waters M.R.; Kang K.H.; Brenneman R.J.; Caruthers D.;	2021	International Journal of Radiation	111	3 Supplement	S65-S66	https://dx.doi.org/10.1016/j.ijrobp.2021.07.164	Waters 2021	Non-Gastro/Hepatology Focus

Oncology-Specific DATA Outperforms Classical Natural Language Processing Approaches for Automated Identification of Common Toxicity Terms	Spraker M.B.; Abraham C.D.		Oncology Biology Physics						
Natural language processing model to extract acute abnormalities from CT head reports	Torres-Lopez V.; Rovenolt G.; Garcia G.; Chacko S.; Herman A.; Alexandria S.; Acosta J.; Payabvash S.; Falcone G.; Sharma R.; Sansing L.; Sheth K.; Kim J.	2021	Neurology	96	15 SUPPL 1			Torres-Lopez 2021	Non-Gastro/Hepatology Focus
Identifying patients at risk for fibrosis in a primary care NAFLD cohort	Schreiner A.; Livingston S.; Zhang J.; Mauldin P.; Moran W.P.	2021	Journal of General Internal Medicine	36	SUPPL 1	S149-S150	https://dx.doi.org/10.1007/s11606-021-06830-5	Schreiner 2021	NLP used only as a study adjunct/enabler
Computational extraction and analysis of de-identified medical records to characterize hyperammonemia in patients with fibrolamellar carcinoma (FLC)	Zack T.; Maisel S.; O'Neill A.F.; La Quaglia M.P.; Herman M.; Knox J.J.; Yaqubie A.; Venook A.P.; Mayer R.J.; Gordan J.D.; Abou-Alfa G.K.	2021	Journal of Clinical Oncology	39	15 SUPPL		https://dx.doi.org/10.1200/JCO.2021.39.15-suppl.e16169	Zack 2021	Non-Gastro/Hepatology Focus

Using open-source natural language processing to classify traumatic cranial hemorrhages	Lopez A.; Crawford M.; Tran D.K.; Chen J.	2021	Journal of Neurotrauma	38	14	A83	https://dx.doi.org/10.1089/neu.2021.29111.abstracts	Lopez 2021	Non-Gastro/Hepatology Focus
Crowdsourcing post-marketing safety surveillance for migraine preventives: Self-reported adverse events associated with calcitonin gene-related peptide (CGRP) therapeutics on a social media forum	Zhang P.; Kamitaki B.; Do T.	2021	Headache	61	SUPPL 1	92-93	https://dx.doi.org/10.1111/head.14130	Zhang 2021	Non-Gastro/Hepatology Focus
Identification of patients with migraine, migraine-related symptoms and migraine medication use within electronic medical records using artificial intelligence	Riskin D.; Cady R.; Shroff A.; Hindiyeh N.A.; Smith T.R.; Kymes S.	2021	Headache	61	SUPPL 1	25-26	https://dx.doi.org/10.1111/head.14130	Riskin 2021	Non-Gastro/Hepatology Focus
Scintigraphic assessment of proximal and distal phasicantral contraction synchronicity for evaluation of patients with suspected gastroparesis	Maurer A.; Cole N.; Dadparvar S.; Powell D.; Mathai M.; Shahsavari D.; Parkman H.	2021	Journal of Nuclear Medicine	62	SUPPL 1			Maurer 2021	Non-NLP Focus

ID: 3523799 REFERRAL PATTERNS, POLYP FEATURES, AND CLINICAL OUTCOMES FOR COLORECTAL POLYPS >= 2 CM IN A LARGE TERTIARY CARE HEALTH SYSTEM	Nguyen B.S.; Soroudi C.; Yu A.R.; Smith B.; Treasure M.; Kahlon S.; Kim S.; Thaker A.M.; Yang L.; May F.F.P.	2021	Gastrointestinal Endoscopy	93	6 Supplement	AB75-AB76	https://dx.doi.org/10.1016/j.gie.2021.03.202	Nguyen 2021	NLP used only as a study adjunct/enabler
Tip of the j-pouch leaks: Diagnosis, management and longterm pouch survival	Rajamanickam R.; Gorgun E.; Valente M.A.; Lightner A.; Hull T.; Church J.; Steele S.; Holubar S.D.	2021	Diseases of the Colon and Rectum	64	5	37	https://dx.doi.org/10.1097/DCR.0000000000002029	Rajamanickam 2021	NLP used only as a study adjunct/enabler
New machine learning approaches to estimate the functional consequence of mutations in diverse human populations	Itan Y.; Wu Y.; Bayrak C.S.	2021	Journal of Clinical Immunology	41	SUPPL 1	S135	https://dx.doi.org/10.1007/s10875-021-01001-x	Itan 2021	Non-Gastro/Hepatology Focus
Abstract No. 112 Deep learning detection of bleeds on angiography	Xi I.; Nadolski G.; Gade T.; Hunt S.	2021	Journal of Vascular and Interventional Radiology	32	5 Supplement	S50	https://dx.doi.org/10.1016/j.jvir.2021.03.118	Xi 2021	Non-NLP Focus
Patient factors and clinical outcomes associated with an atypical presentation of older adults hospitalized with COVID-19	Chauhan L.; Marziliano A.; Sinvani L.; Burns E.; Zhang M.; Makhnevich A.; Carney M.; Diefenbach M.	2021	Journal of the American Geriatrics Society	69	SUPPL 1	S104	https://dx.doi.org/10.1111/jgs.17115	Chauhan 2021	Non-Gastro/Hepatology Focus

Regular individual feedback on key performance indicators improves polyp and adenoma detection in colonoscopy	Lim S.; Tritto G.; Zeki S.; De Martino S.	2021	Gut	70	SUPPL 1	A63-A64	https://dx.doi.org/10.1136/gutjnl-2020-bsgcampus.119	Lim 2021	NLP used only as a study adjunct/enabler
Discrepant fib-4 and nfs scores in a primary care nafld cohort	Schreiner A.; Livingston S.; Marsden J.; Zhang J.; Gebregziabher M.; Durkalski-Mauldin V.; Koch D.; Mauldin P.; Rockey D.; Moran W.	2021	Journal of Investigative Medicine	69	2	446-447	https://dx.doi.org/10.1136/jim-2021-SRMC.74	Schreiner 2021	NLP used only as a study adjunct/enabler
A framework for applying natural language processing in digital health interventions	Funk B.; Sadeh-Sharvit S.; Fitzsimmons-Craft E.E.; Trockel M.T.; Monterubio G.E.; Goel N.J.; Balantekin K.N.; Eichen D.M.; Flatt R.E.; Firebaugh M.-L.; Jacobi C.; Graham A.K.; Hoogendoorn M.; Wilfley D.E.; Barr Taylor C.	2020	Journal of Medical Internet Research	22	2	e13855	https://dx.doi.org/10.2196/13855	Funk 2020	Non-Gastro/Hepatology Focus
Enhancing Prediction of Drug Indication and Side Effects through Named	Mohanapriya D.; Beena R.	2020	European Journal of Molecular and Clinical Medicine	7	6	170-176		Mohanapriya 2020	Non-Gastro/Hepatology Focus

Entity Recognition and Jointly Learning of Syntactic Structures of Sentences									
Ontological organization and bioinformatic analysis of adverse drug reactions from package inserts: Development and usability study	Li X.; Lin X.; Ren H.; Guo J.	2020	Journal of Medical Internet Research	22	7	e20443	https://dx.doi.org/10.2196/20443	Li 2020	Non-Gastro/Hepatology Focus
An Electronic Health Record Text Mining Tool to Collect Real-World Drug Treatment Outcomes: A Validation Study in Patients With Metastatic Renal Cell Carcinoma	van Laar S.A.; Gombert-Handoko K.B.; Guchelaar H.-J.; Zwaveling J.	2020	Clinical Pharmacology and Therapeutics	108	3	644-652	https://dx.doi.org/10.1002/cpt.1966	vanLaar 2020	Non-Gastro/Hepatology Focus
Impact of viral symptoms on the performance of the modified centor score to predict pediatric group A streptococcal pharyngitis	Nadeau N.; Kimia A.; Fine A.M.	2020	American Journal of Emergency Medicine	38	7	1322-1326	https://dx.doi.org/10.1016/j.ajem.2019.10.026	Nadeau 2020	Non-Gastro/Hepatology Focus
Comparing Breast and Abdominal Subspecialists' Follow-Up Recommendations	DiPiro P.J.; Alper D.P.; Giess C.S.; Glazer D.I.; Lee L.K.; Lacson R.; Khorasani R.	2020	Journal of the American College of Radiology	17	6	773-778	https://dx.doi.org/10.1016/j.jacr.2019.12.024	DiPiro 2020	Non-Gastro/Hepatology Focus

for Incidental Liver Lesions on Breast MRI									
eHealth-mind the gap	Hollander-Mieritz C.; Johansen C.; Pappot H.	2020	Acta Oncologica		(Hollander-Mieritz, Johansen, Pappot) Department of Oncology, Rigshospitalet, University of Copenhagen, Copenhagen, Denmark(Johansen, Pappot) Danish Cancer Society, Copenhagen, Denmark	01-Feb	https://dx.doi.org/10.1080/0284186X.2020.1794037	Hollander-Mieritz 2020	Non-Gastro/Hepatology Focus
Leveraging digital data to inform and improve quality cancer care	Hernandez-Boussard T.; Blayney D.W.; Brooks J.D.	2020	Cancer Epidemiology Biomarkers and Prevention	29	4	816-822	https://dx.doi.org/10.1158/1055-9965.EPI-19-0873	Hernandez-Boussard 2020	Non-Gastro/Hepatology Focus
Artificial Intelligence in Cancer Staging: Limitless Potential or Passing Fad?	Kunstman J.W.	2020	Annals of Surgical Oncology	27	4	978-979	https://dx.doi.org/10.1245/s10434-019-08182-1	Kunstman 2020	Non-NLP Focus
Documentation of Palliative and End-of-Life Care Process Measures among Young Adults Who Died of Cancer: A Natural Language	Poort H.; Zupanc S.N.; Leiter R.E.; Wright A.A.; Lindvall C.	2020	Journal of Adolescent and Young Adult Oncology	9	1	100-104	https://dx.doi.org/10.1089/jayao.2019.0040	Poort 2020	Non-Gastro/Hepatology Focus

Processing Approach									
Natural language processing of electronic health records is superior to billing codes to identify symptom burden in hemodialysis patients	Chan L.; Beers K.; Yau A.A.; Chauhan K.; Duffy A.; Chaudhary K.; Debnath N.; Saha A.; Pattharanitima P.; Cho J.; Kotanko P.; Federman A.; Coca S.G.; Van Vleck T.; Nadkarni G.N.	2020	Kidney International	97	2	383-392	https://dx.doi.org/10.1016/j.kint.2019.10.023	Chan 2020	Non-Gastro/Hepatology Focus
Influenza surveillance using family-reported outcomes at emergency triage in southern Taiwan	Weng T.-C.; Ho T.-S.	2020	International Journal of Infectious Diseases	101	Supplement 1	206	https://dx.doi.org/10.1016/j.ijid.2020.09.550	Weng 2020	Non-Gastro/Hepatology Focus
Natural language processing: An automated alternative to determining inappropriate group a streptococcal testing	Lee B.R.; Linafelter A.; Burns A.; Burris A.; Jones H.; Dusin J.; El Feghaly R.E.	2020	Open Forum Infectious Diseases	7	SUPPL 1	S72	https://dx.doi.org/10.1093/ofid/ofaa417.162	Lee 2020	Non-Gastro/Hepatology Focus
Outcomes of patients presenting with headaches hospitalized with COVID-19	Jaladanki S.K.; Kumar A.; Van Vleck T.; Somani S.; Zhao S.; Nadkarni G.N.; Charney A.	2020	Annals of Neurology	88	SUPPL 25	S150	https://dx.doi.org/10.1002/ana.25865	Jaladanki 2020	Non-Gastro/Hepatology Focus

Understanding the interaction between Gut microbiome and the brain through machine learning based modeling	Tirat-Gefen Y.G.	2020	Annals of Neurology	88	SUPPL 25	S194-S195	https://dx.doi.org/10.1002/ana.25865	Tirat-Gefen 2020	Review/Perspective Article Only
Contextualizing background safety rates using comparable historical clinical trials	Li H.; Fantana A.; Connelly M.; Hilzinger T.S.; Singh M.	2020	Pharmacoepidemiology and Drug Safety	29	SUPPL 3	374-375	https://dx.doi.org/10.1002/pds.5114	Li 2020	Non-Gastro/Hepatology Focus
Identifying adenomyosis and undiagnosed adenomyosis within natural language processed clinical notes of an electronic health record database	Loughlin A.M.; Reznor G.; Doherty M.; Missmer S.A.; Chiuve S.E.; Enger C.	2020	Pharmacoepidemiology and Drug Safety	29	SUPPL 3	85	https://dx.doi.org/10.1002/pds.5114	Loughlin 2020	Non-Gastro/Hepatology Focus
Characterization of women with adenomyosis identified in a large U.S electronic health record database with and without hysterectomy	Chiuve S.E.; Loughlin A.M.; Missmer S.A.; Snabes M.C.; Gordon K.; Reznor G.; Doherty M.C.; Enger C.	2020	Pharmacoepidemiology and Drug Safety	29	SUPPL 3	83-84	https://dx.doi.org/10.1002/pds.5114	Chiuve 2020	Non-Gastro/Hepatology Focus
Hepatitis C virus screening of people with severe mental illness: a cost-effectiveness analysis	Girardin F.; Painter C.; Hearmon N.; Eddowes L.; Kaiser S.; Negro F.; Vernaz N.	2020	Journal of Hepatology	73	Supplement 1	S818-S819	https://dx.doi.org/10.1016/S0168-8278%2820%2932079-1	Girardin 2020	NLP used only as a study adjunct/enabler

Natural language processing for the extraction of patient symptoms during cancer radiotherapy	Hong J.	2020	Health Services Research	55	SUPPL 1	44	https://dx.doi.org/10.1111/1475-6773.13387	Hong 2020	Non-Gastro/Hepatology Focus
Adherence to established process measures in the evaluation of hepatic steatosis on imaging in a large tertiary care network	Shroff H.; VanWagner L.; Gregory D.; Damman S.; Keswani R.; Rinella M.	2020	Journal of Hepatology	73	Supplement 1	S414	https://dx.doi.org/10.1016/S0168-8278%2820%2931313-1	Shroff 2020	Non-NLP Focus
Endoscopic outcomes of duodenal carcinoid	Kesar V.; Mir A.S.; Grider D.J.; Yeaton P.	2020	American Journal of Gastroenterology	115	SUPPL	S647		Kesar 2020	NLP used only as a study adjunct/enabler
Treatment and Outcomes of Small Intestine Variceal Bleeding in Cirrhosis	Jansson-Knodell C.; Calderon G.; Ghabril M.	2020	American Journal of Gastroenterology	115	SUPPL	S503	https://dx.doi.org/10.14309/01.ajg.0000705996.53151.00	Jansson-Knodell 2020	Non-NLP Focus
Clinical and Endoscopic Predictors of Metachronous Advanced Serrated Lesions	Rouphael C.; Bena J.; McMichael J.; Burke C.A.	2020	American Journal of Gastroenterology	115	SUPPL	S153-S154	https://dx.doi.org/10.14309/01.ajg.0000703308.19498.3c	Rouphael 2020	Non-NLP Focus
Clinical and tumor characteristics of nonalcoholic fatty liver disease associated hepatocellular carcinoma in patients with and without cirrhosis	Benhammou J.N.; Aby E.S.; Lin J.; Markovic D.; Tong M.J.	2020	Hepatology	72	1 SUPPL	631A-632A	https://dx.doi.org/10.1002/hep.31579	Benhammou 2020	NLP used only as a study adjunct/enabler

Characteristics of malignancy in lesbian,gay, bisexual, and transgender/trans sexual (LGBT)population	Al-Shbool G.; Farid S.; Nassar A.; Kim C.	2020	Cancer Epidemiology Biomarkers and Prevention	29	6 SUPPL 2		https://dx.doi.org/10.1158/1538-7755.DISP19-C021	Al-Shbool 2020	Non-Gastro/Hepatology Focus
Calling attention to hepatic steatosis in a ct scan impression is associated with increased recognition of NAFLD in primary care	Hernandez L.; Evans G.; Busebee B.; White M.; Fondahn E.; Crippin J.S.; McHenry S.	2020	Hepatology	72	1 SUPPL	959A	https://dx.doi.org/10.1002/hep.31579	Hernandez 2020	NLP used only as a study adjunct/enabler
Hepatic encephalopathy is associated with a distinct speech pattern	Bloom P.P.; Arvind A.; Daidone M.; Robin J.; Xu M.; Gupta A.S.; Chung R.T.	2020	Hepatology	72	1 SUPPL	1070A-1071A	https://dx.doi.org/10.1002/hep.31579	Bloom 2020	NLP used only as a study adjunct/enabler
PCN267 Further Development of Artificial Intelligence Supporting Systematic Literature Review for Conducting Cost-Effectiveness Analysis	Sakata Y.; Inoue K.; Nagasawa T.; Ooishi M.; Azuma M.; Kitabayashi H.; Kusaba S.; Tanaka R.; Nawata S.; Takizaki K.; Sudo M.; Okamoto R.; Abe R.; Nakatsui M.; Okuno Y.	2020	Value in Health	23	Supplement 2	S470	https://dx.doi.org/10.1016/j.jval.2020.08.404	Sakata 2020	Non-Gastro/Hepatology Focus
Two diseases in a single lymph node: nodular lymphocyte predominant	Erkek E.T.; Yazici S.; Mazmanoglu S.; Barisik N.O.	2020	Hematology, Transfusion and Cell Therapy	42	Supplement 1	49-50	https://dx.doi.org/10.1016/j.htct.2020.09.088	Erkek 2020	Non-Gastro/Hepatology Focus

hodgkin lymphoma and kaposi's sarcoma									
DERIVATION AND VALIDATION OF THE HA-VTE AND HA-MB INTERMOUNTAIN RISK SCORES FROM UBIQUITOUS CLINICAL BIOMARKERS TO PREDICT 90-DAY HOSPITAL-ASSOCIATED VENOUS THROMBOEMBOLISM AND MAJOR BLEEDING AMONG MEDICAL PATIENTS	Woller S.; Stevens S.; Snow G.; Lloyd J.; Bledsoe J.; Fazili M.; Horne B.	2020	Chest	158	4 Supplement	A2452-A2453	https://dx.doi.org/10.1016/j.chest.2020.09.034	Woller 2020	Non-Gastro/Hepatology Focus
Reviewing electronic health records with the use of natural language processing to determine the prognostic impact of AF and anticoagulation therapy in patients undergoing PCI	Dujardin K.; Anne W.; Pollet P.; Acou W.J.; Galvao P.	2020	Europace	22	SUPPL 1	i398		Dujardin 2020	Non-Gastro/Hepatology Focus
Clinically Significant Toxicities Attributable to Pencil Beam Scanning Proton	Lucas J.T.; Faught A.M.; Tinkle C.L.; Rose M.L.; Hubler A.; Kaste S.; Hsu C.Y.; Harreld J.;	2020	International Journal of Radiation Oncology Biology Physics	108	3 Supplement	S125-S126	https://dx.doi.org/10.1016/j.ijrobp.2020.07.848	Lucas 2020	Non-Gastro/Hepatology Focus

Therapy in Pediatric Patients Enrolled oi a Single Institution Phase IV Clinical Trial: Early Results from SJPROTON1	Sabin N.D.; Acharya S.; Hua C.H.; Armstromg G.T.; Krasin M.; Merchant T.E.								
Comparisons of Treatment Outcomes and Patterns of Lymph Node Involvement in T4 Prostate Cancer Patients	Kim M.; Abu-Gheida I.; Bathala T.; Maldonado J.A.; Khan M.; Anscher M.S.; Frank S.J.; Choi S.; Nguyen Q.N.; Hoffman K.E.; McGuire S.E.; Kuban D.A.; Aparicio A.; Chapin B.; Tang C.	2020	International Journal of Radiation Oncology Biology Physics	108	3 Supplement	e912	https://dx.doi.org/10.1016/j.ijrobp.2020.07.542	Kim 2020	Non-Gastro/Hepatology Focus
OnCare Connect: A Text-Based Platform to Allow for Earlier Interventions to Decrease Toxicity Progression in Oncology Patients Receiving Chemoradiation	Grewal A.; Ngan A.; Laventure B.; Southwick L.; Klinger E.; Merchant R.	2020	International Journal of Radiation Oncology Biology Physics	108	2 Supplement	E66	https://dx.doi.org/10.1016/j.ijrobp.2020.02.631	Grewal 2020	Non-Gastro/Hepatology Focus
Machine learning for prospective identification of immunotherapy related adverse events (irAEs)	Margalski D.; Lycan T.; Rajendran S.; Topaloglu U.	2020	Journal of Clinical Oncology	38	15		https://dx.doi.org/10.1200/JCO.2020.38.15_suppl.e14064	Margalski 2020	Non-Gastro/Hepatology Focus
BERT-based few-shot learning for automatic	Kuwabara R.; Han C.; Murao K.; Satoh S.	2020	International Journal of Computer Assisted	15	1 Supplement	S148-S149	https://dx.doi.org/10.1007/s11548-020-02171-6	Kuwabara 2020	Non-Gastro/Hepatology Focus

anomaly classification from Japanese multi-institutional CT scan reports			Radiology and Surgery						
Mining and analysis of opioid content in longitudinal data posted in a social media forum	Sarker A.; Deroos F.; Gonzalez-Hernandez G.; O'Connor K.; Perrone J.	2020	Clinical Toxicology	58	6	571	https://dx.doi.org/10.1080/15563650.2020.1741981	Sarker 2020	Non-Gastro/Hepatology Focus
23. Natural language processing for automated identification of intraoperative vascular injury in anterior lumbar spine surgery	Karhade A.V.; Bongers M.; Groot O.; Fogel H.A.; Hershman S.H.; Tobert D.G.; Srivastava S.; Bono C.M.; Kang J.D.; Harris M.; Schwab J.H.	2020	Spine Journal	20	9 Supplement	S11-S12	https://dx.doi.org/10.1016/j.spinee.2020.05.126	Karhade 2020	Non-Gastro/Hepatology Focus
The clinical outcome of endoscopic trans-ethmosphenoid optic canal decompression for indirect traumatic optic neuropathy	Ma H.; Gao Y.; Li J.; Shi J.; Lu R.	2020	Investigative Ophthalmology and Visual Science	61	7			Ma 2020	Non-Gastro/Hepatology Focus
The pace, presentation, and profile of community-acquired sepsis and infection	Liu V.; Bhimarao M.; Greene J.D.; Barreda F.; Manickam R.; Escobar G.	2020	American Journal of Respiratory and Critical Care Medicine	201	1			Liu 2020	Non-Gastro/Hepatology Focus
Identification and burden of chronic cough in adults in a large managed care organization	Zeiger R.S.; Schatz M.; Xie F.; Hong B.D.; Weaver J.P.; Schelfhout J.; Chen W.	2020	American Journal of Respiratory and Critical Care Medicine	201	1			Zeiger 2020	Non-Gastro/Hepatology Focus

1131 COLORECTAL CANCER MORTALITY IN VETERANS WITH LOW-RISK ADENOMAS (LRA) AND THE EFFECT OF SUBSEQUENT COLONOSCOPY	Imperiale T.F.; Myers L.; Imler T.D.; Sherer E.A.; Kahi C.J.; Ransohoff D.F.	2020	Gastroenterology	158	6 Supplement 1	S-221	https://dx.doi.org/10.1016/S0016-5085%2820%2931243-9	Imperiale 2020	Non-NLP Focus
SMALL INTESTINE VARICES AT A HIGH-VOLUME LIVER TRANSPLANT CENTER: A CASE SERIES	Jansson-Knodell C.; Calderon G.; Weber R.; Ghabril M.S.	2020	Gastroenterology	158	6 Supplement 1	S-615	https://dx.doi.org/10.1016/S0016-5085%2820%2932235-6	Jansson-Knodell 2020	NLP used only as a study adjunct/enabler
YIELD OF AND RISK FACTORS FOR ADVANCED NEOPLASIA AND LONG-TERM OUTCOMES IN VETERANS WITH 3 OR MORE NON-ADVANCED ADENOMAS ON INDEX COLONOSCOPY	Ha J.; Walker M.J.; Myers L.; Ballard C.J.; Imperiale T.F.	2020	Gastroenterology	158	6 Supplement 1	S-645	https://dx.doi.org/10.1016/S0016-5085%2820%2932306-4	Ha 2020	NLP used only as a study adjunct/enabler
CHARACTERISTICS AND OUTCOMES AMONG INDIVDIUALS WITH TRADITIONAL SERRATED ADENOMA	Godil S.; Demb J.; Bustamante R.; Earles A.; Liu L.; Gupta S.	2020	Gastroenterology	158	6 Supplement 1	S-648	https://dx.doi.org/10.1016/S0016-5085%2820%2932312-X	Godil 2020	NLP used only as a study adjunct/enabler
Risk of metachronous pathology among survivors of	Peacock O.; Yang Y.; Thirumurthi S.; Nguyen S.; Lum	2020	Annals of Surgical Oncology	27	Supplement 1	S17	https://dx.doi.org/10.1245/s10434-020-08278-z	Peacock 2020	NLP used only as a study

sporadic young-onset colorectal cancer: Implications for post-resection colonoscopic surveillance	P.; Rodriguez-Bigas M.; Bednarski B.; Messick C.; Skibber J.; Chang G.; Vilar Sanchez E.; You Y.N.								adjunct/enabler
Metachronous colorectal pathology among survivors of young-onset colorectal cancer: Implications for postresection colonoscopic surveillance	Peacock O.; Yang Y.; Thirumurthi S.; Nguyen S.T.N.; Lum P.; Rodriguez-Bigas M.A.; Bednarski B.K.; Messick C.; Skibber J.M.; Chang G.J.; Sanchez E.V.; You Y.N.	2020	Journal of Clinical Oncology	38	4 Supplement		https://dx.doi.org/10.1200/JCO.2020.38.4_suppl.64	Peacock 2020	NLP used only as a study adjunct/enabler
Association of Asthma Prognosis with Risk of Asthma-Associated Infectious and Inflammatory Multimorbidities (AIMs) in Children with Asthma	Kwon J.; Ryu E.; Wi C.-I.; Seol H.; King K.; Yoon J.; Park M.; Sohn S.; Liu H.; Juhn Y.	2020	Journal of Allergy and Clinical Immunology	145	2 Supplement	AB110	https://dx.doi.org/10.1016/j.jaci.2019.12.547	Kwon 2020	Non-Gastro/Hepatology Focus
SAFETY OF FLEXIBLE SIGMOIDOSCOPY IN PREGNANT PATIENTS WITH INFLAMMATORY BOWEL DISEASE - A 10 YEAR STUDY	Ko M.S.; Rudrapatna V.; Avila P.; Mahadevan U.	2019	Gastroenterology	156	6 Supplement 1	S-19	https://dx.doi.org/10.1016/S0016-5085%2819%2936818-0	Ko 2019	NLP used only as a study adjunct/enabler
CANCER TRACKING SYSTEM	Zhang Y.; Mehta R.; Taddei T.H.	2019	Gastroenterology	156	6 S1	S-1192	https://dx.doi.org/10.1016/S0016-5085%2819%2939956-1	Zhang 2019	NLP used only as a

IMPROVES TIMELINESS OF LIVER CANCER CARE AT A VETERANS HOSPITAL									study adjunct/enabler
THE CLINICAL SPECTRUM OF GROOVE PANCREATITIS IS MUCH WIDER THAN IT IS BELIEVED	Ooka K.; Singh H.; Warndorf M.; Althouse A.; Dasyam A.; Papachristou G.I.; Slivka A.; Yadav D.	2019	Gastroenterology	156	6 Supplement 1	S-548	https://dx.doi.org/10.1016/S0016-5085%2819%2938260-5	Ooka 2019	NLP used only as a study adjunct/enabler
RISK OF METACHRONOUS COLORECTAL CANCER AMONG INDIVIDUALS WITH A HISTORY OF SERRATED POLYPS - DATA FROM A LARGE INTEGRATED HEALTHCARE SYSTEM	Li D.; Fevrier H.; Amsden L.B.; Doherty A.R.; Browne L.W.; Levin T.R.; Lee J.K.; Corley D.A.; Herrinton L.J.	2019	Gastroenterology	156	6 Supplement 1	S-149	https://dx.doi.org/10.1016/S0016-5085%2819%2937160-4	Li 2019	NLP used only as a study adjunct/enabler
HELICOBACTER PYLORI TESTING AND TREATMENT IN A LARGE, INTEGRATED HEALTHCARE DELIVERY SYSTEM IN THE UNITED STATES	Vakil N.; Michalopoulos S.N.; Zajichek A.; Wang T.; Bauman J.; Milinovich A.; Daly T.; Pierz K.A.; Storen J.T.; Kattan M.	2019	Gastroenterology	156	6 S1	S-524	https://dx.doi.org/10.1016/S0016-5085%2819%2938191-0	Vakil 2019	NLP used only as a study adjunct/enabler
A cross-sectional natural history of Parkinson's disease as reported by >10,000 patients	Shoulson I.; Arbatti L.; Marras C.; Standaert D.; Tanner C.; Smolensky L.;	2019	Journal of Parkinson's Disease	9	1	108	https://dx.doi.org/10.3233/JPD-199900	Shoulson 2019	Non-Gastro/Hepatology Focus

	Kopil C.; Hamilton J.; Flagg E.; Christopher C.A.; Nguyen A.								
Accuracy of a Natural Language Processing Pipeline to Identify Patient Symptoms during Radiation Therapy	Hong J.C.; Tanksley J.; Niedzwiecki D.; Palta M.; Tenenbaum J.D.	2019	International Journal of Radiation Oncology Biology Physics	105	1 Supplement	S70	https://dx.doi.org/10.1016/j.ijrobp.2019.06.522	Hong 2019	Non-Gastro/Hepatology Focus
PREVALENCE, INDICATIONS, AND OUTCOMES OF STACKED VENA CAVA FILTERS	Halkar M.G.; Ward R.; Koepsel E.M.K.; Bjarnason H.; McBane R.; Houghton D.	2019	Journal of the American College of Cardiology	73	9 Supplement 1	2074	https://dx.doi.org/10.1016/S0735-1097%2819%2932680-4	Halkar 2019	Non-Gastro/Hepatology Focus
How often do prescribers include indications in drug orders? Analysis of 4 million outpatient prescriptions	Salazar A.; Karmiy S.J.; Forsythe K.J.; Amato M.G.; Wright A.; Lai K.H.; Lambert B.L.; Liebovitz D.M.; Eguale T.; Volk L.A.; Schiff G.D.	2019	American Journal of Health-System Pharmacy	76	13	970-979	https://dx.doi.org/10.1093/ajhp/zxz082	Salazar 2019	Non-Gastro/Hepatology Focus
Weakly supervised natural language processing for assessing patient-centered outcome following prostate cancer treatment	Banerjee I.; Li K.; Seneviratne M.; Ferrari M.; Seto T.; Brooks J.D.; Rubin D.L.; Hernandez-Boussard T.	2019	JAMIA Open	2	1	150-159	https://dx.doi.org/10.1093/jamiaopen/ooy057	Banerjee 2019	Non-Gastro/Hepatology Focus
Embedding of genes using cancer gene expression data: Biological relevance and	Choy C.T.; Wong C.H.; Chan S.L.	2019	Frontiers in Genetics	10	JAN	682	https://dx.doi.org/10.3389/fgene.2018.00682	Choy 2019	Non-Gastro/Hepatology Focus

potential application on biomarker discovery									
Natural Language Processing, or How to Communicate With Your Computer	Moreno C.C.	2019	Journal of the American College of Radiology	16	11	1585-1586	https://dx.doi.org/10.1016/j.jacr.2019.07.007	Moreno 2019	Non-Gastro/Hepatology Focus
Using a Natural Language Processing and Machine Learning Algorithm Program to Analyze Inter-Radiologist Report Style Variation and Compare Variation Between Radiologists When Using Highly Structured Versus More Free Text Reporting	Donnelly L.F.; Grzeszczuk R.; Guimaraes C.V.; Zhang W.; Bisset III G.S.	2019	Current Problems in Diagnostic Radiology	48	6	524-530	https://dx.doi.org/10.1067/j.cpradiol.2018.09.005	Donnelly 2019	Non-Gastro/Hepatology Focus
The power of human touch in the era of artificial intelligence	Bianchi D.W.	2019	Pediatric Research	86	5	670-671	https://dx.doi.org/10.1038/s41390-019-0484-7	Bianchi 2019	Review/Perspective Article Only
Applications of machine learning in drug discovery and development	Vamathevan J.; Clark D.; Czodrowski P.; Dunham I.; Ferran E.; Lee G.; Li B.; Madabhushi A.; Shah P.; Spitzer M.; Zhao S.	2019	Nature Reviews Drug Discovery	18	6	463-477	https://dx.doi.org/10.1038/s41573-019-0024-5	Vamathevan 2019	Non-Gastro/Hepatology Focus

Natural language processing of symptoms documented in free-text narratives of electronic health records: A systematic review	Koleck T.A.; Dreisbach C.; Bourne P.E.; Bakken S.	2019	Journal of the American Medical Informatics Association	26	4	364-379	https://dx.doi.org/10.1093/jamia/ocy173	Koleck 2019	Review/Perspective Article Only
A systems model of liver physiology provides mechanistic insights from toxicogenomics data	Sharma T.; Chandarana P.; Priyadarsini S.; Bhat A.; Subramanian K.; Das S.	2019	International Journal of Toxicology	38	1	72	https://dx.doi.org/10.1177/1091581818818422	Sharma 2019	Non-NLP Focus
Comparison of approaches to the identification of symptom burden in hemodialysis patients utilizing electronic health records	Chan L.; Beers K.H.; Chauhan K.; Debnath N.; Pattharanitima P.; Coca S.G.; Van Vleck T.T.; Nadkarni G.N.	2019	Journal of the American Society of Nephrology	30	(Chan, Beers, Chauhan, Debnath, Pattharanitima, Coca, Van Vleck, Nadkarni) Icahn School of Medicine at Mount Sinai, New York, NY, United States	176		Chan 2019	Non-Gastro/Hepatology Focus
GWAS and enrichment analyses of primary sclerosing cholangitis and autoimmune hepatitis in pediatrics	Namjou B.; Lam S.; Singh R.; Miethke A.G.	2019	Hepatology v70 suppl.1 2019	70	Supplement 1	767A-768A		Namjou 2019	NLP used only as a study adjunct/enabler
Management of methanol-induced toxic optic	Mas Putrawati Triningrat A.A.; Utari N.M.L.;	2019	Clinical Toxicology	57	12	1183	https://dx.doi.org/10.1080/15563650.2019.1676025	MasPutrawatiTriningrat 2019	Non-NLP Focus

neuropathy with high dose corticosteroid and hyperbaric oxygen therapy	Ketut Agus Somia I.; Devi A.								
Prediction of pancreatic cancer based on duct abnormalities using prediction models	Chen W.; Butler R.K.; Zhou Y.; Parker R.A.; Jeon C.Y.; Wu B.U.	2019	Pancreas	48	10	1412	https://dx.doi.org/10.1097/MPA.0000000000001419	Chen 2019	Weak Validation Only (Type 1b or less)
Mortality in patients with chronic pancreatitis in a diverse integrated healthcare setting	Chen Q.; Wu B.U.	2019	Pancreas	48	10	1412	https://dx.doi.org/10.1097/MPA.0000000000001419	Chen 2019	NLP used only as a study adjunct/enabler
Identifying patients with rare diseases for research using billing codes: Is there a better way?	Lee J.; Hoffman J.; Hebert C.; Nuss K.; Hussain C.; Erdman S.	2019	Journal of Pediatric Gastroenterology and Nutrition	69	Supplement 2		https://dx.doi.org/10.1097/MPG.0000000000002518	Lee 2019	Non-Gastro/Hepatology Focus
Creation and external validation of a clinical prediction rule for diarrheal etiology using natural language processing	Howard J.I.; Aoki J.; Ferraro J.; Haaland B.; Pavia A.; Leung D.T.	2019	Open Forum Infectious Diseases	6	Supplement 2	S544	https://dx.doi.org/10.1093/ofid/ofz360.1357	Howard 2019	Lack of Validation
Percutaneous neprolithotomy: Results of our 50 first cases	Jimenez Navarro M.; Fumero Arteaga S.; Amador Robayna A.; Falcon Barroso J.; Ballesta Martinez B.;	2019	European Urology, Supplements	18	7	e2964	https://dx.doi.org/10.1016/S1569-9056%2819%2933124-0	JimenezNavarro 2019	Non-Gastro/Hepatology Focus

	Garcia Garcia L.; Gonzalez Nieto S.; Monllor Gisbert J.								
EP1.16-05 Real World Outcomes of Advanced NSCLC Patients with Liver Metastases	Law J.; Pettengell C.; Chen L.; Le L.; Sung M.; Aviv S.; Lau S.; Sacher A.; Merritt D.; Demarco P.; Leighl N.	2019	Journal of Thoracic Oncology	14	10 Supplement	S1066	https://dx.doi.org/10.1016/j.jtho.2019.08.2370	Law 2019	NLP used only as a study adjunct/enabler
Clinical practice guidelines: Machine learning and natural language processing for automating the rapid identification and annotation of new evidence	Whittington C.; Feinman T.; Lewis S.Z.; Lieberman G.; Del Aguila M.	2019	Journal of Clinical Oncology	37	Supplement 8		https://dx.doi.org/10.1200/JCO.2019.37.8_suppl.77	Whittington 2019	Non-Gastro/Hepatology Focus
Leveraging a conversational agent to support adherence to oral anticancer agents: A usability study	Berges B.M.; Cambareri C.; Takvorian S.U.; Serpa M.; Shulman L.N.; Bekelman J.E.; Rendle K.A.; Argon J.; Rosin R.	2019	Journal of Clinical Oncology	37	Supplement 15		https://dx.doi.org/10.1200/JCO.2019.37.15_suppl.6534	Berges 2019	Non-Gastro/Hepatology Focus
What bothers Parkinson disease patients? verbatim reports from >10,000 patients informing natural history and clinical trials	Shoulson I.; Arbatti L.; Marras C.; Standaert D.; Tanner C.M.; Smolensky L.; Kopil C.; Hamilton J.;	2019	Movement Disorders	34	Supplement 1	S16	https://dx.doi.org/10.1002/mds.27654	Shoulson 2019	Non-Gastro/Hepatology Focus

	Flagg E.; Christopher C.A.; Nguyen A.								
The use of natural language processing to assess patients with bleeds in their notes in the optum EHR database	Rubinstein E.; Spurden D.; Chhabra A.; Tortella B.; Senerchia C.; Alvir J.; Fogarty P.	2019	Haemophilia	25	Supplement 2	61	https://dx.doi.org/10.1111/hae.13786	Rubinstein 2019	Lack of Validation
Pelvic exenteration for non-colorectal malignancy requiring proctectomy: Surgical and oncologic outcomes	Cauley C.E.; Hassab T.; Jarrar A.; Camargo M.; Valente M.A.; Kalady M.; Gorgun E.; Steele S.	2019	Diseases of the Colon and Rectum	62	6	e258	https://dx.doi.org/10.1097/DCR.0000000000001415	Cauley 2019	Non-Gastro/Hepatology Focus
THE RELATIONSHIP OF INDIVIDUAL ENDOSCOPY VOLUME ON COMPLICATION RATES	Hernandez L.V.; Johannessen J.; Manahan T.; Guda N.M.; Manahan A.; Klyve D.	2019	Gastrointestinal Endoscopy	89	6 Supplement	AB428-AB429	https://dx.doi.org/10.1016/j.gie.2019.03.668	Hernandez 2019	Non-NLP Focus
INCORPORATION OF TEMPORAL INFORMATION IN A DEEP NEURAL NETWORK IMPROVES PERFORMANCE LEVEL FOR AUTOMATED POLYP DETECTION AND DELINEATION	Eelbode T.; Demedts I.; Bisschops R.; Roelandt P.; Hassan C.; Coron E.; Bhandari P.; Neumann H.; Pech O.; Repici A.; Maes F.	2019	Gastrointestinal Endoscopy	89	6 Supplement	AB618-AB619	https://dx.doi.org/10.1016/j.gie.2019.03.1075	Eelbode 2019	NLP used only as a study adjunct/enabler

Personalized pressure injury prevention planning: Clinical practice guidelines, the electronic health record and big data	Bogie K.M.; Henzel M.K.; Zhang G.Q.; Roggenkamp S.; Sun J.; Bloostein A.; Seton J.M.; Li Y.; Richmond M.A.; Washington M.	2019	Wound Repair and Regeneration	27	3	A12	https://dx.doi.org/10.1111/wrr.12711	Bogie 2019	Non-Gastro/Hepatology Focus
Evaluation of genetic variants in new-onset hidradenitis suppurativa patients by exome sequencing	Posch C.; Ahn R.; Wiala A.; Rappersberger K.; Liao W.	2019	Experimental Dermatology	28	3	e43	https://dx.doi.org/10.1111/exd.13859	Posch 2019	Non-Gastro/Hepatology Focus
Abstract No. 563 Machine learning techniques to translate follow-up imaging post RFA and TACE for HCC: toward greater shared decision making in IR clinic	Brown A.; Kachura J.	2019	Journal of Vascular and Interventional Radiology	30	3 Supplement	S243-S244	https://dx.doi.org/10.1016/j.jvir.2018.12.644	Brown 2019	Non-NLP Focus
Neuro-linguistic programming (NLP) in haemophilia practice	Ortelee M.; Balkestein F.; Mulders G.	2019	Haemophilia	25	Supplement 1	137	https://dx.doi.org/10.1111/hae.13666	Ortelee 2019	Non-Gastro/Hepatology Focus
Concluding thoughts	Bate A.	2018	Methods in Pharmacology and Toxicology		(Bate) Epidemiology Group Lead, Analytics, Worldwide Safety and Regulatory, Tadworth, Surrey, United	259-268		Bate 2018	Review/Perspective Article Only

					Kingdom(Bate) Division of Translational Medicine Department of Medicine, New York University New York, United States				
Patient narratives in Yelp reviews offer insight into opioid experiences and the challenges of pain management	Graves R.L.; Goldshear J.; Perrone J.; Ungar L.; Klinger E.; Meisel Z.F.; Merchant R.M.	2018	Pain Management	8	2	95-104	https://dx.doi.org/10.2217/pmt-2017-0050	Graves 2018	Non-Gastro/Hepatology Focus
Analysis of klebsiella as a prognostic factor of ocular outcomes in endogenous endophthalmitis with decision tree analysis	Jung H.; Kim S.-W.; Chang H.-H.; Lee S.-A.; Kim Y.; Hwang S.; Kim S.-J.; Lee J.-M.	2018	Infection and Chemotherapy	50	3	238-251	https://dx.doi.org/10.3947/ic.2018.50.3.238	Jung 2018	Non-Gastro/Hepatology Focus
A genotype-first approach identifies atypical PKD in DNAJB11 mutation carriers	Luo J.Z.; Mooore B.S.; Besse W.E.; Somlo S.; Chang A.R.; Mirshahi T.; Hartzel D.N.	2018	Journal of the American Society of Nephrology	29	(Luo, Mooore, Chang, Mirshahi) Geisinger, Danville, PA, United States(Besse, Somlo) Yale University, New Haven, CT, United States(Hartzel) Geisinger	290-291		Luo 2018	Non-Gastro/Hepatology Focus

					Health System, Danville, PA, United States				
using Natural Language Processing to Accurately Identify Dysplasia in Pathology Reports for Patients with Barrett's Esophagus	Thrift A.P.; Natarajan Y.; Mansour N.M.; Wang J.; Xu H.; Hou J.K.; El-Serag H.B.	2018	Gastroenterology	154	6 Supplement 1	S-897	https://dx.doi.org/10.1016/S0016-5085%2818%2933024-5	Thrift 2018	Part of Larger Study
Risk of Pancreatic Cancer in Patients with Newly Diagnosed Chronic Pancreatitis	Wu B.U.; Chung J.W.; Yu W.; Conwell D.L.; Yadav D.; Pandol S.J.	2018	Gastroenterology	154	6 Supplement 1	S-721	https://dx.doi.org/10.1016/S0016-5085%2818%2932520-4	Wu 2018	NLP used only as a study adjunct/enabler
PROFILES OF DISEASE IN CHRONIC PANCREATITIS	Wu B.U.; Chung J.W.; Yu W.; Conwell D.L.; Yadav D.; Pandol S.J.	2018	Gastroenterology	154	6 Supplement 1	S-720	https://dx.doi.org/10.1016/S0016-5085%2818%2932519-8	Wu 2018	NLP used only as a study adjunct/enabler
OUTCOMES IN VETERANS WITH ADVANCED COLORECTAL ADENOMAS	Imperiale T.F.; Myers L.; Imler T.D.; Sherer E.A.; Larson J.A.; Kahi C.J.	2018	Gastroenterology	154	6 Supplement 1	S-769	https://dx.doi.org/10.1016/S0016-5085%2818%2932663-5	Imperiale 2018	NLP used only as a study adjunct/enabler
OUTCOMES OF IMMUNOTHERAPY-INDUCED DIARRHEA AND COLITIS: A CANCER CENTER EXPERIENCE	Wang Y.; Abu-Sbeih H.; Mao E.; Ali N.; Ali F.; Qiao W.; Bresalier R.S.; Davila M.L.; Diab A.; Lum P.; Raju G.S.; Shuttlesworth G.A.; Richards D.M.; Stroehlein J.R.	2018	Gastroenterology	154	6 Supplement 1	S-1001	https://dx.doi.org/10.1016/S0016-5085%2818%2933350-X	Wang 2018	NLP used only as a study adjunct/enabler

Use of machine learning to determine stroke severity of patients diagnosed with stroke in claims data	Kogan E.; Twyman K.; Heap J.; Milentijevic D.; Lin J.H.; Chen Y.-W.; Alberts M.	2018	European Heart Journal	39	Supplement 1	44	https://dx.doi.org/10.1093/eurheartj/ehy564.P287	Kogan 2018	Non-Gastro/Hepatology Focus
Percutaneous nephrolithotomy in transplant patients	Ferreiro C.C.; Torrecilla C.; Riera L.; Fernandez-Concha J.; Colom S.; Vigues F.	2018	Journal of Endourology	32	Supplement 2	A63-A64	https://dx.doi.org/10.1089/end.2018.29043.abstracts	Ferreiro 2018	Non-Gastro/Hepatology Focus
Clinical and laboratory predictors of 30-day hospital readmission risk in adult patients with sickle cell disease	Nouraie S.M.; Saul M.; Novelli E.M.; Kato G.J.; Gladwin M.T.	2018	Blood	132	Suppl. 1		https://dx.doi.org/10.1182/blood-2018-99-115248	Nouraie 2018	Non-Gastro/Hepatology Focus
GENDER DIFFERENCES IN CLINICAL PROFILE AND HEALTHCARE UTILIZATION OF NON ALCOHOLIC FATTY LIVER PATIENTS	Goldshtein I.; Chodick G.; Shibolet O.	2018	Value in Health	21	Supplement 3	S146	https://dx.doi.org/10.1016/j.jval.2018.09.871	Goldshtein 2018	Non-NLP Focus
Problems that bother parkinson disease patients: Framework for a patient-reported natural history of Parkinson and Huntington diseases	Shoulson I.; Arbatti L.; Nguyen A.; Standaert D.; Marras C.; Tanner C.	2018	Neurotherapeutics	15	4	1209	https://dx.doi.org/10.1007/s13311-018-00673-5	Shoulson 2018	Non-Gastro/Hepatology Focus

The harvard necrotizing enterocolitis database: An artificial intelligence-friendly data repository with a unique ontology	Crowley P.A.; Crowley S.; Rogerson S.; Chou J.; Kohane I.	2018	Journal of Pediatric Gastroenterology and Nutrition	67	Supplement 1	S146	https://dx.doi.org/10.1097/MPG.0000000000002164	Crowley 2018	NLP used only as a study adjunct/enabler
Forty-four ways to describe a normal appendix: The importance of understanding onomasiology in gastroenterology pathology reports in the era of artificial intelligence	Crowley P.A.; Brociner E.	2018	Journal of Pediatric Gastroenterology and Nutrition	67	Supplement 1	S6-S7	https://dx.doi.org/10.1097/MPG.0000000000002164	Crowley 2018	Review/Perspective Article Only
The boston children's hospital appendicitis database: Development of a large, multimodal dataset optimized for deep learning	Crowley P.A.; Brociner E.; Kohane I.	2018	Journal of Pediatric Gastroenterology and Nutrition	67	Supplement 1	S300	https://dx.doi.org/10.1097/MPG.0000000000002164	Crowley 2018	Non-Gastro/Hepatology Focus
Problems that bother parkinson patients: The basis for a patient-reported natural history	Arbatti L.; Marras C.; Standaert D.; Tanner C.; Nguyen A.; Shoulson I.	2018	Annals of Neurology	84	Supplement 22	S205		Arbatti 2018	Non-Gastro/Hepatology Focus
Weight loss surgery significantly reduces the risk of liver-related morbidity in obese	Sharpton S.R.; Liu L.; Saxena V.; Herrinton L.J.; Terrault N.	2018	Hepatology	68	Supplement 1	995A-996A	https://dx.doi.org/10.1002/hep.30257	Sharpton 2018	Non-NLP Focus

adults with nonalcoholic fatty liver disease: A long-term cohort study									
Extra hepatic manifestation of non alcoholic fatty liver	Goldshtein I.; Shibolet O.; Chodick G.	2018	Hepatology	68	Supplement 1	989A	https://dx.doi.org/10.1002/hep.30257	Goldshtein 2018	NLP used only as a study adjunct/enabler
Risk of cirrhosis and HCC in patients with steatosis and normal aminotransferases	Natarajan Y.; Kramer J.R.; Yu X.; Wang J.; Xu H.; Kanwal F.	2018	Hepatology	68	Supplement 1	90A	https://dx.doi.org/10.1002/hep.30256	Natarajan 2018	NLP used only as a study adjunct/enabler
Utilizing natural language processing to identify intraoperative medical device failures and subsequant surgeon mitigation	Ghanem A.; Yoo A.	2018	Pharmacoepidemiology and Drug Safety	27	Supplement 2	217	https://dx.doi.org/10.1002/pds.4629	Ghanem 2018	Non-Gastro/Hepatology Focus
Patients with Inflammatory Bowel Disease (IBD) have, similar reproductive outcome compared to the general infertile population	Hernandez-Nieto C.; Sekhon L.; Gounko D.; Lee J.; Luna M.; Cervantes E.; Copperman A.; Sandler B.	2018	Human Reproduction	33	Supplement 1	i304-i305	https://dx.doi.org/10.1093/humrep/33.Supplement_1.1	Hernandez-Nieto 2018	NLP used only as a study adjunct/enabler
How to create an electronic database for hnpcc (lynch syndrome) from existing software	Thirumurthi S.; Pande M.; Lum P.; Bannon S.A.; Mork M.; Rodriguez-Bigas M.A.; You Y.N.;	2018	Gastrointestinal Endoscopy	87	6 Supplement 1	AB493-AB494		Thirumurthi 2018	NLP used only as a study adjunct/enabler

programs: MD anderson cancer center hnpcc surveillance outcomes, a step towards establishing quality metrics for high risk cancer patients	Sanchez E.V.; Lynch P.M.								
Use of natural language processing to identify 414 different chief complaints in adult emergency department patients	Thompson D.A.; Courtney D.M.; Malik S.; Schmidt M.; Weston V.	2018	Academic Emergency Medicine	25	Supplement 1	S193	https://dx.doi.org/10.1111/acem.13424	Thompson 2018	Non-Gastro/Hepatology Focus
"Why am i taking this medication doctor?": Failure to include indications in outpatient drug orders and instructions	Forsythe K.; Salazar A.; Karmiy S.; Amato M.; Wright A.; Volk L.A.; Neri Garabedian P.M.; Eguale T.; Lambert B.; Liebovitz D.M.; Schiff G.D.	2018	Journal of General Internal Medicine	33	2 Supplement 1	85-86		Forsythe 2018	Non-Gastro/Hepatology Focus
The first large scale registry for non alcoholic fatty liver disease in Israel	Goldshtein I.; Oren R.; Chodik G.	2018	Journal of Hepatology	68	Supplement 1	S161-S162		Goldshtein 2018	NLP used only as a study adjunct/enabler
Ovarian reserve is not compromised in patients with inflammatory bowel disease	Rekawek P.; Sekhon L.; Hernandez-Nieto C.; Lee J.A.; Mella M.T.;	2018	Fertility and Sterility	109	3	e50-e51		Rekawek 2018	NLP used only as a study adjunct/enabler

	Sandler B.; Copperman A.B.								
A High-throughput genetic analysis of common drug allergy labels using data from a large biobank	Phillips E.J.; Wei W.-Q.; Shaffer C.M.; Feng Q.P.; Stone C.A.; Stein C.M.; Roden D.M.; Denny J.C.	2018	Journal of Allergy and Clinical Immunology	141	2 Supplement 1	AB399		Phillips 2018	Non-Gastro/Hepatology Focus
Impact of extra-pancreatic cysts on risk of pancreatic cancer	Jaradeh K.; Sharib J.; Donovan I.; Bracci P.; Kirkwood K.	2018	Annals of Surgical Oncology	25	1 Supplement 1	S133	https://dx.doi.org/10.1245/s10434-018-6349-1	Jaradeh 2018	NLP used only as a study adjunct/enabler
ADEPt, a semantically-enriched pipeline for extracting adverse drug events from free-text electronic health records	Iqbal E.; Mallah R.; Rhodes D.; Wu H.; Romero A.; Chang N.; Dzahini O.; Pandey C.; Broadbent M.; Stewart R.; Dobson R.J.B.; Ibrahim Z.M.	2017	PLoS ONE	12	11	e0187121	https://dx.doi.org/10.1371/journal.pone.0187121	Iqbal 2017	Non-Gastro/Hepatology Focus
2017 ASCO Annual Meeting Abstracts	Anonymous.	2017	Journal of Clinical Oncology	35	31 Supplement 1			Anonymous 2017	Non-Gastro/Hepatology Focus
Characteristics of patients with irritable bowel syndrome with constipation and chronic idiopathic constipation and associated burden of illness in the United States	Abel J.; Icten Z.; Elder K.; Carson R.; Taylor D.; Reasner D.	2017	Journal of Managed Care and Specialty Pharmacy	23	3-A SUPPL.	S81		Abel 2017	NLP used only as a study adjunct/enabler
Clinical course of radiation-induced choroidal tumor	Augsburger J.J.; Skinner C.C.; Correa Z.M.	2017	Investigative Ophthalmology and Visual Science	58	8			Augsburger 2017	Non-Gastro/Hepatology Focus

vasculopathy with progressive exudative retinal detachment following plaque radiotherapy for primary posterior uveal melanoma									
Visual, surgical, and anatomic outcomes of consecutive open-globe injuries: A ten-year experience at a single institution	Zhang J.; Kavoussi S.; Alasil T.; Meskin S.; Adelman R.A.	2017	Investigative Ophthalmology and Visual Science	58	8			Zhang 2017	Non-Gastro/Hepatology Focus
New approaches for IBD management based on text mining of digitalised medical reports and latent class modelling	Bergey F.; Saccenti E.; Jonkers D.; Van Den Heuvel T.; Jeuring S.; Pierik M.; Martins Dos Santos V.	2017	Journal of Crohn's and Colitis	11	Supplement 1	S237-S238	https://dx.doi.org/10.1093/ecco-jcc/jjx002.441	Bergey 2017	NLP used only as a study adjunct/enabler
Ambulatory safety nets for lung and colon cancer to prevent missed and delayed diagnosis	Desai S.; Holtz L.; Sequist T.	2017	Diagnosis	4	4	eA56	https://dx.doi.org/10.1515/dx-2017-0034	Desai 2017	Non-NLP Focus
What is the expected incidence of interval colorectal cancer (CRC) for an endoscopist in active clinical practice?	Ertem F.U.; Mehrotra A.; Gourevitch R.A.; Ladabaum U.; Schoen R.E.	2017	Gastrointestinal Endoscopy	85	5 Supplement 1	AB93		Ertem 2017	NLP used only as a study adjunct/enabler
18F-FDG-PET imaging of	Xiao W.; Dong D.; Li D.; Han S.;	2017	European Journal of Nuclear	44	2 Supplement 1	S636	https://dx.doi.org/10.1007/s00259-017-3822-1	Xiao 2017	Non-NLP Focus

genetically engineered mouse models elucidates oncogenic function of Nlp and FAM135B	Yan H.; Wan L.; Xie Q.; Zhan Q.		Medicine and Molecular Imaging						
Physician characteristics associated with higher adenoma detection rates: What makes a good endoscopist?	Ezaz G.; Leffler D.; Gourevitch R.A.; Germansky K.A.; Schoen R.E.; Morris M.; Crockett S.; Carrell D.; Greer J.B.; Rose S.; Mehrotra A.	2017	Gastroenterology	152	5 Supplement 1	S216-S217		Ezaz 2017	NLP used only as a study adjunct/enabler
Identifying gastrointestinal symptoms within an electronic health record database among patients with type 2 diabetes treated with a GLP-1 agonist	Nunes A.P.; Seeger J.D.; Ezzy S.M.; Loughlin A.M.	2017	Pharmacoepidemiology and Drug Safety	26	Supplement 2	233-234	https://dx.doi.org/10.1002/pds.4275	Nunes 2017	Non-Gastro/Hepatology Focus
Characterization of potential serious allergic reactions using data from natural language processing (NLP) within an electronic health record (EHR) based system	Wang F.T.; Song J.; Lin N.D.	2017	Pharmacoepidemiology and Drug Safety	26	Supplement 2	37-38	https://dx.doi.org/10.1002/pds.4275	Wang 2017	Non-Gastro/Hepatology Focus
Utilizing natural language processing to	Lin T.-C.; Cai T.; Kane-Wanger G.; Cagan A.;	2017	Pharmacoepidemiology and Drug Safety	26	Supplement 2	35-36	https://dx.doi.org/10.1002/pds.4275	Lin 2017	Lack of Validation

examine the risk of arthralgia between vedolizumab and tumor necrosis factor inhibitors in inflammatory bowel disease	Murphy S.N.; Ananthakrishnan A.; Liao K.P.								
Creation of a quality-improvement database for transurethral resection of bladder tumors	Cohen J.; Glaser A.; Okorji L.; Oberlin D.; Meeks J.	2017	Journal of Urology	197	4 Supplement 1	e115		Cohen 2017	Non-Gastro/Hepatology Focus
Using natural language processing to automate grading of student's patient notes: A pilot study of machine learning text classification	Kalet A.; Oh S.-Y.; Marin M.; Yu Y.; Dumorne H.; Aphinyanaphongs Y.	2017	Journal of General Internal Medicine	32	2 Supplement 1	S369-S370		Kalet 2017	Non-Gastro/Hepatology Focus
A pilot study of the Vigi4MED project: Comparison of adverse drug reactions (ADRs) of duloxetine between patients' forum posts and the French pharmacovigilance database (FPVD)	Boeuf M.; Bellet F.; Karapetiantz P.; Leprovost D.; Morlane-Hondere F.; Grouin C.; Audeh B.; Bousquet C.; Beyens M.N.	2017	Fundamental and Clinical Pharmacology	31	Supplement 1	33		Boeuf 2017	Non-Gastro/Hepatology Focus
The role of deoxycholic acid in liver	Gutierrez-Gutierrez J.; Sanchez Del Arco R.; Gullon	2016	International Journal of Artificial Organs	39	7	368	https://dx.doi.org/10.5301/ijao.5000508	Gutierrez-Gutierrez 2016	Non-NLP Focus

normothermic perfusion	L.; Chuvieco S.; Fernandez I.; Asencio J.; Del Canizo J.								
Role of prostaglandins in normothermic liver machine perfusion	Rivera A.; Sierra A.; Chuvieco S.; Fernandez-Lopez I.; Gutierrez-Gutierrez J.; Sanchez Del Arco R.; Gullon L.; Asencio J.; Del Canizo J.	2016	International Journal of Artificial Organs	39	7	367-368	https://dx.doi.org/10.5301/ijao.5000508	Rivera 2016	Non-NLP Focus
Racial differences in clinical characteristics of heart failure with preserved ejection fraction	Patel Y.R.; Kurgansky K.E.; Imran T.F.; Orkaby A.R.; Cho K.; Gagnon D.R.; Ho Y.; Gaziano J.M.; Djousse L.; Joseph J.	2016	Cardiology (Switzerland)	134	Supplement 1	424	https://dx.doi.org/10.1159/000447505	Patel 2016	Non-Gastro/Hepatology Focus
Natural language processing to rapidly identify potential signals for adverse events using electronic medical record data: Example of arthralgias and vedolizumab	Cai T.; Kane-Wanger G.; Bond A.; Cagan A.; Murphy S.N.; Ananthakrishnan A.; Liao K.	2016	Arthritis and Rheumatology	68	Supplement 10	2802-2804	https://dx.doi.org/10.1002/art.39977	Cai 2016	Non-Gastro/Hepatology Focus
Feasibility and accuracy of translating a patient safety quality measure into an automated e-measure	Tonner C.; Schmajuk G.; Trupin L.; Yazdany J.	2016	Arthritis and Rheumatology	68	Supplement 10	2627-2629	https://dx.doi.org/10.1002/art.39977	Tonner 2016	Non-Gastro/Hepatology Focus

Analysis of patient narratives in disease blogs: Enhancing pharmacovigilance using real-world health data on the internet	Matsuda S.; Aoki K.; Tomizawa S.; Sone M.; Satomi S.; Nakayama T.; Tanaka R.; Kuriki H.; Takahashi Y.	2016	Pharmacoepidemiology and Drug Safety	25	Supplement 3	501	https://dx.doi.org/10.1002/pds.4070	Matsuda 2016	Non-Gastro/Hepatology Focus
Pro instruments used in studies published since 2005: Which populations and diseases have had the most tools developed?	Martin A.	2016	Value in Health	19	7	A486		Martin 2016	Non-Gastro/Hepatology Focus
Risk factors for advanced colorectal neoplasia in veterans	Imperiale T.F.; Imler T.; Sherer E.A.; Kahi C.; Larson J.; Cardwell J.; Johnson C.S.; Antaki F.; Ashley C.; Baffy G.; Cho I.; Dominitz J.A.; Hou J.K.; Korsten M.A.; Nagar A.B.; Patel S.; Promrat K.; Robertson D.; Saini S.; Shaw R.; Shergill A.; Smalley W.E.	2016	American Journal of Gastroenterology	111	Supplement 1	S136-S137	https://dx.doi.org/10.1038/ajg.2016.353	Imperiale 2016	Non-NLP Focus
A case-study using big data to increase scientific productivity in cancer research	Beird H.; Goldstein J.B.; Zhang J.; Narayanan S.; Belmont C.; Lari	2016	Journal of Clinical Oncology	34	Supplement 15			Beird 2016	Weak Validation Only (Type 1b or less)

	B.; Fassett R.; Pathak D.; Akella P.; Jin J.; Barbosa G.; Kell T.; Punugoti V.; Suh E.; Smith B.; Futreal A.								
Natural language processing (NLP) software use in the discovery of incidental lung cancers	Johnson M.L.; Blakemore B.E.; Baxter T.M.; Ashiq J.; Moore S.P.; Smith P.G.; Stults D.M.; Burris H.A.; Spigel D.R.	2016	Journal of Clinical Oncology	34	Supplement 15			Johnson 2016	Non-Gastro/Hepatology Focus
Identification of persons with congenital hemophilia in a large electronic health record database	Wang M.; Cyhaniuk A.; Cooper D.L.; Iyer N.N.	2016	Value in Health	19	3	A80		Wang 2016	Non-Gastro/Hepatology Focus
Adenoma detection rates correlate with sessile serrated polyp detection rates	Nayor J.; Goryachev S.; Gainer V.S.; Saltzman J.R.	2016	Gastrointestinal Endoscopy	83	5 SUPPL. 1	AB549		Nayor 2016	Non-NLP Focus
Our 9 year experience in peri-ampullary and non-ampullary duodenal adenomas: A retrospective study	Sherid M.; Afghani Z.; Bhagatwala J.; Lee J.E.; Sifuentes H.; Sridhar S.	2016	Gastrointestinal Endoscopy	83	5 SUPPL. 1	AB469		Sherid 2016	Lack of Validation
Using natural language processing to automate grading	Gershgorin I.; Marin M.; Xu J.; Oh S.-Y.; Zabar S.; Crowe R.;	2016	Journal of General Internal Medicine	31	2 SUPPL. 1	S458		Gershgorin 2016	Non-Gastro/Hepatology Focus

of students' patient notes: Proof of concept	Tewksbury L.; Ogilvie J.; Gillespie C.; Cantor M.; Aphinyanaphongs Y.; Kalet A.								
A novel voice-activated web application for rapid knowledge generation and information retrieval through semantic parsing of verbal communication	Metwally O.N.; Sinha S.R.	2016	Gastroenterology	150	4 SUPPL. 1	S433		Metwally 2016	Non-Gastro/Hepatology Focus
Guideline adherence evaluation through Barrett's Esophagus reporting	Shi S.; Makker J.; Jeffrey C.; Esrailian E.; Hommes D.; Yu C.	2016	Gastroenterology	150	4 SUPPL. 1	S262		Shi 2016	NLP used only as a study adjunct/enabler
Cross-cultural analysis of GI symptom expression between caucasians and Chinese	Herndon C.; Ho Siah T.K.; Pritzker S.; Stains J.; Ann G.K.; Mayer E.A.	2016	Gastroenterology	150	4 SUPPL. 1	S253		Herndon 2016	Non-NLP Focus
Prevalence of opioid analgesic use among patients presenting to emergency department with abdominal pain that is investigated with emergent abdominal CT scan	Khemani D.; Roldan A.; Nelson A.D.; Park S.-Y.; Acosta A.; Camilleri M.	2016	Gastroenterology	150	4 SUPPL. 1	S192-S193		Khemani 2016	Non-Gastro/Hepatology Focus

Patient understanding of the risks and benefits of biologic therapies in inflammatory bowel disease (IBD): Insights from a large-scale analysis of social media platforms	Martinez B.M.; Almario C.V.; Dailey F.; Desai M.P.; Dupuy T.; Mosadeghi S.; Whitman C.B.; Lasch K.; Ursos L.; Spiegel B.	2016	Gastroenterology	150	4 SUPPL. 1	S167		Martinez 2016	NLP used only as a study adjunct/enabler
Comparison of an electric pulse lithotripter to the holmium laser: Stone fragmentation efficiency and impact on flexible ureteroscope deflection and flow	Kaplan A.; Chen T.; Shin R.; Dale J.; Cabrera F.; Radvak D.; Ackerman A.; Sankin G.; Zhong P.; Scales Jr. C.; Ferrandino M.; Simmons N.; Preminger G.; Lipkin M.	2016	Journal of Urology	195	4 SUPPL. 1	e408		Kaplan 2016	Non-Gastro/Hepatology Focus
Clinicians' reports in electronic health records versus patients' concerns in social media: A pilot study of adverse drug reactions of aspirin and atorvastatin	Topaz M.; Lai K.; Dhopeshwarkar N.; Seger D.L.; Sa'Adon R.; Goss F.; Rozenblum R.; Zhou L.	2016	Drug Safety	39	3	243-250	https://dx.doi.org/10.1007/s40264-015-0381-x	Topaz 2016	Non-Gastro/Hepatology Focus
Natural language processing in pathology: A scoping review	Burger G.; Abu-Hanna A.; De Keizer N.; Cornet R.	2016	Journal of Clinical Pathology	69	11	949-955	https://dx.doi.org/10.1136/jclinpath-2016-203872	Burger 2016	Review/Perspective Article Only
Machine Learning and Network Methods for	Chen L.; Huang T.; Lu C.; Lu L.; Li D.	2015	Computational and Mathematical	2015	(Chen) College of Information	915124	https://dx.doi.org/10.1155/2015/915124	Chen 2015	Non-Gastro/Hepatology Focus

Biology and Medicine			Methods in Medicine		Engineering, Shanghai Maritime University, Shanghai 201306, China(Huang) Department of Genetics and Genomics Sciences, Mount Sinai School of Medicine, New York, NY 10029, United States(Lu) Institute of Health Sciences, Shangh				
A federated network for translational cancer research using clinical data and biospecimens	Jacobson R.S.; Becich M.J.; Bollag R.J.; Chavan G.; Corrigan J.; Dhir R.; Feldman M.D.; Gaudioso C.; Legowski E.; Maihle N.J.; Mitchell K.; Murphy M.; Sakthivel M.; Tseytlin E.; Weaver J.	2015	Cancer Research	75	24	5194-5201	https://dx.doi.org/10.1158/0008-5472.CAN-15-1973	Jacobson 2015	Non-Gastro/Hepatology Focus
Prevalence and characteristics of pancreatic cystic	Michailidis L.; Aslam B.;	2015	American Journal of Gastroenterology	110	Supplement 1	S903		Michailidis 2015	NLP used only as a study

lesions in patients with cirrhosis	Grigorian A.; Mardini H.								adjunct/enabler
A rare case of hypoplastic pancreas presenting as normal lipase pancreatitis	Vargas J.V.; Mellone J.; Soto L.; Gonzales L.; Tabrez S.	2015	American Journal of Gastroenterology	110	Supplement 1	S69		Vargas 2015	Non-NLP Focus
Perforating wounds observed healthy corneas: A one year observatory report	Famery N.; Romain N.; Bourges J.-L.; Brezin A.P.	2015	Investigative Ophthalmology and Visual Science	56	7	6048		Famery 2015	Non-Gastro/Hepatology Focus
Nucleobindin-1 (NUCB1) encodes an insulinotropic nesfatin-1-like peptide	Ramesh N.; Mohan H.; Unniappan S.	2015	Endocrine Reviews	36	Supplement 2			Ramesh 2015	Non-NLP Focus
Dili and diagnosis tools improvements	Babai S.; Le Louet H.	2015	Drug Safety	38	10	966	https://dx.doi.org/10.1007/s40264-015-0346-0	Babai 2015	Review/Perspective Article Only
Rapid identification of familial hypercholesterolemia from electronic health records	Safarova M.S.; Liu H.; Kullo I.J.	2015	Circulation	132	SUPPL. 3			Safarova 2015	Non-Gastro/Hepatology Focus
Identification of persons with acquired hemophilia in a large electronic health record database	Wang M.; Cyhaniuk A.; Cooper D.L.; Iyer N.N.	2015	Blood	126	23	3271		Wang 2015	Non-Gastro/Hepatology Focus
Prevalence of gastrointestinal symptoms in young people with	Serrano-Drozdowskyj E.; Penzol M.; Fernandez-Diaz A.; Alcon A.;	2015	European Neuropsychopharmacology	25	SUPPL. 2	S205		Serrano-Drozdowskyj 2015	Non-NLP Focus

autism spectrum disorder	Fraguas D.; Alvarez-Calatayud G.; Parrellada M.								
Validity of cancer diagnoses in general practitioner medical records	Fortuny J.; Kaye J.A.; Margulis A.V.; Plana E.; Calingaert B.; Perez-Gutthann S.; Arana A.	2015	Pharmacoepidemiology and Drug Safety	24	SUPPL. 1	513-514	https://dx.doi.org/10.1002/pds.3838	Fortuny 2015	Non-Gastro/Hepatology Focus
Human-algorithm interaction to define variables from free-text notes in electronic health records-introduction and examples	Dore D.D.; Nunes A.P.; Yee C.; Walker A.M.	2015	Pharmacoepidemiology and Drug Safety	24	SUPPL. 1	46-47	https://dx.doi.org/10.1002/pds.3838	Dore 2015	Non-Gastro/Hepatology Focus
Outcomes of surgical resection for benign colorectal neoplasms: An analysis of 359 resections at a single academic medical center	Keswani R.N.; Law R.; Lo A.; Grande D.; Thompson W.K.	2015	Gastrointestinal Endoscopy	81	5 SUPPL. 1	AB283-AB284	https://dx.doi.org/10.1016/j.gie.2015.03.389	Keswani 2015	NLP used only as a study adjunct/enabler
Five-year trends in adenoma detection ratES: A report from a single academic group practice	Miller E.; Thirumurthi S.; Lum P.; Pande M.; Ross W.A.; Davila M.L.; Weston B.R.; Blechacz B.; Bhutani M.S.; Lee J.; Lynch P.M.; Shafi M.A.; Bresalier R.S.; Mishra L.;	2015	Gastrointestinal Endoscopy	81	5 SUPPL. 1	AB213	https://dx.doi.org/10.1016/j.gie.2015.03.203	Miller 2015	NLP used only as a study adjunct/enabler

	Stroehlein J.R.; Raju G.S.								
When patients watch a video, physicians see more adenomas: An educational bowel preparation video improves adenoma detection RATES	Thirumurthi S.; Ross W.A.; Lum P.; Pande M.; Miller E.; Lee J.; Weston B.R.; Lynch P.M.; Davila M.L.; Bhutani M.S.; Shafi M.A.; Blechacz B.; Bresalier R.S.; Stroehlein J.R.; Mishra L.; Raju G.S.	2015	Gastrointestinal Endoscopy	81	5 SUPPL. 1	AB228-AB229	https://dx.doi.org/10.1016/j.gie.2015.03.1308	Thirumurthi 2015	NLP used only as a study adjunct/enabler
Natural Language Processing for Detection of Quality Measures in Endoscopic Retrograde Cholangiopancreatography	Imler T.D.; Sherman S.; Cote G.A.	2015	Gastrointestinal Endoscopy	81	5 SUPPL. 1	AB198-AB199	https://dx.doi.org/10.1016/j.gie.2015.03.171	Imler 2015	Non-NLP Focus
Sustained virologic response (SVR) and liver disease progression after hepatitis c virus (HCV) treatment initiation: A real world analysis using a large electronic health record (EHR) database	LaMori J.C.; Forlenza J.B.; Nelson S.; Cao F.; Liu F.; Song R.; Tandon N.; Buikema A.R.	2015	Gastroenterology	148	4 SUPPL. 1	S1098		LaMori 2015	NLP used only as a study adjunct/enabler
A natural language processing alogrithm for identification of	Kung R.; Ma A.; Dever J.B.; Vadivelu J.; Cherk E.; Koola	2015	Gastroenterology	148	4 SUPPL. 1	S1071-S1072		Kung 2015	Lack of Validation

patients with cirrhosis from electronic medical records	J.D.; Groessl E.J.; Matheny M.E.; Ho S.B.								
Analysis of outcomes after non-medical switching of anti-tumor necrosis factor agents	Rubin D.T.; Skup M.; Johnson S.J.; Chao J.; Gibofsky A.	2015	Gastroenterology	148	4 SUPPL. 1	S853		Rubin 2015	Non-NLP Focus
Performing against the benchmark: Does adenoma detection equally reflect proximal serrated polyp detection in individual endoscopists? Analysis of a large single high volume center using natural language processing	Patel V.D.; Thompson W.K.; Anand V.; Gawron A.J.; Goldstein J.L.; Bianchi L.K.; Yen E.F.	2015	Gastroenterology	148	4 SUPPL. 1	S755		Patel 2015	NLP used only as a study adjunct/enabler
Epicc (electronic pancreatic cyst clinic) for epic: A live electronic medical record based pancreatic cystic neoplasm clinical and research registry	Solad Y.; Kashyap N.; Hsiao A.L.; Farrell J.J.	2015	Gastroenterology	148	4 SUPPL. 1	S525		Solad 2015	Non-NLP Focus
Serrated lesion detection rate-an emerging marker of quality that varies with definition	Patel V.D.; Thompson W.K.; Anand V.; Goldstein J.L.; Bianchi L.K.; Yen E.F.	2015	Gastroenterology	148	4 SUPPL. 1	S126		Patel 2015	NLP used only as a study adjunct/enabler

Natural history after acute necrotizing pancreatitis (NP): A large U.S. tertiary care experience	Umapathy C.; Raina A.; Saligram S.; Papachristou G.I.; Rabinovitz M.; Chennat J.; Zeh H.; Zureikat A.H.; Hogg M.E.; Schraut W.H.; Lee K.; Saul M.I.; Whitcomb D.C.; Slivka A.; Yadav D.	2015	Gastroenterology	148	4 SUPPL. 1	S113		Umapathy 2015	NLP used only as a study adjunct/enabler
Prevalence of advanced colorectal neoplasia in veterans: Effects of age, sex and race	Imperiale T.F.; Imler T.D.; Kahi C.J.; Larson J.; Cardwell J.; Johnson C.S.; Ahnen D.J.; Antaki F.; Ashley C.; Baffy G.; Cho I.; Dominitz J.A.; Hou J.K.; Korsten M.A.; Nagar A.B.; Promrat K.; Robertson D.J.; Saini S.D.; Shergill A.; Smalley W.E.	2015	Gastroenterology	148	4 SUPPL. 1	S95		Imperiale 2015	Part of Larger Study
Identifying Patients at Risk for Fibrosis in a Primary Care NAFLD Cohort	Schreiner, A.D.; Livingston, S.; Zhang, J.; Gebregziabher, M.; Marsden, J.; Koch, D.G.; Petz, C.A.; Durkalski-Mauldin, V.L.;	2023	J. Clin. Gastroenterol.	57	1	89-96	10.1097/MCG.0000000000001585	Schreiner 2023	Non-NLP Focus

	Mauldin, P.D.; Moran, W.P.								
An AI Approach for Identifying Patients with Cirrhosis	Obeid, J.S.; Khalifa, A.; Xavier, B.; Bou-Daher, H.; Rockey, D.C.	2023	J. Clin. Gastroenterol.	57	1	82-88	10.1097/MCG.0000000000001586	Obeid 2023	NLP used only as a study adjunct/enabler
Next generation phenotyping with quantitative narration for DEGCAGS syndrome	Freeman, R.; Noronha, A.; Woods, J.	2023	Am. J. Med. Genet. Part A				10.1002/ajmg.a.63111	Freeman 2023	Non-Gastro/Hepatology Focus
NASH cirrhosis trials and major adverse liver outcomes: Big data needed	Noureddin, M.; Harrison, S.A.	2023	J. Hepatol.	78	1	05-Jul	10.1016/j.jhep.2022.10.022	Noureddin 2023	Review/Perspective Article Only
7th International Conference on ICT for Sustainable Development , ICT4SD 2022		2023	Lect. Notes Networks Syst.	520					Non-Gastro/Hepatology Focus
Monitoring colonoscopy screening program quality at the provider level, the institution level, and beyond	Melson, J.	2023	Gastrointest. Endosc.	97	1	130-131	10.1016/j.gie.2022.08.044	Melson 2023	Review/Perspective Article Only
DxGenerator: An Improved Differential Diagnosis Generator for Primary Care Based on MetaMap and Semantic Reasoning	Sanaeifar, A.; Eslami, S.; Ahadi, M.; Kahani, M.; Vakili Arki, H.	2022	Methods Inf. Med.	61	05-Jun	174-184	10.1055/a-1905-5639	Sanaeifar 2022	Non-Gastro/Hepatology Focus
ASO Visual Abstract:	Kooragayala, K.; Crudeli, C.;	2022	Ann Surg Oncol	29	13	8522	10.1245/s10434-022-12430-2	Kooragayala 2022	Abstract Only

Utilization of Natural Language Processing Software to Identify Worrisome Pancreatic Lesions	Kalola, A.; Bhat, V.; Lou, J.; Sensenig, R.; Atabek, U.; Echeverria, K.; Hong, Y.								
Superoxide-producing thermostable associate from the small intestines of control and alloxan-induced diabetic rats: quantitative and qualitative changes	Simonyan, R.M.; Simonyan, K.V.; Simonyan, G.M.; Khachatryan, H.S.; Babayan, M.A.; Danielyan, M.H.; Darbinyan, L.V.; Simonyan, M.A.	2022	BMC Endocr. Disord.	22	1		10.1186/s12902-022-01160-x	Simonyan 2022	Non-NLP Focus
Natural Language Processing for Automated Classification of Qualitative Data From Interviews of Patients With Cancer	Fang, C.; Markuzon, N.; Patel, N.; Rueda, J.-D.	2022	Value Health	25	12	1995-2002	10.1016/j.jval.2022.06.004	Fang 2022	Non-Gastro/Hepatology Focus
Imaging-Based Prevalence of Oligometastatic Disease: A Single-Center Cross-Sectional Study	Christ, S.M.; Pohl, K.; Muehlematter, U.J.; Heesen, P.; KÃ¼hnis, A.; Willmann, J.; Ahmadsei, M.; Badra, E.V.; Kroeze, S.G.C.; Mayinger, M.; Andratschke, N.; Huellner, M.;	2022	Int. J. Radiat. Oncol. Biol. Phys.	114	4	596-602	10.1016/j.ijrobp.2022.06.100	Christ 2022	Non-NLP Focus

	Guckenberger, M.								
The prevalence and spectrum of reported incidental adrenal abnormalities in abdominal computed tomography of cancer patients: The experience of a comprehensive cancer center	Qdaisat, A.; Bedrose, S.; Ezzeldin, O.; Moawad, A.W.; Yeung, S.-C.J.; Elsayes, K.M.; Habra, M.A.	2022	Front. Endocrinol.	13			10.3389/fendo.2022.1023220	Qdaisat 2022	Non-Gastro/Hepatology Focus
A deep learning and natural language processing-based system for automatic identification and surveillance of high-risk patients undergoing upper endoscopy: A multicenter study	Li, J.; Hu, S.; Shi, C.; Dong, Z.; Pan, J.; Ai, Y.; Liu, J.; Zhou, W.; Deng, Y.; Li, Y.; Yuan, J.; Zeng, Z.; Wu, L.; Yu, H.	2022	eClinicalMedicine	53			10.1016/j.eclinm.2022.101704	Li 2022	Non-NLP Focus
Prediction, Discovery, and Characterization of Plant- and Food-Derived Health-Beneficial Bioactive Peptides	Kussmann, M.	2022	Nutrients	14	22		10.3390/nu14224810	Kussmann 2022	Non-Gastro/Hepatology Focus
A network paradigm predicts drug synergistic effects using downstream proteinâ€“protein interactions	Wilson, J.L.; Steinberg, E.; Racz, R.; Altman, R.B.; Shah, N.; Grimes, K.	2022	CPT Pharmacometrics Syst. Pharmacol.	11	11	1527-1538	10.1002/psp4.12861	Wilson 2022	Non-Gastro/Hepatology Focus

The use of large patient databases to improve disease understanding and care	Paul, C.	2022	Br. J. Dermatol.	187	5	638	10.1111/bjd.21853	Paul 2022	Review/Perspective Article Only
Clinical course of non-alcoholic fatty liver disease and the implications for clinical trial design	Allen, A.M.; Therneau, T.M.; Ahmed, O.T.; Gidener, T.; Mara, K.C.; Larson, J.J.; Canning, R.E.; Benson, J.T.; Kamath, P.S.	2022	J. Hepatol.	77	5	1237-1245	10.1016/j.jhep.2022.07.004	Allen 2022	Non-NLP Focus
Paradoxical reactions and biologic agents: a French cohort study of 9303 patients*	Bataille, P.; Layese, R.; Claudepierre, P.; Paris, N.; Dubiel, J.; Amiot, A.; Sbidian, E.; AP-HP/Universities/Inserm COVID-19 research collaboration and on behalf of the 'Entrepot de Donnees de Sante' AP-HP consortium	2022	Br. J. Dermatol.	187	5	676-683	10.1111/bjd.21716	Bataille 2022	Non-NLP Focus
Systematic review of artificial intelligence-based image diagnosis for inflammatory bowel disease	Kawamoto, A.; Takenaka, K.; Okamoto, R.; Watanabe, M.; Ohtsuka, K.	2022	Dig. Endosc.	34	7	1311-1319	10.1111/den.14334	Kawamoto 2022	Review/Perspective Article Only
Gut commensal E. coli outer membrane proteins activate	Geng, S.; Li, Q.; Zhou, X.; Zheng, J.; Liu, H.; Zeng, J.; Yang, R.; Fu,	2022	Cell Host and Microbe	30	10	1401-1416.e8	10.1016/j.chom.2022.08.004	Geng 2022	Non-NLP Focus

the host food digestive system through neural-immune communication	H.; Hao, F.; Feng, Q.; Qi, B.								
Cross-Domain Text Mining to Predict Adverse Events from Tyrosine Kinase Inhibitors for Chronic Myeloid Leukemia	Mehra, N.; Varmeziar, A.; Chen, X.; Kronick, O.; Fisher, R.; Kota, V.; Mitchell, C.S.	2022	Cancers	14	19		10.3390/cancers14194686	Mehra 2022	Non-Gastro/Hepatology Focus
Artificial Intelligence in Inflammatory Bowel Disease	Stidham, R.W.	2022	Gastroenterol. Hepatol.	18	10	602-605		Stidham 2022	Review/Perspective Article Only
Popliteal cysts are not a risk factor for lower extremity deep vein thrombosis	Daniels, P.; Vlazny, D.; Meverden, R.; Bartlett, M.; Hesley, G.; Lekah, A.; Macedo, T.; Wysokinski, W.E.; Houghton, D.E.	2022	J. Thromb. Thrombolysis	54	3	492-499	10.1007/s11239-022-02685-7	Daniels 2022	Non-Gastro/Hepatology Focus
Erratum: Identifying Patients Who Meet Criteria for Genetic Testing of Hereditary Cancers Based on Structured and Unstructured Family Health History Data in the Electronic Health Record: Natural Language	Shi, J.; Morgan, K.L.; Bradshaw, R.L.; Jung, S.-H.; Kohlmann, W.; Kaphingst, K.A.; Kawamoto, K.; Del Fiol, G.	2022	JMIR Med. Inform.	10	9		10.2196/42533	Shi 2022	Part of Larger Study

Processing Approach (JMIR Med Inform (2022) 10:8 (e									
Overview of PAR-MEX at Iberlef 2022: Paraphrase Detection in Spanish Shared Task	Bel-Enguix, G.; Sierra, G.; GÃ³mez-Adorno, H.; Torres-Moreno, J.-M.; Ortiz-Barajas, J.-G.; VÃ¡squez, J.	2022	Proces. Lenguaje Nat.	69		255-263	10.26342/2022-69-22	Bel-Enguix 2022	Non-Gastro/Hepatology Focus
Classifying unstructured electronic consult messages to understand primary care physician specialty information needs	Ding, X.; Barnett, M.; Mehrotra, A.; Tuot, D.S.; Bitterman, D.S.; Miller, T.A.	2022	J. Am. Med. Informatics Assoc.	29	9	1607-1617	10.1093/jamia/ocac092	Ding 2022	Non-Gastro/Hepatology Focus
Pre-endoscopy coronavirus disease 2019 screening and severe acute respiratory syndrome coronavirus-2 nucleic acid amplification testing in the Veterans Affairs healthcare system: clinical practice patterns, outcomes, and relationship to procedure volume	Gawron, A.J.; Sultan, S.; Glorioso, T.J.; Califano, S.; Kralovic, S.M.; Jones, M.; Kirsh, S.; Dominitz, J.A.	2022	Gastrointest. Endosc.	96	3	423-432.e7	10.1016/j.gie.2022.04.018	Gawron 2022	Non-Gastro/Hepatology Focus

DRCNNTLe: A deep recurrent convolutional neural network with transfer learning through pre-trained embeddings for automated ICD coding	Raz Bhutto, S.; Wu, Y.; Zeng, M.; Wahab Dogar, A.; Ullah, K.; Li, M.	2022	Methods	205		97-105	10.1016/j.ymeth.2022.06.004	RazBhutto 2022	Non-Gastro/Hepatology Focus
Assessing the impact of OCR noise on multilingual event detection over digitised documents	Boros, E.; Nguyen, N.K.; Lejeune, G.; Doucet, A.	2022	Int. J. Digital Libr.	23	3	241-266	10.1007/s00799-022-00325-2	Boros 2022	Non-Gastro/Hepatology Focus
Patient Perspectives on Medical Trauma Related to Inflammatory Bowel Disease	Pothemont, K.; Quinton, S.; Jayoushe, M.; Jedel, S.; Bedell, A.; Hanauer, S.B.; Mutlu, E.A.; Taft, T.H.	2022	J. Clin. Psychol. Med. Settings	29	3	596-607	10.1007/s10880-021-09805-0	Pothemont 2022	Non-NLP Focus
Research trends of artificial intelligence in pancreatic cancer: a bibliometric analysis	Yin, H.; Zhang, F.; Yang, X.; Meng, X.; Miao, Y.; Noor Hussain, M.S.; Yang, L.; Li, Z.	2022	Front. Oncol.	12			10.3389/fonc.2022.973999	Yin 2022	Review/Perspective Article Only
In Vivo Evaluation of an Antibody-Functionalized Lipoidal Nanosystem for Schistosomiasis Intervention	Adekiya, T.A.; Kumar, P.; Kondiah, P.P.D.; Choonara, Y.E.	2022	Pharmaceutics	14	8		10.3390/pharmaceutics14081531	Adekiya 2022	Non-Gastro/Hepatology Focus
DLKN-MLC: A Disease Prediction	Li, B.; Zhang, Y.; Wu, X.	2022	Int. J. Environ. Res. Public Health	19	15		10.3390/ijerph19159771	Li 2022	Non-Gastro/Hepatology Focus

Model via Multi-Label Learning									
Identifying Patients With Inflammatory Bowel Disease on Twitter and Learning From Their Personal Experience: Retrospective Cohort Study	Stemmer, M.; Parmet, Y.; Ravid, G.	2022	J. Med. Internet Res.	24	8		10.2196/29186	Stemmer 2022	Lack of Validation
Role of Disease Progression Models in Drug Development	Barrett, J.S.; Nicholas, T.; Azer, K.; Corrigan, B.W.	2022	Pharm. Res.	39	8	1803-1815	10.1007/s11095-022-03257-3	Barrett 2022	Non-Gastro/Hepatology Focus
Survival after active surveillance versus upfront surgery for incidental small pancreatic neuroendocrine tumours	Ricci, C.; Partelli, S.; Landoni, L.; Rinzivillo, M.; Ingaldi, C.; Andreasi, V.; Savegnago, G.; Muffatti, F.; Fontana, M.; Tamburrino, D.; Deiro, G.; Alberici, L.; Campana, D.; Panzuto, F.; Tuveri, M.; Bassi, C.; Salvia, R.; Falconi, M.; Casadei, R.	2022	Br J Surg	109	8	733-738	10.1093/bjs/znac106	Ricci 2022	Non-NLP Focus
Influence of the Initial Neutrophils to Lymphocytes and Platelets Ratio on the Incidence and Severity of	Xiao, W.; Lu, Z.; Liu, Y.; Hua, T.; Zhang, J.; Hu, J.; Li, H.; Xu, Y.; Yang, M.	2022	Front. Immunol.	13			10.3389/fimmu.2022.925494	Xiao 2022	Non-Gastro/Hepatology Focus

Sepsis-Associated Acute Kidney Injury: A Double Robust Estimation Based on a Large Public Database									
Breast Density and Breast Cancer Risk	Harvey, J.A.	2022	J. Breast Imaging	4	4	339-341	10.1093/jbi/wbac040	Harvey 2022	Non-Gastro/Hepatology Focus
Thematic Analysis of Reddit Content About Buprenorphine-naloxone Using Manual Annotation and Natural Language Processing Techniques	Graves, R.L.; Perrone, J.; Al-Garadi, M.A.; Yang, Y.-C.; Love, J.; O'Connor, K.; Gonzalez-Hernandez, G.; Sarker, A.	2022	J. Addict. Med.	16	4	454-460	10.1097/ADM.0000000000000940	Graves 2022	Non-Gastro/Hepatology Focus
Association between menorrhagia and risk of intrauterine deviceâ€“related uterine perforation and device expulsion: results from the Association of Uterine Perforation and Expulsion of Intrauterine Device study	Getahun, D.; Fassett, M.J.; Gatz, J.; Armstrong, M.A.; Peipert, J.F.; Raine-Bennett, T.; Reed, S.D.; Zhou, X.; Schoendorf, J.; Postlethwaite, D.; Shi, J.M.; Saltus, C.W.; Wang, J.; Xie, F.; Chiu, V.Y.; Merchant, M.; Alabaster, A.; Ichikawa, L.E.; Hunter, S.; Im, T.M.; Takhar, H.S.; Ritchey,	2022	Am. J. Obstet. Gynecol.	227	1	59.e1-59.e9	10.1016/j.ajog.2022.03.025	Getahun 2022	Non-Gastro/Hepatology Focus

	M.E.; Chillemi, G.; Pisa, F.; Asiimwe, A.; Anthony, M.S.								
Type-2 Diabetes Mellitus and Risk of Colorectal Polyps: A Colonoscopy-Based Study Using Natural Language Processing	Hardikar, S.; Krick, B.; Benson, R.; Winn, M.; Winterton, C.; Newcomb, P.A.; Inadomi, J.M.; Ulrich, C.M.	2022	Cancer Epidemiol Biomarkers Prev	31	7	1513	10.1158/1055-9965.EPI-22-0481	Hardikar 2022	NLP used only as a study adjunct/enabler
The Advances in Computer Vision That Are Enabling More Autonomous Actions in Surgery: A Systematic Review of the Literature	Gumbs, A.A.; Grasso, V.; Bourdel, N.; Croner, R.; Spolverato, G.; Frigerio, I.; Illanes, A.; Hilal, M.A.; Park, A.; Elyan, E.	2022	Sensors	22	13		10.3390/s22134918	Gumbs 2022	Non-NLP Focus
Professionalism, Legitimacy and Collegiality	Abu-Elmagd, K.; Mazariegos, G.	2022	J. Pediatr. Surg.	57	7	1448-1449	10.1016/j.jpedsurg.2022.01.016	Abu-Elmagd 2022	Non-Gastro/Hepatology Focus
Artificial intelligence in the diagnosis of cirrhosis and portal hypertension	Li, X.; Kang, N.; Qi, X.; Huang, Y.	2022	J. Med. Ultrason.	49	3	371-379	10.1007/s10396-021-01153-8	Li 2022	Non-NLP Focus
Rigorous mathematical optimization of synthetic hepatic vascular trees	Jessen, E.; Steinbach, M.C.; Debbaut, C.; Schillinger, D.	2022	J. R. Soc. Interface	19	191		10.1098/rsif.2022.0087	Jessen 2022	Non-Gastro/Hepatology Focus
Improving Keyword-Based Topic Classification in Cancer Patient Forums with	Buonocore, T.M.; Parimbelli, E.; Sacchi, L.; Bellazzi, R.; Del	2022	Stud. Health Technol. Informatics	290		597-601	10.3233/SHTI220147	Buonocore 2022	Non-Gastro/Hepatology Focus

Multilingual Transformers	Campo, L.; Quaglini, S.								
A Disease Identification Algorithm for Medical Crowdfunding Campaigns: Validation Study	Doerstling, S.S.; Akrobetu, D.; Engelhard, M.M.; Chen, F.; Ubel, P.A.	2022	J. Med. Internet Res.	24	6		10.2196/32867	Doerstling 2022	Non-Gastro/Hepatology Focus
MVI-Mind: A Novel Deep-Learning Strategy Using Computed Tomography (CT)-Based Radiomics for End-to-End High Efficiency Prediction of Microvascular Invasion in Hepatocellular Carcinoma	Wang, L.; Wu, M.; Li, R.; Xu, X.; Zhu, C.; Feng, X.	2022	Cancers	14	12		10.3390/cancers14122956	Wang 2022	Non-NLP Focus
Implementation of specialised attention mechanisms: ICD-10 classification of Gastrointestinal discharge summaries in English, Spanish and Swedish	Blanco, A.; Remmer, S.; PÃ©rez, A.; Dalianis, H.; Casillas, A.	2022	J. Biomed. Informatics	130			10.1016/j.jbi.2022.104050	Blanco 2022	Non-Gastro/Hepatology Focus
Identification of antimicrobial peptides from the human gut microbiome using deep learning		2022	Nat. Biotechnol.	40	6	838-839	10.1038/s41587-022-01230-4		Non-NLP Focus
Discovering Key Topics in Emergency Medical Dispatch from Free Text	Ferri, P.; SÃ¡ez, C.; FÃ©lix-De Castro, A.; SÃ¡nchez-Cuesta, P.;	2022	Stud. Health Technol. Informatics	294		859-863	10.3233/SHTI220607	Ferri 2022	Non-Gastro/Hepatology Focus

Dispatcher Observations	GarcÃa-GÃ³mez, J.M.								
Regular feedback to individual endoscopists is associated with improved adenoma detection rate and other key performance indicators for colonoscopy	Lim, S.; Tritto, G.; Zeki, S.; Demartino, S.	2022	Frontline Gastroenterol.	13	6	509-516	10.1136/flgastro-2022-102091	Lim 2022	NLP used only as a study adjunct/enabler
BioChemDDI: Predicting Drugâ€“Drug Interactions by Fusing Biochemical and Structural Information through a Self-Attention Mechanism	Ren, Z.-H.; Yu, C.-Q.; Li, L.-P.; You, Z.-H.; Pan, J.; Guan, Y.-J.; Guo, L.-X.	2022	Biology	11	5		10.3390/biology11050758	Ren 2022	Non-Gastro/Hepatology Focus
Risk for Shoulder Conditions After Vaccination: A Population-Based Study Using Real-World Data	Zheng, C.; Duffy, J.; Liu, I.-L.A.; Sy, L.S.; Chen, W.; Qian, L.; Navarro, R.A.; Ryan, D.S.; Kim, S.S.; Mercado, C.; Jacobsen, S.J.	2022	Ann. Intern. Med.	175	5	634-643	10.7326/M21-3023	Zheng 2022	Non-Gastro/Hepatology Focus
Labeling Noncontrast Head CT Reports for Common Findings Using Natural Language Processing	Iorga, M.; Drakopoulos, M.; Naidech, A.M.; Katsaggelos, A.K.; Parrish, T.B.; Hill, V.B.	2022	Am. J. Neuroradiol.	43	5	721-726	10.3174/ajnr.A7500	Iorga 2022	Non-Gastro/Hepatology Focus

Automatic Dental Plaque Segmentation Based on Local-to-Global Features Fused Self-Attention Network	Li, S.; Guo, Y.; Pang, Z.; Song, W.; Hao, A.; Xia, B.; Qin, H.	2022	IEEE J. Biomedical Health Informat.	26	5	2240-2251	10.1109/JBHI.2022.3141773	Li 2022	Non-Gastro/Hepatology Focus
Using the Electronic Health Record to Characterize the Hepatitis C Virus Care Cascade	Christy, S.M.; Reich, R.R.; Rathwell, J.A.; Vadaparampil, S.T.; Isaacs-Soriano, K.A.; Friedman, M.S.; Roetzheim, R.G.; Giuliano, A.R.	2022	Public Health Rep.	137	3	498-505	10.1177/00333549211005812	Christy 2022	Non-NLP Focus
The Research Landscape of Multiple Endocrine Neoplasia Type 1 (2000â€"2021): A Bibliometric Analysis	Feng, C.; Chen, H.; Huang, L.; Feng, Y.; Chang, S.	2022	Front. Med.	9			10.3389/fmed.2022.832662	Feng 2022	Non-Gastro/Hepatology Focus
MDGNN: Microbial Drug Prediction Based on Heterogeneous Multi-Attention Graph Neural Network	Pi, J.; Jiao, P.; Zhang, Y.; Li, J.	2022	Front. Microbiol.	13			10.3389/fmicb.2022.819046	Pi 2022	Non-Gastro/Hepatology Focus
Continuous ADR50 monitoring through automated linkage between endoscopy and pathology: a quality	Rasschaert, G.; Gomez Galdon, M.; Vandevelde, J.; Eisendrath, P.	2022	Acta Gastro-Enterol. Belg.	85	2	259-266	10.51821/85.2.9706	Rasschaert 2022	Non-NLP Focus

improvement initiative in a Brussels public hospital									
The Experiences of Patients with Adjuvant and Metastatic Melanoma Using Disease-Specific Social Media Communities in the Advent of Novel Therapies (Excite Project): Social Media Listening Study	Faust, G.; Booth, A.; Merinopoulou, E.; Halhol, S.; Tosar, H.; Nawaz, A.; Szlachetka, M.; Chiu, G.	2022	JMIR Cancer	8	2		10.2196/34073	Faust 2022	Non-Gastro/Hepatology Focus
Artificial Intelligence for Disease Assessment in Inflammatory Bowel Disease: How Will it Change Our Practice?	Stidham, R.W.; Takenaka, K.	2022	Gastroenterology	162	5	1493-1506	10.1053/j.gastro.2021.12.238	Stidham 2022	Review/Perspective Article Only
LTM-TCM: A comprehensive database for the linking of Traditional Chinese Medicine with modern medicine at molecular and phenotypic levels	Li, X.; Ren, J.; Zhang, W.; Zhang, Z.; Yu, J.; Wu, J.; Sun, H.; Zhou, S.; Yan, K.; Yan, X.; Wang, W.	2022	Pharmacol. Res.	178			10.1016/j.phrs.2022.106185	Li 2022	Non-Gastro/Hepatology Focus
Hand grip force estimation via EMG imaging	Fialkoff, B.; Hadad, H.; Santos, D.; Simini, F.; David, M.	2022	Biomed. Signal Process. Control	74			10.1016/j.bspc.2022.103550	Fialkoff 2022	Non-Gastro/Hepatology Focus

Assessing adverse event reports of hysteroscopic sterilization device removal using natural language processing	Mao, J.; Sedrakyan, A.; Sun, T.; Guiahi, M.; Chudnoff, S.; Kinard, M.; Johnson, S.B.	2022	Pharmacoepidemiol. Drug Saf.	31	4	442-451	10.1002/pds.5402	Mao 2022	Non-Gastro/Hepatology Focus
Machine-Learning-Based Bibliometric Analysis of Pancreatic Cancer Research Over the Past 25 Years	Wang, K.; Herr, I.	2022	Front. Oncol.	12			10.3389/fonc.2022.832385	Wang 2022	NLP used only as a study adjunct/enabler
Reconstruction and Exploratory Analysis of mTORC1 Signaling Pathway and Its Applications to Various Diseases Using Network-Based Approach	Buddham, R.; Chauhan, S.; Narad, P.; Mathur, P.	2022	J. Microbiol. Biotechnol.	32	3	365-377	10.4014/jmb.2108.08007	Buddham 2022	NLP used only as a study adjunct/enabler
Artificial intelligence-assisted colonoscopy: a narrative review of current data and clinical applications	Li, J.W.; Mun Wang, L.; Leong Ang, T.	2022	Singapore Med. J.	63	3	118-124	10.11622/smedj.2022044	Li 2022	Review/Perspective Article Only
S2DV: Converting SMILES to a drug vector for predicting the activity of anti-HBV small molecules	Shao, J.; Gong, Q.; Yin, Z.; Pan, W.; Pandiyan, S.; Wang, L.	2022	Brief. Bioinform.	23	2		10.1093/bib/bbab593	Shao 2022	NLP used only as a study adjunct/enabler
Using Natural Language Processing	Mashima, Y.; Tamura, T.; Kunikata, J.;	2022	Cancer Informatics	21			10.1177/11769351221085064	Mashima 2022	Non-Gastro/Hepatology Focus

Techniques to Detect Adverse Events From Progress Notes Due to Chemotherapy	Tada, S.; Yamada, A.; Tanigawa, M.; Hayakawa, A.; Tanabe, H.; Yokoi, H.								
Digital haemophilia: Insights into the use of social media for haemophilia care, research and advocacy	Chen, R.; Muralidharan, K.; Samelson-Jones, B.J.	2022	Haemophilia	28	2	247-253	10.1111/hae.14510	Chen 2022	Non-Gastro/Hepatology Focus
Increased Oxygen Desaturation Time During Sleep Is a Risk Factor for NASH in Patients With Obstructive Sleep Apnea: A Prospective Cohort Study	Landete, P.; FernÃ¡ndez-GarcÃa, C.E.; Aldave-Orzaiz, B.; HernÃ¡ndez-Olivo, M.; Acosta-GutiÃ©rrez, C.M.; Zamora-GarcÃa, E.; Ancochea, J.; GonzÃ¡lez-RodrÃguez, Ã.; GarcÃa-MonzÃ³n, C.	2022	Front. Med.	9			10.3389/fmed.2022.808417	Landete 2022	Non-NLP Focus
Kimchi improves irritable bowel syndrome: results of a randomized, double-blind placebo-controlled study	Kim, H.-Y.; Park, E.-S.; Choi, Y.S.; Park, S.J.; Kim, J.H.; Chang, H.K.; Park, K.-Y.	2022	Food Nutri. Res.	66			10.29219/fnr.v66.8268	Kim 2022	Non-NLP Focus
Why Do Small Intraductal Papillary Mucinous Neoplasms Create Such a Huge	Khalaf, N.; Abrams, H.R.; Eke, C.; Kanwal, F.; El-Serag, H.B.	2022	Pancreas	51	2	E13-E15	10.1097/MPA.0000000000001989	Khalaf 2022	Review/Perspective Article Only

Management Challenge?									
Key use cases for artificial intelligence to reduce the frequency of adverse drug events: a scoping review	Syrowatka, A.; Song, W.; Amato, M.G.; Foer, D.; Edrees, H.; Co, Z.; Kuznetsova, M.; Dulgarian, S.; Seger, D.L.; Simona, A.; Bain, P.A.; Purcell Jackson, G.; Rhee, K.; Bates, D.W.	2022	Lancet Digit. Heal.	4	2	e137-e148	10.1016/S2589-7500(21)00229-6	Syrowatka 2022	Non-Gastro/Hepatology Focus
Mapping multimorbidity in individuals with schizophrenia and bipolar disorders: Evidence from the South London and Maudsley NHS Foundation Trust Biomedical Research Centre (SLAM BRC) case register	Bendayan, R.; Kraljevic, Z.; Shaari, S.; Das-Munshi, J.; Leipold, L.; Chaturvedi, J.; Mirza, L.; Aldelemi, S.; Searle, T.; Chance, N.; Mascio, A.; Skiada, N.; Wang, T.; Roberts, A.; Stewart, R.; Bean, D.; Dobson, R.	2022	BMJ Open	12	1		10.1136/bmjopen-2021-054414	Bendayan 2022	Non-Gastro/Hepatology Focus
Application of Real-World Data to External Control Groups in Oncology Clinical Trial Drug Development	Yap, T.A.; Jacobs, I.; Baumfeld Andre, E.; Lee, L.J.; Beaupre, D.; Azoulay, L.	2022	Front. Oncol.	11			10.3389/fonc.2021.695936	Yap 2022	Non-Gastro/Hepatology Focus

The Sensitivity of Annotator Bias to Task Definitions in Argument Mining	Jakobsen, T.S.T.; Barrett, M.; SÃ¸gaard, A.; Lassen, D.D.	2022	Proc. Linguist. Annot. Workshop, LAW - held conjunction Lang. Resour. Eval. Conf., LREC Workshop			44-61		Jakobsen 2022	Non-Gastro/Hepatology Focus
17th International Symposium on Visual Computing, ISVC 2022		2022	Lect. Notes Comput. Sci.	13598 LNCS					Non-Gastro/Hepatology Focus
Patient journey of individuals tested for HCV in Spain: LiverTAI, a retrospective analysis of EHRs through natural language processing	Calleja Panero, J.L.; de la Poza, G.; Hidalgo, L.; Aguilera Sancho-Tello, M.V.; Torras, X.; Santos de Lamadrid, R.; MatÃ©, C.; SÃ¡nchez AntolÃn, G.	2022	Gastroenterol. Hepatol.				10.1016/j.gastrohep.2022.10.012	CallejaPanero 2022	NLP used only as a study adjunct/enabler
Machine Learning in Healthcare: Current Trends and the Future	Usmani, U.A.; Jaafar, J.	2022	Lect. Notes Electr. Eng.	758		659-675	10.1007/978-981-16-2183-3_64	Usmani 2022	Review/Perspective Article Only
Keyword-augmented and semi-automatic generation of FESS reports: a proof-of-concept study	Kunz, V.; Wildfeuer, V.; Bieck, R.; Sorge, M.; Zebralla, V.; Dietz, A.; Neumuth, T.; Pirlich, M.	2022	Int. J. Comput. Assisted Radiol. Surg.				10.1007/s11548-022-02791-0	Kunz 2022	Non-Gastro/Hepatology Focus
Effect of Telmisartan and Quercetin in 5 Fluorouracil-Induced Renal Toxicity in Rats	Ali, H.H.; Ahmed, Z.A.; Aziz, T.A.	2022	J. Inflamm. Res.	15		6113-6124	10.2147/JIR.S389017	Ali 2022	Non-Gastro/Hepatology Focus
Registered Trials of Artificial Intelligence Conducted on	Zheng, G.; Shi, L.; Liu, J.; Zhao, Y.; Du, F.; He, Y.; Yang, X.; Song,	2022	Dis. Markers	2022			10.1155/2022/6847073	Zheng 2022	Non-NLP Focus

Chronic Liver Disease: A Cross-Sectional Study on ClinicalTrials.gov	N.; Wen, J.; Gao, H.								
Index diagnoses of gastric intestinal metaplasia in the United States: patient characteristics, endoscopic findings, and clinical practice patterns at a large tertiary care center	Parbhu, S.K.; Shah, S.C.; Sossenheimer, M.J.; Fang, J.C.; Peterson, K.A.; Gawron, A.J.	2022	Ther. Adv. Gastroenterol.	15			10.1177/17562848221117640	Parbhu 2022	NLP used only as a study adjunct/enabler
The Copenhagen Corpus of Eye Tracking Recordings from Natural Reading of Danish Texts	Hollenstein, N.; Barrett, M.; BjÃ¶rnsdÃ³ttir, M.	2022	Lang. Resour. Eval. Conf., LREC			1712-1720		Hollenstein 2022	Non-Gastro/Hepatology Focus
Fast and precise prediction of non-coding RNAs (ncRNAs) using sequence alignment and k-mer counting	Jha, M.; Gupta, R.; Saxena, R.	2022	Int. J. Inf. Technol.				10.1007/s41870-022-01064-y	Jha 2022	Non-Gastro/Hepatology Focus
Machine Learning Approaches in Inflammatory Bowel Disease	Scarpino, I.; Vallelunga, R.; Luzza, F.; Cannataro, M.	2022	Lect. Notes Comput. Sci.	13351 LNCS		539-545	10.1007/978-3-031-08754-7_59	Scarpino 2022	Review/Perspective Article Only
Classification of Liver Tumors from Computed Tomography Using NRSVM	Priyadarsini, S.; Romero, C.A.T.; Mrunalini, M.; Rao, G.R.K.; Sengan, S.	2022	Intell. Autom. Soft Comp.	33	3	1517-1530	10.32604/iasc.2022.024786	Priyadarsini 2022	Non-NLP Focus

Clinical Research on Gastrointestinal Surgery Based on Smart Medicine	Lu, Y.	2022	J. Healthc. Eng.	2022			10.1155/2022/3698721	Lu 2022	Review/Perspective Article Only
Natural Language Processing for Virtual Reference Analysis	Sharma, A.; Barrett, K.; Stapelfeldt, K.	2022	Evid. Based Libr. Inf. Pract.	17	1	78-93	10.18438/eblip30014	Sharma 2022	Non-Gastro/Hepatology Focus
Natural Language Processing of Large-Scale Structured Radiology Reports to Identify Oncologic Patients with or Without Splenomegaly over a 10-Year Period	Sun, S.; Lupton, K.; Batch, K.; Nguyen, H.; Gazit, L.; Gangai, N.; Cho, J.; Nicholas, K.; Zulkernine, F.; Sevilimedu, V.; Simpson, A.; Do, R.K.G.	2022	JCO Clin. Cancer Inform.	6			10.1200/CCI.21.00104	Sun 2022	NLP used only as a study adjunct/enabler
An attentive joint model with transformer-based weighted graph convolutional network for extracting adverse drug event relation	El-allaly, E.-D.; Sarrouti, M.; En-Nahnahi, N.; Ouatik El Alaoui, S.	2022	J. Biomed. Informatics	125			10.1016/j.jbi.2021.103968	El-allaly 2022	Non-Gastro/Hepatology Focus
PASCLex: A comprehensive post-acute sequelae of COVID-19 (PASC) symptom lexicon derived from electronic health record clinical notes	Wang, L.; Foer, D.; MacPhaul, E.; Lo, Y.-C.; Bates, D.W.; Zhou, L.	2022	J. Biomed. Informatics	125			10.1016/j.jbi.2021.103951	Wang 2022	Non-Gastro/Hepatology Focus

Emerging technologies and their impact on regulatory science	Anklam, E.; Bahl, M.I.; Ball, R.; Beger, R.D.; Cohen, J.; Fitzpatrick, S.; Girard, P.; Halamoda-Kenzaoui, B.; Hinton, D.; Hirose, A.; Hoeveler, A.; Honma, M.; Hugas, M.; Ishida, S.; Kass, G.E.N.; Kojima, H.; Krefting, I.; Liachenko, S.; Liu, Y.; Masters, S.; Marx, U.; McCarthy, T.; Mercer, T.; Patri, A.; Pelaez, C.; Pirmohamed, M.; Platz, S.; Ribeiro, A.J.S.; Rodricks, J.V.; Rusyn, I.; Salek, R.M.; Schoonjans, R.; Silva, P.; Svendsen, C.N.; Sumner, S.; Sung, K.; Tagle, D.; Tong, L.; Tong, W.; Eijnden-van-Raaij, J.V.D.; Vary, N.; Wang, T.; Waterton, J.;	2022	Exp. Biol. Med.	247	1	Jan-75	10.1177/15353702211052280	Anklam 2022	Non-Gastro/Hepatology Focus

	Wang, M.; Wen, H.; Wishart, D.; Yuan, Y.; Slikker Jr, W.								
Utilizing GCN and Meta-Learning Strategy in Unsupervised Domain Adaptation for Pancreatic Cancer Segmentation	Li, J.; Feng, C.; Lin, X.; Qian, X.	2022	IEEE J. Biomedical Health Informat.	26	1	79-89	10.1109/JBHI.2021.3085092	Li 2022	Non-NLP Focus
Human Genetics to Identify Therapeutic Targets for NAFLD: Challenges and Opportunities	Du, X.; DeForest, N.; Majithia, A.R.	2021	Front. Endocrinol.	12			10.3389/fendo.2021.777075	Du 2021	Review/Perspective Article Only
User Behaviors and User-Generated Content in Chinese Online Health Communities: Comparative Study	Lei, Y.; Xu, S.; Zhou, L.	2021	J. Med. Internet Res.	23	12		10.2196/19183	Lei 2021	Non-Gastro/Hepatology Focus
Utility of blood cultures and empiric antibiotics in febrile pediatric hemophilia patients with central venous access devices	Al-Samkari, H.; Ozonoff, A.; Landschaft, A.; Kimia, R.; Harper, M.B.; Croteau, S.E.; Kimia, A.A.	2021	Pediatr. Emerg. Care	37	12	E1531-E1534	10.1097/PEC.0000000000002106	Al-Samkari 2021	Non-Gastro/Hepatology Focus
Clinical knowledge extraction via sparse embedding regression (KESER) with multi-center large scale	Hong, C.; Rush, E.; Liu, M.; Zhou, D.; Sun, J.; Sonabend, A.; Castro, V.M.; Schubert, P.; Panickan, V.A.;	2021	npj Digit. Med.	4	1		10.1038/s41746-021-00519-z	Hong 2021	Non-Gastro/Hepatology Focus

electronic health record data	Cai, T.; Costa, L.; He, Z.; Link, N.; Hauser, R.; Gaziano, J.M.; Murphy, S.N.; Ostrouchov, G.; Ho, Y.-L.; Begoli, E.; Lu, J.; Cho, K.; Liao, K.P.; VA Million Veteran Program								
Characterizing shared and distinct symptom clusters in common chronic conditions through natural language processing of nursing notes	Koleck, T.A.; Topaz, M.; Tatonetti, N.P.; George, M.; Miaskowski, C.; Smaldone, A.; Bakken, S.	2021	Res. Nurs. Health	44	6	906-919	10.1002/nur.22190	Koleck 2021	Non-Gastro/Hepatology Focus
Method used to identify adenomyosis and potentially undiagnosed adenomyosis in a large, U.S. electronic health record database	Loughlin, A.M.; Chiuve, S.E.; Reznor, G.; Doherty, M.; Missmer, S.A.; Chomistek, A.K.; Enger, C.	2021	Pharmacoepidemiol. Drug Saf.	30	12	1675-1686	10.1002/pds.5333	Loughlin 2021	Non-Gastro/Hepatology Focus
Development and validation of an automated emergency department-based syndromic surveillance system to enhance public health surveillance in Yukon: a lower-	Bouchouar, E.; Hetman, B.M.; Hanley, B.	2021	BMC Public Health	21	1		10.1186/s12889-021-11132-w	Bouchouar 2021	Non-Gastro/Hepatology Focus

resourced and remote setting									
Nlp promotes autophagy through facilitating the interaction of Rab7 and FYCO1	Xiao, W.; Yeerken, D.; Li, J.; Li, Z.; Jiang, L.; Li, D.; Fu, M.; Ma, L.; Song, Y.; Zhang, W.; Zhan, Q.	2021	Signal Transduct. Target. Ther.	6	1		10.1038/s41392-021-00543-1	Xiao 2021	Non-Gastro/Hepatology Focus
Semantic categorization of Chinese eligibility criteria in clinical trials using machine learning methods	Zong, H.; Yang, J.; Zhang, Z.; Li, Z.; Zhang, X.	2021	BMC Med. Informatics Decis. Mak.	21	1		10.1186/s12911-021-01487-w	Zong 2021	Non-Gastro/Hepatology Focus
Trends in reasons for emergency calls during the COVID-19 crisis in the department of Gironde, France using artificial neural network for natural language classification	Gil-JardinÃ©, C.; Chenais, G.; Pradeau, C.; Tentillier, E.; Revel, P.; Combes, X.; Galinski, M.; Tellier, E.; Lagarde, E.	2021	Scand. J. Trauma Resusc. Emerg. Med.	29	1		10.1186/s13049-021-00862-w	Gil-JardinÃ© 2021	Non-Gastro/Hepatology Focus
Evaluation of an international medical E-learning course with natural language processing and machine learning	Borakati, A.	2021	BMC Med. Educ.	21	1		10.1186/s12909-021-02609-8	Borakati 2021	Non-Gastro/Hepatology Focus
Visual Loss Caused by Central Retinal Artery Occlusion After Bee Sting: A Case Report	Su, Z.; Hu, Z.; Wang, L.; Wang, Y.; Fang, X.; Ye, P.	2021	Front. Med.	8			10.3389/fmed.2021.707978	Su 2021	Non-Gastro/Hepatology Focus

Artificial intelligence language predictors of two-year trauma-related outcomes	Oltmanns, J.R.; Schwartz, H.A.; Ruggero, C.; Son, Y.; Miao, J.; Waszczuk, M.; Clouston, S.A.P.; Bromet, E.J.; Luft, B.J.; Kotov, R.	2021	J. Psychiatr. Res.	143		239-245	10.1016/j.jpsychires.2021.09.015	Oltmanns 2021	Non-Gastro/Hepatology Focus
Using artificial intelligence with natural language processing to combine electronic health record's structured and free text data to identify nonvalvular atrial fibrillation to decrease strokes and death: Evaluation and case-control study	Elkin, P.L.; Mullin, S.; Mardekian, J.; Crowner, C.; Sakilay, S.; Sinha, S.; Brady, G.; Wright, M.; Nolen, K.; Trainer, J.; Koppel, R.; Schlegel, D.; Kaushik, S.; Zhao, J.; Song, B.; Anand, E.	2021	J. Med. Internet Res.	23	11		10.2196/e28946	Elkin 2021	Non-Gastro/Hepatology Focus
Tracing multisensory food experiences on Twitter	KÄ[illegible]le, M.; Å Ä·ilters, J.; Rikters, M.	2021	Int. J. Food Des.	6	2	181-212	10.1386/IJFD_00030_1	KÄ[illegible]le 2021	Non-Gastro/Hepatology Focus
COVID-19 and Risk of VTE in Ethnically Diverse Populations	Go, A.S.; Reynolds, K.; Tabada, G.H.; Prasad, P.A.; Sung, S.H.; Garcia, E.; Portugal, C.; Fan, D.; Pai, A.P.; Fang, M.C.	2021	Chest	160	4	1459-1470	10.1016/j.chest.2021.07.025	Go 2021	Non-Gastro/Hepatology Focus

Patterns of metastatic disease in patients with cancer derived from natural language processing of structured CT radiology reports over a 10-year period	Do, R.K.G.; Lupton, K.; Causa Andrieu, P.I.; Luthra, A.; Taya, M.; Batch, K.; Nguyen, H.; Rahurkar, P.; Gazit, L.; Nicholas, K.; Fong, C.J.; Gangai, N.; Schultz, N.; Zulkernine, F.; Sevilimedu, V.; Juluru, K.; Simpson, A.; Hricak, H.	2021	Radiology	301	1	115-122	10.1148/radiol.2021210043	Do 2021	Non-Gastro/Hepatology Focus
Focus U-Net: A novel dual attention-gated CNN for polyp segmentation during colonoscopy	Yeung, M.; Sala, E.; SchÃ¶nlieb, C.-B.; Rundo, L.	2021	Comput. Biol. Med.	137			10.1016/j.compbiomed.2021.104815	Yeung 2021	Non-NLP Focus
Applying interpretable deep learning models to identify chronic cough patients using EHR data	Luo, X.; Gandhi, P.; Zhang, Z.; Shao, W.; Han, Z.; Chandrasekaran, V.; Turzhitsky, V.; Bali, V.; Roberts, A.R.; Metzger, M.; Baker, J.; La Rosa, C.; Weaver, J.; Dexter, P.; Huang, K.	2021	Comput. Methods Programs Biomed.	210			10.1016/j.cmpb.2021.106395	Luo 2021	Non-Gastro/Hepatology Focus

Artificial intelligence or colonoscopy quality the likes of which have never been seen	Marlicz, W.; Koulaouzidis, A.; Koulaouzidis, G.	2021	Gastrointest. Endosc.	94	4	872-873	10.1016/j.gie.2021.05.007	Marlicz 2021	Review/Perspective Article Only
A risk-stratified approach to venous thromboembolism prophylaxis with aspirin or warfarin following total hip and knee arthroplasty: A cohort study	Johnson, S.A.; Jones, A.E.; Young, E.; Jennings, C.; Simon, K.; Fleming, R.P.; Witt, D.M.	2021	Thromb. Res.	206		120-127	10.1016/j.thromres.2021.08.009	Johnson 2021	Non-Gastro/Hepatology Focus
Adoption of New Technologies: Artificial Intelligence	Glissen Brown, J.R.; Berzin, T.M.	2021	Gastrointest. Endosc. Clin. North Am.	31	4	743-758	10.1016/j.giec.2021.05.010	GlissenBrown 2021	Review/Perspective Article Only
Variation in Radiologistsâ€™ Follow-Up Imaging Recommendations for Small Cystic Pancreatic Lesions	Kapoor, N.; Lacson, R.; Eskian, M.; Cochon, L.; Glazer, D.; Ip, I.; Khorasani, R.	2021	J. Am. Coll. Radiol.	18	10	1405-1414	10.1016/j.jacr.2021.06.007	Kapoor 2021	NLP used only as a study adjunct/enabler
Cerebral Venous Sinus Thrombosis is not Significantly Linked to COVID-19 Vaccines or Non-COVID Vaccines in a Large Multi-State Health System	Pawlowski, C.; RincÃ³n-Hekking, J.; Awasthi, S.; Pandey, V.; Lenehan, P.; Venkatakrishnan, A.J.; Bade, S.; O'Horo, J.C.; Virk, A.; Swift, M.D.; Williams, A.W.; Gores, G.J.; Badley, A.D.; Halamka,	2021	J. Stroke Cerebrovasc. Dis.	30	10		10.1016/j.jstrokecerebrovasdis.2021.105923	Pawlowski 2021	Non-Gastro/Hepatology Focus

	J.; Soundararajan, V.								
A Twitter discourse analysis of negative feelings and stigma related to NAFLD, NASH and obesity	Lazarus, J.V.; Kakalou, C.; Palayew, A.; Karamanidou, C.; Maramis, C.; Natsiavas, P.; Picchio, C.A.; Villota-Rivas, M.; Zelber-Sagi, S.; Carrieri, P.	2021	Liver Int.	41	10	2295-2307	10.1111/liv.14969	Lazarus 2021	Risk Factors For Disease Only
ASO Author Reflections: Developing a Clinician-Friendly Resource to Promote Awareness of Non-Medullary Thyroid Cancer Susceptibility Genes and Their Associated Diseases	Yin, K.; Zhou, J.; Singh, P.; Hughes, K.S.	2021	Ann. Surg. Oncol.	28	11	6601-6602	10.1245/s10434-021-09783-5	Yin 2021	Non-Gastro/Hepatology Focus
Non-medullary Thyroid Cancer Susceptibility Genes: Evidence and Disease Spectrum	Zhou, J.; Singh, P.; Yin, K.; Wang, J.; Bao, Y.; Wu, M.; Pathak, K.; McKinley, S.K.; Braun, D.; Lubitz, C.C.; Hughes, K.S.	2021	Ann. Surg. Oncol.	28	11	6590-6600	10.1245/s10434-021-09745-x	Zhou 2021	Non-Gastro/Hepatology Focus
Development of machine learning and natural language processing	Karhade, A.V.; Bongers, M.E.R.; Groot, O.Q.; Cha, T.D.; Doorly, T.P.;	2021	Spine J.	21	10	1635-1642	10.1016/j.spinee.2020.04.001	Karhade 2021	Non-Gastro/Hepatology Focus

algorithms for preoperative prediction and automated identification of intraoperative vascular injury in anterior lumbar spine surgery	Fogel, H.A.; Hershman, S.H.; Tobert, D.G.; Srivastava, S.D.; Bono, C.M.; Kang, J.D.; Harris, M.B.; Schwab, J.H.								
A Bibliometric Analysis of 8,276 Publications During the Past 25 Years on Cholangiocarcinoma by Machine Learning	Zhang, Z.; Wang, Z.; Huang, Y.	2021	Front. Oncol.	11			10.3389/fonc.2021.687904	Zhang 2021	Review/Perspective Article Only
Learning, visualizing and exploring 16S rRNA structure using an attention-based deep neural network	Zhao, Z.; Woloszynek, S.; Agbavor, F.; Mell, J.C.; Sokhansanj, B.A.; Rosen, G.L.	2021	PLoS Comput. Biol.	17	9		10.1371/journal.pcbi.1009345	Zhao 2021	Non-Gastro/Hepatology Focus
Deep Neural Network Visualization Based on Interpretable Basis Decomposition and Knowledge Graph	Ruan, L.; Wen, S.-S.; Niu, Y.-M.; Li, S.-N.; Xue, Y.-Z.; Ruan, T.; Xiao, L.-M.	2021	Jisuanji Xuebao	44	9	1786-1805	10.11897/SP.J.1016.2021.01786	Ruan 2021	Non-Gastro/Hepatology Focus
Evaluation of a prospective adverse event reporting system in interventional radiology	Mulvihill, S.B.; Healy, G.M.; O'Rourke, C.; Cantwell, C.P.	2021	Clin. Radiol.	76	9	659-664	10.1016/j.crad.2021.04.009	Mulvihill 2021	Non-Gastro/Hepatology Focus

The Design and Optimization of Extractive Distillation for Separating the Acetone/ n-Heptane Binary Azeotrope Mixture	Kianinia, M.; Abdoli, S.M.	2021	ACS Omega	6	34	22447-22453	10.1021/acsomega.1c03513	Kianinia 2021	Non-Gastro/Hepatology Focus
Artificial intelligence surgery: How do we get to autonomous actions in surgery?	Gumbs, A.A.; Frigerio, I.; Spolverato, G.; Croner, R.; Illanes, A.; Chouillard, E.; Elyan, E.	2021	Sensors	21	16		10.3390/s21165526	Gumbs 2021	Review/Perspective Article Only
A framework to extract biomedical knowledge from gluten-related tweets: The case of dietary concerns in digital era	PÃ©rez-PÃ©rez, M.; Igrejas, G.; Fdez-Riverola, F.; LourenÃ§o, A.	2021	Artif. Intell. Med.	118			10.1016/j.artmed.2021.102131	PÃ©rez-PÃ©rez 2021	Risk Factors For Disease Only
Enhancing evidence-based medicine with natural language argumentative analysis of clinical trials	Mayer, T.; Marro, S.; Cabrio, E.; Villata, S.	2021	Artif. Intell. Med.	118			10.1016/j.artmed.2021.102098	Mayer 2021	Non-Gastro/Hepatology Focus
Bidirectional Representation Learning from Transformers Using Multimodal Electronic Health Record Data to Predict Depression	Meng, Y.; Speier, W.; Ong, M.K.; Arnold, C.W.	2021	IEEE J. Biomedical Health Informat.	25	8	3121-3129	10.1109/JBHI.2021.3063721	Meng 2021	Non-Gastro/Hepatology Focus

Immune2vec: Embedding B/T Cell Receptor Sequences in â„� N Using Natural Language Processing	Ostrovsky-Berman, M.; Frankel, B.; Polak, P.; Yaari, G.	2021	Front. Immunol.	12			10.3389/fimmu.2021.680687	Ostrovsky-Berman 2021	Non-Gastro/Hepatology Focus
Finding a new balance between a genetics-first or phenotype-first approach to the study of disease	Kohane, I.S.	2021	Neuron	109	14	2216-2219	10.1016/j.neuron.2021.07.001	Kohane 2021	Non-Gastro/Hepatology Focus
Non-occlusive mesenteric ischemia: Diagnostic challenges and perspectives in the era of artificial intelligence	Bourcier, S.; Klug, J.; Nguyen, L.S.	2021	World J. Gastroenterol.	27	26	4088-4103	10.3748/wjg.v27.i26.4088	Bourcier 2021	Review/Perspective Article Only
Decoding EEG Brain Activity for Multi-Modal Natural Language Processing	Hollenstein, N.; Renggli, C.; Glaus, B.; Barrett, M.; Troendle, M.; Langer, N.; Zhang, C.	2021	Front. Human Neurosci.	15			10.3389/fnhum.2021.659410	Hollenstein 2021	Non-Gastro/Hepatology Focus
Information and communication technologies applied to intelligent buildings: A review	Parisi, F.; Fanti, M.P.; Mangini, A.M.	2021	J. Inf. Technol. Constr.	26		458-488	10.36680/j.itcon.2021.025	Parisi 2021	Non-Gastro/Hepatology Focus
Small Intestine Varices in Cirrhosis at a High-Volume Liver Transplant Center: A Retrospective	Jansson-Knodell, C.L.; Calderon, G.; Weber, R.; Ghabril, M.	2021	Am J Gastroenterol	116	7	1426-1436	10.14309/ajg.0000000000001204	Jansson-Knodell 2021	NLP used only as a study adjunct/enabler

Database Study and Literature Review									
Differences in Biologic Utilization and Surgery Rates in Pediatric and Adult Crohn's Disease: Results from a Large Electronic Medical Record-derived Cohort	Kurowski, J.A.; Milinovich, A.; Ji, X.; Bauman, J.; Sugano, D.; Kattan, M.W.; Achkar, J.-P.	2021	Inflammatory Bowel Dis.	27	7	1035-1044	10.1093/ibd/izaa239	Kurowski 2021	NLP used only as a study adjunct/enabler
Radiologically and clinically diagnosed acute pulmonary oedema in critically ill patients: Prevalence, patient characteristics, treatments and outcomes	El-Khawas, K.; Richmond, D.; Zwakman-Hessels, L.; Cutuli, S.L.; Belletti, A.; Naorungroj, T.; Abdelkarim, H.; Yang, N.; Bellomo, R.	2021	Crit. Care Resusc.	23	2	154-162	10.51893/2021.2.oa2	El-Khawas 2021	Non-Gastro/Hepatology Focus
Natural language processing to assess the epidemiology of delirium-suggestive behavioural disturbances in critically ill patients	Young, M.; Holmes, N.; Robbins, R.; Marhoon, N.; Amjad, S.; Neto, A.S.; Bellomo, R.	2021	Crit. Care Resusc.	23	2	144-153	10.51893/2021.2.oa1	Young 2021	Non-Gastro/Hepatology Focus
Rigorous NLP distillation models for simultaneous optimization to	Yeoh, K.P.; Hui, C.W.	2021	Clean. Eng. Technol.	2			10.1016/j.clet.2021.100066	Yeoh 2021	Non-Gastro/Hepatology Focus

reduce utility and capital costs									
Impact of deep learning-determined smoking status on mortality of cancer patients: never too late to quit	Karlsson, A.; Ellonen, A.; Irjala, H.; VÃ¤liaho, V.; Mattila, K.; Nissi, L.; KytÃ¶, E.; Kurki, S.; RistamÃ¤ki, R.; Vihinen, P.; Laitinen, T.; Ã…lgars, A.; JyrkkiÃ¶, S.; Minn, H.; HeervÃ¤, E.	2021	ESMO Open	6	3		10.1016/j.esmoop.2021.100175	Karlsson 2021	Non-Gastro/Hepatology Focus
Application of Artificial Intelligence for the Diagnosis and Treatment of Liver Diseases	Ahn, J.C.; Connell, A.; Simonetto, D.A.; Hughes, C.; Shah, V.H.	2021	Hepatology	73	6	2546-2563	10.1002/hep.31603	Ahn 2021	Review/Perspective Article Only
Prevalence of Medical Cannabis Use and Associated Health Conditions Documented in Electronic Health Records among Primary Care Patients in Washington State	Matson, T.E.; Carrell, D.S.; Bobb, J.F.; Cronkite, D.J.; Oliver, M.M.; Luce, C.; Ghitza, U.E.; Hsu, C.W.; Campbell, C.I.; Browne, K.C.; Binswanger, I.A.; Saxon, A.J.; Bradley, K.A.; Lapham, G.T.	2021	JAMA Netw. Open	4	5		10.1001/jamanetworkopen.2021.9375	Matson 2021	Non-Gastro/Hepatology Focus
Sociodemographic and economic factors in outcomes of tube shunts for	Shalaby, W.S.; Arbabi, A.; Myers, J.S.; Moster, M.R.; Razeghinejad,	2021	J. Curr. Glaucoma Pract.	15	2	70-77	10.5005/jp-journals-10078-1303	Shalaby 2021	Non-Gastro/Hepatology Focus

neovascular glaucoma	R.; Katz, L.J.; Shukla, A.G.								
Automated Identification of Patients With Immune-Related Adverse Events From Clinical Notes Using Word Embedding and Machine Learning	Gupta, S.; Belouali, A.; Shah, N.J.; Atkins, M.B.; Madhavan, S.	2021	JCO Clin Cancer Inform	5		541-549	10.1200/CCI.20.00109	Gupta 2021	Non-Gastro/Hepatology Focus
Using computable knowledge mined from the literature to elucidate confounders for EHR-based pharmacovigilance	Malec, S.A.; Wei, P.; Bernstam, E.V.; Boyce, R.D.; Cohen, T.	2021	J. Biomed. Informatics	117			10.1016/j.jbi.2021.103719	Malec 2021	Non-Gastro/Hepatology Focus
Disease spectrum of gastric cancer susceptibility genes	McKinley, S.K.; Singh, P.; Yin, K.; Wang, J.; Zhou, J.; Bao, Y.; Wu, M.; Pathak, K.; Mullen, J.T.; Braun, D.; Hughes, K.S.	2021	Med. Oncol.	38	5		10.1007/s12032-021-01495-w	McKinley 2021	Non-Gastro/Hepatology Focus
Development and Use of Natural Language Processing for Identification of Distant Cancer Recurrence and Sites of Distant Recurrence Using Unstructured Electronic Health Record Data	Karimi, Y.H.; Blayney, D.W.; Kurian, A.W.; Shen, J.; Yamashita, R.; Rubin, D.; Banerjee, I.	2021	JCO Clin Cancer Inform	5		469-478	10.1200/CCI.20.00165	Karimi 2021	Non-Gastro/Hepatology Focus
An imageomics and multi-network	He, T.; Fong, J.N.; Moore,	2021	Comput. Med. Imaging Graph.	89			10.1016/j.compmedimag.2021.101894	He 2021	Non-NLP Focus

based deep learning model for risk assessment of liver transplantation for hepatocellular cancer	L.W.; Ezeana, C.F.; Victor, D.; Divatia, M.; Vasquez, M.; Ghobrial, R.M.; Wong, S.T.C.								
Will machines decipher colonoscopy quality from endoscopistsâ€™ notes?	Pilonis, N.D.; Kaminski, M.F.	2021	Gastrointest. Endosc.	93	3	758-760	10.1016/j.gie.2020.11.021	Pilonis 2021	Review/Perspective Article Only
Exploration of text matching methods in Chinese disease Q&A systems: A method using ensemble based on BERT and boosted tree models	Wu, Z.; Liang, J.; Zhang, Z.; Lei, J.	2021	J. Biomed. Informatics	115			10.1016/j.jbi.2021.103683	Wu 2021	Non-Gastro/Hepatology Focus
Applications of Machine Learning in Human Microbiome Studies: A Review on Feature Selection, Biomarker Identification, Disease Prediction and Treatment	Marcos-Zambrano, L.J.; Karaduzovic-Hadziabdic, K.; Loncar Turukalo, T.; Przymus, P.; Trajkovik, V.; Aasmets, O.; Berland, M.; Gruca, A.; Hasic, J.; Hron, K.; Klammsteiner, T.; Kolev, M.; Lahti, L.; Lopes, M.B.; Moreno, V.; Naskinova, I.; Org, E.;	2021	Front. Microbiol.	12			10.3389/fmicb.2021.634511	Marcos-Zambrano 2021	Review/Perspective Article Only

	PaciÃªncia, I.; Papoutsoglou, G.; Shigdel, R.; Stres, B.; Vilne, B.; Yousef, M.; Zdravevski, E.; Tsamardinos, I.; Carrillo de Santa Pau, E.; Claesson, M.J.; Moreno-Indias, I.; Truu, J.; ML4Microbiome								
Advancing care for acute gastrointestinal bleeding using artificial intelligence	Shung, D.L.	2021	J. Gastroenterol. Hepatol.	36	2	273-278	10.1111/jgh.15372	Shung 2021	Review/Perspective Article Only
Deep learning-based detection of hepatobiliary disorders in ophthalmic imaging	Ahn, J.C.; Shah, V.H.	2021	Lancet Digit. Heal.	3	2	e68-e69	10.1016/S2589-7500(20)30319-8	Ahn 2021	Non-NLP Focus
Combined Spiral Transformation and Model-Driven Multi-Modal Deep Learning Scheme for Automatic Prediction of TP53 Mutation in Pancreatic Cancer	Chen, X.; Lin, X.; Shen, Q.; Qian, X.	2021	IEEE Trans. Med. Imaging	40	2	735-747	10.1109/TMI.2020.3035789	Chen 2021	Non-NLP Focus
Importance of Artificial Intelligence in the Medical Device	Singh, S.; Tiwari, R.; Tiwari, G.	2021	Pharma Times	53	11	21-28		Singh 2021	Review/Perspective Article Only

and Health Care Sector									
Decision-Focused Summarization	Hsu, C.-C.; Tan, C.	2021	EMNLP - Conf. Empir. Methods Nat. Lang. Process., Proc.			117-132		Hsu 2021	Non-Gastro/Hepatology Focus
Using NooJ to Formalize French Cooking Expressions	Yang, T.	2021	Commun. Comput. Info. Sci.	1520 CCIS		147-159	10.1007/978-3-030-92861-2_13	Yang 2021	Non-Gastro/Hepatology Focus
Deep Learning-Based Cervical Spine Posterior Percutaneous Endoscopic Disc Nucleus Resection for the Treatment of Cervical Spondylotic Radiculopathy	Zhang, Y.; Zhu, H.; Zhou, Z.; Sun, Y.; Shen, X.; Wu, J.; Li, C.	2021	J. Healthc. Eng.	2021			10.1155/2021/7245566	Zhang 2021	Non-Gastro/Hepatology Focus
Evaluation of academic performance in virtual environments using the nlp model	Aguayo, R.; Lizarraga, C.; QuiÃ±onez, Y.	2021	Rev. Iberica Sist. Tecnol. Inf.	2021	41	34-49	10.17013/RISTI.41.34-49	Aguayo 2021	Non-Gastro/Hepatology Focus
Role of real-world digital data for orthopedic implant automated surveillance: a systematic review	Dhalluin, T.; Fakhiri, S.; BouzillÃ©, G.; Herbert, J.; Rosset, P.; Cuggia, M.; Grammatico-Guillon, L.	2021	Expert Rev. Med. Devices	18	8	799-810	10.1080/17434440.2021.1943361	Dhalluin 2021	Non-Gastro/Hepatology Focus
Artificial intelligence in gastrointestinal endoscopy for inflammatory bowel disease: a	Tontini, G.E.; Rimondi, A.; Vernero, M.; Neumann, H.; Vecchi, M.;	2021	Ther. Adv. Gastroenterol.	14			10.1177/17562848211017730	Tontini 2021	Non-NLP Focus

systematic review and new horizons	Bezzio, C.; Cavallaro, F.								
Artificial intelligence in colorectal cancer screening, diagnosis and treatment. A new era	Mitsala, A.; Tsalikidis, C.; Pitiakoudis, M.; Simopoulos, C.; Tsaroucha, A.K.	2021	Curr. Oncol.	28	3	1581-1607	10.3390/curroncol28030149	Mitsala 2021	Non-NLP Focus
#Crohn's: Historical Cohort of Twitter Activity	Facanali, M.R.; Bortolozzo Graciolli Facanali, C.; Queiroz, N.S.F.; Sobrado, C.W.; Nahas, S.C.; Safatle-Ribeiro, A.V.	2021	Crohn's Colitis 360	3	1		10.1093/crocol/otaa075	Facanali 2021	Lack of Validation
Multiword Expressions in the Medical Domain: Who Carries the Domain-Specific Meaning	Kocijan, K.; Å ojat, K.; Kurolt, S.	2021	Commun. Comput. Info. Sci.	1389		49-60	10.1007/978-3-030-70629-6_5	Kocijan 2021	Non-Gastro/Hepatology Focus
Automated video summarization and label assignment for otoscopy videos using deep learning and natural language processing	Binol, H.; Niazi, M.K.K.; Elmaraghy, C.; Moberly, A.C.; Gurcan, M.N.	2021	Progr. Biomed. Opt. Imaging Proc. SPIE	11601			10.1117/12.2582009	Binol 2021	Non-Gastro/Hepatology Focus
Development of a predictive model for retention in HIV care using natural language processing of clinical notes	Oliwa, T.; Furner, B.; Schmitt, J.; Schneider, J.; Ridgway, J.P.	2021	J. Am. Med. Informatics Assoc.	28	1	104-112	10.1093/jamia/ocaa220	Oliwa 2021	Non-Gastro/Hepatology Focus

Convolutional neural networks in the computer-aided diagnosis of Helicobacter Pylori infection and non-causal comparison to physician endoscopists: A systematic review with meta-analysis	Mohan, B.P.; Khan, S.R.; Kassab, L.L.; Ponnada, S.; Mohy-Ud-Din, N.; Chandan, S.; Dulai, P.S.; Kochhar, G.S.	2021	Ann. Gastroenterol.	34	1	20-25	10.20524/aog.2020.0542	Mohan 2021	Review/Perspective Article Only
Multi disease-prediction framework using hybrid deep learning: an optimal prediction model	Ampavathi, A.; Saradhi, T.V.	2021	Comput. Methods Biomech. Biomed. Eng.	24	10	1146-1168	10.1080/10255842.2020.1869726	Ampavathi 2021	Non-Gastro/Hepatology Focus
Summarization of Wireless Capsule Endoscopy Video Using Deep Feature Matching and Motion Analysis	Sushma, B.; Aparna, P.	2021	IEEE Access	9		13691-13703	10.1109/ACCESS.2020.3044759	Sushma 2021	Non-NLP Focus
Machine learning in the or: A collaborative environment for surgical interventions in visceral medicine	Ostler, D.; Wilhelm, D.; Kranzfelder, M.; Bernhard, L.; Fuchtmann, J.; Vogel, T.; FeuÃŸner, H.	2021	Surg Technol. Int.	37		16-21		Ostler 2021	Non-Gastro/Hepatology Focus
Predicting Host Phenotype Based on Gut Microbiome Using a Convolutional Neural Network Approach	Reiman, D.; Farhat, A.M.; Dai, Y.	2021	Methods Mol. Biol.	2190		249-266	10.1007/978-1-0716-0826-5_12	Reiman 2021	Non-NLP Focus

Evolving Role and Future Directions of Natural Language Processing in Gastroenterology	Nehme, F.; Feldman, K.	2021	Dig. Dis. Sci.	66	1	29-40	10.1007/s10620-020-06156-y	Nehme 2021	Review/Perspective Article Only
Prevalence and Characteristics of Chronic Cough in Adults Identified by Administrative Data	Zeiger, R.S.; Xie, F.; Schatz, M.; Hong, B.D.; Weaver, J.P.; Bali, V.; Schelfhout, J.; Chen, W.	2020	Perm J	24		01-Mar	10.7812/TPP/20.022	Zeiger 2020	Non-Gastro/Hepatology Focus
Feasibility of deep learning algorithms for reporting in routine spine magnetic resonance imaging	Lewandrowski, K.-U.; Muraleedharan, N.; Eddy, S.A.; Sobti, V.; Reece, B.D.; LeÃ³n, J.F.R.; Shah, S.	2020	Int. J. Spine Surg.	14		S86-S97	10.14444/7131	Lewandrowski 2020	Non-Gastro/Hepatology Focus
Using social media data to understand consumers' information needs and emotions regarding cancer: Ontology-based data analysis study	Lee, J.; Park, H.-A.; Park, S.K.; Song, T.-M.	2020	J. Med. Internet Res.	22	12		10.2196/18767	Lee 2020	Non-Gastro/Hepatology Focus
Artificial intelligence in gastrointestinal endoscopy	Pannala, R.; Krishnan, K.; Melson, J.; Parsi, M.A.; Schulman, A.R.; Sullivan, S.; Trikudanathan, G.; Trindade, A.J.; Watson, R.R.; Maple, J.T.;	2020	VideoGIE	5	12	598-613	10.1016/j.vgie.2020.08.013	Pannala 2020	Review/Perspective Article Only

	Lichtenstein, D.R.								
Language-based translation and prediction of surgical navigation steps for endoscopic wayfinding assistance in minimally invasive surgery	Bieck, R.; Heuermann, K.; Pirlich, M.; Neumann, J.; Neumuth, T.	2020	Int. J. Comput. Assisted Radiol. Surg.	15	12	2089-2100	10.1007/s11548-020-02264-2	Bieck 2020	Non-Gastro/Hepatology Focus
Childrenâ€™s rare disease cohorts: an integrative research and clinical genomics initiative	Rockowitz, S.; LeCompte, N.; Carmack, M.; Quitadamo, A.; Wang, L.; Park, M.; Knight, D.; Sexton, E.; Smith, L.; Sheidley, B.; Field, M.; Holm, I.A.; Brownstein, C.A.; Agrawal, P.B.; Kornetsky, S.; Poduri, A.; Snapper, S.B.; Beggs, A.H.; Yu, T.W.; Williams, D.A.; Sliz, P.	2020	npj Genom. Med.	5	1		10.1038/s41525-020-0137-0	Rockowitz 2020	Non-Gastro/Hepatology Focus
Classification of cancer pathology reports: A large-scale comparative study	Martina, S.; Ventura, L.; Frasconi, P.	2020	IEEE J. Biomedical Health Informat.	24	11	3085-3094	10.1109/JBHI.2020.3005016	Martina 2020	Non-Gastro/Hepatology Focus
Enhancing the oral bioavailability of candesartan cilexetil loaded	Anwar, W.; Dawaba, H.M.; Afouna, M.I.; Samy, A.M.;	2020	Pharmaceutics	12	11	Jan-19	10.3390/pharmaceutics12111047	Anwar 2020	Non-Gastro/Hepatology Focus

nanostructured lipid carriers: In vitro characterization and absorption in rats after oral administration	Rashed, M.H.; Abdelaziz, A.E.								
Sequence labelling and sequence classification with gaze: Novel uses of eye-tracking data for Natural Language Processing	Barrett, M.; Hollenstein, N.	2020	Lang. Linguist. Compass	14	11	Jan-16	10.1111/lnc3.12396	Barrett 2020	Non-Gastro/Hepatology Focus
Social Listening as a Rapid Approach to Collecting and Analyzing COVID-19 Symptoms and Disease Natural Histories Reported by Large Numbers of Individuals	Picone, M.; Inoue, S.; Defelice, C.; Naujokas, M.F.; Sinrod, J.; Cruz, V.A.; Stapleton, J.; Sinrod, E.; Diebel, S.E.; Wassman, E.R.	2020	Popul. Heath. Manage.	23	5	350-360	10.1089/pop.2020.0189	Picone 2020	Non-Gastro/Hepatology Focus
From the pages of AllergyWatchÂ®: October 2020	Fineman, S.M.; Hernandez-Trujillo, V.; Lee, G.B.	2020	Ann. Allergy Asthma Immunol.	125	4	495-496	10.1016/j.anai.2020.07.006	Fineman 2020	Non-Gastro/Hepatology Focus
Nanolipid-loaded Preyssler polyoxometalate: Synthesis, characterization and invitro inhibitory effects on HepG2 tumor cells	Razavi, S.F.; Bamoharram, F.F.; Hashemi, T.; Shahrokhabadi, K.; Davoodnia, A.	2020	Toxicol. Vitro	68			10.1016/j.tiv.2020.104917	Razavi 2020	Non-Gastro/Hepatology Focus
Nanolipoprotein Particles as a Delivery Platform	Darwish, M.; Shatz, W.; Leonard, B.;	2020	Bioconjugate Chem.	31	8	1995-2007	10.1021/acs.bioconjchem.0c00349	Darwish 2020	Non-Gastro/Hepatology Focus

for Fab Based Therapeutics	Loyet, K.; Barrett, K.; Wong, J.L.; Li, H.; Abraham, R.; Lin, M.; Franke, Y.; Tam, C.; Mortara, K.; Zilberleyb, I.; Blanchette, C.								
Self-reported COVID-19 symptoms on Twitter: an analysis and a research resource	Sarker, A.; Lakamana, S.; Hogg-Bremer, W.; Xie, A.; Ali Al-Garadi, M.; Yang, Y.-C.	2020	J. Am. Med. Informatics Assoc.	27	8	1310-1315	10.1093/jamia/ocaa116	Sarker 2020	Non-Gastro/Hepatology Focus
Sarcopenia, frailty and cachexia patients detected in a multisystem electronic health record database	Moorthi, R.N.; Liu, Z.; El-Azab, S.A.; Lembcke, L.R.; Miller, M.R.; Broyles, A.A.; Imel, E.A.	2020	BMC Musculoskelet. Disord.	21	1		10.1186/s12891-020-03522-9	Moorthi 2020	Non-Gastro/Hepatology Focus
Artificial intelligence for understanding imaging, text, and data in gastroenterology	Stidham, R.W.	2020	Gastroenterol. Hepatol.	16	7	341-349		Stidham 2020	Review/Perspective Article Only
Drug vector representation: a tool for drug similarity analysis	Lin, L.; Wan, L.; He, H.; Liu, W.	2020	Mol. Genet. Genomics	295	4	1055-1062	10.1007/s00438-020-01665-x	Lin 2020	Non-Gastro/Hepatology Focus
A dynamic reaction picklist for improving allergy reaction documentation in the electronic health record	Wang, L.; Blackley, S.V.; Blumenthal, K.G.; Yerneni, S.; Goss, F.R.; Lo, Y.-C.; Shah, S.N.; Ortega, C.A.; Korach,	2020	J. Am. Med. Informatics Assoc.	27	6	917-923	10.1093/jamia/ocaa042	Wang 2020	Non-Gastro/Hepatology Focus

	Z.T.; Seger, D.L.; Zhou, L.								
Protective malaria vaccine in mice based on the Plasmodium vivax circumsporozoite protein fused with the mumps nucleocapsid protein	Marques, R.F.; Gimenez, A.M.; Aliprandini, E.; Novais, J.T.; Cury, D.P.; Watanabe, I.-S.; Dominguez, M.R.; Silveira, E.L.V.; Amino, R.; Soares, I.S.	2020	Vaccines	8	2		10.3390/vaccines8020190	Marques 2020	Non-Gastro/Hepatology Focus
A common variant in PNPLA3 is associated with age at diagnosis of NAFLD in patients from a multi-ethnic biobank	Walker, R.W.; Belbin, G.M.; Sorokin, E.P.; Van Vleck, T.; Wojcik, G.L.; Moscati, A.; Gignoux, C.R.; Cho, J.; Abul-Husn, N.S.; Nadkarni, G.; Kenny, E.E.; Loos, R.J.F.	2020	J. Hepatol.	72	6	1070-1081	10.1016/j.jhep.2020.01.029	Walker 2020	NLP used only as a study adjunct/enabler
Integrating Artificial and Human Intelligence: A Partnership for Responsible Innovation in Biomedical Engineering and Medicine	Dzobo, K.; Adotey, S.; Thomford, N.E.; Dzobo, W.	2020	OMICS J. Integr. Biol.	24	5	247-263	10.1089/omi.2019.0038	Dzobo 2020	Non-Gastro/Hepatology Focus
Medi-torch: A new framework for user-friendly hepatobiliary cancer guidelines for patients	Igarashi, T.	2020	Conf Hum Fact Comput Syst Proc				10.1145/3334480.3381438	Igarashi 2020	Non-NLP Focus

Identification of Individuals at Increased Risk for Pancreatic Cancer in a Community-Based Cohort of Patients With Suspected Chronic Pancreatitis	Jeon, C.Y.; Chen, Q.; Yu, W.; Dong, E.Y.; Chung, J.; Pandol, S.J.; Yadav, D.; Conwell, D.L.; Wu, B.U.	2020	Clin Transl Gastroenterol	11	4	e00147	10.14309/ctg.0000000000000147	Jeon 2020	NLP used only as a study adjunct/enabler
Utility of a Computerized ICD-10 Algorithm to Identify Idiosyncratic Drug-Induced Liver Injury Cases in the Electronic Medical Record	Yeboah-Korang, A.; Louissaint, J.; Tsung, I.; Prabhu, S.; Fontana, R.J.	2020	Drug Saf.	43	4	371-377	10.1007/s40264-019-00903-5	Yeboah-Korang 2020	Non-NLP Focus
Convolutional neural network technology in endoscopic imaging: Artificial intelligence for endoscopy	Choi, J.; Shin, K.; Jung, J.; Bae, H.-J.; Kim, D.H.; Byeon, J.-S.; Kim, N.	2020	Clin. Endosc.	53	2	117-126	10.5946/ce.2020.054	Choi 2020	Non-NLP Focus
FIB-4 stage of liver fibrosis is associated with incident heart failure with preserved, but not reduced, ejection fraction among people with and without HIV or hepatitis C	So-Armah, K.A.; Lim, J.K.; Lo Re, V., III; Tate, J.P.; Chang, C.-C.H.; Butt, A.A.; Gibert, C.L.; Rimland, D.; Marconi, V.C.; Goetz, M.B.; Ramachandran, V.; Brittain, E.; Long, M.; Nguyen, K.-L.; Rodriguez-Barradas, M.C.;	2020	Prog. Cardiovasc. Dis.	63	2	184-191	10.1016/j.pcad.2020.02.010	So-Armah 2020	NLP used only as a study adjunct/enabler

	Budoff, M.J.; Tindle, H.A.; Samet, J.H.; Justice, A.C.; Freiberg, M.S.; VACS Project Team								
Prediction of Pancreatic Cancer Based on Imaging Features in Patients with Duct Abnormalities	Chen, W.; Butler, R.K.; Zhou, Y.; Parker, R.A.; Jeon, C.Y.; Wu, B.U.	2020	Pancreas	49	3	413-419	10.1097/MPA.0000000000001499	Chen 2020	NLP used only as a study adjunct/enabler
Variation in Serious Illness Communication among Surgical Patients Receiving Palliative Care	Udelsman, B.V.; Lee, K.C.; Lilley, E.J.; Chang, D.C.; Lindvall, C.; Cooper, Z.	2020	J. Palliative Med.	23	3	411-414	10.1089/jpm.2019.0268	Udelsman 2020	Non-Gastro/Hepatology Focus
Outcome of optic nerve decompression through neuroendoscopic transethmoidal-sphenoidal approach in the treatment of traumatic optic neuropathy	Xu, Y.; Xue, Y.; Tang, J.; Lin, S.; Zhang, A.; Lou, M.	2020	Chin. J. Neurosurg.	36	2	168-172	10.3760/cma.j.issn.1001-2346.2020.02.012	Xu 2020	Non-Gastro/Hepatology Focus
Using SNOMED to automate clinical concept mapping	Gupta, S.; Dieleman, F.; Long, P.; Doyle, O.; Leavitt, N.	2020	ACM CHIL - Proc. ACM Conf. Health, Inference, Learn.			131-138	10.1145/3368555.3384453	Gupta 2020	Non-Gastro/Hepatology Focus
Expert artificial intelligence-based natural language processing characterises childhood asthma	Seol, H.Y.; Rolfes, M.C.; Chung, W.; Sohn, S.; Ryu, E.; Park, M.A.; Kita, H.; Ono, J.;	2020	BMJ Open Respir. Res.	7	1		10.1136/bmjresp-2019-000524	Seol 2020	Non-Gastro/Hepatology Focus

	Croghan, I.; Armasu, S.M.; Castro-Rodriguez, J.A.; Weston, J.D.; Liu, H.; Juhn, Y.								
Adverse drug event rates in pediatric pulmonary hypertension: A comparison of real-world data sources	Geva, A.; Abman, S.H.; Manzi, S.F.; Ivy, D.D.; Mullen, M.P.; Griffin, J.; Lin, C.; Savova, G.K.; Mandl, K.D.	2020	J. Am. Med. Informatics Assoc.	27	2	294-300	10.1093/jamia/ocz194	Geva 2020	Non-Gastro/Hepatology Focus
Type B reflexivization as an unambiguous testbed for multilingual multi-task gender bias	GonzÃ¡lez, A.V.; Barrett, M.; Hvingelby, R.; Webster, K.; SÃ¸gaard, A.	2020	EMNLP - Conf. Empir. Methods Nat. Lang. Process., Proc. Conf.			2637-2648		GonzÃ¡lez 2020	Non-Gastro/Hepatology Focus
Machine learning of fecal metabolites of children with autism spectrum disorder during microbiota transfer therapy	Qureshi, F.; Adams, J.; Hanagan, K.; Kang, D.-W.; Krajmalnik-Brown, R.; Hahn, J.	2020	AIChE Annu. Meet. Conf. Proc.	2020-November				Qureshi 2020	Non-Gastro/Hepatology Focus
Bleeding Entity Recognition in Electronic Health Records: A Comprehensive Analysis of End-to-End Systems	Mitra, A.; Rawat, B.P.S.; McManus, D.; Kapoor, A.; Yu, H.	2020	AMIA Annu Symp Proc	2020		860-869		Mitra 2020	Non-Gastro/Hepatology Focus
Clinical annotation research kit (CLARK): Computable	Pfaff, E.R.; Crosskey, M.; Morton, K.; Krishnamurthy, A.	2020	JMIR Med. Inform.	8	1		10.2196/16042	Pfaff 2020	Non-Gastro/Hepatology Focus

phenotyping using machine learning									
Orcokinin neuropeptides regulate sleep in Caenorhabditis elegans	Honer, M.; Buscemi, K.; Barrett, N.; Riazati, N.; Orlando, G.; Nelson, M.D.	2020	J. Neurogenet.	34	03-Apr	440-452	10.1080/01677063.2020.1830084	Honer 2020	Non-Gastro/Hepatology Focus
Can a web accessibility checker be enhanced by the use of ai?	Draffan, E.A.; Ding, C.; Wald, M.; Everett, H.; Barrett, J.; Sasikant, A.; Geangu, C.; Newman, R.	2020	Lect. Notes Comput. Sci.	12376 LNCS		67-73	10.1007/978-3-030-58796-3_9	Draffan 2020	Non-Gastro/Hepatology Focus
Precision medicine for human cancers with Notch signaling dysregulation (Review)	Katoh, M.	2020	Int. J. Mol. Med.	45	2	279-297	10.3892/ijmm.2019.4418	Katoh 2020	Non-Gastro/Hepatology Focus
Deficits in the Palliative Care Process Measures in Patients with Advanced Pancreatic Cancer Undergoing Operative and Invasive Nonoperative Palliative Procedures	Udelsman, B.V.; Lilley, E.J.; Qadan, M.; Chang, D.C.; Lillemoe, K.D.; Lindvall, C.; Cooper, Z.	2019	Ann. Surg. Oncol.	26	13	4204-4212	10.1245/s10434-019-07757-2	Udelsman 2019	NLP used only as a study adjunct/enabler
Polygenic prediction via Bayesian regression and continuous shrinkage priors	Ge, T.; Chen, C.-Y.; Ni, Y.; Feng, Y.-C.A.; Smoller, J.W.	2019	Nat. Commun.	10	1		10.1038/s41467-019-09718-5	Ge 2019	Non-Gastro/Hepatology Focus

Semantic computational analysis of anticoagulation use in atrial fibrillation from real world data	Bean, D.M.; Teo, J.; Wu, H.; Oliveira, R.; Patel, R.; Bendayan, R.; Shah, A.M.; Dobson, R.J.B.; Scott, P.A.	2019	PLoS ONE	14	11		10.1371/journal.pone.0225625	Bean 2019	Non-Gastro/Hepatology Focus
Current status of artificial intelligence applications in urology and their potential to influence clinical practice	Chen, J.; Remulla, D.; Nguyen, J.H.; Aastha, D.; Liu, Y.; Dasgupta, P.; Hung, A.J.	2019	BJU Int.	124	4	567-577	10.1111/bju.14852	Chen 2019	Non-Gastro/Hepatology Focus
Analysis of usage of term weighting algorithm for mapping health procedures into the unified terminology of supplemental health (TUSS)	Saraiva Barretto, E.H.; Da Costa Patrao, D.F.; Itoa, M.	2019	Stud. Health Technol. Informatics	264		1496-1497	10.3233/SHTI190502	SaraivaBarretto 2019	Non-Gastro/Hepatology Focus
A natural language processing framework to analyse the opinions on HPV vaccination reflected in twitter over 10 years (2008 - 2017)	Luo, X.; Zimet, G.; Shah, S.	2019	Hum. Vaccines Immunother.	15	07-Aug	1496-1504	10.1080/21645515.2019.1627821	Luo 2019	Non-Gastro/Hepatology Focus
Using twitter to understand the human bowel disease community: Exploratory	PÃ©rez-PÃ©rez, M.; PÃ©rez-RodrÃ-guez, G.; Fdez-Riverola, F.; LourenÃ§o, A.	2019	J. Med. Internet Res.	21	8		10.2196/12610	PÃ©rez-PÃ©rez 2019	Lack of Validation

analysis of key topics									
Detecting adverse drug reactions in discharge summaries of electronic medical records using Readpeer	Tang, Y.; Yang, J.; Ang, P.S.; Dorajoo, S.R.; Foo, B.; Soh, S.; Tan, S.H.; Tham, M.Y.; Ye, Q.; Shek, L.; Sung, C.; Tung, A.	2019	Int. J. Med. Informatics	128		62-70	10.1016/j.ijmedinf.2019.04.017	Tang 2019	Non-Gastro/Hepatology Focus
Quality of Documentation of Contrast Agent Allergies in Electronic Health Records	Deng, F.; Li, M.D.; Wong, A.; Kowalski, L.T.; Lai, K.H.; Digumarthy, S.R.; Zhou, L.	2019	J. Am. Coll. Radiol.	16	8	1027-1035	10.1016/j.jacr.2019.01.027	Deng 2019	Non-Gastro/Hepatology Focus
Development of a global infectious disease activity database using natural language processing, machine learning, and human expertise	Feldman, J.; Thomas-Bachli, A.; Forsyth, J.; Patel, Z.H.; Khan, K.	2019	J. Am. Med. Informatics Assoc.	26	11	1355-1359	10.1093/jamia/ocz112	Feldman 2019	Non-Gastro/Hepatology Focus
GWAS and enrichment analyses of non-alcoholic fatty liver disease identify new trait-associated genes and pathways across eMERGE Network	Namjou, B.; Lingren, T.; Huang, Y.; Parameswaran, S.; Cobb, B.L.; Stanaway, I.B.; Connolly, J.J.; Mentch, F.D.; Benoit, B.; Niu, X.; Wei, W.-Q.; Carroll, R.J.; Pacheco, J.A.; Harley, I.T.W.; Divanovic, S.; Carrell, D.S.;	2019	BMC Med.	17	1		10.1186/s12916-019-1364-z	Namjou 2019	NLP used only as a study adjunct/enabler

	Larson, E.B.; Carey, D.J.; Verma, S.; Ritchie, M.D.; Gharavi, A.G.; Murphy, S.; Williams, M.S.; Crosslin, D.R.; Jarvik, G.P.; Kullo, I.J.; Hakonarson, H.; Li, R.; Xanthakos, S.A.; Harley, J.B.								
Apixaban for Routine Management of Upper Extremity Deep Venous Thrombosis (ARM-DVT): Methods of a prospective single-arm management study	Woller, S.C.; Stevens, S.M.; Johnson, S.A.; Bledsoe, J.R.; Galovic, B.; Lloyd, J.F.; Wilson, E.L.; Armbruster, B.; Evans, R.S.	2019	Res. Pract. Thromb. Haemost.	3	3	340-348	10.1002/rth2.12208	Woller 2019	Non-Gastro/Hepatology Focus
Association Between Endoscopist Personality and Rate of Adenoma Detection	Ezaz, G.; Leffler, D.A.; Beach, S.; Schoen, R.E.; Crockett, S.D.; Gourevitch, R.A.; Rose, S.; Morris, M.; Carrell, D.S.; Greer, J.B.; Mehrotra, A.	2019	Clin. Gastroenterol. Hepatol.	17	8	1571-1579.e7	10.1016/j.cgh.2018.10.019	Ezaz 2019	Non-NLP Focus
Concordance Study Between IBM Watson for Oncology and Clinical Practice	Zhou, N.; Zhang, C.-T.; Lv, H.-Y.; Hao, C.-X.; Li, T.-J.; Zhu, J.-J.; Zhu, H.; Jiang,	2019	Oncologist	24	6	812-819	10.1634/theoncologist.2018-0255	Zhou 2019	Non-Gastro/Hepatology Focus

for Patients with Cancer in China	M.; Liu, K.-W.; Hou, H.-L.; Liu, D.; Li, A.-Q.; Zhang, G.-Q.; Tian, Z.-B.; Zhang, X.-C.								
iEnhancer-5Step: Identifying enhancers using hidden information of DNA sequences via Chou's 5-step rule and word embedding	Le, N.Q.K.; Yapp, E.K.Y.; Ho, Q.-T.; Nagasundaram, N.; Ou, Y.-Y.; Yeh, H.-Y.	2019	Anal. Biochem.	571		53-61	10.1016/j.ab.2019.02.017	Le 2019	Non-Gastro/Hepatology Focus
Bias of Inaccurate Disease Mentions in Electronic Health Record-based Phenotyping	Kagawa, R.; Shinohara, E.; Imai, T.; Kawazoe, Y.; Ohe, K.	2019	Int. J. Med. Informatics	124		90-96	10.1016/j.ijmedinf.2018.12.004	Kagawa 2019	Non-Gastro/Hepatology Focus
Using natural language processing to extract clinically useful information from Chinese electronic medical records	Chen, L.; Song, L.; Shao, Y.; Li, D.; Ding, K.	2019	Int. J. Med. Informatics	124		06-Dec	10.1016/j.ijmedinf.2019.01.004	Chen 2019	Non-Gastro/Hepatology Focus
Scalable nonlinear programming framework for parameter estimation in dynamic biological system models	Shin, S.; Venturelli, O.S.; Zavala, V.M.	2019	PLoS Comput. Biol.	15	3		10.1371/journal.pcbi.1006828	Shin 2019	Non-Gastro/Hepatology Focus
General hospital admission rates in people diagnosed	Fok, M.L.Y.; Chang, C.-K.; Broadbent, M.;	2019	Acta Psychiatr. Scand.	139	3	248-255	10.1111/acps.13004	Fok 2019	Non-Gastro/Hepatology Focus

with personality disorder	Stewart, R.; Moran, P.								
Evaluation and accurate diagnoses of pediatric diseases using artificial intelligence	Liang, H.; Tsui, B.Y.; Ni, H.; Valentim, C.C.S.; Baxter, S.L.; Liu, G.; Cai, W.; Kermany, D.S.; Sun, X.; Chen, J.; He, L.; Zhu, J.; Tian, P.; Shao, H.; Zheng, L.; Hou, R.; Hewett, S.; Li, G.; Liang, P.; Zang, X.; Zhang, Z.; Pan, L.; Cai, H.; Ling, R.; Li, S.; Cui, Y.; Tang, S.; Ye, H.; Huang, X.; He, W.; Liang, W.; Zhang, Q.; Jiang, J.; Yu, W.; Gao, J.; Ou, W.; Deng, Y.; Hou, Q.; Wang, B.; Yao, C.; Liang, Y.; Zhang, S.; Duan, Y.; Zhang, R.; Gibson, S.; Zhang, C.L.; Li, O.; Zhang, E.D.; Karin, G.; Nguyen, N.; Wu, X.; Wen, C.; Xu, J.; Xu, W.; Wang, W.; Li, J.; Pizzato, B.; Bao, C.; Xiang, D.;	2019	Nat. Med.	25	3	433-438	10.1038/s41591-018-0335-9	Liang 2019	Non-Gastro/Hepatology Focus

	He, S.; Zhou, Y.; Haw, W.; Goldbaum, M.; Tremoulet, A.; Hsu, C.-N.; Carter, H.; Zhu, L.; Zhang, K.; Xia, H.								
Machine Learning Can Improve Estimation of Surgical Case Duration: A Pilot Study	Tuwatananurak, J.P.; Zadeh, S.; Xu, X.; Vacanti, J.A.; Fulton, W.R.; Ehrenfeld, J.M.; Urman, R.D.	2019	J. Med. Syst.	43	3		10.1007/s10916-019-1160-5	Tuwatananurak 2019	Non-Gastro/Hepatology Focus
Natural Language Processing to Assess End-of-Life Quality Indicators in Cancer Patients Receiving Palliative Surgery	Lindvall, C.; Lilley, E.J.; Zupanc, S.N.; Chien, I.; Udelsman, B.V.; Walling, A.; Cooper, Z.; Tulsky, J.A.	2019	J. Palliative Med.	22	2	183-187	10.1089/jpm.2018.0326	Lindvall 2019	Non-Gastro/Hepatology Focus
Special Session - Scaling Automated Scoring: Addressing Practical and Conceptual Challenges	Barrett, M.D.; Lazendic, G.	2019	Proc. IEEE Int. Conf. Teach., Assess., Learn. Eng., TALE			1223-1224	10.1109/TALE.2018.8615198	Barrett 2019	Non-Gastro/Hepatology Focus
Ontology-Based Process for Unstructured Medical Report Mapping	Oliva, J.T.; Lee, H.D.; SpolaÃ´r, N.; Wu, F.C.; Coy, C.S.R.; Fagundes, J.J.; de Lourdes Setsuko Ayrizono, M.	2019	Machine Learning in Bio-Signal Analysis and Diagnostic Imaging			Jan-18	10.1016/B978-0-12-816086-2.00001-1	Oliva 2019	Non-Gastro/Hepatology Focus

Detection of bleeding events in electronic health record notes using convolutional neural network models enhanced with recurrent neural network autoencoders: Deep learning approach	Li, R.; Hu, B.; Liu, F.; Liu, W.; Cunningham, F.; McManus, D.D.; Yu, H.	2019	JMIR Med. Inform.	7	1		10.2196/10788	Li 2019	Non-Gastro/Hepatology Focus
Adversarial removal of demographic attributes revisited	Barrett, M.; Kementchedjhieva, Y.; Elazar, Y.; Elliott, D.; SÃ¸gaard, A.	2019	EMNLP-IJCNLP - Conf. Empir. Methods Nat. Lang. Process. Int. Jt. Conf. Nat. Lang. Process., Proc. Conf.			6330-6335		Barrett 2019	Non-NLP Focus
SuÅ›ruta: Artificial intelligence and bayesian knowledge network in health care â€“ smartphone apps for diagnosis and differentiation of anemias with higher accuracy at resource constrained point-of-care settings	Yadav, S.; Ganesh, S.; Das, D.; Venkanna, U.; Mahapatra, R.; Shrivastava, A.K.; Chakrabarti, P.; Talukder, A.K.	2019	Lect. Notes Comput. Sci.	11932 LNCS		159-175	10.1007/978-3-030-37188-3_10	Yadav 2019	Non-Gastro/Hepatology Focus
Obtaining knowledge in pathology reports through a natural language processing approach with classification, named-entity	Oliwa, T.; Maron, S.B.; Chase, L.M.; Lomnicki, S.; Catenacci, D.V.T.; Furner, B.; Volchenboum, S.L.	2019	JCO Clin. Cancer Inform.	3		01-Aug	10.1200/CCI.19.00008	Oliwa 2019	Non-Gastro/Hepatology Focus

recognition, and relation-extraction heuristics									
A virtual counseling application using artificial intelligence for communication skills training in nursing education: Development study	Shorey, S.; Ang, E.; Yap, J.; Ng, E.D.; Lau, S.T.; Chui, C.K.	2019	J. Med. Internet Res.	21	10		10.2196/14658	Shorey 2019	Non-Gastro/Hepatology Focus
Combination of active transfer learning and natural language processing to improve liver volumetry using surrogate metrics with deep learning	Marinelli, B.; Kang, M.; Martini, M.; Zech, J.R.; Titano, J.; Cho, S.; Costa, A.B.; Oermann, E.K.	2019	Radiology: Art. Int.	1	1		10.1148/ryai.2019180019	Marinelli 2019	NLP used only as a study adjunct/enabler
Thyroid function screening in children and adolescents with mood and anxiety disorders	Luft, M.J.; Aldrich, S.L.; Poweleit, E.; Prows, C.A.; Martin, L.J.; DelBello, M.P.; Keeshin, B.R.; Ramsey, L.B.; Strawn, J.R.	2019	J. Clin. Psychiatry	80	5		10.4088/JCP.18m12626	Luft 2019	Non-Gastro/Hepatology Focus
Design of a Novel Web Utility that Provides Multi-lingual Word Definitions for Child E-Book Applications	Adiani, D.; Lewis, D.; Serao, V.; Barrett, K.; Bennett, A.; Hambly, D.; Shenoda, M.; West, S.; Coulter, G.; Shagal, S.; Biala,	2019	Lect. Notes Comput. Sci.	11591 LNCS		03-Dec	10.1007/978-3-030-21817-1_1	Adiani 2019	Non-Gastro/Hepatology Focus

	T.; Sarkar, M.; Wade, J.; Sarkar, N.								
Percutaneous nephrolithotomy in patients with bleeding disorders: Case report	Monroy, R.E.; Nieto, D.C.; Fierro, M.C.	2019	Urol. Colomb.	28	1	43-46	10.1055/s-0038-1645850	Monroy 2019	Non-Gastro/Hepatology Focus
Cloud-based conversational agents for user acquisition and engagement	Bello, M.J.G.	2019	CLOSER - Proc. Int. Conf. Cloud Comput. Serv. Sci.			528-534	10.5220/0007766105280534	Bello 2019	Non-Gastro/Hepatology Focus
GIDB: A knowledge database for the automated curation and multidimensional analysis of molecular signatures in gastrointestinal cancer	Wang, Y.; Wang, S.; Tong, Y.; Jin, L.; Zong, H.; Zheng, R.; Yang, J.; Zhang, Z.; Ouyang, E.; Zhou, M.; Zhang, X.	2019	Database	2019	1		10.1093/database/baz051	Wang 2019	Risk Factors For Disease Only
Nucleobindins and encoded peptides: From cell signaling to physiology	Leung, A.K.-W.; Ramesh, N.; Vogel, C.; Unniappan, S.	2019	Adv. Protein Chem. Struct. Biol.	116		91-133	10.1016/bs.apcsb.2019.02.001	Leung 2019	Non-Gastro/Hepatology Focus
Investigation of the clinical significance and molecular mechanism of miR-21-5p in hepatocellular carcinoma: A systematic review based on 24 studies and	Zhong, X.-Z.; Deng, Y.U.N.; Chen, G.; Yang, H.	2019	Oncol. Lett.	17	1	230-246	10.3892/ol.2018.9627	Zhong 2019	Non-Gastro/Hepatology Focus

bioinformatics investigation									
G2Vec: Distributed gene representations for identification of cancer prognostic genes	Choi, J.; Oh, I.; Seo, S.; Ahn, J.	2018	Sci. Rep.	8	1		10.1038/s41598-018-32180-0	Choi 2018	Non-Gastro/Hepatology Focus
Predictions of novel Schistosoma mansoni - human protein interactions consistent with experimental data	White Bear, J.; Long, T.; Skinner, D.; McKerrow, J.H.	2018	Sci. Rep.	8	1		10.1038/s41598-018-31272-1	WhiteBear 2018	NLP used only as a study adjunct/enabler
Postoperative bleeding risk prediction for patients undergoing colorectal surgery	Chen, D.; Afzal, N.; Sohn, S.; Habermann, E.B.; Naessens, J.M.; Larson, D.W.; Liu, H.	2018	Surgery	164	6	1209-1216	10.1016/j.surg.2018.05.043	Chen 2018	Non-NLP Focus
A new laparoscopic triangle fixation technique for gastrostomy: A safe and effective procedure for reduction of the wound infection rate	Fujiogi, M.; Tanaka, Y.; Amano, H.; Deie, K.; Suzuki, K.; Kawashima, H.; Murase, N.; Uchida, H.	2018	Nagoya J. Med. Sci.	80	4	497-503	10.18999/nagjms.80.4.497	Fujiogi 2018	Non-NLP Focus
Fragmentation targeted at preferred discontinuities: A new concept in endolithotripsy with Holmium laser:YAG	SÃ¡nchez-MartÃn, F.M.; Emiliani, E.; Pueyo-Morer, E.; Angerri-Feu, O.; Sanguedolce, F.; MillÃ¡n, F.; Villavicencio, H.	2018	Actas Urol. Esp.	42	9	606-609	10.1016/j.acuro.2017.11.008	SÃ¡nchez-MartÃn 2018	Non-Gastro/Hepatology Focus

The association between arthralgia and vedolizumab using natural language processing	Cai, T.; Lin, T.-C.; Bond, A.; Huang, J.; Kane-Wanger, G.; Cagan, A.; Murphy, S.N.; Ananthakrishnan, A.N.; Liao, K.P.	2018	Inflammatory Bowel Dis.	24	10	2242-2246	10.1093/ibd/izy127	Cai 2018	NLP used only as a study adjunct/enabler
Learning predictive models of drug side-effect relationships from distributed representations of literature-derived semantic predications	Mower, J.; Subramanian, D.; Cohen, T.	2018	J. Am. Med. Informatics Assoc.	25	10	1339-1350	10.1093/jamia/ocy077	Mower 2018	Non-Gastro/Hepatology Focus
Discussion of artificial intelligence application in medical imaging	Xiao, Y.; Xia, C.; Zhang, R.-G.; Liu, S.-Y.	2018	Acad. J. Second Mil. Med. Univ.	39	8	813-818	10.16781/j.0258-879x.2018.08.0813	Xiao 2018	Non-Gastro/Hepatology Focus
Correlation between neutrophil/lymphocyte ratio and Ranson score in acute pancreatitis	AbaylÄ±, B.; GenÃ§dal, G.; DeÄŸirmencioÄŸlu, Åž.	2018	J. Clin. Lab. Anal.	32	6		10.1002/jcla.22437	AbaylÄ± 2018	Non-NLP Focus
Outcome of endoscopic trans-ethmosphenoid optic canal decompression for indirect traumatic optic neuropathy in children	Yu, B.; Chen, Y.; Ma, Y.; Tu, Y.; Wu, W.	2018	BMC Ophthalmol.	18	1		10.1186/s12886-018-0792-4	Yu 2018	Non-Gastro/Hepatology Focus
A value set for documenting adverse reactions	Goss, F.R.; Lai, K.H.; Topaz, M.; Acker, W.W.;	2018	J. Am. Med. Informatics Assoc.	25	6	661-669	10.1093/jamia/ocx139	Goss 2018	Non-Gastro/Hepatology Focus

in electronic health records	Kowalski, L.; Plasek, J.M.; Blumenthal, K.G.; Seger, D.L.; Slight, S.P.; Fung, K.W.; Chang, F.Y.; Bates, D.W.; Zhou, L.								
Endoscopist factors that influence serrated polyp detection: A multicenter study	Crockett, S.D.; Gourevitch, R.A.; Morris, M.; Carrell, D.S.; Rose, S.; Shi, Z.; Greer, J.B.; Schoen, R.E.; Mehrotra, A.	2018	Endoscopy	50	10	984-992	10.1055/a-0597-1740	Crockett 2018	NLP used only as a study adjunct/enabler
Use of text-mining methods to improve efficiency in the calculation of drug exposure to support pharmacoepidemiology studies	McTaggart, S.; Nangle, C.; Caldwell, J.; Alvarez-Madrazo, S.; Colhoun, H.; Bennie, M.	2018	Int. J. Epidemiol.	47	2	617-624	10.1093/IJE/DYX264	McTaggart 2018	Non-Gastro/Hepatology Focus
Adenoma Detection Rate Falls at the End of the Day in a Large Multi-site Sample	Marcondes, F.O.; Gourevitch, R.A.; Schoen, R.E.; Crockett, S.D.; Morris, M.; Mehrotra, A.	2018	Dig. Dis. Sci.	63	4	856-859	10.1007/s10620-018-4947-1	Marcondes 2018	Non-NLP Focus
MicroRNA-124-3p expression and its prospective functional pathways in hepatocellular carcinoma: A quantitative	He, R.-Q.; Yang, X.; Liang, L.; Chen, G.; Ma, J.	2018	Oncol. Lett.	15	4	5517-5532	10.3892/ol.2018.8045	He 2018	NLP used only as a study adjunct/enabler

polymerase chain reaction, gene expression omnibus and bioinformatics study									
Comparison of adverse event and device problem rates for transcatheter aortic valve replacement and Mitraclip procedures as reported by the Transcatheter Valve Therapy Registry and the Food and Drug Administration postmarket surveillance data	Galper, B.Z.; Beery, D.E.; Leighton, G.; Englander, L.L.	2018	Am. Heart J.	198		64-74	10.1016/j.ahj.2017.10.013	Galper 2018	Non-Gastro/Hepatology Focus
Physician characteristics associated with higher adenoma detection rate	Mehrotra, A.; Morris, M.; Gourevitch, R.A.; Carrell, D.S.; Leffler, D.A.; Rose, S.; Greer, J.B.; Crockett, S.D.; Baer, A.; Schoen, R.E.	2018	Gastrointest. Endosc.	87	3	778-786.e5	10.1016/j.gie.2017.08.023	Mehrotra 2018	NLP used only as a study adjunct/enabler
Potential role of microRNA-223-3p in the tumorigenesis of hepatocellular carcinoma: A comprehensive	Zhang, R.; Zhang, L.-J.; Yang, M.-L.; Huang, L.-S.; Chen, G.; Feng, Z.-B.	2018	Mol. Med. Rep.	17	2	2211-2228	10.3892/mmr.2017.8167	Zhang 2018	NLP used only as a study adjunct/enabler

study based on data mining and bioinformatics									
Common variable immunodeficiency non-infectious disease endotypes redefined using unbiased network clustering in large electronic datasets	Farmer, J.R.; Ong, M.-S.; Barmettler, S.; Yonker, L.M.; Fuleihan, R.; Sullivan, K.E.; Cunningham-Rundles, C.; Walter, J.E.; Lugar, P.; Suez, D.; Routes, J.; Bonilla, F.A.; Kleiner, G.; Ballas, Z.K.; Secord, E.A.; Buckley, R.; Joshi, A.; Akhter, J.; Puck, J.; Haddad, E.; Calabrese, L.; Strober, W.; Patel, N.C.; Ochs, H.D.; Uygungil, B.; Stein, M.R.; Chen, K.; Ballow, M.; Bennett, N.; Lehman, H.; Dorsey, M.; Fernandez, J.; Caldwell, J.; Hostoffer, R.; Knight, A.; Shapiro, R.; Apter, A.J.; Bennion, J.R.;	2018	Front. Immunol.	8	JAN		10.3389/fimmu.2017.01740	Farmer 2018	Non-Gastro/Hepatology Focus

	Berger, M.; Calderon, J.; Cheng, L.; Cooper, M.; Reis, P.C.; George, C.; Gonzalez, G.E.; Guillot, R.J.; Gundling, K.E.; Hernandez-Trujillo, V.; Kirkpatrick, C.H.; Kobayashi, R.H.; Lowe, D.; Muskat, M.; Notarangelo, L.; Overby, T.L.; Rabinowitz, R.; Tanner, B.; White, M.; Wright, D.; Yu, G.; The USIDNET Consortium								
Sequence classification with human attention	Barrett, M.; Bingel, J.; Hollenstein, N.; Rei, M.; SÃ¸gaard, A.	2018	CoNLL - Conf. Comput. Nat. Lang. Learn., Proc.			302-312	10.18653/v1/k18-1030	Barrett 2018	Non-Gastro/Hepatology Focus
Percutaneous nephrolithotomy ambulatory: Case series and literature review	GarcÃa, C.E.H.; Chaparro, D.; Ramos, G.; de la Hoz, J.	2018	Urol. Colomb.	27	3	260-265	10.1055/s-0038-1645843	GarcÃa 2018	Non-Gastro/Hepatology Focus
Trigger words detection by integrating attention mechanism into Bi-LSTM neural	Zhou, K.; Yao, X.; Wang, S.; Kim, J.-D.; Cohen, K.B.; Chen, R.; Wang, Y.; Xia, J.	2018	Lect. Notes Comput. Sci.	11221 LNAI		398-409	10.1007/978-3-030-01716-3_33	Zhou 2018	Risk Factors For Disease Only

networkâ€"A case study in PubMED-wide trigger words detection for pancreatic cancer									
Detection of serrated polyps: How do endoscopists rate?	Anderson, J.C.	2018	Endoscopy	50	10	950-952	10.1055/a-0637-9072	Anderson 2018	Non-NLP Focus
Polypeptide-decorated nanoliposomes as novel delivery systems for lutein	Jiao, Y.; Li, D.; Liu, C.; Chang, Y.; Song, J.; Xiao, Y.	2018	RSC Adv.	8	55	31372-31381	10.1039/c8ra05838e	Jiao 2018	Non-Gastro/Hepatology Focus
SemEHR: A general-purpose semantic search system to surface semantic data from clinical notes for tailored care, trial recruitment, and clinical research	Wu, H.; Toti, G.; Morley, K.I.; Ibrahim, Z.M.; Folarin, A.; Jackson, R.; Kartoglu, I.; Agrawal, A.; Stringer, C.; Gale, D.; Gorrell, G.; Roberts, A.; Broadbent, M.; Stewart, R.; Dobson, R.J.B.	2018	J. Am. Med. Informatics Assoc.	25	5	530-537	10.1093/JAMIA/OCX160	Wu 2018	Part of Larger Study
Kaibel Column: Modeling and Optimization	Soraya Lopez-Saucedo, E.; Chen, Q.; Grossmann, I.E.; Caballero, J.A.	2018	Comput. Aided Chem. Eng.	44		1183-1188	10.1016/B978-0-444-64241-7.50192-0	SorayaLopez-Saucedo 2018	Non-Gastro/Hepatology Focus
Lactobacillus spp. belonging to the Casei group display a variety of adhesins	Konieczna, C.; Olejnik-Schmidt, A.; Schmidt, M.T.	2018	Acta Sci. Pol. Technol. Aliment.	17	1	69-82	10.17306/J.AFS.2018.0538	Konieczna 2018	Non-NLP Focus
Analysis of microarrays of	Ren, F.-H.; Yang, H.; He, R.; Lu, J.;	2018	BMC Cancer	18	1		10.1186/s12885-017-3941-x	Ren 2018	Non-NLP Focus

miR-34a and its identification of prospective target gene signature in hepatocellular carcinoma	Lin, X.; Liang, H.-W.; Dang, Y.-W.; Feng, Z.-B.; Chen, G.; Luo, D.-Z.								
Diagnostic value of strand-specific miRNA-101-3p and miRNA-101-5p for hepatocellular carcinoma and a bioinformatic analysis of their possible mechanism of action	Yang, X.; Pang, Y.-Y.; He, R.-Q.; Lin, P.; Cen, J.-M.; Yang, H.; Ma, J.; Chen, G.	2018	FEBS Open Bio.	8	1	64-84	10.1002/2211-5463.12349	Yang 2018	NLP used only as a study adjunct/enabler
Content-based processing and analysis of endoscopic images and videos: A survey	MÃ¼nzer, B.; Schoeffmann, K.; BÃ¶szÃ¶rmenyi, L.	2018	Multimedia Tools Appl	77	1	1323-1362	10.1007/s11042-016-4219-z	MÃ¼nzer 2018	Non-NLP Focus
Clinical features of visual disturbances secondary to isolated sphenoid sinus inflammatory diseases	Chen, L.; Jiang, L.; Yang, B.; Subramanian, P.S.	2017	BMC Ophthalmol	17	1	237	10.1186/s12886-017-0634-9	Chen 2017	Non-Gastro/Hepatology Focus
Comparison of outcomes between endoscopic and transcleral cyclophotocoagulation	Beardsley, R.; Law, S.K.; Caprioli, J.; Coleman, A.L.; Nouri-Mahdavi, K.; Hubschman, J.-P.; Schwartz, S.D.; Giaconi, J.A.	2017	Vision (Switzerland)	1	4		10.3390/vision1040024	Beardsley 2017	Non-NLP Focus

A qRT-PCR and Gene Functional Enrichment Study Focused on Downregulation of miR-141-3p in Hepatocellular Carcinoma and Its Clinicopathological Significance	Liu, C.-Z.; Ye, Z.-H.; Ma, J.; He, R.-Q.; Liang, H.-W.; Peng, Z.-G.; Chen, G.	2017	Technol. Cancer Res. Treat.	16	6	835-849	10.1177/1533034617705056	Liu 2017	Non-NLP Focus
Tolerability and Effectiveness of Exenatide Once Weekly Relative to Basal Insulin Among Type 2 Diabetes Patients of Different Races in Routine Care	Nunes, A.P.; Loughlin, A.M.; Qiao, Q.; Ezzy, S.M.; Yochum, L.; Clifford, C.R.; Gately, R.V.; Dore, D.D.; Seeger, J.D.	2017	Diabetes Ther.	8	6	1349-1364	10.1007/s13300-017-0314-z	Nunes 2017	Non-Gastro/Hepatology Focus
Effects of non-medical switching on outcomes among patients prescribed tumor necrosis factor inhibitors	Gibofsky, A.; Skup, M.; Mittal, M.; Johnson, S.J.; Davis, M.; Chao, J.; Rubin, D.T.	2017	Curr. Med. Res. Opin.	33	11	1945-1953	10.1080/03007995.2017.1375903	Gibofsky 2017	NLP used only as a study adjunct/enabler
Upper gastrointestinal complications following ablation therapy for atrial fibrillation	Park, S.-Y.; Camilleri, M.; Packer, D.; Monahan, K.	2017	Neurogastroenterol. Motil.	29	11		10.1111/nmo.13109	Park 2017	NLP used only as a study adjunct/enabler
Small-calibre percutaneous nephrolithotomy (SC-PCNL). Therapeutic decision algorithm	AmÃ³n Sesmero, J.H.; Cepeda Delgado, M.; de la Cruz MartÃn, B.; Mainez Rodriguez, J.A.; Alonso	2017	Actas Urol. Esp.	41	9	552-561	10.1016/j.acuro.2016.11.006	AmÃ³nSesmero 2017	Non-Gastro/Hepatology Focus

	FernÃ¡ndez, D.; Rodriguez Tesedo, V.; MartÃn Way, D.A.; GutiÃ©rrez Aceves, J.								
Using Pathfinder networks to discover alignment between expert and consumer conceptual knowledge from online vaccine content	Amith, M.; Cunningham, R.; Savas, L.S.; Boom, J.; Schvaneveldt, R.; Tao, C.; Cohen, T.	2017	J. Biomed. Informatics	74		33-45	10.1016/j.jbi.2017.08.007	Amith 2017	Non-Gastro/Hepatology Focus
Identification of patients with congenital hemophilia in a large electronic health record database	Wang, M.; Cyhaniuk, A.; Cooper, D.L.; Iyer, N.N.	2017	J. Blood Med.	8		131-139	10.2147/JBM.S133616	Wang 2017	Non-Gastro/Hepatology Focus
Editorial learning for multimodal data	Zhu, X.; Luo, X.; Xu, C.	2017	Neurocomputing	253		01-May	10.1016/j.neucom.2017.02.080	Zhu 2017	Non-Gastro/Hepatology Focus
Continued statin prescriptions after adverse reactions and patient outcomes: A cohort study	Zhang, H.; Plutzky, J.; Shubina, M.; Turchin, A.	2017	Ann. Intern. Med.	167	4	221-227	10.7326/M16-0838	Zhang 2017	Non-Gastro/Hepatology Focus
Reverse translation of adverse event reports paves the way for de-risking preclinical off-targets	Maciejewski, M.; Lounkine, E.; Whitebread, S.; Farmer, P.; DuMouchel, W.; Shoichet, B.K.; Urban, L.	2017	eLife	6			10.7554/eLife.25818	Maciejewski 2017	Non-Gastro/Hepatology Focus

Identification of people with acquired hemophilia in a large electronic health record database	Wang, M.; Cyhaniuk, A.; Cooper, D.L.; Iyer, N.N.	2017	J. Blood Med.	8		89-97	10.2147/JBM.S136060	Wang 2017	Non-Gastro/Hepatology Focus
Patient Understanding of the Risks and Benefits of Biologic Therapies in Inflammatory Bowel Disease: Insights from a Large-scale Analysis of Social Media Platforms	Martinez, B.; Dailey, F.; Almario, C.V.; Keller, M.S.; Desai, M.; Dupuy, T.; Mosadeghi, S.; Whitman, C.; Lasch, K.; Ursos, L.; Spiegel, B.M.R.	2017	Inflammatory Bowel Dis.	23	7	1057-1064	10.1097/MIB.0000000000001110	Martinez 2017	Lack of Validation
Focal Cystic Pancreatic Lesion Follow-up Recommendations After Publication of ACR White Paper on Managing IncidentalÂ Findings	Bobbin, M.D.; Ip, I.K.; Sahni, V.A.; Shinagare, A.B.; Khorasani, R.	2017	J. Am. Coll. Radiol.	14	6	757-764	10.1016/j.jacr.2017.01.044	Bobbin 2017	Non-Gastro/Hepatology Focus
Assigning clinical codes with data-driven concept representation on Dutch clinical free text	Scheurwegs, E.; Luyckx, K.; Luyten, L.; Goethals, B.; Daelemans, W.	2017	J. Biomed. Informatics	69		118-127	10.1016/j.jbi.2017.04.007	Scheurwegs 2017	Non-Gastro/Hepatology Focus
Adverse and Hypersensitivity Reactions to Prescription Nonsteroidal Anti-Inflammatory	Blumenthal, K.G.; Lai, K.H.; Huang, M.; Wallace, Z.S.; Wickner, P.G.; Zhou, L.	2017	J. Allergy Clin. Immunol. Pract.	5	3	737-743.e3	10.1016/j.jaip.2016.12.006	Blumenthal 2017	Non-Gastro/Hepatology Focus

Agents in a Large Health Care System									
Opportunities for developing therapies for rare genetic diseases: Focus on gain-of-function and allostery	Chen, B.; Altman, R.B.	2017	Orphanet J. Rare Dis.	12	1		10.1186/s13023-017-0614-4	Chen 2017	Non-Gastro/Hepatology Focus
Surrogate-assisted feature extraction for high-throughput phenotyping	Yu, S.; Chakrabortty, A.; Liao, K.P.; Cai, T.; Ananthakrishnan, A.N.; Gainer, V.S.; Churchill, S.E.; Szolovits, P.; Murphy, S.N.; Kohane, I.S.	2017	J. Am. Med. Informatics Assoc.	24	e1	e143-e149	10.1093/jamia/ocw135	Yu 2017	Non-Gastro/Hepatology Focus
Down-regulation of miR-146a-5p and its potential targets in hepatocellular carcinoma validated by a TCGA- and GEO-based study	Zhang, X.; Ye, Z.-H.; Liang, H.-W.; Ren, F.-H.; Li, P.; Dang, Y.-W.; Chen, G.	2017	FEBS Open Bio.	7	4	504-521	10.1002/2211-5463.12198	Zhang 2017	NLP used only as a study adjunct/enabler
Use of electronic healthcare records to identify complex patients with atrial fibrillation for targeted intervention	Wang, S.V.; Rogers, J.R.; Jin, Y.; Bates, D.W.; Fischer, M.A.	2017	J. Am. Med. Informatics Assoc.	24	2	339-344	10.1093/jamia/ocw082	Wang 2017	Non-Gastro/Hepatology Focus

Identifying Drug-Induced Liver Illness (DILI) with Computerized Information Extraction: No More Dilly-Dallying	Shen, H.; Monto, A.	2017	Dig. Dis. Sci.	62	3	564-566	10.1007/s10620-016-4359-z	Shen 2017	Review/Perspective Article Only
Efficient visual attention driven framework for key frames extraction from hysteroscopy videos	Muhammad, K.; Sajjad, M.; Lee, M.Y.; Baik, S.W.	2017	Biomed. Signal Process. Control	33		161-168	10.1016/j.bspc.2016.11.011	Muhammad 2017	Non-Gastro/Hepatology Focus
Operational optimization of binary distillation column to achieve product quality using Imperialist Competitive Algorithm (ICA)	Fitriyani, N.; Nahdliyah, S.D.N.; Biyanto, T.R.	2017	Proc. - Int. Annu. Eng. Semin., InAES			112-115	10.1109/INAES.2016.7821917	Fitriyani 2017	Non-Gastro/Hepatology Focus
Multivariate Linear Regression of Symptoms-related Tweets for Infectious Gastroenteritis Scale Estimation	Takeuchi, R.; Iso, H.; Ito, K.; Wakamiya, S.; Aramaki, E.	2017	DDDSM - Int. Workshop Digit. Disease Detect. Soc. Media, Proc. Workshop			18-25		Takeuchi 2017	Risk Factors For Disease Only
Detecting signals of interactions between warfarin and dietary supplements in electronic health records	Fan, Y.; Adam, T.J.; McEwan, R.; Pakhomov, S.V.; Melton, G.B.; Zhang, R.	2017	Stud. Health Technol. Informatics	245		370-374	10.3233/978-1-61499-830-3-370	Fan 2017	Non-Gastro/Hepatology Focus
Down-regulation of MiR-365 as a novel indicator to assess the	He, R.-Q.; Pang, Y.-Y.; Zhang, R.; Liang, H.-W.; Li, C.-Y.; Ma, J.;	2017	Int. J. Clin. Exp. Pathol.	10	9	9164-9176		He 2017	Non-NLP Focus

progression and metastasis of hepatocellular carcinoma	Feng, Z.-B.; Peng, Z.-G.; Chen, G.								
Improving a full-text search engine: The importance of negation detection and family history context to identify cases in a biomedical data warehouse	Garcelon, N.; Neuraz, A.; Benoit, V.; Salomon, R.; Burgun, A.	2017	J. Am. Med. Informatics Assoc.	24	3	607-613	10.1093/jamia/ocw144	Garcelon 2017	Risk Factors For Disease Only
Percutaneous nephrolithotomy (PCNL): Conditions for a safe performance	DÃaz, P.A.E.; La Riva, P.E.E.; Escovar, F.P.R.; LÃ³pez, M.E.E.; LÃ³pez, M.X.E.; Arias, M.R.C.; Franco, A.	2017	Arch. Esp. Urol.	70	1	173-195		DÃaz 2017	Non-Gastro/Hepatology Focus
Automatic learning of medical text annotation rules â€“ A case study on endoscopies	Slavescu, R.R.; Oltean, M.N.; Torok, A.P.; Slavescu, K.C.	2017	IFMBE Proc.	59		248-251	10.1007/978-3-319-52875-5_53	Slavescu 2017	Non-NLP Focus
From big data to diagnosis and prognosis: Gene expression signatures in liver hepatocellular carcinoma	Yang, H.; Zhang, X.; Cai, X.-Y.; Wen, D.-Y.; Ye, Z.-H.; Liang, L.; Zhang, L.; Wang, H.-L.; Chen, G.; Feng, Z.-B.	2017	PeerJ	2017	3		10.7717/peerj.3089	Yang 2017	NLP used only as a study adjunct/enabler
Outcome of endoscopic trans-ethmosphenoid optic canal decompression combined with	Yu, B.; Gong, C.; Ma, Y.-J.; Wu, W.-C.	2016	Intern. Eye Sci.	16	11	1981-1987	10.3980/j.issn.1672-5123.2016.11.01	Yu 2016	Non-Gastro/Hepatology Focus

steroid and nerve growth factor therapy for short-time traumatic optic neuropathy									
Text Mining Genotype-Phenotype Relationships from Biomedical Literature for Database Curation and Precision Medicine	Singhal, A.; Simmons, M.; Lu, Z.	2016	PLoS Comput. Biol.	12	11		10.1371/journal.pcbi.1005017	Singhal 2016	Non-Gastro/Hepatology Focus
Lower expressed mir-198 and its potential targets in hepatocellular carcinoma: A clinicopathological and in silico study	Huang, W.-T.; Wang, H.-L.; Yang, H.; Ren, F.-H.; Luo, Y.-H.; Huang, C.-Q.; Liang, Y.-Y.; Liang, H.-W.; Chen, G.; Dang, Y.-W.	2016	OncoTargets Ther.	9		5163-5180	10.2147/OTT.S108828	Huang 2016	NLP used only as a study adjunct/enabler
Call Case Dashboard: Tracking R1 Exposure to High-Acuity Cases Using Natural Language Processing	Kelahan, L.C.; Fong, A.; Ratwani, R.M.; Filice, R.W.	2016	J. Am. Coll. Radiol.	13	8	988-991	10.1016/j.jacr.2016.03.012	Kelahan 2016	Non-Gastro/Hepatology Focus
An encapsulation of gene signatures for hepatocellular carcinoma, microRNA-132 predicted target genes and the corresponding overlaps	Zhang, X.; Tang, W.; Chen, G.; Ren, F.; Liang, H.; Dang, Y.; Rong, M.	2016	PLoS ONE	11	7		10.1371/journal.pone.0159498	Zhang 2016	Non-NLP Focus

Characteristics of chronic megacolon among patients diagnosed with multiple endocrine neoplasia type 2B	Gibbons, D.; Camilleri, M.; Nelson, A.D.; Eckert, D.	2016	United Eur. Gastroenterol. J.	4	3	449-454	10.1177/2050640615611630	Gibbons 2016	NLP used only as a study adjunct/enabler
Timing of endoscopic surgical decompression in traumatic optic neuropathy: a systematic review of the literature	Dhaliwal, S.S.; Sowerby, L.J.; Rotenberg, B.W.	2016	Int. Forum Allergy Rhinol.	6	6	661-667	10.1002/alr.21706	Dhaliwal 2016	Non-Gastro/Hepatology Focus
The utility of web mining for epidemiological research: Studying the association between parity and cancer risk	Tourassi, G.; Yoon, H.-J.; Xu, S.; Han, X.	2016	J. Am. Med. Informatics Assoc.	23	3	588-595	10.1093/jamia/ocv141	Tourassi 2016	Non-Gastro/Hepatology Focus
Long-Term Outcomes after Proton Beam Therapy for Sinonasal Squamous Cell Carcinoma	Russo, A.L.; Adams, J.A.; Weyman, E.A.; Busse, P.M.; Goldberg, S.I.; Varvares, M.; Deschler, D.D.; Lin, D.T.; Delaney, T.F.; Chan, A.W.	2016	Int. J. Radiat. Oncol. Biol. Phys.	95	1	368-376	10.1016/j.ijrobp.2016.02.042	Russo 2016	Non-Gastro/Hepatology Focus
Heterogeneity of non-cancerous liver parenchyma on gadoxetic acid-enhanced MRI: An imaging biomarker for hepatocellular carcinoma development in	Asayama, Y.; Nishie, A.; Ishigami, K.; Ushijima, Y.; Takayama, Y.; Okamoto, D.; Fujita, N.; Morita, K.;	2016	Clin. Radiol.	71	5	432-437	10.1016/j.crad.2016.01.023	Asayama 2016	Non-NLP Focus

chronic liver disease	Obara, M.; Honda, H.								
Predictive Parameters of Symptomatic Hematochezia Following 5-Fraction Gantry-Based SABR in Prostate Cancer This study was presented in part at the 29th Canadian Association of Radiation Oncology Annual Scientific Meeting. Kelowna, BC, September 9-12, 201	Musunuru, H.B.; Davidson, M.; Cheung, P.; Vesprini, D.; Liu, S.; Chung, H.; Chu, W.; Mamedov, A.; Ravi, A.; D'Alimonte, L.; Commisso, K.; Helou, J.; Deabreu, A.; Zhang, L.; Loblaw, A.	2016	Int. J. Radiat. Oncol. Biol. Phys.	94	5	1043-1051	10.1016/j.ijrobp.2015.12.010	Musunuru 2016	Non-NLP Focus
Use of data mining at the food and drug administration	Duggirala, H.J.; Tonning, J.M.; Smith, E.; Bright, R.A.; Baker, J.D.; Ball, R.; Bell, C.; Bright-Ponte, S.J.; Botsis, T.; Bouri, K.; Boyer, M.; Burkhart, K.; Steven Condrey, G.; Chen, J.J.; Chirtel, S.; Filice, R.W.; Francis, H.; Jiang, H.; Levine, J.; Martin, D.; Oladipo, T.;	2016	J. Am. Med. Informatics Assoc.	23	2	428-434	10.1093/jamia/ocv063	Duggirala 2016	Non-Gastro/Hepatology Focus

	O'Neill, R.; Palmer, L.A.M.; Paredes, A.; Rochester, G.; Sholtes, D.; Szarfman, A.; Wong, H.-L.; Xu, Z.; Kass-Hout, T.								
Development and Validation of an Algorithm to Identify Nonalcoholic Fatty Liver Disease in the Electronic Medical Record	Corey, K.E.; Kartoun, U.; Zheng, H.; Shaw, S.Y.	2016	Dig. Dis. Sci.	61	3	913-919	10.1007/s10620-015-3952-x	Corey 2016	NLP used only as a study adjunct/enabler
The utility of including pathology reports in improving the computational identification of patients	Chen, W.; Huang, Y.; Boyle, B.; Lin, S.	2016	J. Pathol. Inform.	7	1		10.4103/2153-3539.194838	Chen 2016	Non-Gastro/Hepatology Focus
Cross-lingual transfer of correlations between parts of speech and gaze features	Keller, F.; Barrett, M.; SÃ¸gaard, A.	2016	COLING - Int. Conf. Comput. Linguist., Proc. COLING : Tech. Papers			1330-1339		Keller 2016	Non-Gastro/Hepatology Focus
Wireless capsule endoscopy video summarization: A learning approach based on Siamese neural network and support vector machine	Chen, J.; Zou, Y.; Wang, Y.	2016	Proc. Int. Conf. Pattern Recognit.	0		1303-1308	10.1109/ICPR.2016.7899817	Chen 2016	Non-NLP Focus
Weakly supervised part-of-speech	Barrett, M.; Bingel, J.; Keller, F.; SÃ¸gaard, A.	2016	Annu. Meet. Assoc. Comput. Linguist., ACL - Short Pap.			579-584	10.18653/v1/p16-2094	Barrett 2016	Non-Gastro/Hepatology Focus

tagging using eye-tracking data									
The Outcome of Endoscopic Transethmosphenoid Optic Canal Decompression for Indirect Traumatic Optic Neuropathy with No-Light-Perception	Yu, B.; Ma, Y.; Tu, Y.; Wu, W.	2016	J. Ophthalmol.	2016			10.1155/2016/6492858	Yu 2016	Non-Gastro/Hepatology Focus
Natural Language Processing Based Instrument for Classification of Free Text Medical Records	Khachidze, M.; Tsintsadze, M.; Archuadze, M.	2016	BioMed Res. Int.	2016			10.1155/2016/8313454	Khachidze 2016	Non-Gastro/Hepatology Focus
Dietary nanosized Lactobacillus plantarum enhances the anticancer effect of kimchi on azoxymethane and dextran sulfate sodium-induced colon cancer in C57BL/6J mice	Lee, H.A.; Kim, H.; Lee, K.-W.; Park, K.-Y.	2016	J. Environ. Pathol. Toxicol. Oncol.	35	2	147-159	10.1615/JEnvironPatholToxicolOncol.2016015633	Lee 2016	Non-NLP Focus
Predictors of Locoregional Failure and Impact on Overall Survival in Patients with Resected Exocrine Pancreatic Cancer	Merrell, K.W.; Haddock, M.G.; Quevedo, J.F.; Harmsen, W.S.; Kendrick, M.L.; Miller, R.C.; Hallemeier, C.L.	2016	Int. J. Radiat. Oncol. Biol. Phys.	94	3	561-570	10.1016/j.ijrobp.2015.11.003	Merrell 2016	Non-NLP Focus
Cliniciansâ€™ reports in electronic health records versus patientsâ€™	Topaz, M.; Lai, K.; Dhopeshwarkar, N.; Seger, D.L.; Saâ€™Adon, R.;	2015	Drug Saf.	39	3	243-250	10.1007/s40264-015-0381-x	Topaz 2015	Non-Gastro/Hepatology Focus

concerns in social media: A pilot study of adverse drug reactions of aspirin and atorvastatin	Goss, F.; Rozenblum, R.; Zhou, L.								
Finding text-supported gene-to-disease Co-appearances with MOPED-digger	Kolker, E.; Janko, I.; Montague, E.; Higdon, R.; Stewart, E.; Choiniere, J.; Lai, A.; Eckert, M.; Broomall, W.; Kolker, N.	2015	OMICS J. Integr. Biol.	19	12	754-756	10.1089/omi.2015.0151	Kolker 2015	Non-Gastro/Hepatology Focus
Dead Nano-Sized Lactobacillus plantarum Inhibits Azoxymethane/Dextran Sulfate Sodium-Induced Colon Cancer in Balb/c Mice	Lee, H.A.; Kim, H.; Lee, K.-W.; Park, K.-Y.	2015	J. Med. Food	18	12	1400-1405	10.1089/jmf.2015.3577	Lee 2015	Non-Gastro/Hepatology Focus
Erratum : Multi-center colonoscopy quality measurement utilizing natural language processing (American Journal of Gastroenterology (2015) 110 (543-552) DOI: 10.1038/ajg.2015.51	Imler, T.D.; Morea, J.; Kahi, C.; Cardwell, J.; Johnson, C.S.; Xu, H.; Ahnen, D.; Antaki, F.; Ashley, C.; Baffy, G.; Cho, I.; Dominitz, J.; Hou, J.; Korsten, M.; Nagar, A.; Promrat, K.; Robertson, D.; Saini, S.; Shergill, A.; Smalley, W.; Imperiale, T.F.	2015	Am. J. Gastroenterol.	110	12	1743	10.1038/ajg.2015.349	Imler 2015	Part of Larger Study

Identifying risk factors for heart disease over time: Overview of 2014 i2b2/UTHealth shared task Track 2	Stubbs, A.; Kotfila, C.; Xu, H.; Uzuner, Ã–.	2015	J. Biomed. Informatics	58		S67-S77	10.1016/j.jbi.2015.07.001	Stubbs 2015	Non-Gastro/Hepatology Focus
RFA-cut: Semi-automatic segmentation of radiofrequency ablation zones with and without needles via optimal s-t-cuts	Egger, J.; Busse, H.; Brandmaier, P.; Seider, D.; Gawlitza, M.; Strocka, S.; Voglreiter, P.; Dokter, M.; Hofmann, M.; Kainz, B.; Chen, X.; Hann, A.; Boechat, P.; Yu, W.; Freisleben, B.; Alhonnoro, T.; Pollari, M.; Moche, M.; Schmalstieg, D.	2015	Proc. Annu. Int. Conf. IEEE Eng. Med. Biol. Soc. EMBS	2015-November		2423-2429	10.1109/EMBC.2015.7318883	Egger 2015	Non-NLP Focus
Proceedings - 2015 IEEE 11th International Conference on Intelligent Computer Communication and Processing, ICCP 2015		2015	Proc. - IEEE Int. Conf. Intell. Comput. Commun. Process., ICCP						Non-Gastro/Hepatology Focus
Patient Electronic Health Records as a Means to Approach Genetic Research in Gastroenterology	Ananthakrishnan, A.N.; Lieberman, D.	2015	Gastroenterology	149	5	1134-1137	10.1053/j.gastro.2015.06.005	Ananthakrishnan 2015	Review/Perspective Article Only
Effect of Nanometric Lactobacillus plantarum in Kimchi on Dextran Sulfate Sodium-	Lee, H.A.; Bong, Y.-J.; Kim, H.; Jeong, J.-K.; Kim, H.-Y.; Lee, K.-W.; Park, K.-Y.	2015	J. Med. Food	18	10	1073-1080	10.1089/jmf.2015.3509	Lee 2015	Non-NLP Focus

Induced Colitis in Mice									
The impact of exclusion criteria on a physician's adenoma detection rate	Marcondes, F.O.; Dean, K.M.; Schoen, R.E.; Leffler, D.A.; Rose, S.; Morris, M.; Mehrotra, A.	2015	Gastrointest. Endosc.	82	4	668-675	10.1016/j.gie.2014.12.056	Marcondes 2015	Non-NLP Focus
Public reporting of colonoscopy quality is associated with an increase in endoscopist adenoma detection rate	Abdul-Baki, H.; Schoen, R.E.; Dean, K.; Rose, S.; Leffler, D.A.; Kuganeswaran, E.; Morris, M.; Carrell, D.; Mehrotra, A.	2015	Gastrointest. Endosc.	82	4	676-682	10.1016/j.gie.2014.12.058	Abdul-Baki 2015	NLP used only as a study adjunct/enabler
Continuing Medical Education Exam: September 2015	Buxbaum, J.; Ravi, K.; Ross, W.; Weston, B.; Iyer, P.G.; Schwartz, D.; Wallace, M.B.	2015	Gastrointest. Endosc.	82	3	557-557.e5	10.1016/j.gie.2015.07.022	Buxbaum 2015	Review/Perspective Article Only
Natural Language Processing and the Promise of Big Data: Small Step Forward, but Many Miles to Go	Maddox, T.M.; Matheny, M.A.	2015	Circ. Cardiovasc. Qual. Outcomes	8	5	463-465	10.1161/CIRCOUTCOMES.115.002125	Maddox 2015	Review/Perspective Article Only
Balancing opioid-induced gastrointestinal side effects with pain management: Insights from the online community	Whitman, C.B.; Reid, M.W.; Arnold, C.; Patel, H.; Ursos, L.; Sa'adon, R.; Pourmorady, J.; Spiegel, B.M.R.	2015	J. Opioid Manage.	11	5	383-391	10.5055/jom.2015.0288	Whitman 2015	NLP used only as a study adjunct/enabler
Measuring the quality of colonoscopy: Where are we	Imler, T.D.; Imperiale, T.F.	2015	Gastrointest. Endosc.	82	3	520-522	10.1016/j.gie.2015.03.1961	Imler 2015	Review/Perspective Article Only

now and where are we going?									
Methods to develop an electronic medical record phenotype algorithm to compare the risk of coronary artery disease across 3 chronic disease cohorts	Liao, K.P.; Ananthakrishnan, A.N.; Kumar, V.; Xia, Z.; Cagan, A.; Gainer, V.S.; Goryachev, S.; Chen, P.; Savova, G.K.; Agniel, D.; Churchill, S.; Lee, J.; Murphy, S.N.; Plenge, R.M.; Szolovits, P.; Kohane, I.; Shaw, S.Y.; Karlson, E.W.; Cai, T.	2015	PLoS ONE	10	8		10.1371/journal.pone.0136651	Liao 2015	Non-Gastro/Hepatology Focus
Prevalence of Inflammatory Bowel Disease among Patients with Autism Spectrum Disorders	Doshi-Velez, F.; Avillach, P.; Palmer, N.; Bousvaros, A.; Ge, Y.; Fox, K.; Steinberg, G.; Spettell, C.; Juster, I.; Kohane, I.	2015	Inflammatory Bowel Dis.	21	10	2281-2288	10.1097/MIB.0000000000000502	Doshi-Velez 2015	Non-NLP Focus
The prevalence of problem opioid use in patients receiving chronic opioid therapy: Computer-assisted review of electronic health record clinical notes	Palmer, R.E.; Carrell, D.S.; Cronkite, D.; Saunders, K.; Gross, D.E.; Masters, E.; Donevan, S.; Hylan, T.R.; Von Kroff, M.	2015	Pain	156	7	1208-1214	10.1097/j.pain.0000000000000145	Palmer 2015	Non-Gastro/Hepatology Focus

The use of big data in transfusion medicine	Pendry, K.	2015	Transfus. Med.	25	3	129-137	10.1111/tme.12223	Pendry 2015	Review/Perspective Article Only
Nucleobindin-1 encodes a nesfatin-1-like peptide that stimulates insulin secretion	Ramesh, N.; Mohan, H.; Unniappan, S.	2015	Gen. Comp. Endocrinol.	216		182-189	10.1016/j.ygcen.2015.04.011	Ramesh 2015	Non-Gastro/Hepatology Focus
The medical history: Form and function	Lafsky, R.D.	2015	Gastroenterology	148	5	1079	10.1053/j.gastro.2015.01.049	Lafsky 2015	Review/Perspective Article Only
Development of phenotype algorithms using electronic medical records and incorporating natural language processing	Liao, K.P.; Cai, T.; Savova, G.K.; Murphy, S.N.; Karlson, E.W.; Ananthakrishnan, A.N.; Gainer, V.S.; Shaw, S.Y.; Xia, Z.; Szolovits, P.; Churchill, S.; Kohane, I.	2015	BMJ (Online)	350			10.1136/bmj.h1885	Liao 2015	Non-Gastro/Hepatology Focus
Automated curation of gene name normalization results using the Konstanz information miner	Zwick, M.	2015	J. Biomed. Informatics	53		58-64	10.1016/j.jbi.2014.08.016	Zwick 2015	Non-Gastro/Hepatology Focus
Validating estimates of prevalence of non-communicable diseases based on household surveys: The symptomatic diagnosis study	James, S.L.; Romero, M.; RamÃrez-Villalobos, D.; GÃ³mez, S.; Pierce, K.; Flaxman, A.; Serina, P.; Stewart, A.; Murray, C.J.L.;	2015	BMC Med.	13	1		10.1186/s12916-014-0245-8	James 2015	Non-Gastro/Hepatology Focus

	Gakidou, E.; Lozano, R.; Hernandez, B.								
In-depth annotation for patient level liver cancer staging	Yim, W.-W.; Kwan, S.; Yetisgen, M.	2015	EMNLP - Int. Workshop Health Text Min. Inf. Anal., Proc. Workshop			01-Nov		Yim 2015	Non-Gastro/Hepatology Focus
Reviewing 741 patients records in two hours with FASTVISU	EscudiÃ©, J.-B.; Jannot, A.-S.; Zapletal, E.; Cohen, S.; Malamut, G.; Burgun, A.; Rance, B.	2015	AMIA Annu Symp Proc	2015		553-559		EscudiÃ© 2015	Non-Gastro/Hepatology Focus
Syndromic Surveillance of Infectious Diseases meets Molecular Epidemiology in a Workflow and Phylogeographic Application	Janies, D.; Witter, Z.; Gibson, C.; Kraft, T.; Senturk, I.F.; Ã‡atalyÃ¼rek, U.	2015	Stud. Health Technol. Informatics	216		766-770	10.3233/978-1-61499-564-7-766	Janies 2015	Non-Gastro/Hepatology Focus
Identification of Patients with Family History of Pancreatic Cancer-Investigation of an NLP System Portability	Mehrabi, S.; Krishnan, A.; Roch, A.M.; Schmidt, H.; Li, D.; Kesterson, J.; Beesley, C.; Dexter, P.; Schmidt, M.; Palakal, M.; Liu, H.	2015	Stud. Health Technol. Informatics	216		604-608	10.3233/978-1-61499-564-7-604	Mehrabi 2015	Non-NLP Focus
Gastrointestinal stromal tumor: Optimizing the use of cross-sectional chest imaging during follow-up	Shinagare, A.B.; Ip, I.K.; Lacson, R.; Ramaiya, N.H.; George, S.; Khorasani, R.	2015	Radiology	274	2	395-404	10.1148/radiol.14132456	Shinagare 2015	NLP used only as a study adjunct/enabler

Using machine learning to identify major shifts in human gut microbiome protein family abundance in disease	M. Yazdani; B. C. Taylor; J. W. Debelius; W. Li; R. Knight; L. Smarr	2016	2016 IEEE International Conference on Big Data (Big Data)			1272-1280	10.1109/BigData.2016.7840731	M 2016	Risk Factors For Disease Only
A Comparitive Study of Clasification Liver Dysfunction with Machine Learning	S. Thaiparnit; N. Chumuang; M. Ketcham	2018	2018 International Joint Symposium on Artificial Intelligence and Natural Language Processing (iSAI-NLP)			01-Apr	10.1109/iSAI-NLP.2018.8692808	S 2018	Non-NLP Focus
A Computational Framework to Analyze the Associations Between Symptoms and Cancer Patient Attributes Post Chemotherapy Using EHR Data	X. Luo; P. Gandhi; S. Storey; Z. Zhang; Z. Han; K. Huang	2021	IEEE Journal of Biomedical and Health Informatics	25	11	4098-4109	10.1109/JBHI.2021.3117238	X 2021	Non-Gastro/Hepatology Focus
Building annotation rules for text description of endoscopies in romanian - an NLP-free approach	R. R. Slavescu; A. Bali; K. C. Slavescu	2015	2015 IEEE International Conference on Intelligent Computer Communication and Processing (ICCP)			19-25	10.1109/ICCP.2015.7312600	R 2015	Non-NLP Focus
Domain over size: Clinical ELECTRA surpasses general BERT for bleeding site classification in the free text of electronic health records	J. S. Pedersen; M. S. Laursen; C. Soguero-Ruiz; T. R. Savarimuthu; R. S. Hansen; P. J. Vinholt	2022	2022 IEEE-EMBS International Conference on Biomedical and Health Informatics (BHI)			01-Apr	10.1109/BHI56158.2022.9926955	J 2022	Non-Gastro/Hepatology Focus

Research on Compliance Supervision Data Analysis Model Based on Mass Chat Records in the Inter-Bank Market	Y. Wang; Y. Li; T. Wu	2021	2021 IEEE 2nd International Conference on Big Data, Artificial Intelligence and Internet of Things Engineering (ICBAIE)			368-380	10.1109/ICBAIE52039.2021.9389994	Y 2021	Non-Gastro/Hepatology Focus
Traditional Chinese medicine prescription mining based on abstract text	D. Xie; W. Pei; W. Zhu; X. Li	2017	2017 IEEE 19th International Conference on e-Health Networking, Applications and Services (Healthcom)			01-May	10.1109/HealthCom.2017.8210822	D 2017	Non-Gastro/Hepatology Focus
A First Step Towards NLP from Digitized Manuscripts: Virtual Restoration	F. Debole; M. Ranif; E. Salerno; P. Savino; A. Tonazzini	2018	2018 IEEE 5th International Congress on Information Science and Technology (CiSt)			188-193	10.1109/CIST.2018.8596494	F 2018	Non-Gastro/Hepatology Focus
An AI-Based Approach to Analysis of Medical Innovation: A Case of Ucnerative Colitis Treatment	K. Yamasaki; R. Hosoya	2019	2019 Portland International Conference on Management of Engineering and Technology (PICMET)			01-May	10.23919/PICMET.2019.8893669	K 2019	Review/Perspective Article Only
PhysioVec: IoT Biosignal Based Search Engine for Gastrointestinal Health	Y. Huang; I. Song	2022	2022 7th International Conference on Computational Intelligence and Applications (ICCIA)			230-236	10.1109/ICCIA55271.2022.9828432	Y 2022	Non-NLP Focus
Combining deep learning and fuzzy logic to predict rare ICD-10 codes from clinical notes	T. Chomutare; A. Budrionis; H. Dalianis	2022	2022 IEEE International Conference on Digital Health (ICDH)			163-168	10.1109/ICDH55609.2022.00033	T 2022	Non-Gastro/Hepatology Focus
Standardized Assessment of Automatic Segmentation of White Matter	H. J. Kuijf; J. M. Biesbroek; J. De Bresser; R. Heinen; S. Andermatt; M.	2019	IEEE Transactions on Medical Imaging	38	11	2556-2568	10.1109/TMI.2019.2905770	H 2019	Non-Gastro/Hepatology Focus

Hyperintensities and Results of the WMH Segmentation Challenge	Bento; M. Berseth; M. Belyaev; M. J. Cardoso; A. Casamitjana; D. L. Collins; M. Dadar; A. Georgiou; M. Ghafoorian; D. Jin; A. Khademi; J. Knight; H. Li; X. LladÃ³; M. Luna; Q. Mahmood; R. McKinley; A. Mehrtash; S. Ourselin; B. -Y. Park; H. Park; S. H. Park; S. Pezold; E. Puybareau; L. Rittner; C. H. Sudre; S. Valverde; V. Vilaplana; R. Wiest; Y. Xu; Z. Xu; G. Zeng; J. Zhang; G. Zheng; C. Chen; W. van der Flier; F. Barkhof; M. A. Viergever; G. J. Biessels								
Saying What You're Looking For: Linguistics Meets Video Search	D. P. Barrett; A. Barbu; N. Siddharth; J. M. Siskind	2016	IEEE Transactions on Pattern Analysis and Machine Intelligence	38	10	2069-2081	10.1109/TPAMI.2015.2505297	D 2016	Non-Gastro/Hepatology Focus

Language Model Supervision for Handwriting Recognition Model Adaptation	C. Tensmeyer; C. Wigington; B. Davis; S. Stewart; T. Martinez; W. Barrett	2018	2018 16th International Conference on Frontiers in Handwriting Recognition (ICFHR)			133-138	10.1109/ICFHR-2018.2018.00032	C 2018	Non-Gastro/Hepatology Focus
Driving Under the Influence (of Language)	D. P. Barrett; S. A. Bronikowski; H. Yu; J. M. Siskind	2018	IEEE Transactions on Neural Networks and Learning Systems	29	7	2668-2683	10.1109/TNNLS.2017.2693278	D 2018	Non-Gastro/Hepatology Focus
Phenonizer: A fine-grained phenotypic named entity recognizer for Chinese clinical texts	Q. Zou; K. Yang; K. Chang; X. Zhang; X. Li; X. Zhou	2021	2021 IEEE International Conference on Bioinformatics and Biomedicine (BIBM)			3963-3970	10.1109/BIBM52615.2021.9669766	Q 2021	Non-Gastro/Hepatology Focus
Operational optimization of binary distillation column to achieve product quality using Imperialist Competitive Algorithm (ICA)	N. Fitriyani; S. D. N. Nahdliyah; T. R. Biyanto	2016	2016 6th International Annual Engineering Seminar (InAES)			112-115	10.1109/INAES.2016.7821917	N 2016	Non-Gastro/Hepatology Focus
A database for degraded Arabic historical manuscripts	A. Sulaiman; K. Omar; M. F. Nasrudin	2017	2017 6th International Conference on Electrical Engineering and Informatics (ICEEI)			01-Jun	10.1109/ICEEI.2017.8312375	A 2017	Non-Gastro/Hepatology Focus
Grantha script recognition from ancient palm leaves using histogram of orientation shape context	V. A. Raj; R. L. Jyothi; A. Anilkumar	2017	2017 International Conference on Computing Methodologies and Communication (ICCMC)			790-794	10.1109/ICCMC.2017.8282574	V 2017	Non-Gastro/Hepatology Focus
Denoising Heterogeneous Malayalam Document Images	A. S; G. Z. V; H. M; N. Narayanan; A. John	2021	2021 IEEE Madras Section Conference (MASCON)			01-Jun	10.1109/MASCON51689.2021.9563591	A 2021	Non-Gastro/Hepatology Focus

Through Binarization									
BD2K Training Coordinating Center's ERuDIte: The Educational Resource Discovery Index for Data Science	J. L. Ambite; L. Fierro; J. Gordon; G. A. P. C. Burns; F. Geigl; K. Lerman; J. D. Van Horn	2021	IEEE Transactions on Emerging Topics in Computing	9	1	316-328	10.1109/TETC.2019.2903466	J 2021	Non-Gastro/Hepatology Focus
Planning of Power Grasps Using Infinite Program Under Complementary Constraints	Z. Pan; D. Zhang; C. Tu; X. Gao	2022	IEEE Robotics and Automation Letters	7	1	650-657	10.1109/LRA.2021.3130376	Z 2022	Non-Gastro/Hepatology Focus
IEEE Standard for Ethernet		2018	IEEE Std 802.3-2018 (Revision of IEEE Std 802.3-2015)			Jan-00	10.1109/IEEESTD.2018.8457469		Non-Gastro/Hepatology Focus
Uncertainty-aware self-supervised neural network for liver T _{1Ï�} mapping with relaxation constraint	Huang, Chaoxing; Qian, Yurui; Yu, Simon Chun-Ho; Hou, Jian; Jiang, Baiyan; Chan, Queenie; Wong, Vincent Wai-Sun; Chu, Winnie Chiu-Wing; Chen, Weitian	2022	Phys. Med. Biol.	67	22	225019	10.1088/1361-6560/ac9e3e	Huang 2022	Non-NLP Focus
Label-free metabolic imaging of non-alcoholic-fatty-liver-disease (NAFLD) liver by volumetric dynamic optical coherence tomography	Mukherjee, Pradipta; Fukuda, Shinichi; Lukmanto, Donny; Yamashita, Toshiharu; Okada, Kosuke; Makita, Shuichi;	2022	Biomed. Opt. Express	13	7	4071	10.1364/BOE.461433	Mukherjee 2022	Non-NLP Focus

	Abd El-Sadek, Ibrahim; Miyazawa, Arata; Zhu, Lida; Morishita, Rion; Lichtenegger, Antonia; Oshika, Tetsuro; Yasuno, Yoshiaki								
Pulse-echo speed-of-sound imaging using convex probes	Jaeger, Michael; StÃ¤hli, Patrick; Martiartu, Naiara Korta; Yolgunlu, Parisa Salemi; Frappart, Thomas; Fraschini, Christophe; Frenz, Martin	2022	Phys. Med. Biol.	67	21	215016	10.1088/1361-6560/ac96c6	Jaeger 2022	Non-Gastro/Hepatology Focus
RA V-Net: deep learning network for automated liver segmentation	Lee, ZhiQi; Qi, SuMin; Fan, ChongChong; Xie, ZiWei; Meng, Jing	2022	Phys. Med. Biol.	67	12	125022	10.1088/1361-6560/ac7193	Lee 2022	Non-NLP Focus
Realistic Ultrasound Image Synthesis for Improved Classification of Liver Disease	Che, Hui; Ramanathan, Sumana; Foran, David J.; Nosher, John L.; Patel, Vishal M.; Hacihaliloglu, Ilker	2021	Simplifying Medical Ultrasound	129 67		179-188		Che 2021	Non-NLP Focus
Phase-Aberration Correction in Shear-Wave Elastography Imaging Using Local Speed-of-	Chintada, Bhaskara R.; Rau, Richard; Goksel, Orcun	2021	Front. Phys.	9		690385	10.3389/fphy.2021.690385	Chintada 2021	Non-Gastro/Hepatology Focus

Sound Adaptive Beamforming									
Combining a convolutional neural network with autoencoders to predict the survival chance of COVID-19 patients	Khozeimeh, Fahime; Sharifrazi, Danial; Izadi, Navid Hoseini; Joloudari, Javad Hassannataj; Shoeibi, Afshin; Alizadehsani, Roohallah; Gorriz, Juan M.; Hussain, Sadiq; Sani, Zahra Alizadeh; Moosaei, Hossein; Khosravi, Abbas; Nahavandi, Saeid; Islam, Sheikh Mohammed Shariful	2021	Sci Rep	11	1	15343	10.1038/s41598-021-93543-8	Khozeimeh 2021	Non-Gastro/Hepatology Focus
Characterization of frequency-dependent material properties of human liver and its pathologies using an impact hammer	Umut Ozcan, M.; Ocal, Sina; Basdogan, Cagatay; Dogusoy, Gulen; Tokat, Yaman	2011	Medical Image Analysis	15	1	45-52	10.1016/j.media.2010.06.010	UmutOzcan 2011	Non-NLP Focus
A Digital Image Processing Approach for Hepatic Diseases Staging based on	Trombini, Marco; Borro, Paolo; Ziola, Sebastiano; Dellepiane, Silvana	2020	2020 2nd International Conference on Electrical, Control and Instrumentation Engineering (ICECIE)			01-Jun	10.1109/ICECIE50279.2020.9309633	Trombini 2020	Non-NLP Focus

the Glissonâ€™s Capsule									
Fully automatic liver attenuation estimation combing CNN segmentation and morphological operations	Huo, Yuankai; Terry, James G.; Wang, Jiachen; Nair, Sangeeta; Lasko, Thomas A.; Freedman, Barry I.; Carr, J. Jeffery; Landman, Bennett A.	2019	Med. Phys.	46	8	3508-3519	10.1002/mp.13675	Huo 2019	Non-NLP Focus
Machine Learning with Abstention for Automated Liver Disease Diagnosis	Hamid, Kanza; Asif, Amina; Abbasi, Wajid; Sabih, Durre; Minhas, Fayyaz-ul-Amir Afsar	2017	2017 International Conference on Frontiers of Information Technology (FIT)			356-361	10.1109/FIT.2017.00070	Hamid 2017	Non-NLP Focus
Encoding feature supervised UNet++: Redesigning Supervision for liver and tumor segmentation	Cui, Jiahao; Xiao, Ruoxin; Fang, Shiyuan; Pei, Minnan; Yu, Yixuan	2022						Cui 2022	Non-NLP Focus
Efficient liver segmentation with 3D CNN using computed tomography scans	Humady, Khaled; Al-Saeed, Yasmeen; Eladawi, Nabila; Elgarayhi, Ahmed; Elmogy, Mohammed; Sallah, Mohammed	2022						Humady 2022	Non-Gastro/Hepatology Focus
Liver Segmentation using Turbolift Learning for CT and Cone-beam C-	HaseljiÄ‡, Hana; Chatterjee, Soumick; Frysch, Robert; Kulvait,	2022						HaseljiÄ‡ 2022	Non-NLP Focus

arm Perfusion Imaging	VojtÄ›ch; Semshchikov, Vladimir; Hensen, Bennet; Wacker, Frank; BrÃ¼sch, Inga; Werncke, Thomas; Speck, Oliver; NÃ¼rnberger, Andreas; Rose, Georg								
FedNorm: Modality-Based Normalization in Federated Learning for Multi-Modal Liver Segmentation	Bernecker, Tobias; Peters, Annette; Schlett, Christopher L.; Bamberg, Fabian; Theis, Fabian; Rueckert, Daniel; WeiÃŸ, Jakob; Albarqouni, Shadi	2022						Bernecker 2022	Non-NLP Focus
PET CMR$_{glc}$ mapping and ^{1}H MRS show altered glucose uptake and neurometabolic profiles in BDL rats	Mosso, Jessie; Yin, Ting; Poitry-Yamate, Carole; Simicic, Dunja; Lepore, Mario; McLin, ValÃ©rie A.; Braissant, Olivier; Cudalbu, Cristina; Lanz, Bernard	2021						Mosso 2021	Non-Gastro/Hepatology Focus

Pseudo-domains in imaging data improve prediction of future disease status in multi-center studies	Perkonigg, Matthias; Mesenbrink, Peter; Goehler, Alexander; Martic, Miljen; Ba-Ssalamah, Ahmed; Langs, Georg	2021						Perkonigg 2021	Non-Gastro/Hepatology Focus
Accurate and Generalizable Quantitative Scoring of Liver Steatosis from Ultrasound Images via Scalable Deep Learning	Li, Bowen; Tai, Dar-In; Yan, Ke; Chen, Yi-Cheng; Huang, Shiu-Feng; Hsu, Tse-Hwa; Yu, Wan-Ting; Xiao, Jing; Lu, Le; Harrison, Adam P.	2021						Li 2021	Non-NLP Focus
Discovery of temporal structure intricacy in arterial blood pressure waveforms representing acuity of liver transplant and forecasting short term surgical outcome via unsupervised manifold learning	Wang, Shen-Chih; Ting, Chien-Kun; Chen, Cheng-Yen; Liu, Chin-Su; Lin, Niang-Cheng; Loon, Che-Chuan; Wu, Hau-Tieng; Lin, Yu-Ting	2021						Wang 2021	Non-NLP Focus
Unsupervised domain adaptation for cross-modality liver segmentation via joint adversarial learning and self-learning	Hong, Jin; Yu, Simon Chun-Ho; Chen, Weitian	2022						Hong 2022	Non-Gastro/Hepatology Focus

Non-invasive Assessment of Hepatic Venous Pressure Gradient (HVPG) Based on MR Flow Imaging and Computational Fluid Dynamics	Wang, Kexin; Wang, Shuo; Xiong, Minghua; Wang, Chengyan; Wang, He	2021						Wang 2021	Non-NLP Focus
Liver Fibrosis and NAS scoring from CT images using self-supervised learning and texture encoding	Jana, Ananya; Qu, Hui; Minacapelli, Carlos D.; Catalano, Carolyn; Rustgi, Vinod; Metaxas, Dimitris	2021						Jana 2021	Non-NLP Focus
Magnetic Resonance Elastography and Portal Hypertension: Influence of the Portal Venous Flow on the Liver Stiffness	Chatelin, Simon; Pop, Raoul; Giraudeau, CÃ©line; Ambarki, Khalid; Jin, Ning; Severac, FranÃ§ois; Breton, Elodie; Vappou, Jonathan	2020						Chatelin 2020	Non-NLP Focus
Fat and fibrosis as confounding cofactors in viscoelastic measurements of the liver	Poul, Sedigheh S.; Parker, Kevin J.	2020						Poul 2020	Non-NLP Focus
Deep Learning based NAS Score and Fibrosis Stage Prediction from CT and Pathology Data	Jana, Ananya; Qu, Hui; Rattan, Puru; Minacapelli, Carlos D.; Rustgi, Vinod;	2020						Jana 2020	Non-NLP Focus

	Metaxas, Dimitris								
Adversarial attacks on deep learning models for fatty liver disease classification by modification of ultrasound image reconstruction method	Byra, Michal; Styczynski, Grzegorz; Szmigielski, Cezary; Kalinowski, Piotr; Michalowski, Lukasz; Paluszkiewicz, Rafal; Ziarkiewicz-Wroblewska, Bogna; Zieniewicz, Krzysztof; Nowicki, Andrzej	2020						Byra 2020	Non-NLP Focus
Liver Steatosis Segmentation with Deep Learning Methods	Guo, Xiaoyuan; Wang, Fusheng; Teodorou, George; Farris, Alton B.; Kong, Jun	2019						Guo 2019	Non-NLP Focus
Hybrid Adaptive Neuro-Fuzzy Inference System for Diagnosing the Liver Disorders	Rajabi, Mina; Sadeghizadeh, Hajar; Mola-Amini, Zahra; Ahmadyrad, Niloofar	2019						Rajabi 2019	Non-NLP Focus
A Two-Parameter Model for Ultrasonic Tissue Characterization with Harmonic Imaging	Krishnan, Kajoli Banerjee; Nagaraj, Nithin; Singhal, Nitin; Thapar, Shalini; Yadav, Komal	2017						Krishnan 2017	Non-NLP Focus
